# Challenges in Mechanistic Investigation of a Flexible Aminocatalyst as Demonstrated through Enamine Formation

**DOI:** 10.1002/open.202500116

**Published:** 2025-06-25

**Authors:** Irina Osadchuk, Tõnis Kanger

**Affiliations:** ^1^ Department of Chemistry and Biotechnology Tallinn University of Technology Akadeemia tee 15 12618 Tallinn Estonia

**Keywords:** computational chemistry, conformational analyses, noncovalent interactions, organocatalysis, reaction mechanisms

## Abstract

Asymmetric catalysis has become a prominent topic in synthesis over recent decades. Conformational changes in the catalyst core play a significant role in the reaction, determining both its rate and selectivity. This article presents computational studies of enamine formation from cyclohexanone and the tripeptide catalyst Phe–Lys–Phe and considers challenges related to conformational search and the selection of an appropriate level of theory for studying flexible catalysts. This also demonstrates the importance of selecting the initial model system and how reducing the system under study or including the entire system in the model can impact the study's outcome. Furthermore, incorporating a water molecule into the model system significantly reduces the energy of proton transfer. Finally, the catalyst's ability to reorganize plays an important role, since it allows the energy of the transition states to be reduced. Thus, this shows that an alternative reaction pathway is more favorable than the one initially found, and the catalyst's flexibility allows and contributes its conformations to vary at different stages of the reaction.

## Graphical abstract

The enamine formation catalyzed by a flexible asymmetric tripeptide is studied computationally. The catalyst's ability to reorganize plays a crucial role, enabling the reaction and providing multiple pathways for its occurrence. Furthermore, issues related to conformational search, method selection, and model system choices are discussed.

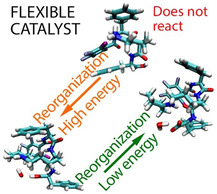

## 1 Introduction

During the last decades, more attention has been paid to the catalyst's flexibility in the design of catalysts. Conformational changes in the catalyst core have been found to be an essential part in forming lower‐energy transition states that participate in the catalytic cycle. In addition to the lock‐and‐key model used for enzymatic reactions for more than a century,^[^ ^1^ ^]^ new explanations considering the flexibility of the catalytic site have been proposed.^[^ ^2^, ^3^ ^]^ Conformational changes are not only obligatory to achieve catalytic activity, but they are also important throughout the catalytic cycle, being responsible for substrate recruitment and product release.^[^ ^4^ ^]^ It has been shown by theoretical calculations^[^ ^5^ ^]^ and experimentally^[^ ^6^ ^]^ that flexibility enhances catalysis and may play a substantial role in metal catalysis.^[^ ^7^ ^]^

Nowadays, asymmetric organocatalysis has become a widely used method in organic synthesis. In 2021, MacMillan and List were awarded the Noble Prize in chemistry for their development of asymmetric organocatalysis.^[^ ^8^ ^]^ In asymmetric organocatalysis, low molecular weight enantiomerically pure compounds catalyze organic reactions. Among these, chiral amino‐catalysts have found a special place due to their ability to form enamines and react stereoselectively with various electrophiles.^[^ ^9^, ^10^ ^]^ For example, Wennemers et al. used enamines derived from tripeptide and carbonyl compounds for the stereoselective Michael addition. It was shown that the β‐turn of the structure of the tripeptide and specific amino acids sequence are necessary for the stereoselective reaction.^[^ ^11^ ^]^ Lee et al.^[^ ^12^ ^]^ concluded that the introduction of an acidic Tf‐amide group in a rigid amine backbone improves the catalytic performance. Wang et al.^[^ ^13^ ^]^ developed a highly diastereo‐ and enantioselective reaction of glycine esters with ketones by anodic oxidation and amino‐organocatalysis.

The high modularity of di‐, tri‐, or tetrapeptidic catalysts enables access to a vast structural diversity of catalysts. Utilizing solid‐phase or combinatorial synthesis facilitates their efficient preparation. Short oligopeptides have been established as highly active organocatalysts, exhibiting exceptional stereo‐, chemo‐, and site‐selectivity in several important C–C and C–heteroatom bond‐forming reactions.

**Scheme 1:**
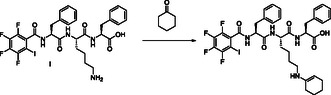
Formation of enamine studied.

The following study is a part of our broader program of studying tripeptide catalysts in asymmetric reactions. In this article, we present only the computational results on the formation of enamine from cyclohexanone and tripeptide Phe‐Lys‐Phe **I** (**Scheme** **1**, for mechanism, see Scheme S1, Supporting Information) where a primary amino group of Lys enables the formation of nucleophilic enamine needed for the reactions with various electrophiles. Characteristic features of the tripeptide studied include a halogen bond donor moiety, a number of functional groups capable of forming hydrogen bonds, and a large number of rotating bonds, making the computational study challenging. Therefore, special attention in this work is paid to the choice of computational methods and the different approaches used to stabilize the system.

## 2 Results and Discussion

### 2.1 Choice of Method

In computational chemistry, the accuracy of the results depends on the level of theory chosen.^[^ ^14^, ^15^ ^]^ The conformation of our catalyst is determined by noncovalent interactions (π–π stacking, hydrogen bonds, and potential halogen bonds). Thus, a method is needed that handles noncovalent interactions well. Among all the noncovalent interactions listed above, the halogen bond is the most complicated to model, since it is a combination of different bonding models (dispersion, electrostatics, charge transfer, etc.). Herein, the molecule under study is flexible; thus, the first step of the study should be a conformational search to determine the most probable conformer(s). At this stage, some cost‐effective method capable of describing noncovalent interactions is needed. Molecular mechanics (MMs) are fast methods, which can be used to study large molecules.^[^ ^16^, ^17^, ^18^ ^]^ However, classical MM is unable to correctly describe halogen bonds. AMBER,^[^ ^19^ ^]^ CHARMM,^[^ ^20^ ^]^ and OPLS^[^ ^16^ ^]^ force fields incorporate a virtual site with a partial positive charge to mimic quantum chemical effects for a σ‐hole description.^[^ ^17^, ^18^, ^19^ ^]^ However, the number of systems studied was limited, and there may be a problem with the transferability of the halogen parameters and possibly the need for their reparameterization.^[^ ^21^, ^22^, ^23^ ^]^

Modern semiempirical methods (SQM) can also be classified as cost‐effective. They are accurate when used for the same type of molecules that were used in the parameterization of a method.^[^ ^24^, ^25^ ^]^ Therefore, most SQM methods have difficulties describing halogen bonds.^[^ ^19^, ^26^ ^]^ However, several modern SQM methods (PM*x*,^[^ ^24^, ^25^, ^27^, ^28^, ^29^ ^]^ DFTB,^[^ ^30^, ^31^, ^32^, ^33^, ^34^ ^]^ and GFN*n*‐xTB^[^ ^35^, ^36^ ^]^) were parameterized with consideration hydrogen bonds and halogen bonds. All SQM have their advantages and disadvantages.^[^ ^23^, ^35^, ^37^, ^38^, ^39^ ^]^ We decided that in our investigation, it would be more reliable to use one of the SQM methods.

The density functional theory (DFT) approach is generally more accurate than SQM or MM methods, but its accuracy depends on the level of theory used (functional, dispersion correction, basis set).^[^ ^40^, ^41^ ^]^ M06‐2X^[^ ^42^ ^]^ is the recommended functional for describing halogen bonding and other noncovalent interactions, according to several benchmark studies.^[^ ^40^, ^41^, ^43^, ^44^, ^45^ ^]^ The M06‐2X functional was initially designed for main group thermochemistry calculations and the description of noncovalent interactions. Several benchmark studies have shown that better performance in halogen bond description is achieved by functionals that implicitly or explicitly account for dispersion corrections.^[^ ^23^, ^45^ ^]^ At the same time, a benchmark study revealed that the D3 dispersion correction^[^ ^46^ ^]^ changed the accuracy insignificantly,^[^ ^41^ ^]^ so we decided not to use the dispersion correction. The size of the basis set also plays an important role in the accuracy of a method. Although large basis sets are preferred for halogen bond calculations with the DFT approach,^[^ ^40^, ^47^, ^48^ ^]^ double‐zeta basis sets are also suitable, especially for large systems.^[^ ^48^, ^49^ ^]^ Moreover, since relativistic effects are important in halogen bond description, basis sets with electronic core potentials (ECPs) are recommended.^[^ ^40^ ^]^ Based on this, we chose def2‐SVP^[^ ^50^ ^]^ and def2‐TZVPP^[^ ^50^ ^]^ for our calculations, which include ECP and account for scalar relativistic effects. Additionally, other noncovalent interactions, such as hydrogen bonding, dipole and Van der Waals interactions determine the geometry of the studied catalyst. The M06‐2X functional, including its combination with double‐zeta basis sets, has shown good performance in describing noncovalent interactions.^[^ ^14^, ^15^, ^51^, ^52^, ^53^, ^54^ ^]^ However, our main goal was to study the reaction mechanism of enamine formation. Therefore, the method used should also be accurate in predicting barrier heights, and the M06‐2X functional has been recommended for this in several benchmark studies.^[^ ^14^, ^15^, ^52^, ^55^, ^56^ ^]^ Exactly, the M06‐2X/def2‐TZVPP//M06‐2X/def2‐SVP level of theory is also frequently used for reaction mechanism studies by other research groups.^[^ ^57^, ^58^, ^59^, ^60^ ^]^

A benchmark study by Robidas and Legault^[^ ^61^ ^]^ for molecular iodine using different functionals and solvent models demonstrated that all continuum solvent models have comparable accuracy for neutral molecules. In contrast, spectroscopic studies have shown a systematic improvement in the energy calculated using the solvation model based on density solvent (SMD) model compared to polarizable continuum model (PCM).^[^ ^62^, ^63^ ^]^ There is also evidence that the accuracy of continuum solvent models depends on the solvent.^[^ ^64^ ^]^

In summary, for the conformational study, we decided to use one of the modern SQM methods. For further reaction mechanism modeling, we chose a more accurate DFT approach—the M06‐2X functional with Karlsruhe basis sets, CPCM continuum solvent models for geometry optimization and SMD—for single‐point calculations.

### 2.2 Conformational Search Problems

It is crucial to find the correct conformation of a molecule, as its reactivity depends on its geometry, and even small structural changes can significantly affect the molecule's properties.^[^ ^65^, ^66^, ^67^ ^]^ Thus, exploring conformational space is the first step in most computational studies.^[^ ^14^, ^68^ ^]^ Many algorithms^[^ ^69^, ^70^, ^71^, ^72^ ^]^ and software (such as, RDKit,^[^ ^73^ ^]^ wSterimol,^[^ ^65^, ^74^ ^]^ ConfGen,^[^ ^75^ ^]^ CREST^[^ ^66^, ^76^ ^]^) have been developed to perform conformational search. Although the system under study is of medium size (less than 100 atoms), the conformational search is complicated due to its flexibility.^[^ ^77^ ^]^ Moreover, the geometry of the catalyst is determined by halogen and hydrogen bonds, with π–π stacking also being possible. Therefore, we needed a conformational search program that incorporates one of the modern SQM methods parameterized to account for all of the aforementioned interactions. We chose CREST because it includes semiempirical GFNn‐xTB methods, features three algorithms for conformational search (*meta*‐dynamics, MD simulations, and Genetic Z‐matrix crossing), and sorts conformers based on several criteria (energy, root‐mean‐square deviation of atomic Cartesian coordinates, and the difference between rotational constants of two molecules). Additionally, the conformational ensembles generated by CREST have been validated by several experimental spectroscopic studies.^[^ ^78^, ^79^, ^80^, ^81^ ^]^

For the conformational search with CREST, an energy window of 10.0 kcal mol^−1^ and five different geometries were used since the results of the conformational search may depend on the starting geometry. Totally 13,344 structures were found, 1535, 5339, 213, 5346 and 911 for each run, respectively. To decrease the number of calculations, the energy window was decreased to 8.0 kcal mol^−1^, and 1732 structures in this energy window (661, 405, 89, 122, 455, respectively) were optimized using DFT (ORCA, RI‐BP86‐D3BJ/def2‐SVP, CPCM(toluene)). For frequency calculations, the energy window was reduced to 3.0 kcal mol^−1^, and only four unique structures fit into this energy window. These geometries are provided in Supporting Information. To calculate the abundance of those conformers, the Boltzmann energy distribution was used, along with more accurate electronic energy from single‐point calculations at RI‐M06‐2x/def2‐TZVPP (ma‐def2‐TZVPP for I) SMD(toluene).

### 2.3 Different Starting and Reacting Geometries

**Figure 1:**
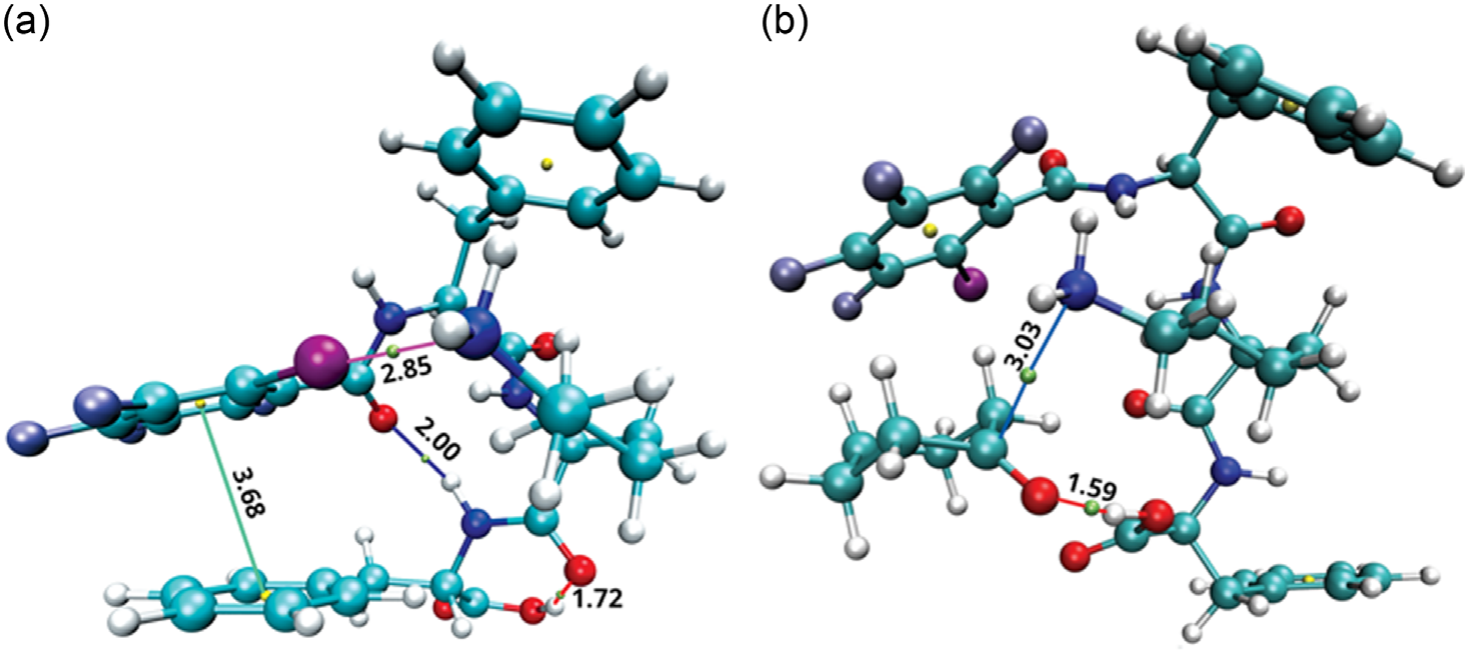
a) The lowest energy conformation of catalyst and b) the reacting system (conformer **O**).

Conformational search revealed that only one dominant conformer of the catalyst is present in the solution with an abundance of 92.9% according to the Boltzmann distribution (Figure S1 and Table S1, Supporting Information). In this conformer, the NH_2_‐group points toward the I atom, and the N‐I distance is 2.85 Å (**Figure** **1**a). Topology analysis showed the presence of a halogen bond (purple). Additionally, π–π stacking between C_6_H_5_ and C_6_F_4_I rings, two hydrogen bonds (OH‐O (red) and NH‐O (blue)), and many weak Van der Waals interactions were observed (Table S2, Supporting Information). However, for enamine formation, the catalyst conformation must change. In the preferred conformation, the amino group attacks the carbonyl of cyclohexanone, as shown in Figure 1b, and the catalyst must undergo some reorganization before the reaction between the NH_2_‐group and cyclohexanone becomes possible.

### 2.4 Simplification of the Studied System

As mentioned previously, the studied catalyst is flexible, with many minima located close to each other, both in terms of energy and geometry. The addition of cyclohexanone in the model causes the formation of a hydrogen bond and stabilization of the entire system by 2.5 kcal mol^−1^, while it also increases the system size and, more importantly, enlarges the number of minima. After several unsuccessful attempts to calculate the catalyst reorganization in the presence of cyclohexanone, it was decided to simplify the model by eliminating cyclohexanone. Since the catalyst reorganization precedes the reaction with cyclohexanone, such a simplification of the system does not carry any risks.

**Figure 2:**
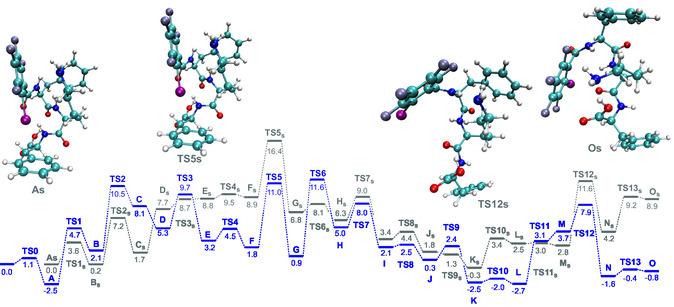
Possible pathway of catalyst reorganization needed for the reaction with cyclohexanone: Gray, simplified modeled system; blue, entire catalytic system.

Catalyst reorganization includes the formation and breaking of H‐bonds, rotation of phenyl and iodotetrafluorophenyl rings, carboxyl and hydroxyl groups, and so on (Figure S2 and Table S3, Supporting Information). We found 13 TSs, and one more was too close to the product and had too smooth a potential energy surface, so we could not localize it. The highest barrier, 16.4 kcal mol^−1^, corresponds to **TS5**_**s**_ (where the last “s” refers to simplified model) and is caused by the rotation of the OH group and the breaking of the H‐bond between the OH and O=C groups (**Figure** **2**). A description of the negative frequency modes in the TSs is presented in Figure S2 and Table S3, Supporting Information.

From the above calculations, it is clear that the catalyst can change its conformation rapidly at room temperature. We do not claim that the pathway found is the only or optimal way for catalyst reorganization for the reaction. Moreover, the final conformer **O**_**s**_, which is needed for further reaction (where the NH_2_‐group and cyclohexanone come close enough to react), has an energy that is 8.9 kcal mol^−1^ higher than the reactant, making the formation of conformer **O**_**s**_ unlikely (Figure 2). However, if we are able to find another conformer where the NH_2_‐group and cyclohexanone are situated close to each other, or if we manage to reduce the relative energy of conformer **O**_**s**_, then we could claim that our catalyst reacts with cyclohexanone.

### 2.5 Conformational Changes of the Catalyst in the Presence of Cyclohexanone: from Reduced to Entire Model

Because of the high energy of conformer **O**_**s**_, the catalyst will not adopt this conformation for long enough to give cyclohexanone time to approach and react. However, the presence of cyclohexanone stabilizes the catalyst through an H‐bond between the carboxyl hydrogen and cyclohexanone's oxygen, as well as other noncovalent and electrostatic interactions (Figure 1, Table S4, Supporting Information). This makes conformer **O** 0.8 kcal mol^−1^ lower in energy than the reagents (Figure 2 and Table S5, Supporting Information), which allows us to conclude that the formation of conformer **O** in solution is possible, considering the accuracy of the DFT approach.^[^ ^14^ ^]^

Since the inclusion of cyclohexanone in the model system can significantly affect the energy, we decided to recalculate the catalyst reorganization with the presence of cyclohexanone. Optimization of the entire catalytic system, including cyclohexanone, was done in two steps. Firstly, the previously found TSs were frozen, and only the position of the added cyclohexanone was optimized. In the second stage, the structures were completely reoptimized. Results are shown in Figure 2 and Table S5, Supporting Information. To escape artificial stabilization caused by unbalanced basis set expansion of the catalyst and cyclohexanone, a geometrical correction for the basis set superposition error (gCP)^[^ ^82^ ^]^ was used as a single‐point calculation with the def2‐TZVPP basis set. In most cases, the inclusion of cyclohexanone caused a change in the relative Gibbs free energy of several kcal mol^−1^, and this could be either an increase or a decrease. Thus, in **TS1**–**TS3**, **TS6**, and **TS9**, the inclusion of cyclohexanone increased the relative Gibbs free energy by 1.1, 3.3, 1,0, 3.5 and 1.1 kcal mol^−1^, respectively. This increase in energy may be caused by nonoptimal substrate positioning or changes in catalyst geometry caused by the presence of the substrate. In contrast, in **TS4**, **TS5**, **TS10**–**TS13**, the presence of cyclohexanone decreased the relative Gibbs free energy by 5.0, 5.4, 5.4, 3.7, and 9.6 kcal mol^−1^, respectively. Herein, gCP changed in a range of −0.8 to +0.7 kcal mol^−1^. Thus, we conclude that stabilization was achieved by H‐bonding and other noncovalent interactions but not due to artificial energy decrease caused by the basis set superposition error (BSSE), and all further calculations will contain the gCP correction.

Remarkably, the greatest energy reduction of 9.6 and 9.7 kcal mol^−1^ was observed for conformer **O** and **TS13**. We assume that the catalyst reorganization can happen with or without cyclohexanone, and that it can also be a combination of the blue and grey pathways. However, after the formation of conformer **K**, the presence of cyclohexanone is preferred, and in the case of **TS13**, it is mandatory since cyclohexanone plays a critical role in the stabilization of the system and the formation of conformer **O**.

### 2.6 Additional Stabilization by Water

**Figure 3:**
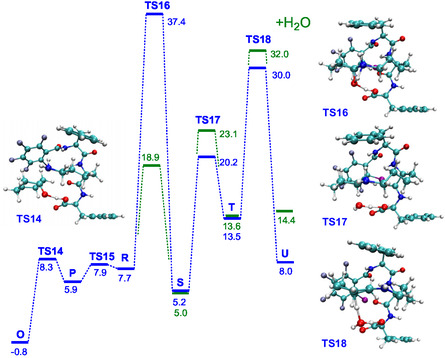
Enamine formation with (green) and without (blue) the presence of water.

Calculations showed that when cyclohexanone approaches the NH_2_‐group of the catalyst, the formation of the C‐N bond occurs with a barrier of 8.3 kcal mol^−1^ (**TS14**, **Figure** **3** and Table S6, Supporting Information). After a small rearrangement (**TS15**), protonation of the hydroxyl group takes place (**TS16**); however, the barrier for this is 37.4 kcal mol^−1^. This is too high for a reaction that occurs at room temperature.

Since there are numerous examples when adding water lowers the reaction barrier, particularly in the case of proton transfer^[^ ^83^, ^84^, ^85^, ^86^ ^]^ in a variety of solvents,^[^ ^87^, ^88^, ^89^, ^90^ ^]^ we decided to include a water molecule in our model. Furthermore, it was reported that water, even at low concentrations, may be essential to the reaction^[^ ^88^, ^89^ ^]^ and promote the synthesis of enamine intermediates.^[^ ^91^, ^92^, ^93^ ^]^ Even though the reaction occurs in toluene, the solution may contain some water held by H‐bonds. In addition, water is formed during the reaction.

The inclusion of a water molecule into the model decreased the barrier for proton transfer to 18.9 kcal mol^−1^ (Figure 3 and Table S7, Supporting Information). However, the presence of water did not contribute to the reduction of the energy of other barriers. Elimination of water (**TS17**) has a barrier of 20.2 kcal mol^−1^ (and 23.1 kcal mol^−1^ in the presence of the water molecule). Subsequent formation of the enamine (**TS18**) required 30.0 kcal mol^−1^ (and 32.0 kcal mol^−1^ in the presence of water). This is again too high for a reaction that occurs at room temperature.

### 2.7 Multiple Reaction Pathways

To lower the energy of **TS18**, other paths for enamine formation with and without the presence of water were tested. Our attempts resulted in a decrease of **TS18** to 27.3 kcal mol^−1^, which is still too high for a reaction at room temperature. However, most of the attempts increased the energy (more details can be found in Table S6, Supporting Information).

**Figure 4:**
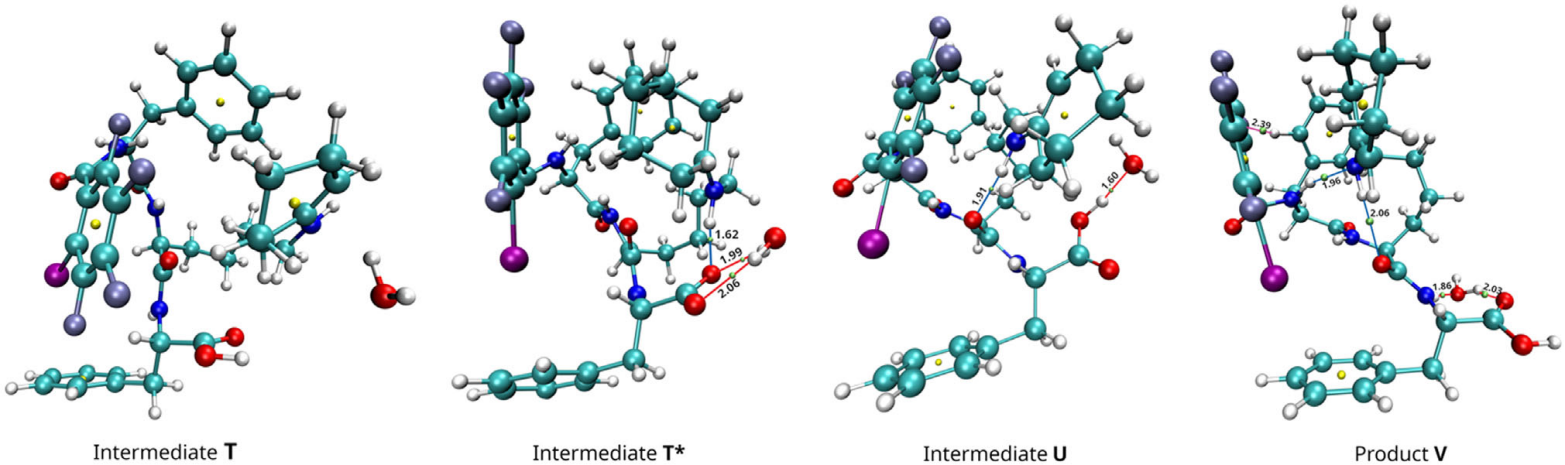
Geometries of intermediates **T**, T*, U*, and **V**.

**Figure 5:**
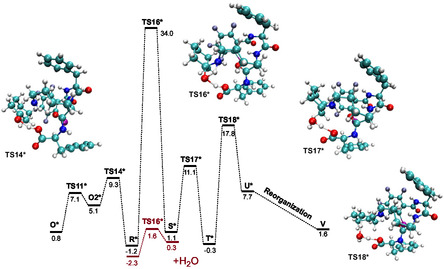
An alternative pathway for enamine formation without (black) and with (red) the presence of water.

Another possible reaction pathway could involve a new rearrangement of the system, so we performed a new conformational search for intermediate **T**. During one run with gbsa‐GFN2‐xTB level of theory 157 conformers were found in an energy window of 6.0 kcal mol^−1^. Optimization at the DFT level of theory showed that the difference between the two lowest energy conformers is 3.8 kcal mol^−1^. In the newly found structure T*, the aminobutyl chain changed its conformation, resulting in the formation of a new H‐bond between the NH and C=O groups of the catalyst, which caused a shift in the C_6_F_4_I ring, rotation of the carboxyl group and one of the C_6_H_5_ rings, and a significant change in the position of cyclohexene (**Figure** **4** and Tables S8 and S9, Supporting Information). Structure T* was 13.2 kcal mol^−1^ lower in energy than the initial one (**Figure** **5**). As a result, the barrier heights for the new TS17* (elimination of water) and TS18* (formation of enamine) also decreased in energy to 10.3 kcal mol^−1^ and 17.0 kcal mol^−1^, respectively (Figure 5, Table S10, Supporting Information). Proton transfer in TS16* still has a high barrier of 32.8 kcal mol^−1^, but the presence of water reduced it to 1.6 kcal mol^−1^ (Table S11, Supporting Information). As in the case of the previously shown catalyst reorganization, the conformational change in the catalyst (TS11*) has a low energy barrier of 7.1 kcal mol^−1^.

The reaction results in the formation of the enamine U* which includes a H‐bond between the NH and C=O groups (distinct from the one in T*), with the substituted cyclohexene parallel to the C_6_F_4_I ring, but is shifted (Figure 4 and Table S12, Supporting Information). However, despite all the lowering energy interactions, U* is 7.7 kcal mol^−1^ higher in energy than the reagents. To understand if it is possible to reduce the energy of the product, we performed one more conformation search. During one run with gbsa‐GFN2‐xTB level of theory, 278 conformers were found in an energy window of 6.0 kcal mol^−1^. Optimization at the DFT level of theory showed that the difference between the two lowest energy conformers is 1.6 kcal mol^−1^. In the lowest‐energy conformer, **V** cyclohexene is also parallel to the C_6_F_4_I ring but is not shifted (Figure 4 and Table S13, Supporting Information). In this conformer, the H‐bond between the NH and C=O remains and a new H‐bond is formed between NH and another NH group. Additionally, the water molecule changed its position so that two new H‐bonds are formed. Conformer **V** is 6.1 kcal mol^−1^ lower than intermedia U* and 1.6 kcal mol^−1^ higher than the reagents. This allows us to conclude that the formation and presence of conformer **V** in solution is possible.

### 2.8 Other Methods

To evaluate the performance of our method, we used two functionals (B3LYP and PBE0), which are common in reaction mechanism studies, to recalculate the five highest energy barriers (three TSs determining catalyst reorganization (**TS2**, **TS5**, and **TS6**) and two TSs related to proton transfer during enamine formation (TS17* and TS18*)). Additionally, the abovementioned TSs were recalculated using M06‐2X functional with D3 dispersion correction added (**Table** **1** and S14, Supporting Information). In most cases, the energies were similar, differing by 0.5–2 kcal mol^−1^. Herein, the M06‐2X functional, combined with the D3 dispersion correction, gave the closest TS energies, differing by −0.9–1.1 kcal mol^−1^ compared to those calculated with the M06‐2X functional without the dispersion correction. This agrees with the conclusions by Liu et al.,^[^ ^41^ ^]^ that the dispersion correction does not significantly change the accuracy of the M06‐2X functional. The functionals B3LYP and PBE0, combined with the D4 dispersion correction, increased the energy of **TS2** by 1.2 and 1.3 kcal mol^−1^, respectively. B3LYP‐D4 also increased the energy of **TS3** by 0.7 kcal mol^−1^, but PBE0‐D4 converged into another TS with lower energy. Because of the shallow potential energy surface, we could not find **TS6**—optimization with B3LYP‐D4 and PBE0‐D4 resulting in a different position for cyclohexanone and significantly different energies. In the case of TSs connected to proton transfer, B3LYP‐D4 and PBE0‐D4 decreased the energy by 1.3 and 2.7 kcal mol^−1^ for **TS17**, and by 4.1 and 7.0 kcal mol^−1^ for **TS18,** respectively.

**Table 1:** Recalculated TSs using RI and SMD‐**X**/def2‐TZVPP//CPCM‐X/def2‐SVP level of theory where **X** is M06‐2X, M06‐2X‐D3, B3LYP‐D4, or PBE0‐D4.

	M06‐2X	M06‐2X‐D3	PBE0‐D4	B3LYP‐D4
**A**	0.0	0.0	0.0	0.0
**TS2**	10.5	11.6	11.7	11.8
**TS5**	11.0	12.1	5.8	11.7
**TS6**	11.6	13.4	N/A	N/A
**TS17**	11.1	10.7	8.4	9.8
**TS18**	17.8	16.9	10.8	13.7

In addition, we performed the single‐point calculations of the above‐mentioned TSs using B2PLYP‐D3BJ and DSD‐PBEP86‐D3BJ double‐hybrid functionals, which outperform hybrid functionals in terms of energy calculation accuracy (**Table** **2** and S15, Supporting Information).^[^ ^94^, ^95^ ^]^ Energies of TSs determining catalyst reorganization increased by 1–2 kcal mol^−1^, up to 13.3 kcal mol^−1^ when triple‐zeta basis set was used. In contrast, increasing the size of the basis set to quadruple‐zeta placed energies lower than doble‐hybrid/def2‐TZVPP and higher than M06‐2X/def2‐TZVPP. Energies of TSs related to proton transfer decreased by 0.5–1 kcal mol^−1^ when a triple‐zeta basis set was used. The use of a quadruple zeta basis set additionally decreased barrier energies up to 9.9 kcal mol^−1^. Thus, the results obtained from single‐point calculations using double‐hybrid functionals differ by 1‐2 kcal mol^−1^ from the M06‐2X results. Our observations agree well with the conclusion made by Portela and Fernández^[^ ^95^ ^]^ that hybrid functionals (PCM‐M06‐2X/def2‐TZVPP//PCM‐B3LYP‐D3/def2‐SVP) reproduce well the reactivity trends.

**Table 2:** Recalculated TSs using RI and SMD‐**X**//CPCM‐M06‐2X/def2‐SVP level of theory where **X** is M06‐2X/def2‐TZVPP, B2LYP‐D3BJ/def2‐TZVPP, B2LYP‐D3BJ/def2‐QZVPP, DSD‐PBEP86‐D3BJ/def2‐TZVPP, and DSD‐PBEP86‐D3BJ/def2‐QZVPP.

	M06‐2X	B2LYP‐D3BJ		DSD‐PBEP86‐D3BJ	
	def2‐TZVPP	def2‐TZVPP	def2‐QZVPP	def2‐TZVPP	def2‐QZVPP
**A**	0.0	0.0	0.0	0.0	0.0
**TS2**	10.5	12.3	11.8	12.7	12.1
**TS5**	11.0	13.1	12.9	13.3	13.1
**TS6**	11.6	12.7	12.1	13.2	12.4
**TS17**	11.1	10.2	9.7	10.6	9.9
**TS18**	17.8	16.0	15.4	17.4	16.5

At the end of this study, we found that the Coulomb radius of iodine was underestimated in the original SMD model, and it is recommended to use the SMD18 model^[^ ^96^ ^]^ to more accurately describe the halogen bonding, especially in the SMD‐M06‐2X+gCP combination.^[^ ^61^ ^]^ We performed SP calculations for the highest energy TSs using SMD18 and CPCM models. The results presented in **Tables** **3** and S16, Supporting Information, show that the relative energy of TSs changed within 1 kcal mol^−1^ and changed more when the CPCM model was used.

**Table 3:** Recalculated TSs using RI and **X**‐M06‐2X/def2‐TZVPP//CPCM‐M06‐2X/def2‐SVP level of theory where **X** is continuum solvation model.

	SMD + gCP	SMD18 + gCP	SMD18	CPCM
**A**	0.0	0.0	0.0	0.0
**TS2**	10.5	10.0	10.1	11.0
**TS5**	11.0	11.4	11.4	12.3
**TS6**	11.6	10.0	10.9	12.3
**TS17**	11.1	10.8	10.1	10.7
**TS18**	17.8	17.5	16.7	17.8

Based on the above, we conclude that the SMD‐M06‐2X/def2‐TZVPP//CPCM‐M06‐2X/def2‐SVP level of theory is accurate enough. However, to determine the exact proton transfer barriers, optimization should be performed with a larger basis set.

## 3 Conclusion

The formation of enamine from cyclohexanone and tripeptide Phe–Lys–Phe was computationally studied and analyzed. Despite the large flexibility of the catalyst, the conformational search revealed the presence of one main conformer with an abundance of 92.9% according to the Boltzmann distribution. However, further study of the catalyst showed that it has low energy barriers of reorganization (in the range of 0.5–16.4 kcal mol^−1^), and the presence of a number of functional groups capable of H‐bond formation and π–π stacking prolongs the lifetime of higher‐energy conformers. Thus, we conclude that the studied catalyst is quite mobile in solvent and adopts many conformations, despite the results of the conformational search. The catalyst's ability to reorganize makes further reaction possible, since in the lowest‐energy conformer the carbonyl group of the cyclohexanone is situated on the opposite side of the NH_2_ group, and they cannot react.

We have also shown how important the choice of the initial model is. The inclusion of cyclohexanone in the model significantly complicated the study, as its presence increased the number of potential energy minima, which were separated by low barriers on a shallow potential energy surface, often leading to the identification of incorrect TS and ground states. Excluding cyclohexanone from the model significantly simplified the catalyst reorganization study and TS search. However, in the simplified model, the conformation we searched for was 8.9 kcal mol^−1^ higher in energy compared to the reactants. The presence of cyclohexanone decreased the energy by up to −0.8 kcal mol^−1^ compared to the reactants. We have also demonstrated how important the environment can be. Firstly, the presence of cyclohexanone significantly lowered the energy of the conformation. In addition, the inclusion of a water molecule in the model system notably decreased the energy of protonation of the hydroxyl group (from 37.4 to 18.9 and from 34.0 to 1.6 kcal mol^−1^). Finally, we demonstrated that the alternative reaction pathway would be preferable to the one originally found and that the flexibility and mobility of the catalyst allow and contribute to the catalyst's conformations varying at different stages of the reaction.

In the present article, we also observed and discussed aspects such as the selection of computational methods and programs, as well as the evaluation of method performance in the absence of experimental data and data obtained using higher‐precision approaches, such as coupled‐cluster calculations.

To sum up, using the example of catalytic enamine formation, we considered both the computational difficulties (e.g., choice of suitable software and proper method) to solve a specific problem, and the limitations of the models used, as well as the ways to overcome those limitations.

## 4 Computational Details

**A conformational search** was performed using the CREST version 2.12,^[^ ^76^, ^97^ ^]^ the GFN2‐xTB^[^ ^36^ ^]^ semiempirical method and an energy window of 10.0 kcal mol^−1^. To account for the effect of toluene, a generalized born solvation model (gbsa) was applied. Subsequently, geometries were optimized at the DFT level using ORCA 5.0.4 software.^[^ ^98^, ^99^ ^]^ First, geometries within an energy window of 8.0 kcal mol^−1^ (for catalyst) and 6.0 kcal mol^−1^ (for conformers **T** and **V**) found by CREST were reoptimized using the resolution of identity (RI‐J)^[^ ^100^ ^]^ approximation for the Coulomb part, along with the BP86^[^ ^101^, ^102^ ^]^‐D3BJ^[^ ^103^ ^]^/def2‐SVP^[^ ^50^ ^]^ level of theory and the CPCM continuum solvent model.^[^ ^104^ ^]^ Next, conformers within the energy window of 3.0 kcal mol^−1^ were reoptimized using the resolution of identity (RI‐JONX), M06‐2X^[^ ^42^ ^]^/def2‐SVP (ma‐def2‐SVP for I) level of theory, and the continuum solvent model CPCM. To confirm that the obtained structures correspond to energy minima, frequency calculations were performed at RI‐M06‐2X/def2‐SVP level of theory (and ma‐def2‐SVP for I). For more accurate electronic energy, single‐point calculations were performed using RI‐M06‐2X/def2‐TZVPP (and ma‐def2‐TZVPP for I) level of theory and the SMD continuum solvent model.^[^ ^105^ ^]^

The geometries of the four lowest‐energy conformers are provided as Cartesian coordinates in Supporting Information.

All calculations for **reaction modelling** were performed using ORCA 5.0.4 software. For the TS search, potential energy relaxation scans and the NEB‐TS algorithm were used, the latter serving to connect the previously identified TSs into a single pathway. Optimization and frequency calculations were carried out using the RI‐M06‐2X/def2‐SVP (for I – ma‐def2‐SVP) level of theory and the CPCM continuum solvent model to include the effects of toluene. The structure was classified as a ground state if it had no imaginary frequency, and as a transition state if it had one imaginary frequency. The exception is TS6, where we were unable to remove the second imaginary frequency (−3.04). The transition states were confirmed via intrinsic reaction coordinate (IRC) calculations. For more accurate electronic energy, single‐point calculations were performed using RI‐M06‐2X/def2‐TZVPP (and ma‐def2‐TZVPP for I) level of theory and the SMD continuum solvent model. For part 2.8, additional single‐point calculations were done using SMD‐M06‐2X‐D3^[^ ^46^ ^]^/def2‐TZVPP (I – ma‐def2‐TZVPP), SMD‐PBE0^[^ ^106^, ^107^, ^108^, ^109^ ^]^‐D4^[^ ^110^ ^]^/def2‐TZVPP (I – ma‐def2‐TZVPP), SMD‐B3LYP^[^ ^111^, ^112^ ^]^‐D4/def2‐TZVPP (I – ma‐def2‐TZVPP), SMD‐B2PLYP^[^ ^113^ ^]^‐D3BJ/def2‐TZVPP,^[^ ^50^ ^]^ (I – ma‐def2‐TZVPP), SMD‐B2PLYP‐D3BJ/def2‐QZVPP SMD‐DSD‐PBEP86^[^ ^114^, ^115^ ^]^‐D3BJ/def2‐TZVPP (I – ma‐def2‐ TZVPP), SMD‐DSD‐PBEP86‐D3BJ/def2‐QZVPP, SMD18^[^ ^96^ ^]^ and CPCM solvent models. To avoid artificial stabilization caused by unbalanced basis set expansion, a geometric correction for inter‐ and intra‐molecular BSSE (gCP)^[^ ^82^ ^]^ was applied as a single‐point calculation with the def2‐TZVPP basis set. The final optimized geometries are provided as Cartesian coordinates in SI.

Files with TS vibrations corresponding to imaginary modes as well as IRC paths for catalyst reorganization (simplified system) – https://data.taltech.ee/records/bswb6‐vyh93, catalyst reorganization (entire system) – https://data.taltech.ee/records/fhfm8‐90f91, reaction pathway 1 – https://data.taltech.ee/records/39wh1‐fwh67, reaction pathway 2.

**Topology analysis** ^[^ ^116^ ^]^ was performed with MultiWFN software version 3.8.^[^ ^117^ ^]^

**Visualization** was done with VMD^[^ ^118^ ^]^ and Jmol^[^ ^119^ ^]^ programs.

## Conflict of Interest

The authors declare no conflict of interest.

## Supporting information

Supplementary Material

## Data Availability

The data that support the findings of this study are available in the supplementary material of this article.
