# Supplementary material for: Challenges in Mechanistic Investigation of a Flexible Aminocatalyst as Demonstrated through Enamine Formation
Source: ChemistryOpen. 2025 Jun 25;14(9):e202500116. doi: 10.1002/open.202500116 (PMC12409831; doi:10.1002/open.202500116)
Supplement: Supplementary file 1 — Supplementary Material [file OPEN-14-e202500116-s001.zip › Geometries.pdf]

Supporting Information

Problems in Reaction Mechanism Search for Flexible Aminocatalyst on Example of Enamine Formation

Irina Osadchuk,* Tõnis Kanger*

Cartesian coordinates

Catalyst *4*

Catalyst conformation 1 4

Catalyst conformation 2 5

Catalyst conformation 3 6

Catalyst conformation 4 7

Reduced system *8*

Reduced Intermedia A_S 8

Reduced $TS1_S$ 9

Reduced Intermedia B_S 10

Reduced $TS2_S$ 11

Reduced Intermedia C_S 12

Reduced Intermedia D_S 13

Reduced $TS3_S$ 14

Reduced Intermedia E_S 15

Reduced $TS4_S$ 16

Reduced Intermedia F_S 17

Reduced $TS5_S$ 18

Reduced Intermedia G_S 19

Reduced $TS6_S$ 20

Reduced Intermedia H_S 21

Reduced $TS7_S$ 22

Reduced Intermedia I_S 23

Reduced $TS8_S$ 24

Reduced Intermedia J_S 25

Reduced $TS9_S$ 27

Reduced Intermedia K_S 28

Reduced $TS10_S$ 29

Reduced Intermedia L_S 30

Reduced $TS11_S$ 31

Reduced Intermedia M_S 32

Reduced $TS12_S$ 33

Reduced Intermedia N_s 34
Reduced $TS13_s$ 35
Reduced Intermedia O_s 36
Entire system 37
Entire reagents 37
Entire TS0 38
Entire Intermedia A 40
Entire TS1 41
Entire Intermedia B 42
Entire TS2 43
Entire Intermedia C 45
Entire Intermedia D 46
Entire TS3 47
Entire Intermedia E 48
Entire TS4 50
Entire Intermedia F 51
Entire TS5 52
Entire Intermedia G 53
Entire TS6 55
Entire Intermedia H 56
Entire TS7 57
Entire Intermedia I 58
Entire TS8 60
Entire Intermedia J 61
Entire TS9 62
Entire Intermedia K 64
Entire TS10 65
Entire Intermedia L 66
Entire TS11 67
Entire Intermedia M 69
Entire TS12 70
Entire Intermedia N 71
Entire TS13 72
Entire Intermedia O 74
Pathway 1 75
TS14 75
Intermedia P 76
TS15 77

Intermedia R 79
TS16 80
Intermedia S 81
TS17 83
Intermedia T 84
TS18 85
Intermedia U 86
TS18-1 88
TS18-2 89
TS18-3 90
TS18-4 91
TS18-5 93
TS18-6 94
Intermedia R + H2O 95
TS16 + H2O 96
Intermedia S + H2O 98
TS17 + H2O 99
Intermedia T + H2O 100
TS18 + H2O 102
Intermedia U + H2O 103
Pathway 2 104
Intermedia O* 104
TS11-1* 106
Intermedia O1* 107
TS11-2* 108
Intermedia O2* 109
TS14* 111
Intermedia R* 112
TS16* 113
Intermedia S* 114
TS17* 116
Intermedia T* 117
TS18* 118
Intermedia U* 119
Intermedia R* + H2O 121
TS16* + H2O 122
Intermedia S* + H2O 123
Product V 125

Catalyst

76

Catalyst conformation 1

C	-4.07316420224514	1.08664308965635	-1.71895200586937
C	-4.08077401635119	1.96543320918784	-0.64610629830893
C	-2.90060644082854	2.21406190939943	0.04888976941678
C	-1.70361898626112	1.60294948242547	-0.30673885318715
C	-1.69565728737007	0.70089176596285	-1.38527144775551
C	-2.88272335584911	0.46306350339463	-2.07991243879117
F	-5.18626024100227	0.83961934503347	-2.38624414761247
F	-5.21210999196843	2.52909167487951	-0.26137637517935
F	-2.97297040738521	3.03690898834670	1.08774699089269
I	-0.02270741326617	2.01653721554214	0.91985375878824
F	-2.91324641964678	-0.35411600384133	-3.12164813376619
C	-0.50194556631223	-0.10356674808522	-1.84381118891901
O	-0.58264623123671	-1.31426455300018	-1.98117677694428
N	0.62861916889031	0.58046306855400	-2.12458712991442
C	1.72902007192584	-0.05340529336020	-2.82767119175924
C	2.76879950099385	0.98786877427938	-3.27052626143910
C	3.49106043511796	1.65528213579229	-2.12360406701848
C	3.03631920869532	2.86452058220769	-1.58451599511986
C	3.70207522159192	3.46676367318031	-0.51455080991933
C	4.84040288276055	2.86839195046442	0.02636337668404
C	5.30387895322384	1.66135410454067	-0.50277336059463
C	4.63160078285077	1.05869566744750	-1.56488370313950
C	2.42727639834305	-1.18266376164415	-2.06082201024195
O	3.14986706181178	-1.94541200303736	-2.67157909803804
N	2.21205662879493	-1.25110929025155	-0.72480783345903
H	0.63025213558331	1.59069750297520	-2.01786414148893
H	1.33354869721197	-0.55545790550569	-3.72478197360277
H	3.48835315734245	0.45700723852540	-3.90803905680086
H	2.26145332067944	1.73874427356238	-3.89436472234217
H	2.16257264152434	3.35853790650859	-2.01985566851104
H	3.33547180688546	4.41464540026443	-0.11469246258984
H	5.37213027950447	3.34467299273165	0.85253869781671
H	6.19641608056135	1.18884464231853	-0.08825297075921
H	4.99623833368649	0.11546414578952	-1.98076484744689
H	1.65923064168374	-0.52072054158630	-0.28532986305933
C	2.73320012334348	-2.34888715211128	0.06335937944625
C	3.91096483137110	-2.00987478088425	0.98739386547882
C	1.62924202537833	-3.12299163539740	0.77819637409258
O	1.90516530737573	-3.87767021548931	1.71236599761941
C	-0.73798042109380	-3.77294302806067	0.83287859740168
H	4.36798645262388	-2.96187520712581	1.29072318159736
H	3.11917440462673	-3.06363420803077	-0.68240183686543
H	-0.61569846865190	-3.80281248979988	1.92895582609279
H	4.64928458960393	-1.47962508823456	0.36480008523945
N	0.38211425790673	-3.00920454581539	0.29169148390032
H	0.21346487671998	-2.46480860017149	-0.55638550386678
C	-2.06338188189856	-3.13308273645904	0.45648200201555
H	-2.05180581231670	-2.89494951124380	-0.62008447107767
C	-2.44963421248211	-1.90006964507073	1.24528498548241
C	-3.70340768559380	-1.31746526551752	0.99507843372660
C	-1.64796611599773	-1.33818213742104	2.24403690353568
C	-4.14858217385664	-0.22404039584588	1.73254224525062
H	-4.34139272076707	-1.74458350546926	0.21644689117240
C	-2.09592325626398	-0.24240545930732	2.98939338584680
H	-0.66244300040893	-1.75551161471845	2.46212783354969
C	-3.34565746436395	0.31767799156109	2.74103715230147
H	-5.12785952471505	0.21173296600941	1.52090162876166
H	-1.45476141339625	0.17512702473974	3.76826082466118
H	-3.69234956757200	1.17430806997543	3.32120545964907
H	-2.84538485523291	-3.89439815337742	0.58435288015633
C	-0.66536190418822	-5.24085916118070	0.36815548151201
O	-1.53358807312076	-5.78255014865218	-0.25554922349666
O	0.44162691217636	-5.87598319214403	0.73187611263695
H	1.02698897566264	-5.28550107581440	1.26358357523892
C	3.59987464560465	-1.18635342370807	2.23682852300774
H	4.55642045220845	-0.95336680412797	2.73340500492369
H	3.02147083075694	-1.79917712250442	2.94675980136907
C	2.85006613846190	0.10656096377944	1.93916724552370
H	3.29813356354662	0.60371806341859	1.05921524338127
H	1.80833274773004	-0.13140930170489	1.67235166378103

C	2.81460185360002	1.10687252162391	3.08586155202817
H	2.19084345508848	0.71559844302291	3.90516389931078
H	3.83483061298279	1.23495154025747	3.49471251558295
N	2.23767723890795	2.37182869609085	2.62539233318474
H	2.07806718023009	3.00678729440530	3.40675105265378
H	2.89836025057340	2.84023900214547	2.00318704237322

76

Catalyst conformation 2

C	-4.03037558079824	1.13366741644883	-1.98265972923202
C	-4.04913257519538	1.97622123815819	-0.87829676337616
C	-2.89861145106989	2.12831819756410	-0.10980990389510
C	-1.72003414047747	1.45774414225517	-0.41880869628008
C	-1.69869183729662	0.61010291015887	-1.53738172145105
C	-2.85569719722320	0.45955571482484	-2.30369256379326
F	-5.11260501471057	0.98792147089397	-2.72673055330932
F	-5.15682999076856	2.61821935399097	-0.55353581036318
F	-2.98544611551619	2.91503759311464	0.95685530387884
I	-0.15197058733574	1.63636830733846	1.00705794302935
F	-2.86492286172048	-0.31267135823272	-3.37946724324902
C	-0.50086462773863	-0.20135932929084	-1.96532370754231
O	-0.58235544045582	-1.40631448245706	-2.14495673995554
N	0.64510142630843	0.48497884909193	-2.16220096767361
C	1.79459861397080	-0.15829182621647	-2.76675290626404
C	2.79428703520574	0.87571135113433	-3.30206064833715
C	3.49031066283398	1.67121830724398	-2.22360178110181
C	2.94953881651415	2.86530340415846	-1.72976488147189
C	3.60400955367282	3.58653191625976	-0.72874412524659
C	4.82059173906998	3.13077704521079	-0.22186650867551
C	5.36930898258341	1.94215650949800	-0.70666065202040
C	4.70393006036009	1.21311197309499	-1.69041073907974
C	2.50994239665620	-1.17584598048455	-1.86696202170355
O	3.32125676027985	-1.93271915977769	-2.36399513639334
N	2.18008393541265	-1.17453049061309	-0.55299168897666
H	0.65742071985694	1.48786390769743	-2.00170081343949
H	1.43783347974153	-0.76854565646263	-3.61115688939827
H	3.53705074717594	0.31694828026284	-3.88638207698035
H	2.25973443446894	1.54454381453852	-3.99281585726224
H	2.01966065831299	3.25830609232079	-2.15190718129865
H	3.16902887039117	4.51720576684424	-0.35953636121681
H	5.34295445609757	3.70303296350561	0.54699911626458
H	6.32214728671477	1.58018353349104	-0.31586430541297
H	5.13126542349402	0.28017690286409	-2.06662021873945
H	1.53703680146635	-0.46587741054386	-0.20736161772209
C	2.61875713772477	-2.22206204453136	0.34748646266076
C	3.59576252007113	-1.77803720367397	1.43332643748849
C	1.42124257606107	-2.96698670264831	0.96306206044709
O	1.52189716014056	-3.55622130483452	2.02458673088876
C	-0.87798526964660	-3.71980720937289	0.55912783801050
H	3.83213107596028	-2.67743645493549	2.01599280524364
H	3.12636594404586	-2.96206481784680	-0.29189974526077
H	-0.69441897170796	-4.16213671134146	1.55207396215478
H	4.52633127314467	-1.44656811517958	0.94598244264584
N	0.28382289194282	-2.93500878675121	0.23012637414929
H	0.28198600115397	-2.47385588659334	-0.67728423473793
C	-2.15515519589481	-2.88529381952274	0.57084842577063
H	-2.27093270015403	-2.41496286576039	-0.42028556090315
C	-2.22072193012068	-1.82080871765009	1.64334125624801
C	-1.36992767904330	-1.79573359214939	2.75620365533561
C	-3.21162323602894	-0.83321255366312	1.54353855044241
C	-1.52921222113480	-0.82686139459399	3.75118253883818
H	-0.56874487484492	-2.53122984784506	2.86153304421345
C	-3.37133942429755	0.13449252118682	2.53315553722297
H	-3.87186321030237	-0.83134638302227	0.67062072396704
C	-2.53010045607607	0.13765243886680	3.64778427868780
H	-0.86108901181798	-0.83199641253815	4.61515510056256
H	-4.15009947211001	0.89325077060533	2.43039639314897
H	-2.65216442871883	0.89282623184843	4.42625643518401
H	-3.00427720838643	-3.57475469202028	0.68891934035553
C	-1.04285673264612	-4.92037320562345	-0.37069710437114
O	-2.08628439371103	-5.48244239820366	-0.56360217838071
O	0.10734910405509	-5.31099121171916	-0.91880168005530
H	-0.07729458435611	-6.09639357440447	-1.46057771818927
C	3.06805155348452	-0.67789104717379	2.36351336418612
H	3.47610841819055	-0.83405282412133	3.37602945439983
H	1.97139423689155	-0.78035353686413	2.46349922358840

C	3.43561356885944	0.73425724870671	1.91053386115442
H	4.52617894218635	0.78668322308770	1.76553141491131
H	2.98567575839956	0.95564207498363	0.92651638917784
C	3.03601205938899	1.81759078675724	2.90233319363644
H	3.62198430234056	1.69391013157794	3.83303455089523
H	3.29778210955026	2.80242581783479	2.48642344730492
N	1.59601507733660	1.80545280634851	3.15685306204969
H	1.33457122856784	0.98333401112224	3.70310144458965
H	1.32108174852056	2.61206847127235	3.71676527752694

76

Catalyst conformation 3

C	-4.07058502056052	0.93393139755307	-2.03550037489953
C	-4.13374419843455	1.83019701561832	-0.97666280147869
C	-2.98952565036221	2.08835084595978	-0.22740636931492
C	-1.77760393113547	1.46765958095807	-0.51005748880909
C	-1.71055427517737	0.55873668713449	-1.57956988432968
C	-2.86179164154253	0.31043794885426	-2.33067659552041
F	-5.14445837641376	0.69181797868312	-2.76667125175079
F	-5.27454235559651	2.42706926565585	-0.68167097812859
F	-3.10976969198923	2.93713447464410	0.78679301383631
I	-0.18541994699936	1.85387497152760	0.83556863998041
F	-2.83821660892474	-0.50879076101555	-3.37067608825074
C	-0.48146594385926	-0.22704933509382	-1.97859813636227
O	-0.53564981536546	-1.43570501047532	-2.13249679665944
N	0.64948297564156	0.48354251862463	-2.18845818842250
C	1.80880845278630	-0.11803141405137	-2.82201654110084
C	2.83417008358187	0.95777379862849	-3.21444544138936
C	3.48223289818189	1.64284131492896	-2.03356807381242
C	2.96536092761351	2.83220632022154	-1.50651579305596
C	3.56450886516983	3.44717577371907	-0.40456088515775
C	4.69650438757763	2.88125618090847	0.18238877963630
C	5.22191742082048	1.69463302736536	-0.33480130412109
C	4.61686468838007	1.07995903220173	-1.42976205264873
C	2.50413834336846	-1.21455924210924	-2.00350275123765
O	3.28037180688190	-1.96426272888396	-2.56390022374282
N	2.22441878815950	-1.26192347220058	-0.68186375955010
H	0.62334720470745	1.49157169075966	-2.06725688831537
H	1.48172150571495	-0.64049099758300	-3.73473460065627
H	3.59988245210045	0.45235417730184	-3.81795256333550
H	2.33319008700416	1.69460031520637	-3.85993668303357
H	2.09556650096694	3.30138139388691	-1.97595916305678
H	3.15026810075189	4.38000723702619	-0.01570465053777
H	5.17433156543032	3.36672468955955	1.03568822303969
H	6.10934678911916	1.24648775179480	0.11620813581899
H	5.02843194706492	0.15146580443901	-1.83443466967910
H	1.61515330910828	-0.54852154286864	-0.29095770217314
C	2.70915577283523	-2.33727407131368	0.16440987507935
C	3.89552811982356	-1.97654532820838	1.06747284032816
C	1.55461228241870	-3.00286083447563	0.92148741876064
O	1.72996437039306	-3.57155478862254	1.98423638336324
C	-0.78619928909994	-3.69408069888498	0.72585564558631
H	4.35002523493120	-2.91870744620067	1.40529551781356
H	3.06783346994042	-3.10524387718977	-0.54059943014568
H	-0.57735922163892	-4.07921410985830	1.73569938987569
H	4.63435181002699	-1.48031930748815	0.41778667043505
N	0.35225854350649	-2.92847255024740	0.29895075788186
H	0.30614150894280	-2.54207934163154	-0.64277430491574
C	-2.06308380762146	-2.85074928066989	0.73326386379766
H	-2.21089107254489	-2.43777897892186	-0.27920654423986
C	-2.07938544183752	-1.72414997018887	1.74405465251340
C	-1.16034698259384	-1.62216434162706	2.79612398488685
C	-3.09909994487658	-0.76544511969481	1.65606502564028
C	-1.28396026427150	-0.60829743626660	3.75041669037876
H	-0.33660975778441	-2.33458945366960	2.88836055286177
C	-3.22124600575433	0.24861997280872	2.60363524052286
H	-3.81454746241341	-0.82620463215702	0.83014582261514
C	-2.31390518614599	0.32621703872966	3.66221544824471
H	-0.56499061993433	-0.55401400475882	4.57023384031547
H	-4.02417931809485	0.98313277365545	2.51299800670354
H	-2.40672700485310	1.11664789286324	4.40895140010721
H	-2.91684606947134	-3.51565045602993	0.93191153429450
C	-0.94485869745601	-4.92145554867869	-0.16625635744885
O	-0.22180034823378	-5.20720140643417	-1.07831218649475
O	-1.99597693315792	-5.66326870854469	0.19515114616454
H	-2.03769264651590	-6.42886768235042	-0.40192857742192

C	3.60287372296306	-1.10186457352893	2.28841816795958
H	4.56741869184273	-0.77457528700112	2.71164695589503
H	3.10679985734363	-1.70919291366762	3.06058783464002
C	2.74266148435710	0.11580390007223	1.97699812190322
H	3.12233558674687	0.62749601645628	1.07380841435005
H	1.71580679677526	-0.21506729197496	1.74716673047598
C	2.65879774891304	1.13418617939967	3.10391971872248
H	2.10636315023389	0.70560892603433	3.95435795216333
H	3.67773975509234	1.36390580801703	3.46981206004378
N	1.95286917645304	2.33246838415616	2.64543503723988
H	1.71971120957579	2.94369662624385	3.42706042884895
H	2.56356788501422	2.87173482296972	2.02969132317313

76

Catalyst conformation 4

C	-4.97325037595696	-1.26347785049394	-1.02529090331852
C	-5.36309565350865	-0.13140812356947	-0.32029474630711
C	-4.40127267162125	0.77224022213119	0.12847253188263
C	-3.05071998523419	0.55964485351670	-0.12727834030664
C	-2.65387870877775	-0.57554931302595	-0.84219958836331
C	-3.62063642571831	-1.47327059057327	-1.27764629722736
F	-5.87805480340650	-2.12919483974783	-1.44443950254290
F	-6.64227444489847	0.08159422405654	-0.06915853859302
F	-4.81731409561963	1.82951916764174	0.80913521183664
I	-1.66537439292485	1.93912648659608	0.63141752150340
F	-3.26281365445280	-2.56821275510258	-1.93885693542575
C	-1.22373063375064	-0.85990226618155	-1.27299217064821
O	-0.86925696485478	-0.57316025362580	-2.39931445113134
N	-0.42763905138707	-1.48183281572618	-0.37731065469461
C	-0.69714653332230	-1.71805622944661	1.02383724270769
C	-0.95956419619954	-3.20002421793605	1.34948083206337
C	0.23590935759071	-4.08669654875228	1.10979314919801
C	0.50991277048848	-4.59470368071045	-0.16723813778610
C	1.61873561105288	-5.41447739692836	-0.38418867360498
C	2.46981154002507	-5.73626091424515	0.67358479963028
C	2.21232554520918	-5.22706684436732	1.94710556984157
C	1.10644275105092	-4.40424944356786	2.16176951838106
C	0.39947242675222	-1.16712788371494	1.95090419197833
O	0.23644804022918	-1.19277665877293	3.15575794059728
N	1.51015097027695	-0.68018276978204	1.34435210406042
H	0.51066646579601	-1.67693302055251	-0.75382006925366
H	-1.60046901452467	-1.15985121492452	1.30678284378906
H	-1.26176036082250	-3.25502193865631	2.40384690157334
H	-1.80794028738515	-3.52641556845568	0.72941842593944
H	-0.16701426267935	-4.36009665957703	-0.99298240489912
H	1.81119620454509	-5.81354992940004	-1.38201251957563
H	3.33159062086896	-6.38469480242541	0.50606494695337
H	2.87336587085653	-5.47471818681223	2.77969387924983
H	0.90372112986408	-4.00539723930791	3.15867658719916
H	1.63380464223675	-0.80181824772296	0.33746548890078
C	2.63833143900004	-0.14412420844692	2.07491904795986
C	3.87794072966461	-1.04663277528032	2.04891905089083
C	3.01898914980307	1.25578796176659	1.56276563853982
O	4.09223315359645	1.75870098594331	1.83721329227391
C	2.27290132893569	3.20001154609294	0.27530983734181
H	3.64269522373237	-1.98437306439412	2.57668882211760
H	2.30533997992141	-0.01515491044306	3.11653269692705
H	3.30001038344922	3.50663499707594	0.51748095873756
H	4.65269976558988	-0.52584003486544	2.62649557554450
N	2.08900295748134	1.87056333214490	0.78644385653762
H	1.20350738721821	1.40063518249498	0.62503234191619
C	1.33567136719332	4.17976461928432	0.96305240499111
O	0.34567973713950	3.87444299538824	1.57794667869404
O	1.70886278247572	5.43944397830636	0.76968433641820
H	1.05326455574868	6.02273913185479	1.18958732606468
C	2.03361573454739	3.28287144071650	-1.24208573022107
H	0.96626123251496	3.09800162835810	-1.44567016726471
H	2.23932414968946	4.31658408547756	-1.56205894372767
C	2.86812901007525	2.32080001898201	-2.05578768042057
C	4.14489482250560	1.91184550002818	-1.64590339787052
C	2.36470720685178	1.83052590390096	-3.26793614602127
C	4.90637523048525	1.05556228922386	-2.44430410787540
H	4.55156823935287	2.24381201206924	-0.68716622237310
C	3.12510289150273	0.97577858138912	-4.06603135753809
H	1.35983219540971	2.11793603418264	-3.58627218367406
C	4.40239927189244	0.58898115764636	-3.65888694754632

H	5.89731133519101	0.74572538562446	-2.10680268729634
H	2.71296940524142	0.60530542907513	-5.00639424757891
H	4.99828205287020	-0.08249245541299	-4.27945384680067
C	4.38810716732826	-1.34535102916160	0.63237017263484
H	4.28089270334929	-0.42999677908377	0.02177536306311
H	5.47224928357207	-1.52530376163903	0.67489746148111
C	3.70518473969486	-2.54027778092542	-0.06528110394353
H	4.37063554553912	-3.41687171872141	-0.03962033817146
H	2.79747362613486	-2.83677261191947	0.48498764653046
C	3.30264345090604	-2.26931393676358	-1.51011048733194
H	4.19611359827546	-2.02616655498985	-2.11646095104868
H	2.86305649005421	-3.18414664111899	-1.93516981659881
N	2.29123740637875	-1.21019132512581	-1.57408675840596
H	2.73406506736459	-0.29250534592705	-1.47874495837545
H	1.83091307832522	-1.18463724504464	-2.48458889458716

Reduced system

76

Reduced Intermedia A_s

C	-0.44773215123784	1.09669713595627	-5.51863754956066
C	-1.56681810356537	1.89579884934330	-5.35519488403123
C	-2.28736196092998	1.83334914823935	-4.16538586015415
C	-1.91536106104977	0.97692969153748	-3.13748618125318
C	-0.76651447490438	0.17503518058583	-3.28290920547226
C	-0.06178709981567	0.24845631807869	-4.48521393222921
F	0.25246939456860	1.14430981204274	-6.63683233813321
F	-1.92790185333184	2.73538470668534	-6.30613464845227
F	-3.32022648340640	2.65366391082106	-4.04537116055491
I	-3.11895778772016	1.04311961519373	-1.41747761625994
F	1.02883074023969	-0.48751315206762	-4.69562959566633
C	-0.26847057741828	-0.71231348188150	-2.16380104719681
O	-1.01708643098411	-1.31930420782444	-1.41919789991706
N	1.07594475407303	-0.73478724256021	-1.99913286461111
C	1.67298852909385	-1.52139401781183	-0.94143012911347
C	3.20939799653911	-1.50961187281038	-1.06249341185228
C	3.77877816785726	-0.11278495394536	-0.98509571642579
C	4.00661228774875	0.63843743479957	-2.14503162500226
C	4.43824242660788	1.96434292360663	-2.06565848573374
C	4.65744917521919	2.55303080896739	-0.82079731031311
C	4.45360146806347	1.80720834082028	0.34290741885956
C	4.01730635796805	0.48605233708907	0.25989791297740
C	1.24806106952962	-1.01954240029902	0.44055847612951
O	1.26166326564343	-1.76192301031734	1.41269051278285
N	0.88835135060138	0.27517341823483	0.50960856584876
H	1.67672150941354	-0.20963657064166	-2.62487135418019
H	1.30853385771791	-2.55789435836910	-0.99453257865018
H	3.60398666005972	-2.13595874629596	-0.25055910090579
H	3.47867265112992	-1.98631459742436	-2.01625368612623
H	3.85625335889769	0.17727483707136	-3.12622015394782
H	4.61192631080215	2.53353991439958	-2.98038742222842
H	4.99966556574994	3.58707739887668	-0.75589661813704
H	4.63810458901034	2.25673503057949	1.32024897291987
H	3.85384828109781	-0.09411527125215	1.17279610133527
H	1.02934881778854	0.84926137360182	-0.31225558240205
C	0.61417759355024	0.92147735397087	1.77140200394314
C	0.63327603713957	2.44753435029197	1.65685152443406
C	-0.67059423348855	0.42545993267573	2.44324595891645
O	-0.90656621900870	0.77252299935332	3.58701290741033
C	-2.56347813081686	-1.11916186165570	2.37618204701152
H	0.37350556971487	2.82125363761820	2.65691184562528
H	1.39345493986441	0.62115269835505	2.49328065445635
H	-2.65840270235858	-0.69022127736637	3.38627791764673
H	1.67075496734237	2.76221002416550	1.45678342395160
N	-1.46700464376437	-0.42067897933301	1.74116383092939
H	-1.27391414246435	-0.60500131060939	0.75597096919658
C	-3.89093057062544	-0.98582831452388	1.63769105014382
H	-3.77289756902496	-1.38645347486123	0.61554784760651
C	-4.46987175237778	0.40895971602070	1.56326920125647
C	-5.60695028195983	0.61445209258931	0.76429566600786
C	-3.93659967872954	1.50562401001386	2.25302856097304
C	-6.19006040679675	1.87507797147146	0.64941208582602
H	-6.03499565119906	-0.23278208787881	0.22165159091961
C	-4.51966721141314	2.77078676892234	2.13578785291599

H	-3.06042517894514	1.38783117627460	2.89375497039060
C	-5.64302524105346	2.96268063070528	1.33359932919613
H	-7.07164742875928	2.00872471268329	0.02012667445735
H	-4.08718809896256	3.61187001376565	2.68066017868444
H	-6.09311214993827	3.95239737724670	1.24280409512861
H	-4.60545093442436	-1.65227221889947	2.14156483468999
C	-2.25286120943806	-2.60860416053723	2.61765441539289
O	-3.12572446202279	-3.38368681746754	2.90249750287426
O	-0.98918623093226	-2.99670935485712	2.55000881814045
H	-0.36122134574409	-2.30810838651865	2.25590734348815
C	-0.30120783387063	3.07621720953791	0.61538369879686
H	-0.38168438899847	4.14751515158676	0.86119255962121
H	-1.32173975664634	2.66553916717320	0.71791200501946
C	0.16097853420347	2.96306518498545	-0.84171647507803
H	1.25971520338401	3.09305528677961	-0.89310466554644
H	-0.06360970046833	1.97000020097246	-1.26265415824396
C	-0.49528511870644	3.97055187813598	-1.77752386423354
H	-1.58742732004836	3.81638047848598	-1.75699546516173
H	-0.31562381654469	4.99241055660006	-1.38872773790976
N	-0.02647549954152	3.76995990007410	-3.14449280203478
H	-0.53070418103875	4.38620536014726	-3.78086843468875
H	0.95430060600654	4.04212609530639	-3.21289728903422

76

Reduced TS1$_S$

C	-2.32225764575144	-0.78041592969416	-5.32383431640101
C	-3.03275200340765	0.41129081795849	-5.22913983071899
C	-2.89582057891866	1.21894828977006	-4.09959104441131
C	-2.05125132980997	0.84205823046025	-3.06196818013800
C	-1.34676155905395	-0.36299095786805	-3.14605407806083
C	-1.48625729521382	-1.15962198777469	-4.27573372980333
F	-2.44641675558388	-1.54198670622149	-6.39364679891435
F	-3.83051186714713	0.77936744321757	-6.21239726660052
F	-3.58659110947241	2.34563559272220	-4.05350645455214
I	-1.82597725699016	2.11794295890286	-1.42804580457883
F	-0.82599376810774	-2.30596074997234	-4.36869601597318
C	-0.53550913557743	-0.89354201822845	-1.98819860586763
O	-1.07739879472112	-1.50501756933946	-1.08214071619744
N	0.78884127487690	-0.65420002736533	-2.01543733235160
C	1.65233012468231	-1.31165407340870	-1.04876211391431
C	3.13504954274104	-1.01826871756016	-1.33263329173627
C	3.53704206470449	0.43301765190800	-1.20822153238101
C	3.45516092553418	1.30500692118925	-2.30085015233374
C	3.82463060124176	2.64638746787090	-2.17762821492901
C	4.29985021349591	3.12810142966548	-0.95925749435154
C	4.38928694729395	2.26854797063080	0.13781030842067
C	4.00364154827169	0.93525775880893	0.01485318349891
C	1.27321821076456	-0.97666174571409	0.39692291344444
O	1.32363793194872	-1.84384990554855	1.25697797854105
N	0.91174344696725	0.29663608779652	0.66340158307184
H	1.19049367984879	-0.16215450505488	-2.80712896850216
H	1.49436968405083	-2.39861048531395	-1.11362668733007
H	3.71653516992013	-1.63022017995507	-0.62903648065718
H	3.35696551120072	-1.38820427931435	-2.34533344232150
H	3.12906352607669	0.92865177308498	-3.27485486483672
H	3.74981215745630	3.31047620289771	-3.04010062831756
H	4.60356630241682	4.17220548198199	-0.86272880918485
H	4.76282353752910	2.63866481928781	1.09445122768592
H	4.07541985505191	0.26487700231795	0.87593995954611
H	0.87772241123559	0.97147423888463	-0.09439339563363
C	0.60762286674909	0.69974949766995	2.02354680596712
C	0.95629019912552	2.14400415836802	2.37147515231316
C	-0.82085432183455	0.33480587683845	2.46138069282089
O	-1.25137012729913	0.72217702576295	3.53242928952937
C	-2.68085952404356	-1.21746123431653	2.06252680442083
H	0.69749335157490	2.24387969335574	3.43355232454372
H	1.21886622065798	0.04929419468400	2.67125094163881
H	-3.01653951209143	-0.76463931987816	3.00778752433174
H	2.04756367398152	2.26935877462343	2.29037007232732
N	-1.48906219414758	-0.51998983427516	1.64939876354649
H	-1.15309349410859	-0.70894826247708	0.70679434764649
C	-3.79266871214916	-1.14849562154959	1.01701910717744
H	-3.43447409841123	-1.62920327826075	0.09230388527360
C	-4.28431810026257	0.24328956130565	0.69137351283375
C	-5.01063631840113	0.44004703722629	-0.49204791683403
C	-4.05912952767289	1.34519724030366	1.52490679440216

C	-5.51453090484090	1.69510818217950	-0.82874924873958
H	-5.18156540082580	-0.41006879402446	-1.15803416664821
C	-4.55977486170577	2.60587830398047	1.18695239955662
H	-3.47834809110444	1.23741495297373	2.44418717169318
C	-5.29141590541332	2.78577640958125	0.01371556569483
H	-6.07641330961878	1.82451910088606	-1.75582417784768
H	-4.37473243442294	3.45232509897789	1.85127107955342
H	-5.68058437295811	3.77138770724302	-0.24705052799745
H	-4.62541851777391	-1.76340255927566	1.38979974492302
C	-2.39977848961277	-2.69104244145764	2.40458641395770
O	-3.29611483821546	-3.44233825265196	2.67745587288327
O	-1.13665042716933	-3.09775296313830	2.40492306309766
H	-0.50445519037254	-2.41463107969417	2.11329125321863
C	0.24727097263756	3.23896538236827	1.56587426559605
H	0.11367153266461	4.11919702005181	2.21426859228110
H	-0.77892268094202	2.91139675289677	1.30980723356514
C	1.00438655405606	3.68656108409693	0.31774231138206
H	2.03759295729005	3.94439031940583	0.60541184805344
H	1.10396102642720	2.87691222258417	-0.42722758785843
C	0.37538836043315	4.88202378274249	-0.38386495694113
H	-0.70251238326290	4.69936393948292	-0.53374220644986
H	0.44089497529163	5.76061078983005	0.28871169044641
N	0.99305949057322	5.08466644102580	-1.68717346525494
H	0.61418257910322	5.91879758812656	-2.13229176172221
H	1.99070341054089	5.25995720140499	-1.56494941159206

76

Reduced Intermedia B_s

C	3.14594499394042	3.99881939922415	0.06849999544745
C	3.13784565376334	3.84522866714034	1.44417575115192
C	2.07725051558612	3.18180038722621	2.05576981853737
C	1.02149922231720	2.66481661207202	1.31740429664211
C	1.03776541961934	2.78113890270192	-0.08776888464597
C	2.10052312287084	3.46502158530659	-0.67947233050188
F	4.13873553520032	4.62868055009493	-0.53172408467578
F	4.14610537591197	4.28853265326541	2.16933574132264
F	2.14736147048670	3.02732378493696	3.36958834150306
I	-0.47442820344997	1.69211315156408	2.43069516261087
F	2.16961335105382	3.63092377554246	-1.99920771599034
C	-0.03666190040440	2.15345545426930	-0.94438073893705
O	-1.19889842835614	2.08028762926394	-0.58608103695172
N	0.37761153917787	1.60669384687739	-2.11428694744025
C	-0.58832625099116	0.94912228793864	-2.96619073712764
C	-0.02520382260795	0.69725693032088	-4.37293358587255
C	1.19348442143225	-0.19352447553816	-4.36695130821858
C	2.48394935354831	0.34784792205270	-4.40709915004248
C	3.60746871496562	-0.48086747080057	-4.35954964886993
C	3.45158959831281	-1.86368149218530	-4.27541595085536
C	2.16833547261863	-2.41485269601107	-4.23536473573748
C	1.04893375393614	-1.58588976837021	-4.28053515837703
C	-1.09850296977619	-0.35437072406971	-2.34858080899129
O	-2.13770699380221	-0.86467642764644	-2.74819820908285
N	-0.35701897204252	-0.88738539449213	-1.36105731313308
H	1.35021414040794	1.64882446966873	-2.40105404445476
H	-1.48367123104644	1.58316240508945	-3.04464627546779
H	-0.83276977748973	0.23960998090926	-4.96106232793265
H	0.21018189917434	1.67130924913999	-4.82503204527413
H	2.61169332661106	1.43128676551503	-4.49148982720422
H	4.60628000954914	-0.04309579721600	-4.39856971340904
H	4.32769295385176	-2.51336775603268	-4.24654678064926
H	2.03962950787719	-3.49706882016954	-4.17754711038429
H	0.04449698140076	-2.01874089786741	-4.25922194666266
H	0.53775366709897	-0.46643594394514	-1.14266591971855
C	-0.72740675555585	-2.13664329675684	-0.73635375803696
C	0.41041401141866	-2.74920348726314	0.08176029524171
C	-2.01539272185834	-2.04702220738492	0.09226445191108
O	-2.50994694999225	-3.07640773160436	0.51594472007517
C	-3.90653612630644	-0.67577805337592	0.81279918120878
H	0.00333477668320	-3.68457946049980	0.48922067551382
H	-1.00302552383628	-2.84945986554702	-1.53178478305453
H	-4.21723259727749	-1.68219154292042	1.13491318009005
H	1.22200687384455	-3.02790710916610	-0.61004710595285
N	-2.57243425921906	-0.82149735658885	0.27332383071335
H	-2.06612533870361	0.01868855263595	-0.00793789764681
C	-3.99625308660574	0.28896692952145	1.99076375161772
H	-3.66381225310386	1.28947040626725	1.66395846570313

C	-3.23328423584148	-0.11071773379672	3.23304419910933
C	-3.07253045330750	0.84457565206405	4.25033875265110
C	-2.70217510788967	-1.39129631005529	3.43458373600935
C	-2.39678356455711	0.53586052845504	5.43016739862919
H	-3.48661899827320	1.84676874109482	4.10947982103634
C	-2.03118179347578	-1.70315668621573	4.62077144959774
H	-2.80258928309866	-2.16195330153480	2.66729762150128
C	-1.87084163542190	-0.74359336058520	5.61907907194736
H	-2.28176256506556	1.29731724755290	6.20351951313931
H	-1.63571571129998	-2.71042309040477	4.76521403465379
H	-1.34268624485161	-0.99054119025849	6.54129562260036
H	-5.06358077460672	0.39188337204905	2.23421524578595
C	-4.93956728358424	-0.28085699449416	-0.25958677316361
O	-6.02380111984176	0.13479941096709	0.04953475668182
O	-4.61488035725324	-0.46149401437268	-1.53016983365943
C	0.97307993375846	-1.89640050495285	1.22666659695989
H	1.47933952930759	-2.58242110658427	1.92448914158099
H	0.14816982038244	-1.43741536865240	1.80003892115112
C	1.98340031165327	-0.82038084568637	0.81700740361074
H	2.65770191767674	-1.22752303641482	0.04192858259794
H	1.48919820307719	0.06196889138673	0.37649341131207
C	2.83549439583729	-0.30848414830506	1.97291516668105
H	2.18019694999627	0.16457186373997	2.72270393355262
H	3.30830087448294	-1.17539602659169	2.47559601978971
N	3.79029912942614	0.68850857120434	1.50151094215653
H	4.28207319848637	1.10212477124613	2.29297509885696
H	4.50728375897230	0.23724000900150	0.93374634376787
H	-3.69615196428454	-0.75672115895592	-1.68662729101433

76

Reduced TS2$_s$

C	3.07532043801428	4.03268256059723	0.12926645400422
C	3.08190270323506	3.86460367760191	1.50325803337631
C	2.04743189658292	3.16042952250689	2.11373732517711
C	1.00285242670008	2.62061529580363	1.37476909997010
C	0.99524912226029	2.76659192109079	-0.02741872544591
C	2.03493313054132	3.48763870124078	-0.61668258883677
F	4.04851164153243	4.69365422796917	-0.47045711289859
F	4.07903386380734	4.33711439606451	2.22628366720236
F	2.12554401187184	2.99893970959870	3.42624618502717
I	-0.43715013152876	1.56087211721508	2.47798092370451
F	2.08747399129458	3.68134665457007	-1.93415058726286
C	-0.08428547874359	2.14674704210167	-0.88662927464440
O	-1.24187203938958	2.05141041198512	-0.51955445449883
N	0.32507479668830	1.64188610284489	-2.07632855775695
C	-0.63076162604200	1.01435098127169	-2.96296501328260
C	0.00967853385560	0.71138162206311	-4.33066240111007
C	1.20722020605256	-0.20136059384404	-4.21528803539957
C	2.50297809434254	0.31990702168343	-4.11210770523191
C	3.59907523116683	-0.52272084012424	-3.91364067945389
C	3.41164061181871	-1.90141503346466	-3.82315973210452
C	2.12538319341303	-2.43421267155766	-3.93968610547877
C	1.03352170369026	-1.59027965683954	-4.13469701831705
C	-1.21215751334071	-0.26774815366174	-2.36025873009026
O	-2.29478034928173	-0.69544873187502	-2.74095795115432
N	-0.44829831426033	-0.87917311663442	-1.43934606234262
H	1.29574003067261	1.71073388827598	-2.36210381958159
H	-1.49576079145228	1.67895808752872	-3.10391239299000
H	-0.76905310687327	0.25275897051600	-4.95565535643652
H	0.29409333185706	1.66760585666994	-4.79304639785528
H	2.65941291354434	1.39970467592813	-4.19825993260758
H	4.60185305218661	-0.09906043254973	-3.83802756065886
H	4.26664593946844	-2.56242111998409	-3.67294872479371
H	1.97287625585066	-3.51347029720746	-3.88261793387882
H	0.02740998288945	-2.01015790737994	-4.22425339160028
H	0.48043277326202	-0.50678396028687	-1.28302912502178
C	-0.79715115025206	-2.14869212030354	-0.84038022850927
C	0.39564026047841	-2.79926065439374	-0.13652146928786
C	-2.00797667864095	-2.06933875326162	0.09722669833079
O	-2.50466674472104	-3.10392112077885	0.50480417223742
C	-3.83806338013103	-0.65448853572614	0.88007728074805
H	0.01835648034537	-3.75154939849627	0.26070680296161
H	-1.14511487236253	-2.82789930629565	-1.63716312446488
H	-4.20206611770049	-1.64974379903110	1.17383187638671
H	1.15519707519095	-3.04513756958090	-0.89725044502355
N	-2.47185248635103	-0.83724539366418	0.41626146730754

H	-1.98841561351969	-0.01109629169946	0.06545386071614
C	-3.96443420087644	0.33959933907920	2.01870270427079
H	-3.59204446608987	1.32111584823811	1.67937910197932
C	-3.25921406676387	-0.05761187740626	3.29740351932237
C	-3.18841564773799	0.87585655453443	4.34401017825577
C	-2.66769956919035	-1.31418532213692	3.48698181000174
C	-2.54296323288688	0.57011503508387	5.54110262211972
H	-3.64289009607726	1.86106117490091	4.20892174460005
C	-2.01880606602079	-1.62039640998927	4.68640847883998
H	-2.70145062015404	-2.06869291828844	2.69808948086646
C	-1.95052866853703	-0.68243513370583	5.71523676752522
H	-2.49870303670566	1.31424178195383	6.33823561909776
H	-1.56231181293805	-2.60381312608530	4.81175492303687
H	-1.43995769713484	-0.92437451534322	6.64864530959585
H	-5.03714414031670	0.48589112019318	2.22049508866538
C	-4.62950024872440	-0.23468092346634	-0.36131884311490
O	-5.20350823671871	0.80974797223562	-0.47114511519101
O	-4.58754397899382	-1.17478631197581	-1.32744179431252
C	1.04303786612978	-1.99474918641488	0.99801917137498
H	1.66341543125796	-2.69611322530247	1.57892429225862
H	0.26790187615906	-1.62688224669349	1.69456817505538
C	1.93592999125972	-0.83203310074903	0.55278008771584
H	2.50707997171695	-1.12955999365748	-0.34801207721510
H	1.33744939934120	0.05026304270488	0.27486393851400
C	2.92070614995318	-0.36362302381955	1.61712809369935
H	2.35508462207244	0.01567973070153	2.48513488291972
H	3.50021538078886	-1.23648462697570	1.97767177894048
N	3.75118880511645	0.71776423415049	1.09759594863112
H	4.34736468942657	1.08613427622131	1.83788943275020
H	4.38609939050558	0.34957137799851	0.38905860022568
H	-3.91941808588381	-0.95393053247254	-2.01182812955936

76

Reduced Intermedia C_s

C	3.03509260462744	4.24351503549720	-0.16693979073591
C	3.04827055471123	4.14250567096865	1.21693290483834
C	2.04107074650697	3.43306505606109	1.86405720813963
C	1.00882574443636	2.83303477779977	1.15199541813156
C	0.99699695509228	2.90926854707356	-0.25096748115961
C	2.01392461839570	3.62921731738059	-0.88365808378652
F	3.98239757924171	4.91555654704299	-0.79579770349586
F	4.02128052577297	4.70259629941708	1.91010717090335
F	2.12339774076280	3.33501290112433	3.18074537974281
I	-0.39545391756902	1.77596546441751	2.29593455410744
F	2.05364935366737	3.75509092559636	-2.20918660909543
C	-0.06576154569518	2.22457485024208	-1.08227273535488
O	-1.24153713101007	2.20849330429329	-0.76955571441680
N	0.40151889035207	1.59428556070506	-2.18577787296282
C	-0.49580064618726	0.95506012479109	-3.12185836090743
C	0.22218859721936	0.68591343026692	-4.45604764594220
C	1.43784320895505	-0.19266199969669	-4.27986454340638
C	2.71705897649022	0.36551359324979	-4.16299201895529
C	3.83095745036953	-0.44230264853348	-3.92312764554708
C	3.67808942916702	-1.82280304361451	-3.80260989383166
C	2.40849352255977	-2.39188827789814	-3.92853582187867
C	1.29794356073107	-1.58344305626961	-4.16480023246034
C	-1.09837584340165	-0.34992564422131	-2.58885715722009
O	-2.01463475881892	-0.88740800256721	-3.18064725025727
N	-0.53045539753321	-0.85337162878494	-1.46859543171368
H	1.39454224084339	1.60602122690893	-2.39482243046961
H	-1.35819957349368	1.61495740629816	-3.29975648633784
H	-0.50907751664906	0.20834538592960	-5.12206374193719
H	0.50648570856286	1.65204574338948	-4.89702396043216
H	2.84424827403947	1.44740633190657	-4.26818033336980
H	4.82046184613950	0.00982285107280	-3.83738294528148
H	4.54707110879747	-2.45688285700695	-3.61994714627890
H	2.28343498169522	-3.47307480978520	-3.84675039579666
H	0.30547208839467	-2.03087899685802	-4.26714347398931
H	0.30923800642006	-0.41275884358689	-1.11478850576379
C	-0.95058078968988	-2.11972108570239	-0.91929437905434
C	0.17926925981545	-2.85683416740523	-0.19665087123189
C	-2.18112865747984	-2.02568039919926	-0.01018920610274
O	-2.67802549498227	-3.05300950046481	0.42205275213677
C	-3.92199118908548	-0.62568431718113	0.95677402702211
H	-0.23778655450214	-3.83278327977878	0.08585914621092
H	-1.29689582042817	-2.74504035113626	-1.75791549106242

H	-4.21504637492086	-1.63279498525166	1.29670962429846
H	0.98787798165311	-3.04566985271641	-0.92154223556591
N	-2.65085464549124	-0.79060885912076	0.28714134057308
H	-2.20418687642704	0.02737214113162	-0.12500866745092
C	-3.88474445926372	0.32845941654132	2.14340568483739
H	-3.57179235588322	1.32732345680780	1.79299143942991
C	-3.00248416783800	-0.09764804886012	3.29513986144323
C	-2.78838264313334	0.81244753399470	4.34322615267449
C	-2.38908496038074	-1.35478663376434	3.37205540791654
C	-1.97541048605998	0.48609653236595	5.42782696691176
H	-3.26406850836461	1.79601562481091	4.29876042013015
C	-1.57075513646614	-1.68088558611906	4.45719540774801
H	-2.53797892377780	-2.09673442196031	2.58466698404544
C	-1.35566071105279	-0.76384967456947	5.48470347825202
H	-1.82086353812743	1.21319504069008	6.22688178518067
H	-1.09699211773564	-2.66371332959069	4.49252860508204
H	-0.71298320152385	-1.02134855272718	6.32793281714116
H	-4.91863207325412	0.44930464556828	2.49839198538122
C	-5.02391357229387	-0.21465105958285	-0.02030737350848
O	-5.97178109767919	0.46168981775287	0.27749813370057
O	-4.84096797664294	-0.73077666870244	-1.23171565863764
C	0.74068576661226	-2.17546822538818	1.05575153799091
H	1.27588193486385	-2.94540919666489	1.63552649681364
H	-0.08804583225294	-1.83629520361081	1.70433665937100
C	1.70757518160527	-1.01107806922605	0.82862284136598
H	2.41784016957749	-1.25883659465861	0.01813773837810
H	1.17459018249703	-0.09466091691870	0.52278320153740
C	2.47899024241534	-0.66042781678912	2.09596188149840
H	1.75190723584050	-0.49176763233010	2.90729261988290
H	3.08491364778484	-1.53897528248535	2.39506371020516
N	3.25786884850365	0.55887907555295	1.91944114844928
H	3.68794741797735	0.83235103365156	2.80149200809158
H	4.02855519788783	0.38532776842962	1.27421888696090
H	-5.58811941708826	-0.46650897487434	-1.79359711762235

76

Reduced Intermedia D_s

C	2.18664426619006	4.86144701792489	-1.14959115104079
C	2.13247711428453	5.10976466456119	0.21505297884551
C	1.34003732774910	4.30527281909331	1.03420922558836
C	0.59087146007539	3.26564622957940	0.49728825596330
C	0.63533338323679	3.00996279896576	-0.87821982987199
C	1.44105778709828	3.81166476306670	-1.68275159958954
F	2.93282914930435	5.61733711004312	-1.93271699521557
F	2.83211878100374	6.09902009948436	0.73559718359796
F	1.33926400204554	4.56115098861549	2.33230767451645
I	-0.47961157234359	2.06730041856737	1.83579111864450
F	1.50962178935464	3.61079343253483	-2.99335283083355
C	-0.18800422601152	1.93649506176000	-1.55704955331081
O	-1.41012069540363	1.96645247224290	-1.55465542987799
N	0.55491684488819	1.01483983725814	-2.18961857022276
C	0.03607826214336	0.05273288193164	-3.14834201395766
C	1.12697375171724	-0.29309280781255	-4.17589153932843
C	2.49023602513250	-0.53917636301952	-3.56442541802704
C	3.41029739378392	0.51524193337319	-3.46825059900688
C	4.66319897365638	0.32502568907666	-2.88222745137619
C	5.01906500567448	-0.93042408042224	-2.38861465905512
C	4.11174462570299	-1.98851800746845	-2.47945515735863
C	2.85674311240069	-1.79486533575996	-3.05851984678560
C	-0.54560698930479	-1.21565719551896	-2.48364081725305
O	-0.17015412178994	-2.33607520844688	-2.77951337105269
N	-1.50137290730652	-0.97066906367251	-1.56185432067734
H	1.53425048850415	0.93926462185541	-1.91135484140602
H	-0.80590654789598	0.54332847174506	-3.66374746723515
H	0.79140711776097	-1.18005884662674	-4.72733611795664
H	1.20049416124209	0.54532980319294	-4.88180616118994
H	3.13778309121111	1.50015959458046	-3.85694225429931
H	5.36421017944692	1.15898888110754	-2.81871049172201
H	5.99983348844806	-1.08491515321731	-1.93573898140349
H	4.38338476827527	-2.97447211822278	-2.09809831945664
H	2.14157531847319	-2.61591830150503	-3.12001137291310
H	-1.87449358129358	-0.02248312397020	-1.51829718392582
C	-2.08556520256244	-2.03770252297157	-0.76379825668025
C	-1.08447912043926	-2.81633370940046	0.12429583285531
C	-3.23296945854514	-1.51944715308521	0.09427777948893
O	-4.19340145858520	-2.23080727531928	0.32570829271601

C	-4.13189006666306	0.32743900010048	1.41728241204714
H	-1.69967637649572	-3.50788475156000	0.72162026321425
H	-2.55323067923226	-2.77693380591994	-1.43178397237396
H	-4.96538610651149	-0.39282202173130	1.38120653651066
H	-0.46960585381338	-3.43231892811101	-0.54734078582804
N	-3.08921097575768	-0.27501789413495	0.62056158289520
H	-2.26362357087608	0.27445114950788	0.40045650671089
C	-3.76222447504210	0.60845214116472	2.88630804337773
H	-3.28916614458402	1.59796120783243	2.96396789777155
C	-2.87079201060343	-0.43686981973598	3.51176294819481
C	-1.77155349135211	-0.04768335421792	4.28640796610732
C	-3.10922515537481	-1.80726485610647	3.33357068571464
C	-0.91595330380185	-0.99625703606694	4.85304838216933
H	-1.59022016928769	1.01599183831873	4.46810412184159
C	-2.26085811048411	-2.75592252343591	3.90192927703843
H	-3.95267957316797	-2.13853120937230	2.72370659848842
C	-1.15696080215131	-2.35466493709577	4.65839757523529
H	-0.06316751606483	-0.66822617075131	5.45004169293272
H	-2.46009761581805	-3.81803430298579	3.74709884175783
H	-0.49058323778560	-3.09968381669621	5.09610379770814
H	-4.70515873569159	0.69427848988348	3.44826101809403
C	-4.66453067348632	1.59752847300328	0.76118107828086
O	-5.03981004010125	2.56127051300962	1.37243154532114
O	-4.69554900326139	1.50896872791173	-0.56654200362304
C	-0.15803655639903	-2.01831652228873	1.05022625657252
H	0.37020359182599	-2.74983612940202	1.68536900396040
H	-0.74323760730827	-1.38852394062320	1.74562647629464
C	0.85911694260002	-1.16338872862217	0.29807602572340
H	1.22959360916439	-1.71882900780073	-0.58344963485656
H	0.35825719327005	-0.27073407370102	-0.08898789795509
C	2.06250419582857	-0.66946408882658	1.08143067983019
H	1.72441107505652	-0.13474741815996	1.98409956884494
H	2.66866995390334	-1.52934400392292	1.42876810789195
N	2.80710012103382	0.26535129032829	0.23324370085613
H	3.60784974481251	0.64515921851509	0.73606486313446
H	3.20287366502498	-0.24841413904182	-0.55741240473635
H	-5.04756360243806	2.34396309890951	-0.91734571103525

76

Reduced $TS3_s$

C	2.43351392035381	4.65624456564919	-1.11197798452734
C	2.32430435847661	4.93477483734089	0.24348986305147
C	1.37970048038645	4.25713212921677	1.01362779775367
C	0.54477411246120	3.30537303337017	0.43904215516354
C	0.65531636470341	3.00618306592470	-0.92368426870722
C	1.60162204617856	3.69419005927251	-1.68040333350194
F	3.31528024884671	5.30013175708333	-1.85262708048418
F	3.10671282693274	5.83929595310837	0.79894174726082
F	1.31749783953504	4.54629714031353	2.30335555695219
I	-0.79635350832870	2.34017699664669	1.72165185731504
F	1.73323080288394	3.46501902805824	-2.98134549721745
C	-0.19338337507145	1.96973736649198	-1.63231619090928
O	-1.41099518506144	2.04388715992425	-1.68412073567202
N	0.54435932141538	1.01063971076994	-2.21531727267255
C	0.02651561491369	0.03789229512189	-3.16102382848284
C	1.11662313585600	-0.31639609087927	-4.18742981791689
C	2.48323351132432	-0.53911643111230	-3.57395725933342
C	3.39903371133072	0.52133851179126	-3.51525602983360
C	4.65370135828324	0.35639649793400	-2.92606799544742
C	5.01515177878060	-0.87934551937114	-2.38809011412918
C	4.11153090070898	-1.94311750076689	-2.43979777706560
C	2.85530631748260	-1.77480546199152	-3.02493077170628
C	-0.54451887772182	-1.22501050839825	-2.47804324615033
O	-0.16701456950656	-2.34654770052315	-2.76814095311457
N	-1.48870311660171	-0.98402512752549	-1.54220728277236
H	1.50088657348916	0.89730951583857	-1.86940526746912
H	-0.82041866848544	0.51898531474056	-3.67660103665175
H	0.78772078705705	-1.21416573542736	-4.72532771310256
H	1.18199955156576	0.51242548372205	-4.90527281619823
H	3.12110222117820	1.49160310890544	-3.93399239953341
H	5.35161992328913	1.19485295973866	-2.89307554444187
H	5.99729453542493	-1.01417372336661	-1.93174517410383
H	4.38732190825396	-2.91400932871318	-2.02417549987862
H	2.14387486609883	-2.60079230269799	-3.05736764568926
H	-1.86559776333358	-0.03846708148141	-1.47823653712124
C	-2.01550556882244	-2.06001907056749	-0.71259449104946

C	-0.96387044472362	-2.77393305744013	0.17445597905040
C	-3.17283549132704	-1.57518633723273	0.15087172261713
O	-4.14066586877520	-2.28499036213567	0.35016831708499
C	-4.03372115473719	0.22596662714998	1.56803578051334
H	-1.53146907646988	-3.49310560411602	0.78634874086081
H	-2.45518459377342	-2.83094464365861	-1.36239701394431
H	-4.87730077820867	-0.48181547566382	1.51836108977812
H	-0.32625546049976	-3.36224313992220	-0.50084030913498
N	-3.01967669063169	-0.35049259317893	0.71898307410471
H	-2.16770278361489	0.17442142790478	0.54907099507977
C	-3.62219626021162	0.42888386223037	3.03919097409101
H	-3.13624531349413	1.40928798824314	3.15907006666879
C	-2.72985220960085	-0.65492655270820	3.59263519763020
C	-1.58844958319740	-0.31504139568366	4.32750526862893
C	-3.00861573476481	-2.01188500829869	3.37787332992527
C	-0.73923060517201	-1.30137072414864	4.83377655246294
H	-1.36093865422458	0.73839073460796	4.51338400705074
C	-2.16106310365348	-2.99850094659319	3.87956196459253
H	-3.88337253332405	-2.30240424004911	2.79158057973234
C	-1.02201061432607	-2.64718550552277	4.60765340788115
H	0.14582925467415	-1.01374597211910	5.40391385969954
H	-2.39075881401476	-4.04998386190269	3.69703089564650
H	-0.35845206534949	-3.42130630073839	4.99658074697826
H	-4.54874677002148	0.50527595380323	3.62911647007962
C	-4.56326761002795	1.53561407068102	0.98954370624460
O	-4.89555734507011	2.47685688736419	1.65853351142613
O	-4.63944434004037	1.50983258281898	-0.33814055865978
C	-0.06723143659694	-1.92249519109425	1.08495922052127
H	0.53764487812480	-2.62340375948777	1.68503528187997
H	-0.67165594794601	-1.35330802959071	1.81324580540148
C	0.85374555801879	-0.98230539730460	0.31161019321897
H	1.24637854406824	-1.50677054237297	-0.58012148923940
H	0.27011094622577	-0.13713445735675	-0.06582686243489
C	2.03699808530432	-0.37943049372179	1.04887604541631
H	1.67604704773940	0.24274546263901	1.88484816465530
H	2.66328573612791	-1.17781916444793	1.49251886200702
N	2.76840506550257	0.47660920960547	0.10809013994843
H	3.48471765448010	1.01457476117181	0.59364046369871
H	3.27371725484698	-0.11275139892917	-0.55731402966048
H	-4.98512212559343	2.36641867905761	-0.64002456411433

76

Reduced Intermedia E_s

C	2.78604063576424	4.17067030831957	-0.39201326986381
C	2.24240430240618	4.67987966423493	0.77728937896964
C	0.92897582778935	4.36125157842209	1.11750115331944
C	0.15414082112654	3.54379741721468	0.30081416097570
C	0.69801545023150	3.01253892826761	-0.88100735445521
C	2.01195870120444	3.34950639738356	-1.20695359107608
F	4.02935821410027	4.46238955266863	-0.72971909902497
F	2.96327082517142	5.45776164529608	1.56145774746634
F	0.45371645002747	4.86557870123650	2.24608578436681
I	-1.79769307615717	3.16905079369640	0.97516042454820
F	2.58962545106601	2.90139981760689	-2.31295157528096
C	-0.09396606225996	2.10292788455734	-1.80111600939388
O	-1.25927004404323	2.32863157012430	-2.08411795828648
N	0.59653400106225	1.03923793782568	-2.25091512539690
C	0.04891578951254	0.10112516408397	-3.21222231023767
C	1.10886721267473	-0.29992315442710	-4.25134700270698
C	2.46619329133706	-0.62186332324858	-3.66141905749400
C	3.47801741561727	0.34660615129185	-3.68670896084763
C	4.73404710364404	0.09285907134937	-3.13356706188349
C	5.00091464740562	-1.14478547925971	-2.54686831589532
C	4.00191649314796	-2.12083625043199	-2.51710182771650
C	2.74538035044317	-1.86245642185288	-3.06791678810167
C	-0.57865009250506	-1.14061356978367	-2.53884405475119
O	-0.30403466960287	-2.27321590914021	-2.89544138658675
N	-1.44474180176274	-0.89064803550935	-1.52944493663025
H	1.50512478871135	0.84296689198507	-1.81384348783171
H	-0.77763828436776	0.62268432565896	-3.71998641984853
H	0.71890293562200	-1.16195803839499	-4.80728914076711
H	1.22027388639778	0.53776598096599	-4.95289058517012
H	3.27624364806735	1.31581797922899	-4.14728677021256
H	5.50603831350065	0.86371937748408	-3.16650171824295
H	5.98330864614581	-1.35021118377017	-2.11821628577470
H	4.20319546326798	-3.09452521411693	-2.06670997387665

H	1.96485060659473	-2.62375125935684	-3.04444603348139
H	-1.74939536249523	0.06734970666205	-1.37059456602782
C	-1.93693079841112	-1.99051585133120	-0.70736899147309
C	-0.84240371810017	-2.72380587353425	0.11004293615218
C	-3.07088510356313	-1.57143036220201	0.21759574337484
O	-4.00884941990225	-2.31795068122385	0.41829948487632
C	-3.90139975581276	0.06761229841899	1.83499689054949
H	-1.37159671844906	-3.48097241720676	0.71032772412222
H	-2.38652961779481	-2.74342543248852	-1.36996054082406
H	-4.65015491301330	-0.73657230364834	1.88301644530373
H	-0.22232569925343	-3.26915887572897	-0.61630222244070
N	-2.93753438197976	-0.36996138216196	0.84499340839681
H	-2.07589036896385	0.16021363210762	0.74916030207844
C	-3.29322087785529	0.32763602582810	3.21436416451379
H	-2.75067265084830	1.28340373636757	3.20181259840755
C	-2.36743335895409	-0.76782179500184	3.68688288928990
C	-1.17101912657612	-0.43315437677972	4.33167446764175
C	-2.66236073755552	-2.12278683954106	3.48190263758259
C	-0.29055100631235	-1.42370981240705	4.76969708472443
H	-0.92459350122287	0.62052816284433	4.48604180507903
C	-1.78113702721145	-3.11399409110462	3.91306121509649
H	-3.57588662163964	-2.41325854666689	2.95766925386604
C	-0.59282388017950	-2.76860865761846	4.55954503604538
H	0.63734110898904	-1.14162946008545	5.27052002506012
H	-2.02382697489665	-4.16376261486334	3.73856438533315
H	0.09600055408831	-3.54595872517263	4.89412271555670
H	-4.12585469531162	0.46363845502711	3.92327162634804
C	-4.63835150362591	1.29967331107190	1.31817981340975
O	-4.60517526987955	2.38834665206917	1.83713942675256
O	-5.30892071640264	1.04227619235607	0.20379021531875
C	0.07576332864403	-1.89575594572043	1.01985298369787
H	0.75239652750944	-2.60721989469110	1.52283362267558
H	-0.49913253657129	-1.41262998837983	1.82678398797649
C	0.89771475291922	-0.86709144752994	0.24758933058040
H	1.19735922897146	-1.31357304770933	-0.71880504605942
H	0.27090370647822	-0.00179561618546	-0.00104850343259
C	2.16552775436354	-0.31461092374312	0.88078257931608
H	1.91221603203888	0.35059531298368	1.72203325168103
H	2.77993641405500	-1.13595104770117	1.29667622038483
N	2.87106440136137	0.46683760691975	-0.14321423110377
H	3.58848857386612	1.05691303549414	0.27560842467847
H	3.36927081133619	-0.16794222669377	-0.77100759719542
H	-5.73039609318011	1.86236980936048	-0.10495354612460

76

Reduced $TS4_s$

C	2.62504827283523	4.46663597579913	-0.98373852883374
C	2.40824373667777	4.80347898675020	0.34516344517568
C	1.31575069763754	4.26404253396055	1.02240931160779
C	0.44329842496878	3.38812341658335	0.38414549445427
C	0.66617537476946	3.02077690583185	-0.94867951960029
C	1.75496721002618	3.58165145910692	-1.61437931640086
F	3.64462077947167	4.98538400720973	-1.64183543681272
F	3.22310636467002	5.64008585365811	0.95798592172880
F	1.14156537367089	4.61815804649048	2.28573200725807
I	-1.17589430105230	2.70237069721847	1.51844642514471
F	2.00018654493041	3.29983784480458	-2.88792467037706
C	-0.19322038390835	2.02232374152492	-1.70059722225427
O	-1.40351148351227	2.13818530109272	-1.80683417042167
N	0.53132544438531	1.02633720818671	-2.23840805403607
C	0.01233101322034	0.05812318942346	-3.18652875175219
C	1.10470289161533	-0.30867303905676	-4.20692574722463
C	2.46406151141765	-0.54936822415539	-3.58295164853076
C	3.41083778346207	0.48438053115286	-3.56455536822917
C	4.65809752351373	0.30825821091961	-2.96321575891444
C	4.98118214435484	-0.91296530182237	-2.37043180255452
C	4.04745280978184	-1.95154862161572	-2.38342598065696
C	2.79936056504849	-1.77215725305360	-2.98264100688147
C	-0.56793329283853	-1.20253090760501	-2.50666872437353
O	-0.22480234914607	-2.32625370252667	-2.83075961976782
N	-1.47517737005767	-0.96831992612018	-1.53310054553065
H	1.47412773704871	0.89392617396392	-1.85962802436210
H	-0.82953691633126	0.54411547575565	-3.70556681680580
H	0.76759934536842	-1.20106929251135	-4.74881209084825
H	1.18621977296343	0.52044234280165	-4.92264393544863
H	3.16390703322719	1.44286157971697	-4.02575638074686

H	5.38000288731778	1.12689866723131	-2.96341395986467
H	5.95726811906300	-1.05683861103623	-1.90393614351193
H	4.29355926596411	-2.91241780158595	-1.92779910960746
H	2.06735138177001	-2.58054522149788	-2.98838245790337
H	-1.82345493456458	-0.01786665606420	-1.41327800307280
C	-1.95154980993035	-2.06018136212451	-0.69133647790800
C	-0.84981448417367	-2.74693069998605	0.15549352577037
C	-3.09627617773002	-1.61312845438258	0.20731918462454
O	-4.06493064341863	-2.32494054936339	0.39050109306835
C	-3.91208682422094	0.11484538433182	1.74055581397935
H	-1.36596739218633	-3.51149399752120	0.75788502611180
H	-2.38594916897090	-2.83808563986342	-1.33529063701319
H	-4.74917801841429	-0.59985304456168	1.69703531993979
H	-0.20037203662035	-3.28283728661345	-0.55175463568734
N	-2.92823150478696	-0.41320648046426	0.82433733322005
H	-2.05643940584867	0.09292565666598	0.70129102552592
C	-3.42887486737201	0.27060963039396	3.19228084732620
H	-2.92110080764094	1.23808056150394	3.31667454988693
C	-2.51826385721102	-0.83334275454360	3.66873249211464
C	-1.33478626317066	-0.51569325406405	4.34478573614832
C	-2.81768349649233	-2.18227996035226	3.43438414953544
C	-0.46838201107167	-1.51902841291071	4.78274964075742
H	-1.08588500354189	0.53364585536573	4.52378677941710
C	-1.95047655307372	-3.18558511636750	3.86497002672835
H	-3.72409835544751	-2.45301737284034	2.88750730705090
C	-0.77299110269547	-2.85770044396902	4.54085668299312
H	0.45007598182429	-1.25121136541227	5.30805960932299
H	-2.19500222758320	-4.23110977681607	3.66830514730824
H	-0.09502710345286	-3.64476070912723	4.87513564932695
H	-4.32424948148279	0.33717991054199	3.83029701895419
C	-4.46353724341010	1.44114992421121	1.22114452631524
O	-4.57785958670744	2.43402165588765	1.89106089579467
O	-4.80124647646941	1.37528266867490	-0.06199273781378
C	0.02498872813565	-1.88036193922025	1.07291385922909
H	0.71616584889094	-2.56457296829530	1.59361887902323
H	-0.57919112342967	-1.41073906403634	1.86651584550560
C	0.82880471057396	-0.82732222602484	0.31539724199811
H	1.21217202942692	-1.27620847586324	-0.62060027869206
H	0.16851178405831	-0.00764508638076	0.00802400087244
C	2.01055104390291	-0.17973526383884	1.01943056623743
H	1.64716965843783	0.48234158017821	1.82365917048035
H	2.64398339176775	-0.94975665979950	1.50074794129243
N	2.73705512026139	0.63030697530259	0.03425700598996
H	3.40359326452859	1.24854546142169	0.49438356333456
H	3.29651253356497	0.01532196090363	-0.56104862207899
H	-5.12714701489647	2.24774737737693	-0.34014562308171

76

Reduced Intermedia F_s

C	2.83538278135631	4.13769625178898	-0.39551577603373
C	2.94406505254653	4.04555410612356	0.98520508610793
C	2.03262387204321	3.26785090621921	1.69806358930178
C	1.01993104256345	2.57104143097602	1.04664233791774
C	0.92022750276638	2.63746165343953	-0.34941844818099
C	1.82479329386814	3.43642694521683	-1.04652207978681
F	3.68124281654423	4.88233752812870	-1.08285322251814
F	3.90017617269004	4.69687648964673	1.61933070479825
F	2.17590040128426	3.21338023367737	3.01344313201312
I	-0.26404504786052	1.44394962275513	2.26154696773890
F	1.75563405095085	3.55890710232348	-2.36721458273016
C	-0.06548085396820	1.82101833420860	-1.15849257804912
O	-1.27624903541273	1.87102859446569	-0.98856108126499
N	0.55494295801363	1.05638979521215	-2.07287950817204
C	-0.10959431717370	0.25978664053323	-3.09014202049394
C	0.80414591773979	0.11441247681574	-4.31996247293766
C	2.25401489184794	-0.15578166564287	-3.97643983332704
C	3.14329434989501	0.91866528529284	-3.82876632474265
C	4.47434241588315	0.70685654656747	-3.46692823161702
C	4.94305458072703	-0.59138627961794	-3.26161866524357
C	4.06899893893448	-1.67023800407348	-3.41282018110312
C	2.73424885849209	-1.45528963465996	-3.76151249054905
C	-0.55980358639016	-1.11451937053219	-2.54334457855325
O	-0.17462600253532	-2.16811708521024	-3.01672522517112
N	-1.39809744057883	-1.02868560540907	-1.48775036614968
H	1.55288798660069	0.89261329107008	-1.91291514206608
H	-1.02502842228786	0.80294761667656	-3.37514900446204

H	0.40019467238561	-0.69654326566773	-4.93886828290569
H	0.74473693518196	1.04878777961507	-4.89431544348328
H	2.78327619857950	1.93759423131298	-3.98969834870810
H	5.14537717075999	1.56004207015937	-3.35092370771735
H	5.98496486541937	-0.76295714540655	-2.98601095354640
H	4.42829235469827	-2.68922244976377	-3.25804740260859
H	2.04661044283689	-2.29540219530947	-3.86496177423423
H	-1.79175945537518	-0.10955098986459	-1.29925192496041
C	-1.83013181946865	-2.19797858693851	-0.74136844078831
C	-0.69803450893001	-3.02997761869202	-0.09464340906836
C	-2.89193648121997	-1.86475259826527	0.30312739840113
O	-3.61458189384789	-2.75971996434661	0.70621551703562
C	-4.13608228248103	-0.18627551551657	1.54585867328255
H	-1.20987945328564	-3.82819950280178	0.46331915014478
H	-2.35383225497880	-2.88348914970769	-1.42703843169710
H	-4.66103348904131	-1.11289238626630	1.82367959079332
H	-0.14392482210921	-3.51348529369859	-0.91149201707126
N	-2.98531858435880	-0.59125558867414	0.77115668541605
H	-2.35591420818116	0.13823235119153	0.44660473350350
C	-3.80838350816022	0.59946545350254	2.81662061936342
H	-3.38906059635098	1.58242524168604	2.54402302706905
C	-2.87747802629537	-0.09381509952053	3.78520308062505
C	-2.28745506304777	0.66485557143257	4.80830560478369
C	-2.58920682296767	-1.46372993102947	3.72020334359631
C	-1.42624883658396	0.07850831326024	5.73477682824307
H	-2.50599407038897	1.73423973163361	4.87365623284760
C	-1.72546966246032	-2.05116113980636	4.64780942212820
H	-3.03390951430229	-2.08923147817501	2.94298587858015
C	-1.13822939969581	-1.28480369879493	5.65371783613383
H	-0.97633564995052	0.69007579561710	6.51867212234286
H	-1.51171609454070	-3.11932897695495	4.57970348794095
H	-0.46085460239718	-1.74735502442503	6.37318894906836
H	-4.76834418573312	0.81134762039634	3.31125408540356
C	-5.14333228887470	0.61071386429676	0.71966285985122
O	-6.11765843597425	1.12697446021326	1.19629896227583
O	-4.84041045590183	0.66990535506510	-0.57567374173667
C	0.29570782565524	-2.30136157504734	0.81806090941544
H	0.93186092853298	-3.06817433671579	1.29072718832504
H	-0.23440504058159	-1.78995314279491	1.64216228897884
C	1.16262953741993	-1.30398124393514	0.05373865749242
H	1.41084096519133	-1.73028361617889	-0.93580328224117
H	0.56687265638657	-0.40853783379705	-0.14055314703641
C	2.46402603793981	-0.83394172214738	0.68118807205136
H	2.25880567906888	-0.30003862568839	1.62380928468921
H	3.10115472555386	-1.70214981330851	0.93989548853902
N	3.10184432946414	0.09680183294196	-0.25588146600680
H	3.92885581258324	0.52032512230491	0.16257210269975
H	3.44155597542255	-0.42328128487393	-1.06808354592113
H	-5.53623544803035	1.18142550818563	-1.02191821366671

76

Reduced $TS5_s$

C	3.17258960904834	4.00516261640580	-0.15498943809193
C	3.19591382519204	3.87516152203477	1.22695193929979
C	2.18512587483596	3.16210024460301	1.87020557360717
C	1.15531115054250	2.57012543544838	1.14592550424740
C	1.13386520746372	2.68244583749257	-0.24956078638527
C	2.14235513454559	3.41047954547492	-0.87767328936384
F	4.11911795733646	4.68599570405284	-0.77380319542176
F	4.16571861808730	4.43195691941383	1.92663889342236
F	2.24914396033608	3.06614762669289	3.18940051575307
I	-0.27707774695605	1.51488653558986	2.25381156698392
F	2.15514604269396	3.56569512001556	-2.19616371984978
C	0.10369000538419	1.99902111006173	-1.12424758916392
O	-1.09741821454872	2.18387184715901	-1.00914485001851
N	0.67267020860892	1.18606555174897	-2.03107070466483
C	-0.04608845167661	0.52702618916537	-3.10512298392666
C	0.87310046545746	0.38030276516095	-4.33106923721718
C	2.27820520963140	-0.06801242527835	-3.98584008511115
C	3.28374153566002	0.89023430901466	-3.79334367584073
C	4.57763652380605	0.51285600030378	-3.43074793160432
C	4.89036802666765	-0.83680736337478	-3.26428018171269
C	3.89831982781884	-1.80107954486086	-3.45582562561504
C	2.60223573306784	-1.42100939699985	-3.80935245835210
C	-0.64046000018763	-0.83297378684502	-2.67799007869341
O	-0.39025076821519	-1.86668592069446	-3.27281816059996

N	-1.45765680585975	-0.79425448975601	-1.60093144290469
H	1.63983824637428	0.90501244963550	-1.84349433089973
H	-0.90096711467421	1.17415136236792	-3.35876327045777
H	0.40091571106163	-0.33097656657417	-5.01997125042008
H	0.92580486927470	1.35884063442326	-4.82702200956840
H	3.04533518497854	1.94854676019557	-3.92170809805373
H	5.34394426263794	1.27636736007011	-3.28544754189661
H	5.90274228502576	-1.13691454875386	-2.98835976822656
H	4.13483485848196	-2.85946514925595	-3.33188675521213
H	1.82320736632789	-2.17150501193253	-3.94719742685775
H	-1.70701745783269	0.11469409785388	-1.21313168343074
C	-1.96638790484643	-2.03804713337538	-1.04020750299759
C	-0.90100468414885	-2.94355166489143	-0.37118784993353
C	-3.14256240366533	-1.80393463290196	-0.10510789024435
O	-4.12604235518858	-2.52822538856346	-0.15275788770781
C	-4.17073228721710	-0.60865451796884	1.70775062650641
H	-1.45834696827281	-3.75878234831052	0.11659687057206
H	-2.39515485117642	-2.62706529741746	-1.86269376587898
H	-4.39415974343564	-1.56778562926189	2.20500401003565
H	-0.32437514857197	-3.40400225339628	-1.18696760791736
N	-3.05664323332686	-0.80911191868485	0.80867684669164
H	-2.25705955750062	-0.18249563773749	0.84017133317423
C	-3.89056885246767	0.44242845208346	2.77538815596854
H	-3.61329235793430	1.39442267574508	2.28929839457330
C	-2.83828306776867	0.06645803821755	3.79815415435574
C	-2.52941091743752	0.99087204698926	4.80927282232735
C	-2.16252998467864	-1.16013890859058	3.79021690381373
C	-1.57118578108374	0.70234052229027	5.77767803105459
H	-3.04970077335455	1.95188284955097	4.82799533052244
C	-1.19570297803925	-1.44869731614352	4.75785134974248
H	-2.38020632749316	-1.90447558526501	3.02240466806961
C	-0.89503521237230	-0.52011045472746	5.75163476035176
H	-1.34649329241025	1.43678557833163	6.55265686051129
H	-0.67656295653258	-2.40826144481619	4.72834761857921
H	-0.13887170006304	-0.74571612739362	6.50496141449826
H	-4.84166484831131	0.62767211561840	3.29590355004189
C	-5.48095710817599	-0.28188027033753	0.97493566070705
O	-6.51664248009357	-0.24165038572373	1.56923774163438
O	-5.37862348576745	-0.00377723520118	-0.34508947484512
C	0.08715657281672	-2.31003658280787	0.61785502194538
H	0.66797946620164	-3.13218594844171	1.06830688361227
H	-0.44551417706013	-1.81966815068418	1.45252461280980
C	1.03075848057836	-1.31697522115431	-0.05796313699799
H	1.27538194307294	-1.68627228715022	-1.07197797149238
H	0.50125410670334	-0.37191744729949	-0.19891496520985
C	2.34371764284554	-0.97194712809770	0.62512501373750
H	2.14619392842609	-0.46437009248510	1.58410076180769
H	2.91109382065080	-1.89303114531343	0.86199770293839
N	3.07307283253286	-0.04940276260206	-0.25177517145679
H	3.88838355033233	0.33423998846992	0.22387760211752
H	3.43869483861186	-0.56344850360969	-1.05639876222074
H	-5.55210785308160	-0.81182635845219	-0.85586688659647

76

Reduced Intermedia G_s

C	3.16329959716027	4.02940753940816	-0.14128139714959
C	3.17413655246803	3.89664974636143	1.24051787280426
C	2.16354337476852	3.17327256171026	1.87281883835571
C	1.14518909549648	2.57471135624890	1.13812642335696
C	1.13611145411236	2.69162834045322	-0.25760889332395
C	2.14517989364518	3.42759466159809	-0.87516067688297
F	4.11041569011572	4.71851617873340	-0.74967732787472
F	4.13284129434885	4.45903577027438	1.95072675146077
F	2.21763089844250	3.07342021204910	3.19206824862403
I	-0.28252422286450	1.49303563477819	2.22758757658981
F	2.17087411091738	3.58237011564659	-2.19331388832851
C	0.11690454688478	2.00256025161879	-1.13832390333735
O	-1.08764977521080	2.16861089656790	-1.01710713396791
N	0.69169870579545	1.20121507225744	-2.05110827603185
C	-0.02727744013115	0.52142673648903	-3.11306185409204
C	0.88897983689744	0.35352818434535	-4.33772721468494
C	2.29619802590697	-0.08392188274141	-3.98779391904332
C	3.30191276095549	0.87993587771321	-3.82782887440505
C	4.59869805839579	0.51309377753183	-3.46469037763255
C	4.91372857876402	-0.83145192866470	-3.26493808632742
C	3.92098558390348	-1.80122756536511	-3.42252657386842

C	2.62197268909473	-1.43145643791004	-3.77606677571140
C	-0.61936692901743	-0.83122516995909	-2.65870759456991
O	-0.37967956479239	-1.87578620043203	-3.23735022185515
N	-1.42553393819250	-0.76794059300270	-1.57323654806909
H	1.66437765896498	0.93434746078257	-1.87133569226231
H	-0.88424849674736	1.16147227325600	-3.37772351703081
H	0.41668129020843	-0.37298282459740	-5.01051903875348
H	0.93690235604476	1.32167735064822	-4.85400862297298
H	3.06195470891435	1.93421681571398	-3.98408173945046
H	5.36555117898608	1.28054837109348	-3.34578271153375
H	5.92850934946540	-1.12337601880793	-2.98917535472753
H	4.15930656067659	-2.85571340896591	-3.27183903469185
H	1.84242100750452	-2.18590279685498	-3.88775733728338
H	-1.65762604400086	0.15475286672247	-1.20959428354438
C	-1.93229215460661	-1.99681599942936	-0.98541636014343
C	-0.86432905258337	-2.90110746425211	-0.31799777211745
C	-3.10429563395670	-1.77559429458284	-0.04379152856146
O	-3.95904974074701	-2.65437108793863	0.05383538734472
C	-4.26948675446891	-0.47126600824447	1.65792604241199
H	-1.42086792451265	-3.70813579943642	0.18301280486291
H	-2.36818460288886	-2.59816805468921	-1.79623490123754
H	-4.47465123430221	-1.46392010511383	2.09249546790943
H	-0.29850940923926	-3.37074739581139	-1.13577480292466
N	-3.18286651998194	-0.64955889118919	0.69302651960669
H	-2.47626876187710	0.07892887905061	0.62739233764667
C	-3.90675785393660	0.51422226851990	2.75129181653089
H	-3.59163907339803	1.46586267478676	2.28864164909632
C	-2.86055061978456	0.04199867973186	3.73806283454534
C	-2.51265203667605	0.89486485836671	4.79820948629658
C	-2.22499435298618	-1.20327391713670	3.65302962040407
C	-1.55518452646336	0.52089780167569	5.73762945695117
H	-3.00208719518734	1.86891980248351	4.87838270997725
C	-1.25946467871435	-1.57845466500172	4.59198417416402
H	-2.47370406037506	-1.89798468835644	2.84836157710363
C	-0.91937838962696	-0.71904399512352	5.63406739280333
H	-1.30015800075552	1.20155320792183	6.55138230798413
H	-0.77327651175667	-2.55142880697608	4.50217817742913
H	-0.16476853825275	-1.01267320498787	6.36516509604807
H	-4.83212396867509	0.75640054181607	3.29299628162643
C	-5.55888103108501	-0.04396662874945	0.93244271429646
O	-6.10899037968847	1.00246196857288	1.13228280621250
O	-6.02705549175077	-0.93161416708372	0.06456517252825
C	0.13157828731250	-2.25336878401721	0.65253409427605
H	0.70538627934518	-3.06898575573460	1.12346193207977
H	-0.39556109295530	-1.73504293019908	1.47367466848587
C	1.08232233895408	-1.28532662855378	-0.04879908162954
H	1.32882024867731	-1.68271701328351	-1.05187213226034
H	0.55646094908997	-0.34321744473212	-0.21609344652809
C	2.39276143802813	-0.92383067065034	0.63040277356399
H	2.19158255770634	-0.40780812234012	1.58402986037822
H	2.96657922415529	-1.83822168419287	0.87746049316937
N	3.11530254491766	-0.00519143748483	-0.25603919792556
H	3.92818378819923	0.38828645868827	0.21573038755967
H	3.48421916424420	-0.52477085332913	-1.05544102677115
H	-5.46576441923809	-1.73997902788895	0.05372044673253

76

Reduced TS6$_S$

C	0.49935773604332	4.27367586355981	-3.35438234758479
C	-0.54059377373425	4.83295988262699	-2.62448774425557
C	-1.37655608904548	4.01002378248432	-1.87006540135641
C	-1.18162001050921	2.63357411918986	-1.84394784794009
C	-0.13526746921397	2.05990422967515	-2.57500078848259
C	0.69538158305542	2.89477500107244	-3.31813058410072
F	1.29121845432245	5.04281091696023	-4.07692873875669
F	-0.73792471045894	6.13680208254196	-2.64539223246640
F	-2.34674009861989	4.58730300380590	-1.18026672953343
I	-2.45877888686247	1.52773476834804	-0.60893904020069
F	1.69694057128282	2.40006886310752	-4.03480673604190
C	0.13060359318600	0.57003023441644	-2.61746321508578
O	-0.69751873905368	-0.22562057368779	-3.02912363302009
N	1.36143148805598	0.24146869548707	-2.19052557756992
C	1.93190466779342	-1.08559433047081	-2.33242719969728
C	3.44960998998334	-0.98031943765807	-2.55526486677808
C	4.13758217214321	-0.00119900730298	-1.62745942085263
C	4.36779528229451	1.31491955265924	-2.05270569891142

C	4.98038728186626	2.24564021427472	-1.21179831786526
C	5.37464872085808	1.87186575102613	0.07355660433650
C	5.14676868373554	0.56487372298552	0.51036944490963
C	4.53232626249769	-0.36379758156331	-0.33134678549913
C	1.59668420585428	-2.02501426860816	-1.15346487867277
O	2.45880749715478	-2.63423732483590	-0.54513306046765
N	0.28215394242002	-2.13787039505986	-0.85346259985120
H	1.85416969144601	0.93819464615239	-1.62368029339005
H	1.46054313837759	-1.53611732972299	-3.22075685263847
H	3.87441651836043	-1.98452942774703	-2.43459211597437
H	3.61153779048662	-0.65907322949694	-3.59302823870828
H	4.06003344429375	1.61324447636210	-3.05793337828921
H	5.15467739207522	3.26342699916909	-1.56520099277849
H	5.85979932950478	2.59460952157569	0.73175211310339
H	5.45504987813459	0.26282467038339	1.51294140934987
H	4.34820237835766	-1.38350253751168	0.00898851167811
H	-0.40055864841510	-1.65872675917546	-1.44021038081336
C	-0.11746470846474	-2.89860679036402	0.32338449906298
C	0.26692330427248	-2.26330879952057	1.68620227999015
C	-1.59524621189681	-3.25350378956046	0.25252933725364
O	-1.97153953752665	-4.42200548046438	0.24907323229769
C	-3.90103277935177	-2.47464435699062	0.46404155432761
H	-0.25250004031194	-2.85113143462529	2.46097126928166
H	0.39397806141966	-3.86939015873658	0.27771879594200
H	-3.96620130310879	-3.34292466471201	1.14089040208307
H	1.34138681124777	-2.46050891262231	1.81692077884564
N	-2.47913898544298	-2.23550470581799	0.23903043585426
H	-2.13909104020165	-1.27817637537779	0.24284790948114
C	-4.57959717108852	-1.26602577867513	1.09195621537553
H	-4.67439603969070	-0.46923375672621	0.33734349693782
C	-3.87138880499935	-0.74967933611323	2.32602816616806
C	-3.81641611819098	0.62573104351861	2.57823398185305
C	-3.25321100141725	-1.61853223399135	3.23694267305973
C	-3.14702669137418	1.12706947590302	3.69680044865800
H	-4.31370852108347	1.31774988555298	1.89267677823433
C	-2.59158869968382	-1.12305011060378	4.35908794032360
H	-3.27659373794575	-2.69784907300010	3.06799977350828
C	-2.52985779027029	0.25270895551951	4.58987351745995
H	-3.11194353468946	2.20425557881449	3.86856551136610
H	-2.11351423830233	-1.81612049778867	5.05372710182841
H	-2.00375572124002	0.63996750137982	5.46379592440724
H	-5.61308438063521	-1.55949467362493	1.33269124309544
C	-4.59309268744181	-2.90696584253023	-0.83710007465319
O	-5.48103618840693	-2.28428755361020	-1.34947165851779
O	-4.13473504808555	-4.04119802459556	-1.34924792017038
C	0.03549238360685	-0.76419679754305	1.93425106242037
H	0.33001282657872	-0.57021146809961	2.97956553719027
H	-1.03623029223705	-0.49840219096094	1.88892127759191
C	0.84087752297623	0.13033750132913	0.99741930239202
H	1.85159179618609	-0.29851261807871	0.86023647212454
H	0.37079060649719	0.12697042330808	0.01109186709175
C	1.00281367274912	1.59277299782250	1.37409282859881
H	0.01128629680354	2.07287386693686	1.42792191388710
H	1.45879503259453	1.68764404339613	2.37879013658060
N	1.78394910194990	2.24339669541066	0.31663065123597
H	1.79563925785493	3.25397395180504	0.44611781008129
H	2.75985797264873	1.94674241089914	0.38937623367559
H	-3.41648364196855	-4.41385870188526	-0.78760909201897

76

Reduced Intermedia H_s

C	0.06773301989440	4.21763987962751	-1.79421190284346
C	-1.26430072359167	4.31242977526831	-1.41537592709038
C	-2.10808911859742	3.21339251752294	-1.57800914006420
C	-1.63514639822282	2.02781707882641	-2.12957805557810
C	-0.29226943620196	1.93112174433196	-2.51798844950293
C	0.53857038809581	3.03044623329080	-2.34580874874773
F	0.89123544988355	5.23350376142076	-1.60091942448123
F	-1.72542565984631	5.43052721029291	-0.88618709698207
F	-3.36285332456306	3.33562336471307	-1.16739006876567
I	-2.96955016320948	0.41324669862482	-2.23692670518251
F	1.82253029750826	2.96783720862598	-2.66995158670753
C	0.28957041538087	0.62835267168928	-3.03069417564601
O	-0.11688670530249	0.08714546965536	-4.03644173925307
N	1.24029475934838	0.13187361165272	-2.20467402163811
C	1.91535938104585	-1.12797177954212	-2.44121346752425

C	3.42552701513744	-0.95041629704791	-2.66530425624840
C	4.08993946859604	-0.03649336182077	-1.65998820034689
C	4.34922845653838	1.29750508294615	-1.99882879725932
C	4.94748744544483	2.16895834951841	-1.08758824239979
C	5.29628904500828	1.71549652551873	0.18562641509734
C	5.03724629613294	0.38878421622216	0.53875582350320
C	4.43941474985527	-0.48066022252066	-0.37569065680948
C	1.62683016758800	-2.14753189517835	-1.31618876775758
O	2.48284906055171	-2.90598827639073	-0.89814369358387
N	0.35547397281308	-2.16060850530913	-0.84891517031788
H	1.53884458844037	0.72662960853252	-1.42349303825825
H	1.46787886886941	-1.54816566661702	-3.35583443283263
H	3.88318613144931	-1.94776102576671	-2.64182181516975
H	3.56357254053990	-0.53255884601503	-3.67163016504099
H	4.07642421986234	1.65776282300742	-2.99302086359559
H	5.13712069895209	3.20505855203800	-1.37399387982029
H	5.76894636976795	2.39162425820633	0.90008974784966
H	5.31132551431627	0.02445897790148	1.53052180881525
H	4.24364065946179	-1.51947152418334	-0.10363318944248
H	-0.29792383128963	-1.47793656802675	-1.21811424083216
C	0.00891921606553	-2.93712367975520	0.33544754770660
C	0.58274593891991	-2.38562506616700	1.66140837855668
C	-1.49600518908994	-3.15608940014018	0.39004300909033
O	-1.97386801020392	-4.28667315873792	0.42109282657758
C	-3.72871025949004	-2.17835494723154	0.57862510033307
H	0.23738260883650	-3.06139314798448	2.45964169687712
H	0.43024594976600	-3.94151267654211	0.20055855195912
H	-3.89831999173071	-3.00258656021246	1.29086425089261
H	1.67352340782667	-2.51837157258721	1.58961633996401
N	-2.28248312825733	-2.05989924669689	0.41229614825691
H	-1.87511956059959	-1.12916281927648	0.44554435306643
C	-4.35928268309120	-0.88512316781509	1.09618363123995
H	-4.47383955151889	-0.18245930461600	0.26180921727607
C	-3.56930258401293	-0.23772135669560	2.20729587753616
C	-3.02731506510554	1.04099008655300	2.03313314096459
C	-3.34597556381863	-0.90617882724912	3.41927562495511
C	-2.28841308148122	1.64553395012454	3.05404520843972
H	-3.20027007695258	1.57391318593828	1.09364555696961
C	-2.60282327042541	-0.30784589353061	4.43487628517101
H	-3.76404318283427	-1.90501360112360	3.56971688144785
C	-2.07193452030747	0.97224268444766	4.25495548334901
H	-1.88850318857775	2.65101080918288	2.91024667880514
H	-2.44032612250507	-0.83964460640509	5.37392014630045
H	-1.49535302276865	1.44341015184813	5.05257368746879
H	-5.37552538078020	-1.13378073898987	1.43683805639466
C	-4.34841998612779	-2.60074650343422	-0.76813116653898
O	-4.99722130067494	-1.85470301990674	-1.45588007317361
O	-4.08699595595156	-3.84136166798476	-1.13772664109034
C	0.30030206370159	-0.92774988042445	2.05089847360476
H	0.82670718624348	-0.74475292846043	3.00237569003838
H	-0.76697740012146	-0.76403757833471	2.27448489010404
C	0.79950318663158	0.06396030261041	1.00286067979679
H	1.73802033605153	-0.33450415070896	0.57500400730259
H	0.09049712580668	0.13457283851464	0.16256145374699
C	1.10132508484661	1.49120908841982	1.43271595152091
H	0.16629921742803	2.02930283183952	1.65039727827491
H	1.69685605991088	1.49068033291236	2.36594156730290
N	1.78050323468131	2.14930029077642	0.31383614501183
H	1.77991979335956	3.16282648183468	0.42100551737768
H	2.76502398256133	1.87603186549567	0.30183442770558
H	-3.45005012894510	-4.26206678789873	-0.51141023458003

76

Reduced $TS7_s$

C	0.70001462214557	4.26021309200520	-2.62401774990068
C	-0.56258919792361	4.63351066594119	-2.18884627626915
C	-1.54007738685581	3.65872826437664	-1.99752546750811
C	-1.27258586106696	2.31468088459453	-2.23915130639053
C	0.00727603382094	1.92747429819853	-2.67480900971603
C	0.97194380175194	2.91646032393936	-2.86217852393634
F	1.64036954510057	5.17248497317405	-2.79632954150183
F	-0.83505627783906	5.90252725358296	-1.95269751647464
F	-2.72504804404399	4.06665515803198	-1.56898862190977
I	-2.86271932292952	0.98694046753771	-1.88106210015052
F	2.19984380628921	2.61765297921312	-3.26335252359182
C	0.36516171559369	0.47295762876414	-2.90290616909444

O	-0.38892618684523	-0.29229470461066	-3.48060348721227
N	1.54328670694380	0.10459746532391	-2.36385067051875
C	2.01597705351412	-1.26722478369180	-2.40081242735412
C	3.54311908503883	-1.31992996467902	-2.55573319292443
C	4.29039072276992	-0.33742942227528	-1.67946905554651
C	4.69030451868210	0.89756005040988	-2.20672573241755
C	5.37183755786575	1.82860848855802	-1.42187383373475
C	5.66746280702954	1.53538964800231	-0.08980752346274
C	5.26933341843249	0.30987093515216	0.44971342630710
C	4.58367702010320	-0.61794697347068	-0.33672822539886
C	1.54183999116290	-2.07734181516827	-1.16984714926533
O	2.30886410739680	-2.72330533443788	-0.47804367878023
N	0.21421040391930	-2.01710377692543	-0.91134395274987
H	2.01434368275866	0.78372024130236	-1.75173138608266
H	1.54368136552917	-1.73941893491301	-3.27616660043042
H	3.86421522802764	-2.34631994837179	-2.33764131905873
H	3.77763948724027	-1.10362968815102	-3.60663180783815
H	4.46219375303954	1.13173837332261	-3.24904090810590
H	5.67621534607618	2.78317956312397	-1.85466652126388
H	6.20719240564272	2.25735402871559	0.52542126374755
H	5.49954408094072	0.07069735975563	1.48954464634923
H	4.26886621635563	-1.57478107723173	0.08209585145804
H	-0.39222250728991	-1.50028279198252	-1.54527605962785
C	-0.32351950625556	-2.57982901900757	0.32222573204282
C	0.06965079500985	-1.84763286625298	1.62964423699508
C	-1.81625290056873	-2.79783055713921	0.10760865480972
O	-2.24139805112509	-3.83142977630466	-0.40473795460250
C	-4.08664299063397	-1.93008029982585	0.44058984566332
H	-0.51955804770154	-2.30832850185170	2.43949968527174
H	0.08371339324188	-3.59756547289378	0.41053010094614
H	-4.32908110106220	-2.94702886286928	0.79341027056201
H	1.11655601716607	-2.13235049808242	1.81195349408742
N	-2.63557492885856	-1.79826433037939	0.47100006352150
H	-2.24434445065182	-0.95964331835547	0.88692117417719
C	-4.73839978971210	-0.87388787518452	1.33026808915036
H	-4.67374010677036	0.10129476032494	0.82622786135007
C	-4.09620534400843	-0.79837609415352	2.69521897734952
C	-3.50253883179926	0.38971632947832	3.13273707084690
C	-4.03921784522103	-1.92520499022347	3.52747761314604
C	-2.86229200832912	0.45506105753681	4.37311800342010
H	-3.54025870455075	1.27391116964918	2.49074573519403
C	-3.39977091248833	-1.86440902247847	4.76361482495152
H	-4.49907753608819	-2.86186617804575	3.20172363354066
C	-2.80631401146143	-0.67275622687351	5.18936873923292
H	-2.40448287406378	1.39079243311548	4.69856376010680
H	-3.36380145201731	-2.75035408173045	5.39984870634776
H	-2.30468060856359	-0.62616284673999	6.15721474348924
H	-5.80874495333946	-1.11560923706856	1.40699757432805
C	-4.58615310885585	-1.82394555898292	-1.01144129673368
O	-5.24288113577383	-0.89241896749805	-1.40471320683240
O	-4.22036963552644	-2.80961473785286	-1.80583208762993
C	0.00608811164497	-0.31358843241699	1.75433078592077
H	0.41705727058950	-0.06986782655712	2.74823897030911
H	-1.02456807927206	0.08135044689670	1.78183543304421
C	0.81178847460877	0.39727238884979	0.67500625836002
H	1.74819841277239	-0.16785965610749	0.51111819154204
H	0.25578128409423	0.35432934795715	-0.26703776966128
C	1.20501059876690	1.85106680923701	0.89335922527839
H	0.32360108329147	2.50318276095105	0.77640864124896
H	1.58251928285008	2.00126997813717	1.92300631936688
N	2.18711369876803	2.20156749366325	-0.13741345419623
H	2.34121341472188	3.20825164875527	-0.17692110904149
H	3.09278455815398	1.79113434947564	0.09965699368373
H	-3.59078717935835	-3.42028866626855	-1.34028938023252

76

Reduced Intermedia I_s

C	1.48102512682330	3.57724947280891	-4.48038661658348
C	0.35515346210390	4.28768415887997	-4.08486116981008
C	-0.68377721250553	3.62861521041820	-3.42933528071624
C	-0.61196720145677	2.26426328143781	-3.16678442579551
C	0.53024862794070	1.54730980811685	-3.54704607245906
C	1.55952296584060	2.21641853493852	-4.20137635549941
F	2.46777485608676	4.19398395478783	-5.10618922490869
F	0.26930582274883	5.58178665417361	-4.33339100551157
F	-1.73328254155005	4.34922858769496	-3.06558804686200

I	-2.24494312197685	1.38098548903615	-2.19099824503866
F	2.66206001531904	1.57388236077040	-4.56915792850645
C	0.72142538530824	0.08701954829026	-3.19352920296212
O	-0.04401317566058	-0.78753836340660	-3.55875683424391
N	1.80081826513259	-0.11686701676587	-2.41396902262062
C	2.18417318810847	-1.42546760850119	-1.91807466420889
C	3.71466212419306	-1.54592270780141	-1.84300595242584
C	4.39270021164716	-0.31892336142888	-1.27173870791101
C	4.87925918649586	0.67168246193691	-2.13608289888820
C	5.48442918827966	1.82638713462693	-1.63805998984890
C	5.61357851298791	2.00851114769323	-0.26031645537082
C	5.12847615353664	1.03104882774582	0.61125780875558
C	4.52189948790748	-0.12247539513156	0.11070215055549
C	1.52122908551938	-1.74771317248404	-0.55725951873118
O	2.16776565191847	-2.12211410443349	0.40712888119278
N	0.18332618935112	-1.57304678200535	-0.52028315646772
H	2.24564657634330	0.71742446231731	-2.01666732630834
H	1.79057490607924	-2.16162561793013	-2.63636126416603
H	3.95138877029394	-2.43096747649625	-1.23939970293953
H	4.08697316616672	-1.71405735460894	-2.86245762991215
H	4.77758237894035	0.53593384216527	-3.21534147569247
H	5.85986936762102	2.58284948468697	-2.32943075482968
H	6.09214273858762	2.90703844449814	0.13304458311843
H	5.22884341836367	1.16356541437401	1.69011090630899
H	4.13732909383396	-0.88551688337202	0.78882334713529
H	-0.33152428407882	-1.40236816707834	-1.38436405889199
C	-0.56535027012022	-1.63058315695050	0.72119105874232
C	-0.11545791745436	-0.61264642706347	1.78851018218761
C	-2.04565761176316	-1.56820254687373	0.35523653256689
O	-2.43736055589880	-1.94463354287934	-0.75048876807568
C	-4.34680491894298	-1.26371748238036	1.16294162554867
H	-0.68213442655357	-0.81995118485139	2.71087462868489
H	-0.43078810120607	-2.63368886136555	1.16936130349147
H	-4.54950531157825	-2.25459579823840	0.72020710876932
H	0.92465724035275	-0.86794275609741	2.03413025399380
N	-2.89900896578082	-1.13045185138810	1.29365255457422
H	-2.53866249020993	-0.80402918872215	2.18370234975263
C	-5.01610142447664	-1.14239172295967	2.52976252545082
H	-4.89762952440139	-0.10880450011161	2.88659027687315
C	-4.44959508389349	-2.12106572531088	3.52965603409449
C	-3.64668807565095	-1.68028138829064	4.58745440172832
C	-4.68345325636333	-3.49558879561925	3.39058611530760
C	-3.08392969724769	-2.59056934987813	5.48535231477978
H	-3.46794726308578	-0.60916366895484	4.71551331165157
C	-4.12525856928043	-4.40620207527753	4.28531907102475
H	-5.31436339218763	-3.85481120132054	2.57319636276575
C	-3.32110335120052	-3.95567483537824	5.33507589355574
H	-2.46258238104508	-2.22919018735227	6.30645145592696
H	-4.31999308190490	-5.47327937199322	4.16554391305578
H	-2.88443956726666	-4.66905104558886	6.03568184543075
H	-6.09347532175550	-1.30427347617924	2.38579986409887
C	-4.89501282889067	-0.20979508559693	0.18517927367536
O	-5.65827011136708	0.65505962389546	0.50916496138160
O	-4.45222828171681	-0.33184674121428	-1.06344044315440
C	-0.18998987804347	0.87646317554885	1.42566615102617
H	0.04554177685532	1.45436030709994	2.33451668724601
H	-1.21658739087179	1.15980294627371	1.13218597657275
C	0.78986631333886	1.23466375048326	0.31425052214802
H	1.75223477515144	0.72376319130049	0.50986665118808
H	0.41528651043324	0.82839556785152	-0.62869331946695
C	1.09563928083861	2.70034597557106	0.05820723810216
H	0.19781801437677	3.20403386369309	-0.33864909285106
H	1.36158419909904	3.21593701556334	1.00150697581337
N	2.15401788660795	2.75886393900117	-0.95418783847811
H	2.27014194269908	3.70732334583066	-1.30801585361106
H	3.04865768954296	2.50824648572485	-0.52774664739872
H	-3.80034602960270	-1.08209898926013	-1.13168433289724

76

Reduced TS8$_S$

C	1.47012713020062	3.58048067465459	-4.51938451205404
C	0.34062301436839	4.29069846148161	-4.13265831880997
C	-0.69207346519349	3.63702614408882	-3.46152800683045
C	-0.60811920990494	2.27870396640578	-3.17316892090310
C	0.53499183284725	1.56153546413292	-3.54810145170878
C	1.55896875882185	2.22500568719282	-4.21631841946732

F	2.45114193209274	4.19255027068906	-5.15842512622083
F	0.24582794090438	5.57940904768298	-4.40404435318735
F	-1.74418729802032	4.35695657479233	-3.10478824611794
I	-2.22076690968349	1.39600005131320	-2.16583166726761
F	2.66240150488511	1.58197240377062	-4.57952890302414
C	0.72379061340888	0.10413640604421	-3.18281268433707
O	-0.04108146327535	-0.77103533059097	-3.54775064712420
N	1.80080343083774	-0.09692739517553	-2.40033004317915
C	2.18079062061427	-1.40807554413317	-1.90705447044357
C	3.71085525977758	-1.53777389606783	-1.83811218911139
C	4.40045118864113	-0.31125482771343	-1.28012138562944
C	4.88775355548067	0.66983490288719	-2.15473344800120
C	5.50174589601281	1.82474747293734	-1.66885800669651
C	5.64171680535709	2.01525289305215	-0.29322536433081
C	5.15667136346941	1.04677774098494	0.58817669275148
C	4.53914674021968	-0.10600710897500	0.09957056373761
C	1.51874221223862	-1.72963411221419	-0.54524411262239
O	2.16573658386955	-2.09159969161457	0.42251528544227
N	0.17864786491437	-1.56002808498448	-0.50838041348664
H	2.24758091607098	0.73991669938101	-2.00468202196515
H	1.78010340928761	-2.14103908609599	-2.62465024942989
H	3.94492393154927	-2.41999325551264	-1.22922957656199
H	4.07675734251397	-1.71647230457036	-2.85810298509837
H	4.77819474802599	0.52693253939320	-3.23232199891837
H	5.87652360200082	2.57446396076317	-2.36796400431726
H	6.12851206638987	2.91348288713015	0.09068988777666
H	5.26448152467999	1.18624045339342	1.66550986347464
H	4.15276072634935	-0.86118470804295	0.78539792395249
H	-0.34021550426923	-1.40723215468337	-1.37361504276756
C	-0.56588920769833	-1.63033722970544	0.73541553660264
C	-0.12290279368711	-0.62030708930806	1.81411896353441
C	-2.04693364806722	-1.56914842873545	0.36906024195673
O	-2.43620497391215	-1.94812305531886	-0.73685293689211
C	-4.35104974975534	-1.26368941701618	1.16599777759933
H	-0.71257353197322	-0.82492457968166	2.72200640452486
H	-0.42926419302809	-2.63749966335391	1.17422033467108
H	-4.55033531998551	-2.25306994982402	0.71850054620175
H	0.90788669822116	-0.88717514568610	2.08473826084536
N	-2.90390593126610	-1.12967168940629	1.30326826521486
H	-2.54850264332143	-0.79621505825431	2.19246997594635
C	-5.02692097272851	-1.14857543737610	2.53001269052467
H	-4.90911132736755	-0.11704421988543	2.89305009709373
C	-4.46705738965932	-2.13349844511903	3.52755179523573
C	-3.66985876212034	-1.69979890042533	4.59254044493418
C	-4.70229822787106	-3.50687297644029	3.37964099826833
C	-3.11412399981019	-2.61583888337510	5.48894138517833
H	-3.49019193585169	-0.62967903482821	4.72749347368430
C	-4.15096598021000	-4.42325516094563	4.27273854342722
H	-5.32892432327869	-3.86062811749759	2.55659510705927
C	-3.35258934550499	-3.97973027603551	5.32984902757379
H	-2.49733328396661	-2.25998953601848	6.31588415375039
H	-4.34668466063427	-5.48933396221675	4.14600643919408
H	-2.92144041231252	-4.69758753328910	6.02928750722427
H	-6.10375629412675	-1.30825906162760	2.37962398152346
C	-4.89496158534801	-0.20686146710045	0.18935117206006
O	-5.66312979811283	0.65442621170826	0.51118141000001
O	-4.44086249693650	-0.32129054844224	-1.05600988979399
C	-0.17765182766833	0.86907081912150	1.44199318412983
H	-0.14828282261027	1.46444126209320	2.36879311088059
H	-1.13524877149324	1.10919813951204	0.94603541187286
C	0.98795662141494	1.24720809959865	0.53512443905962
H	1.93727185895056	0.96752559307290	1.02723888872866
H	0.91822001455586	0.63916437219993	-0.36676887212094
C	1.09374326710551	2.67864726731848	0.03814892173280
H	0.15424018981098	2.96267433168950	-0.46593044772052
H	1.23148853099190	3.38467773666134	0.87949770429061
N	2.17984970035482	2.72051453988777	-0.94612264928169
H	2.24010756568466	3.63882196276237	-1.38420363339355
H	3.07601198917453	2.57217132917675	-0.47721393756291
H	-3.79214889144190	-1.07400399968656	-1.12416747528106

76

Reduced Intermedia J_s

C	1.19281068839883	3.86559312512712	-4.25828059866393
C	-0.06191376131521	4.41407549955780	-4.03243361410161
C	-1.08266839285631	3.61750121980654	-3.51495699319072

C	-0.86482150909127	2.27492207487744	-3.22823861928868
C	0.40434898953642	1.71422757364578	-3.44760745439518
C	1.41652422573658	2.52372225207607	-3.95671361011001
F	2.16192161701492	4.61458234149913	-4.75423677303980
F	-0.28517425237889	5.68605937121171	-4.30266207282617
F	-2.25560018373887	4.19225511872771	-3.30087036101474
I	-2.47195484212479	1.20733193872862	-2.40126032946022
F	2.63470824881309	2.04472603598386	-4.17429163918599
C	0.68712309550015	0.25517916376424	-3.15896542941581
O	-0.04530730341079	-0.62388584692914	-3.58566429089383
N	1.77173424367969	0.03452655841462	-2.39669931734386
C	2.16254128156053	-1.31531735037262	-2.02893387980819
C	3.68887565340243	-1.47188660593800	-1.98262356956406
C	4.42084013852942	-0.36617180470504	-1.25307280535507
C	4.99400796767476	0.68483745857902	-1.98109529007174
C	5.67429866390546	1.71874631169205	-1.33659207048030
C	5.77907552935649	1.72491085755277	0.05490918123071
C	5.20733068727470	0.68566634824706	0.79200287453186
C	4.53935262386604	-0.35452162259872	0.14418912142615
C	1.49300455492813	-1.76228031460817	-0.71112874502188
O	2.11534783730726	-2.28179940907797	0.19886639835877
N	0.16602396773820	-1.50768916624722	-0.63573524256527
H	2.22382352657338	0.85430414896820	-1.94544156417904
H	1.76095323383134	-1.98316175858903	-2.80752156669411
H	3.90369085484125	-2.44332498772267	-1.51843128865377
H	4.04803035588716	-1.50389276604465	-3.02020843002586
H	4.90656512968740	0.69005962068270	-3.07025414902002
H	6.13142708916745	2.51829576040152	-1.92199917757296
H	6.31294358196682	2.53020854533831	0.56257772077761
H	5.29433367267759	0.67648795088262	1.88019530124092
H	4.10589197642795	-1.17481522825994	0.71791716761772
H	-0.35609829604931	-1.30411956680001	-1.48805202549283
C	-0.56142982701569	-1.71660848124493	0.60001008196767
C	-0.12824367105805	-0.74620555486651	1.71113810959897
C	-2.04762998269227	-1.68796018167054	0.25221218612313
O	-2.44153921451656	-2.15496090225241	-0.81842250559948
C	-4.34579616359795	-1.35629681036664	1.06316902750758
H	-0.73026473311405	-0.94912992008973	2.61076096598376
H	-0.37935062976854	-2.74767325673727	0.95739221511339
H	-4.53153988893989	-2.39294482231298	0.73075764596154
H	0.89843885709103	-1.02168301973377	1.99214579254702
N	-2.89904374468815	-1.17951349120037	1.15499058359650
H	-2.53518389006718	-0.77134893003583	2.00918824798420
C	-4.99508858841917	-1.10877584734362	2.42337925112513
H	-4.90375198143793	-0.03959173345812	2.66440582146822
C	-4.37894836720192	-1.95602066303372	3.50966397088990
C	-3.53176281987669	-1.38690993801322	4.46694981278498
C	-4.60686143383791	-3.33785496238040	3.55012336960363
C	-2.91917497756269	-2.17885452349247	5.44106266656580
H	-3.35702651985576	-0.30729506168423	4.45522652329865
C	-3.99991538511944	-4.13048355054426	4.52208764874609
H	-5.27164057809400	-3.79570474153878	2.81284228904561
C	-3.15131899962379	-3.55288089169199	5.46954057185118
H	-2.26344986185529	-1.71807349337601	6.18175384940479
H	-4.19089688587727	-5.20472612293521	4.54292235523997
H	-2.67621585151293	-4.17396859534532	6.23042488388706
H	-6.06868734616831	-1.31835467632623	2.32027947187349
C	-4.94488254589126	-0.42815892611744	-0.00842478615870
O	-5.75292009263453	0.42221461119029	0.23692605924765
O	-4.49958771634405	-0.64758248653006	-1.24173268597853
C	-0.17248741262243	0.73218808856894	1.28827410301584
H	-0.50533645024646	1.36327937078734	2.12662491055688
H	-0.92363720090897	0.86286046410341	0.48860944837762
C	1.18857677841071	1.21354173294898	0.78115314140403
H	1.87798990886311	1.35385204792221	1.63173467819449
H	1.63985886145248	0.42245317767563	0.17096888843496
C	1.16217569168642	2.46713327960458	-0.08357542685228
H	0.29660072515375	2.41591478224093	-0.76689956173432
H	1.01692206893164	3.36666504635430	0.54258946752475
N	2.37788793654039	2.51206400549821	-0.89823297412760
H	2.46291032379343	3.40268626241171	-1.38699298257361
H	3.20860015878521	2.42978550875010	-0.30745836572973
H	-3.81017073595338	-1.37119817576414	-1.24338704879697

76

Reduced $TS9_s$

C	1.12655856854750	3.65070616259938	-4.95617286574445
C	0.05854289112916	4.47513496482401	-4.62514626262434
C	-0.97684443036491	3.98346633122856	-3.82983761000487
C	-0.95307185724115	2.67145660596193	-3.37129211461971
C	0.12052079783683	1.83318212910135	-3.69915394558855
C	1.14857623243449	2.33868173479426	-4.48721443578930
F	2.11001152449188	4.11011969535251	-5.70914859900044
F	0.02948109418132	5.72256481040736	-5.05572692480583
F	-1.96545375020837	4.80736376759607	-3.52389048609178
I	-2.48869637543478	2.03896730531632	-2.10096929162084
F	2.18762370723906	1.58225714133255	-4.81689890689968
C	0.17161287219147	0.38331215762010	-3.25224875844749
O	-0.68876371328158	-0.40629962847035	-3.59460889047152
N	1.22271293891218	0.09471693246354	-2.46324060123150
C	1.49658374783803	-1.26032586087605	-2.02662333821809
C	2.95741706614473	-1.65835932119065	-2.29138919003031
C	3.96440494328128	-0.60587944783118	-1.88436870458990
C	4.51133476297290	0.24692222806350	-2.85129292734990
C	5.41646032161372	1.24796502351192	-2.49766527486637
C	5.78906776468503	1.41309822579010	-1.16278213481586
C	5.24875952670047	0.57034206029910	-0.18889721446724
C	4.34543439320996	-0.43186902997780	-0.54621392984451
C	1.09921875324625	-1.49624048719045	-0.55566161791305
O	1.80138714460768	-2.13122177899127	0.21398713643158
N	-0.08770733214026	-0.97151921228175	-0.18052252570426
H	1.85039137137382	0.87804173544213	-2.18234727333577
H	0.83570233631553	-1.91562477767875	-2.61605160556895
H	3.14553000410928	-2.59800571606213	-1.75614296773756
H	3.05921607642456	-1.85044523744834	-3.36803480444386
H	4.21894599407488	0.12549628862588	-3.89663032507410
H	5.83431069355625	1.89840811733653	-3.26821033372377
H	6.50137769915241	2.19121390132852	-0.88265956073327
H	5.53954860374950	0.68771789210114	0.85686856191400
H	3.92340071962550	-1.09218402014101	0.21329426977217
H	-0.68349437576863	-0.47836437399455	-0.84269109474026
C	-0.50382942579586	-1.01593970121588	1.20222736458641
C	0.27193972325674	-0.01234723032059	2.08825862821567
C	-1.99746063187163	-0.72328444552949	1.25752796494527
O	-2.61748353962414	-0.32552470131696	0.26987369904574
C	-4.02994287250889	-0.71672006453045	2.64089012857642
H	-0.17476512795392	-0.01370897174875	3.09302045226295
H	-0.33366086549477	-2.03269639230375	1.59200758886764
H	-4.52544875050187	-1.16867187019258	1.76357992545930
H	1.29710970922033	-0.39604753395216	2.20095475778403
N	-2.59030725780696	-0.86970203090809	2.45279811935182
H	-2.04722681908385	-1.22612665709227	3.23352982672880
C	-4.47883205649069	-1.41569596931102	3.92112027025796
H	-4.03812956747464	-0.89085771316499	4.78167437577339
C	-4.10603306710992	-2.87807988310169	3.93784896962512
C	-3.11237248710529	-3.35673954699023	4.79810820884189
C	-4.72975770611725	-3.77918433127501	3.06436358351026
C	-2.74480878864982	-4.70429299134732	4.78624233043751
H	-2.62668264203644	-2.66723183376174	5.49390419953354
C	-4.36641739744064	-5.12396351286900	3.05018398384109
H	-5.51373324794591	-3.42208769944621	2.39126450084955
C	-3.37031294472531	-5.59054905540579	3.91129779785811
H	-1.96940913664569	-5.06108612906004	5.46619578066962
H	-4.86465097002496	-5.81323765412409	2.36640177851806
H	-3.08613320304882	-6.64390399105954	3.90086462699758
H	-5.56710768214309	-1.28674511054181	4.00029828131944
C	-4.42523513153672	0.77175231566261	2.65885344230068
O	-4.97324466687120	1.29051912099465	3.59057214190533
O	-4.12400708437406	1.44224838548223	1.55419180472444
C	0.29779189619750	1.40167156676621	1.48834569279366
H	0.23901525118876	2.15508617552726	2.28739479615625
H	-0.60256452568997	1.55224990799077	0.86444105325338
C	1.54830268761754	1.64101355472455	0.63643609331062
H	2.40812400858738	1.87406329158748	1.28841151825411
H	1.82540171057735	0.71044732973568	0.12220961561929
C	1.39627056735371	2.71831355554682	-0.42797371212366
H	0.41137201098961	2.59748209592879	-0.91207777527667
H	1.39839825308215	3.72126992802604	0.03678890757087
N	2.42707732660100	2.54708596717728	-1.45164050186808
H	2.47171292828906	3.35551662569456	-2.07082903254146

H	3.35159860859125	2.45533458641273	-1.02568807403149
H	-3.69465780068604	0.84557429434932	0.89571143407508

76

Reduced Intermedia K_s

C	0.98465237484875	3.53224524704554	-5.15441162195400
C	-0.08635585367612	4.31214898118570	-4.73064251156052
C	-1.04021007318298	3.77436418404654	-3.86656565490566
C	-0.92967664157860	2.45924562736302	-3.42873201103871
C	0.14362676646467	1.67004083932946	-3.85266707409235
C	1.09034343292011	2.21706615503293	-4.70730031074188
F	1.88941420594044	4.04076846318852	-5.97085892662223
F	-0.19609788705829	5.56036330089248	-5.14750751527560
F	-2.03746084236536	4.55177931595049	-3.47767388124708
I	-2.33453550363757	1.70241963630160	-2.07746387926055
F	2.12897888582786	1.49352214531418	-5.10829213598400
C	0.29379818783489	0.22017100347138	-3.42443136564377
O	-0.30362002662075	-0.66464636624354	-4.00269811133894
N	1.10853547039771	0.06563573647987	-2.36122456327173
C	1.45636920357957	-1.25156376528416	-1.86945624658427
C	2.92535911063703	-1.60904427301158	-2.15348256621956
C	3.89351658400800	-0.50746695314861	-1.79020366541481
C	4.38544186903004	0.34897902577032	-2.78338427076604
C	5.24992828220443	1.39667300613583	-2.46484435327300
C	5.63230209841452	1.60849754191200	-1.13900403362742
C	5.14433837223979	0.76418437654761	-0.13927375427050
C	4.28467081833975	-0.28694036633427	-0.46220920549437
C	1.10049777232104	-1.43725431492005	-0.38396081105446
O	1.78424345926747	-2.11957676776314	0.36117509303469
N	-0.03933622252306	-0.83866394767541	0.02831347769752
H	1.63705520431839	0.90839268254762	-2.03888231869446
H	0.81430549275012	-1.96301815759231	-2.41319630662893
H	3.15697391835256	-2.52804142058504	-1.59953549021743
H	3.01099001880294	-1.82302319422877	-3.22770620145060
H	4.08359929960092	0.19193587865635	-3.82133492972846
H	5.62689108738053	2.04824853978399	-3.25535983715046
H	6.31210637035074	2.42409662692150	-0.88606620588647
H	5.44272355832438	0.91890668651793	0.89948011367454
H	3.90465549801721	-0.94888997930102	0.31845033898525
H	-0.56739225653703	-0.23679523097005	-0.59994592206658
C	-0.42208851838748	-0.87261528301786	1.42047598318931
C	0.38489144335841	0.12214209685109	2.28981763820823
C	-1.90573594128663	-0.55039857164655	1.52173763055340
O	-2.51651123416239	0.00140035699674	0.60398540696840
C	-3.92076070701745	-0.60001283836555	2.91952519454486
H	-0.02214279264345	0.09723115074865	3.31136022454561
H	-0.25005170806116	-1.89050168488941	1.80342153884872
H	-4.44319511110316	-0.86241631591837	1.98339077154544
H	1.41603819997596	-0.25867816690343	2.35126268799130
N	-2.49719054524169	-0.82751188789923	2.69385948051171
H	-1.96870109441366	-1.30419443961271	3.41866444093980
C	-4.42935057964474	-1.46397498748675	4.06907029734372
H	-3.94502362065956	-1.13446171118222	5.00032233967901
C	-4.18604706265193	-2.93434521017031	3.82968156144096
C	-3.25622902916591	-3.64333992514281	4.59696009870349
C	-4.86894132959400	-3.60903599408956	2.80869462722330
C	-3.00913694359871	-4.99589902754466	4.35075068056168
H	-2.72553465242209	-3.13173359751753	5.40427176201636
C	-4.62542599063407	-4.95803170173394	2.56031452488101
H	-5.60428180240205	-3.07077314012681	2.20466154943934
C	-3.69234077325922	-5.65577361487951	3.33097592089741
H	-2.28221109034244	-5.53445201947935	4.96096005311101
H	-5.16799988692797	-5.46950341853452	1.76346254243469
H	-3.50196198863287	-6.71241641614293	3.13691648439886
H	-5.50244342239885	-1.25732070411710	4.18483346799338
C	-4.19137112927924	0.89229362781078	3.18217816638195
O	-4.67151323203557	1.30034360923814	4.20207196449096
O	-3.85474987701024	1.70573170260472	2.18752447872594
C	0.37995975544992	1.54889054516430	1.72101892754742
H	0.41252282187063	2.27864841930232	2.54275847411500
H	-0.57503703054164	1.73057739293917	1.19520767219366
C	1.54794665133615	1.79151724027498	0.75636884519154
H	2.44796003781492	2.09315014006668	1.31933429798596
H	1.82292350596875	0.84593591771394	0.26837601067293
C	1.26546355963928	2.80231728320105	-0.34727203776474
H	0.24718088242298	2.62968953970536	-0.73555978528063

H	1.27888888688480	3.83047608612744	0.05851165026888
N	2.20406262474662	2.59678316442112	-1.44966874213473
H	2.14571135761859	3.35218697177730	-2.13191454384038
H	3.16852704313145	2.58010776453182	-1.11179696051165
H	-3.49383471169438	1.19755038358860	1.42579933205970

76

Reduced TS10$_S$

C	2.49069717579718	1.75559726173154	-5.56990513306119
C	1.40240859018233	2.57606620005195	-5.29727231545115
C	0.43977143875260	2.16945369458979	-4.37330646726879
C	0.56599121985451	0.95473397965567	-3.71058731601125
C	1.65801323843319	0.12294720387134	-3.98131104812582
C	2.60481989562676	0.53322077855400	-4.91071572058029
F	3.40642297309114	2.13762450124474	-6.44137755334027
F	1.28129258348934	3.73823762736423	-5.91281594623657
F	-0.58178634027705	2.98019426459125	-4.14412677991006
I	-0.88070639283820	0.41420659495489	-2.30077414150309
F	3.66318526889545	-0.22511264985251	-5.17099116034472
C	1.87214518622077	-1.21799676323471	-3.30255049268114
O	1.70372764892239	-2.26259674116565	-3.89455727645521
N	2.25869597689158	-1.10018344716923	-2.01114019158985
C	2.69933356167534	-2.25544641599688	-1.25279973329718
C	4.23337105966263	-2.38549910014580	-1.25659946139212
C	4.94298043844782	-1.08115893728212	-0.97959129075445
C	5.35005534534116	-0.26678768185215	-2.04565473528243
C	5.98364585670456	0.95480405802537	-1.81438717291492
C	6.21206589361748	1.38686290034908	-0.50633320692000
C	5.80293826746050	0.58822247984992	0.56363787623905
C	5.17535992193681	-0.63665732325765	0.32895949363296
C	2.12269435902584	-2.28647116069847	0.17722587381198
O	2.80265418597575	-2.61190277215228	1.13524426068978
N	0.80944414845398	-1.98043578533732	0.28802474179257
H	2.50371752017081	-0.15023288943199	-1.69661185318277
H	2.27696161033415	-3.13310310928662	-1.76718769786141
H	4.51336999666918	-3.14587017367556	-0.51665553220510
H	4.52482318288219	-2.74736781037440	-2.25261775127937
H	5.16973400961180	-0.59797181185192	-3.07173948511685
H	6.30072850613982	1.56982356524016	-2.65861229382200
H	6.71052344929881	2.33998304195471	-0.32133084125820
H	5.98076294547233	0.91703239496852	1.58923742030705
H	4.85339547768684	-1.26243922422399	1.16349959765473
H	0.30370968874027	-1.64661790557740	-0.53000249533338
C	0.18280340755750	-1.78321896371439	1.58003710715713
C	0.77741258122014	-0.63238961426280	2.43622096550882
C	-1.29368687947757	-1.49693721961692	1.35139168119510
O	-1.71925358691998	-1.09358693332045	0.26626601631918
C	-3.53175541321572	-1.37551063502684	2.34989496410826
H	0.24639376767165	-0.64260442654578	3.40007109543690
H	0.27947788488861	-2.71089434845705	2.16742184164591
H	-3.88039643159481	-1.76604136109063	1.37837259360909
H	1.82155248441598	-0.90183118416328	2.65029704347645
N	-2.09497447617526	-1.61956750599014	2.41953710152135
H	-1.71394994054160	-1.97594248725103	3.29133666210120
C	-4.25382039945794	-2.08068187817838	3.49394271255516
H	-3.95108547503885	-1.61426994306523	4.44310731222979
C	-3.97694357501535	-3.56436303565446	3.51346487336681
C	-3.19412465953978	-4.13531368848801	4.52218381061473
C	-4.47271360735180	-4.39218415152547	2.49726139275422
C	-2.90871626947195	-5.50263247975917	4.51763395222533
H	-2.81128938713240	-3.50267016446257	5.32740989675177
C	-4.19024964434929	-5.75645977483859	2.48944695796518
H	-5.09221395919302	-3.96213921094066	1.70566000601190
C	-3.40494697848055	-6.31576930973127	3.50033948840359
H	-2.29896082953119	-5.93228152663956	5.31413229451244
H	-4.58688052603425	-6.38818817545984	1.69283096760865
H	-3.18415950886225	-7.38427644826413	3.49487542923461
H	-5.32830746233049	-1.88258745881385	3.37627328940092
C	-3.81872733835819	0.13665114342425	2.35911180508456
O	-4.46208756589334	0.67614558406590	3.21396397824439
O	-3.29705638172801	0.81218444380121	1.33936591461277
C	0.72274900323143	0.77207686775150	1.82093791785565
H	0.97983201043465	1.50131393280734	2.60593201250619
H	-0.30515111199307	1.01811372776217	1.49731053871487
C	1.68774084680587	0.88968025892826	0.64887751807927
H	2.69107144266177	0.55465168933239	0.97071380051963

H	1.36206763282783	0.19114218906276	-0.12158806653645
C	1.82623567804561	2.21402315652815	-0.07890089852320
H	0.83732107354826	2.53674373061233	-0.44300112629394
H	2.20216866118194	3.01006684700928	0.59110154355158
N	2.68993655114521	1.96551183270308	-1.23816810504203
H	2.67796185043850	2.75242675618874	-1.88542271702220
H	3.65965319547976	1.86538183137712	-0.93087061067857
H	-2.82179155315522	0.20752308581675	0.72734489032191

76

Reduced Intermedia L_s

C	2.50589088462679	1.73816410106376	-5.56034421710022
C	1.40073798947810	2.54623678135803	-5.32088065987253
C	0.41916939257743	2.13154715559095	-4.42132956059128
C	0.54189789565466	0.92103163369633	-3.74947935969301
C	1.65257873745590	0.10327826683480	-3.98333558193139
C	2.61805320191707	0.52158264861377	-4.89061549319901
F	3.43829478612798	2.12664294415794	-6.41086903912319
F	1.28136621592455	3.70372717871736	-5.94548315081845
F	-0.61803185944565	2.93043439523321	-4.22404164987719
I	-0.94496772430604	0.37478506014839	-2.38551024127500
F	3.69223997517034	-0.22477869177774	-5.12019619004393
C	1.87286716146783	-1.23457043755369	-3.30064150730601
O	1.75478226876119	-2.27826239381738	-3.90623856722234
N	2.21583636411148	-1.12158538216751	-1.99555521365526
C	2.69502254003415	-2.27517726030033	-1.25717339022631
C	4.23119986124404	-2.37182020328975	-1.28561094613099
C	4.91875340566284	-1.06220660392548	-0.97964048113395
C	5.31309250802212	-0.21781072011243	-2.02676043765919
C	5.92990547862589	1.00709191917790	-1.76744705329852
C	6.14940415620660	1.41402641842101	-0.44993395691622
C	5.75204840150492	0.58548923896199	0.60168397778534
C	5.14558998815182	-0.64428680273327	0.33907290636745
C	2.14201167621711	-2.33816125674334	0.17933805212993
O	2.83512668718135	-2.69886531603172	1.11566657544698
N	0.83564043867618	-2.02136506619891	0.31736052473009
H	2.42627120787874	-0.17062545175184	-1.66273892687390
H	2.28711437615218	-3.15696043873458	-1.77617134330910
H	4.53549665483174	-3.14737574173770	-0.57135490861225
H	4.51620434650217	-2.69947496628296	-2.29532513526285
H	5.13835705910310	-0.52867342508278	-3.06007254697210
H	6.23964427834750	1.64513898071901	-2.59714379893395
H	6.63261954362638	2.37052128732610	-0.24328539346544
H	5.92460129584158	0.89389405350093	1.63444454791194
H	4.83847160077567	-1.29628189879357	1.15908508583334
H	0.32415405256459	-1.66479244825818	-0.48715831387203
C	0.22708960706324	-1.83442819231885	1.61936223169298
C	0.86917228675860	-0.71986395283146	2.48825402177505
C	-1.23867794825342	-1.49041922919547	1.40659511041008
O	-1.64967794608184	-1.03811422909838	0.33538328132242
C	-3.47138609882848	-1.33410738482297	2.40993966383968
H	0.38429590358573	-0.75779174006843	3.47571117090689
H	0.29748271744053	-2.77647018580477	2.18710575511214
H	-3.82739783298526	-1.68625903650458	1.42638466189113
H	1.91909963227999	-1.00694457324283	2.64256533982732
N	-2.04139479662856	-1.61649988366394	2.47316396870332
H	-1.67102956028872	-2.01288823513418	3.33219431517362
C	-4.21315456404117	-2.05601113102893	3.53096224568196
H	-3.90586110298203	-1.62237703151841	4.49408532654577
C	-3.96508128403937	-3.54482628495700	3.51106185623659
C	-3.20288963244524	-4.15815299022118	4.51070992424580
C	-4.46516346581926	-4.33463936013595	2.46707417299627
C	-2.94171557435204	-5.52973185073619	4.47017252325115
H	-2.81678114270327	-3.55543326111722	5.33706638794275
C	-4.20651196430088	-5.70298748935266	2.42313502331774
H	-5.06869964554759	-3.87134285374250	1.68196444297602
C	-3.44157687706943	-6.30473965103528	3.42527337875687
H	-2.34770683819396	-5.99253626078055	5.25998493057343
H	-4.60584790192362	-6.30464245365817	1.60491305077593
H	-3.23934360340757	-7.37638095967518	3.39131174851791
H	-5.28284852587849	-1.83310477166954	3.41400483359607
C	-3.72310873413446	0.18319648726409	2.46314596201170
O	-4.36646863594602	0.71041382448033	3.32570911309291
O	-3.17254498462977	0.87846441647371	1.47227811751333
C	0.80180468923776	0.70467330120029	1.92499108441801
H	1.22556761840304	1.38640283891378	2.68025199306596

H	-0.24704649388324	1.01973709494822	1.77932403712885
C	1.57458392491433	0.81662613613741	0.61791900882195
H	2.55386285933419	0.31587468316651	0.74068907805970
H	1.03613340973049	0.26012060025255	-0.15239134872656
C	1.82835573565428	2.18210781388542	0.00652641525981
H	0.86938066249057	2.64184087042817	-0.28230207193199
H	2.31269388875406	2.86547382583422	0.72956123603553
N	2.62407621274332	1.95610632228931	-1.20478560282529
H	2.61903072734236	2.77438803528093	-1.81159484383089
H	3.60116237829176	1.79987687440587	-0.94921481845282
H	-2.70399519636820	0.28413409336223	0.84472425972089

76

Reduced TS11$_s$

C	0.60168363233653	3.50525172862788	-4.64206934035785
C	-0.69249192461593	3.95056100410289	-4.41611466559371
C	-1.68135608512236	3.04477514974712	-4.03110395719525
C	-1.38155429699188	1.69721338203555	-3.86698594802579
C	-0.07707436942707	1.24249247695703	-4.09397724702685
C	0.89401584054943	2.15536815965671	-4.47914663505483
F	1.55409210682070	4.36109624035810	-4.96807384866480
F	-0.98413562488593	5.23112653768923	-4.55288409808356
F	-2.89870705706783	3.51547566060827	-3.81591968307310
I	-2.90312668203762	0.41602409945737	-3.22238012534387
F	2.15147322198769	1.76374902756881	-4.66843180822928
C	0.31670836341953	-0.20521298935697	-3.87375837337173
O	-0.07882926919859	-1.10311738430151	-4.58217544577857
N	1.11717016767950	-0.36256536541636	-2.79032852430980
C	1.64926310117228	-1.63974440181152	-2.36508201724193
C	3.18656185469978	-1.69228379962137	-2.48249256368607
C	3.85204716521001	-0.59395279816596	-1.68595114063658
C	4.08959746210451	0.65870060968999	-2.26841647290221
C	4.61323345493858	1.71187541128940	-1.51487015759254
C	4.90580872097614	1.52838182325359	-0.16270805638577
C	4.69765285176356	0.27765097599552	0.42155423313336
C	4.18061063344792	-0.77518592033333	-0.33432554075625
C	1.23746344553174	-1.98476978750315	-0.92059037439425
O	1.86236917665776	-2.80749256061924	-0.27288320641120
N	0.18644826766020	-1.30400698610960	-0.41999542580531
H	1.41711239799749	0.47640901561360	-2.29733860507617
H	1.21071672314430	-2.39925746311528	-3.02978658495418
H	3.51011285514955	-2.67810432116396	-2.12616406297656
H	3.44530150007862	-1.60072077718896	-3.54646045178082
H	3.85930189227899	0.80985236951146	-3.32514000564797
H	4.79943286851251	2.67803158979732	-1.98900681233858
H	5.31155581098852	2.35118306538110	0.42832278469217
H	4.94207229911878	0.11861187968727	1.47357181459809
H	4.00990194937569	-1.75029364689932	0.12487999514088
H	-0.32932785301276	-0.65257417409022	-1.00352801289572
C	-0.08108292948792	-1.27286638447781	0.99912651954086
C	0.97727594370026	-0.45881542310774	1.78438343154608
C	-1.44252262255823	-0.63258256638694	1.21010181701254
O	-1.98179086226462	0.03237070580780	0.32440369849516
C	-3.27829212265795	-0.19781675747386	2.78270991038422
H	0.85453160899915	-0.68080325933455	2.85599237332753
H	-0.08681818541670	-2.30356007845096	1.38631540774248
H	-3.94455151581008	-0.37741136874885	1.92078149838416
H	1.95936560146227	-0.86095254071533	1.49151325757878
N	-1.96954660782613	-0.74424968749814	2.43875987618003
H	-1.49517308589066	-1.31751490502577	3.13026958498416
C	-3.83128868446894	-0.87826037214992	4.03219869448317
H	-3.20242359321236	-0.60007397798862	4.89082281986351
C	-3.90368030162970	-2.37887855186915	3.88844338433563
C	-3.04988384562333	-3.21136731154442	4.61964282189521
C	-4.81126124299032	-2.96446357505835	2.99551163685697
C	-3.09675588889671	-4.59854387475024	4.46294367851256
H	-2.34602724857921	-2.76806111886376	5.32915797884141
C	-4.86139401497666	-4.34772016389990	2.83708760808907
H	-5.49081707600008	-2.32767572307794	2.42293143019829
C	-4.00206912849986	-5.16951034707489	3.57039368180067
H	-2.42534701575491	-5.23312658539509	5.04378385502033
H	-5.57676792634687	-4.78859508006010	2.14080349152792
H	-4.04211772938724	-6.25286314309956	3.44721276104676
H	-4.82639352565229	-0.45357999856036	4.22393506933479
C	-3.20360556377169	1.32786185043428	2.97960124206463
O	-3.52124956480541	1.86776224756329	4.00207688499776

O	-2.76817694799442	2.01397876498434	1.92950702429779
C	0.94359441079722	1.05902747590901	1.57521286819553
H	1.74587073520662	1.49435785554086	2.19394592037706
H	-0.00119649469138	1.47539159960049	1.96785047170597
C	1.13162767383893	1.51306345641779	0.12944527795332
H	1.99743402552082	0.97768104908086	-0.30314475366734
H	0.24533515293551	1.25415268271234	-0.46944022240741
C	1.35369553621129	3.00637852810181	-0.05166197753754
H	0.48687036352945	3.55429781340973	0.35164180929739
H	2.23650267266591	3.32288529143348	0.53935608344815
N	1.46763252651425	3.30265412964633	-1.47800256347855
H	1.49157824087295	4.30899051514901	-1.63692424450689
H	2.35954183196806	2.94620321226938	-1.82429747512852
H	-2.58572720026951	1.41768778521995	1.16536773143295

76

Reduced Intermedia M_s

C	0.32575849890350	3.57552938052130	-4.07838983742896
C	-1.03185038283926	3.84783373397886	-4.10733659690772
C	-1.95019753796532	2.79666746664392	-4.06259708333211
C	-1.52155489494146	1.47700948349399	-3.98903066274872
C	-0.14528074815053	1.19254554172487	-3.96686719049106
C	0.75149328994193	2.25217027150553	-4.02128287492919
F	1.20485387325864	4.55961178839512	-4.08555081640929
F	-1.45740982521688	5.09617403018894	-4.15660687377162
F	-3.23513318401524	3.11048192832575	-4.07470411707786
I	-3.00245002645963	0.00313268222698	-3.82954736276042
F	2.06341125838079	2.03398604412742	-3.99026268098496
C	0.37343305998692	-0.22653351653972	-3.84024789053162
O	0.00210047268307	-1.11857439401060	-4.56984088626936
N	1.23496966642207	-0.39049663974050	-2.80397540368627
C	1.75599029766272	-1.67687074093757	-2.39502649482788
C	3.29085144009823	-1.75711569309400	-2.50929613023110
C	3.98092424337319	-0.67824904452900	-1.70766996734365
C	4.25088295199216	0.56924520774283	-2.28612177506248
C	4.80982947172502	1.60301150839832	-1.53138195881799
C	5.10553398423420	1.40432518957956	-0.18187968201180
C	4.86074757507223	0.15880700098476	0.39958469507693
C	4.30749285180477	-0.87448557004567	-0.35761874698108
C	1.33276268968351	-2.02051789434110	-0.95294134414500
O	1.92017724025728	-2.87595551653857	-0.31295452811353
N	0.31216978500361	-1.29597420376767	-0.45164510046617
H	1.53359272379872	0.43370327009632	-2.28609352891298
H	1.30654941895242	-2.42598579473331	-3.06373412457020
H	3.59258556283219	-2.75124661333566	-2.15634100901400
H	3.55455723333064	-1.66740122911389	-3.57208218968985
H	4.02004687149279	0.73023482929064	-3.34138904028856
H	5.02254221549602	2.56501433446559	-2.00273677447516
H	5.54074487905687	2.21154668446913	0.40971771590202
H	5.10538237358345	-0.01143096705351	1.44980648143270
H	4.10990111243486	-1.84641985698739	0.09816651637439
H	-0.16949474513664	-0.61632390447796	-1.03086185024963
C	0.01511298950848	-1.26941950843220	0.95985583156870
C	1.10705074939199	-0.52798476702787	1.76852729280447
C	-1.31686986331493	-0.55830897382554	1.13983877416645
O	-1.80177083336209	0.13038730135836	0.24001407169006
C	-3.16575825145529	-0.03589924522170	2.67682977993022
H	0.96539935061926	-0.76800848611873	2.83390078896348
H	-0.06306486321738	-2.30131573432364	1.33631244072864
H	-3.82527003011641	-0.18938754462648	1.80463151071445
H	2.06827852804982	-0.97491106971767	1.47132937356302
N	-1.87661563287612	-0.64065496152662	2.35572850225962
H	-1.44582889004733	-1.23511573241412	3.05791920879449
C	-3.76708715179748	-0.68510080466221	3.92087034854956
H	-3.14800905277709	-0.41715522485939	4.78965632057983
C	-3.88570985533550	-2.18453172535566	3.79563743814122
C	-3.08346978333747	-3.03231403057178	4.56686723589973
C	-4.78474283039482	-2.75498707898032	2.88440011799285
C	-3.17281071132643	-4.41967477488903	4.43198884273181
H	-2.38702871577786	-2.60044288340265	5.29053047642176
C	-4.87721615412736	-4.13839935027171	2.74780484695744
H	-5.42411077777652	-2.10626175000772	2.27989116885889
C	-4.06943428592558	-4.97555579849827	3.52141868045171
H	-2.54164319183900	-5.06602346231175	5.04414882113513
H	-5.58538345976462	-4.56728712654592	2.03680009225372
H	-4.14285215815443	-6.05899928419462	3.41535946229313

H	-4.75142660880317	-0.22559332806651	4.08656368967272
C	-3.02900392563409	1.48614718330539	2.87071702932120
O	-3.34675743989849	2.04347349243478	3.88369420308335
O	-2.53940565569766	2.14798665120198	1.82931301220465
C	1.15432694305852	0.99365199763953	1.59863787531790
H	1.97578411696719	1.36877848012578	2.23158123062379
H	0.23218588554295	1.44784659728042	2.00309511320291
C	1.36862138890461	1.49029484409083	0.16935512700741
H	2.20763823104286	0.93295848713036	-0.28708626532772
H	0.47036363025409	1.30198992110572	-0.43945188668725
C	1.66775825495817	2.97878042856400	0.06821430437434
H	0.83629536180675	3.54648868934282	0.51663955787129
H	2.57129509827261	3.20834203462818	0.66870576836560
N	1.78612063480961	3.35852304435253	-1.33510103753706
H	1.90699719742398	4.36538890897386	-1.43073609165220
H	2.62456622123261	2.93439117697383	-1.73314997773114
H	-2.37235404741875	1.53833251742053	1.07212704151400

76

Reduced $TS12_s$

C	0.92422320662554	3.08984990397882	-3.55220394802280
C	-0.43310374377024	3.34703743154918	-3.65406185513154
C	-1.32652120676059	2.28986564389745	-3.84085796018604
C	-0.87290530363284	0.97928792656349	-3.92964162376905
C	0.50367984446627	0.71249989427472	-3.83802867219381
C	1.37578836450583	1.77811453673976	-3.65604697618031
F	1.77970836939280	4.07206776953331	-3.34020808540166
F	-0.88468760747999	4.58343685840504	-3.55683233785363
F	-2.61381701563881	2.58553714919945	-3.91118311532651
I	-2.32249584470427	-0.52162474525302	-4.11273362960752
F	2.68602349823224	1.57367115029517	-3.54755493817955
C	1.04714331146115	-0.70227857620335	-3.88470976245715
O	0.77581318370028	-1.47219480355689	-4.77877483531072
N	1.80689635875800	-1.01369275034668	-2.80447449053080
C	2.31099192310950	-2.34223427238870	-2.53342966248194
C	3.85080684580808	-2.40202601336609	-2.53263066308433
C	4.45624307205821	-1.43937084575028	-1.53802620877911
C	4.74705066714014	-0.12191659242028	-1.91701575285621
C	5.22883410161010	0.80140065274168	-0.98655286440528
C	5.42507367469113	0.41990623104654	0.34165864593928
C	5.15792598017691	-0.89540284061431	0.72607741743088
C	4.68083427218756	-1.81767095330713	-0.20608442392888
C	1.77831175493934	-2.87264856808140	-1.18748701866058
O	2.32667550932009	-3.80001785556925	-0.61584808633102
N	0.71198961998932	-2.22124823079552	-0.68431641776752
H	2.02277279530580	-0.27632858453897	-2.13698684273000
H	1.92621738915364	-2.99527117771538	-3.33089914736423
H	4.13827923643749	-3.43372402110473	-2.29422766950502
H	4.19777302400858	-2.16677392716382	-3.54828914605915
H	4.59356583544779	0.18182558531924	-2.95479864015224
H	5.45920912172070	1.82086518017664	-1.30355566732993
H	5.80067499076343	1.14039182499916	1.07031950229097
H	5.32496061118936	-1.20748478681281	1.75879303067798
H	4.46410696654850	-2.84392788420175	0.09567469353962
H	0.25168700159540	-1.49162290715486	-1.21900979538136
C	0.28150380574840	-2.38205715597040	0.68318292020696
C	1.29943965272987	-1.79729712997067	1.69001134197964
C	-1.06192122880202	-1.67284856599904	0.82456063309326
O	-1.47707926610270	-0.90134398472491	-0.03424893722320
C	-3.00834293680743	-1.30340348195739	2.26370115710238
H	1.05812879134738	-2.18865344747295	2.69085441351425
H	0.15318384248596	-3.45459815981462	0.90087059679557
H	-3.79867231128210	-1.83205773858031	1.70635442826633
H	2.28087239324056	-2.21654006857001	1.41964256774259
N	-1.71211183540135	-1.88097733347548	1.98306002780782
H	-1.35261105607130	-2.57339014590316	2.63386904130451
C	-3.30694196104964	-1.38891522943378	3.77289712782385
H	-2.59009719608824	-0.74824837372080	4.30845077694939
C	-3.23111990591268	-2.80478156589293	4.29082936112748
C	-2.14848653861874	-3.22747645371863	5.07049760512691
C	-4.22699525914409	-3.73353616898144	3.95917687996550
C	-2.06017269575080	-4.55053139236116	5.51065059990515
H	-1.37103222266517	-2.50902758266547	5.34373479631424
C	-4.14218575513240	-5.05263265191159	4.39861555917307
H	-5.07985595008091	-3.41476965673241	3.35421774175002
C	-3.05653432146890	-5.46524601176322	5.17522193638703

H	-1.21142869419740	-4.86348298385880	6.12095027759292
H	-4.92805170682850	-5.76314417800173	4.13701574226203
H	-2.99102120029687	-6.49845182472203	5.51961584346659
H	-4.31379779358061	-0.97571831820457	3.92773399560864
C	-3.12098608336756	0.16147352054651	1.84812069507706
O	-4.17161978703921	0.63755165934207	1.54193339303167
O	-1.99066482132740	0.90096960742596	1.98917805956689
C	1.36874706658750	-0.26875984756372	1.75389247023497
H	2.14865332728317	-0.00271589937288	2.48692066772188
H	0.42186674541859	0.12517963946867	2.16221157150470
C	1.67984991328217	0.43081571045195	0.43094548898538
H	2.55829288996038	-0.05150505122285	-0.03648650686632
H	0.83310164591805	0.32662192023190	-0.26642996119081
C	1.96326564178253	1.91955660526659	0.56560421195917
H	1.09542085925957	2.41458831598148	1.03273773106802
H	2.81955983154155	2.06444900171178	1.25507373274289
N	2.17181104795583	2.49674146766575	-0.75712312703572
H	2.30279373325480	3.50517983878995	-0.69890567888349
H	3.03214021291081	2.12270477572724	-1.15890737433863
H	-1.52989212838568	0.91247881288086	1.13211905619969

76

Reduced Intermedia N_s

C	0.74748434453723	2.99213693092487	-3.53280285001753
C	-0.62198640269576	3.19048643760607	-3.59603558183540
C	-1.47433638628869	2.09701477652465	-3.76276170977692
C	-0.96825181982524	0.80710160403417	-3.86655262152204
C	0.42028664372426	0.59918694895291	-3.81004710987937
C	1.25131907570238	1.70060003288193	-3.64985912616679
F	1.56502883033626	4.01151140011025	-3.34739034632250
F	-1.12396704754145	4.40622136952930	-3.48292017021178
F	-2.77422404630517	2.33940936956509	-3.80096009262741
I	-2.35725222427223	-0.75236220581374	-4.02248817989559
F	2.57140326396299	1.55056453354706	-3.57634242926212
C	1.02073769663505	-0.79240926927210	-3.86445575901779
O	0.76784859934066	-1.57506959702594	-4.75295370756123
N	1.80853628971680	-1.06893205502067	-2.79544176914557
C	2.35266787393380	-2.37954951358993	-2.51427708127979
C	3.89298914054222	-2.39974910420797	-2.54957540905407
C	4.49495993787432	-1.40385496165580	-1.58615304722297
C	4.74013804680484	-0.08515819027560	-1.99216806598870
C	5.21713582339732	0.86554978555427	-1.08713875759670
C	5.45350266460479	0.51090465107907	0.24208336586009
C	5.23080327082512	-0.80451376819593	0.65309226750402
C	4.75923564122704	-1.75419506559676	-0.25391790247042
C	1.86613165408619	-2.89630743515044	-1.14485086979762
O	2.44733242699032	-3.80403036713946	-0.57335181067301
N	0.80555564621136	-2.25098622440357	-0.62542481923708
H	1.99566476333511	-0.32367226633577	-2.12815574236152
H	1.96627810013397	-3.05643390796668	-3.29075952414908
H	4.21294923054211	-3.41909851687500	-2.29949713088081
H	4.20974785895053	-2.17411702158518	-3.57722988096026
H	4.55378031294752	0.19790210377365	-3.03049069615169
H	5.41217338884367	1.88578312206157	-1.42475269873506
H	5.82529496151875	1.25312933131424	0.95064335505881
H	5.42801715006625	-1.09523743049844	1.68672005849090
H	4.57646986427954	-2.78084195776604	0.06832321858330
H	0.30567585456773	-1.55415313018134	-1.16887868968082
C	0.40077587460236	-2.38532764057679	0.75295924207738
C	1.44079923269527	-1.78950472470010	1.72826918552048
C	-0.94541481378416	-1.67573281101543	0.89110492168067
O	-1.41531406403182	-0.99630137009004	-0.00839788615051
C	-2.85091286155641	-1.19399657389181	2.30704003261721
H	1.22574336552250	-2.17129410301990	2.73888849853298
H	0.26920987472555	-3.45314510707759	0.99344492806120
H	-3.60918111839211	-1.65401681261935	1.65293248957375
H	2.41540643788820	-2.21219476040653	1.43914591210698
N	-1.55698862055383	-1.79643289682072	2.08814421832281
H	-1.18416613558371	-2.42921495974529	2.78873017506793
C	-3.27445516771002	-1.34877665878419	3.77729032747748
H	-2.57527174728447	-0.77773987992274	4.40654720909991
C	-3.30801753192993	-2.79323869106865	4.21446027544902
C	-2.34036502688323	-3.30135678023907	5.08789046580553
C	-4.29185125108438	-3.65980576946670	3.71955669454193
C	-2.35191084351888	-4.64789501179890	5.45980814291040
H	-1.57430266084788	-2.63222382810665	5.48851507580893

C	-4.30640608747928	-5.00246318383853	4.08990821342839
H	-5.05644853597125	-3.27409618324459	3.04012988841981
C	-3.33448683044906	-5.50089679105265	4.96108008112904
H	-1.59181349182686	-5.02804524015943	6.14427445518179
H	-5.08130263783528	-5.66437124717583	3.69954480267464
H	-3.34674571312650	-6.55259568681672	5.25137214330437
H	-4.26983855372602	-0.89278054060177	3.87992268936141
C	-2.87586914018779	0.28768641937536	1.95503274887572
O	-3.84849175142854	0.83820219432154	1.51944131997424
O	-1.74789810780105	0.92423272061565	2.27528531988223
C	1.50809312287800	-0.26009756877007	1.77586644033812
H	2.33125779077010	0.01540794211502	2.45676797415607
H	0.58262099497966	0.13180349279862	2.22941637728834
C	1.73081465349738	0.42978657585799	0.43092356999241
H	2.59483540943913	-0.03522019958450	-0.07901166686942
H	0.84770982557353	0.29886441056308	-0.21457880440617
C	1.98566896957278	1.92541282834909	0.54027423465750
H	1.12596467418919	2.40165863251699	1.04108955790017
H	2.86754133904220	2.09638884830823	1.19076757116242
N	2.12312908828665	2.49737823328683	-0.79375480386216
H	2.24658324089592	3.50736641348393	-0.74788501656254
H	2.96711733730917	2.12973209812532	-1.23437429071618
H	-1.85550752535169	1.86024194079263	2.03650655023778

76

Reduced TS13$_s$

C	0.09697977691582	2.77390623123934	-2.67505964967410
C	-1.28257799910895	2.65645877654009	-2.60090255561546
C	-1.90048513279527	1.46009223427796	-2.96926653763867
C	-1.15097935192866	0.37445054093621	-3.41003352226678
C	0.25064709537269	0.47669164214497	-3.46472994526097
C	0.84515894469498	1.68086845584882	-3.10109887958901
F	0.68778670697804	3.90722343754936	-2.34912506896594
F	-2.01116137442234	3.67776049757637	-2.18852129158090
F	-3.21842131604540	1.40795325024195	-2.89089232034593
I	-2.21737868837786	-1.35063726142322	-3.93216599678437
F	2.17034217970028	1.81803015587428	-3.13182278715989
C	1.11776683688056	-0.72038852720768	-3.81191729288737
O	0.92483831505642	-1.41143517414512	-4.78606347802317
N	2.06378654915265	-0.97180042107270	-2.86790305886681
C	2.72599101440749	-2.24972792530607	-2.70837802181304
C	4.25779499018794	-2.11585552947126	-2.67071256436917
C	4.71475091364481	-1.16448867899536	-1.59033836155021
C	4.77224259374400	0.21433488943865	-1.83849190866195
C	5.12103284107165	1.10947208779599	-0.82518264057419
C	5.41947586504899	0.63670441355190	0.45405046542339
C	5.37597569489441	-0.73497407271244	0.70956446090341
C	5.02603061413062	-1.62797072141697	-0.30423706267987
C	2.23145909144607	-2.95458039654684	-1.41970291088338
O	2.87767986904537	-3.84737562408525	-0.89812824297758
N	1.07368446163563	-2.46931575615846	-0.93768216983892
H	2.14637791650211	-0.31719051894010	-2.09381402033544
H	2.43608395864393	-2.87071021527741	-3.56906916222244
H	4.67255196216348	-3.11741493966796	-2.50094625198916
H	4.59009343957760	-1.76070347058774	-3.65592739144482
H	4.54009022808116	0.58819638603911	-2.83874515975179
H	5.16891893224468	2.17935114024326	-1.03873282264488
H	5.69654238315827	1.33452680540966	1.24616550772987
H	5.61827883326493	-1.11351812492172	1.70434889508728
H	4.97850639684543	-2.69996455081651	-0.10334501137519
H	0.55262184308149	-1.78878780975239	-1.48154798119922
C	0.50667517642171	-2.72092760977396	0.36624522475613
C	1.46551447752773	-2.33771711272635	1.51524358651579
C	-0.79992051771023	-1.91359266674488	0.38361395211270
O	-1.19595602181290	-1.32199555133758	-0.61162454727603
C	-2.65134698297112	-1.01271499258606	1.64511574099413
H	1.14682804596339	-2.86878557357483	2.42664183100788
H	0.24724123336831	-3.78895839236766	0.47044392017128
H	-3.48351326510803	-1.46034110284923	1.07920988379228
H	2.44319624290144	-2.76487934413312	1.24738431017417
N	-1.46679314575185	-1.83874447752923	1.55027358867788
H	-1.14345783229245	-2.34522937383651	2.36827012828865
C	-3.05997552424855	-0.82780471764402	3.11545676531172
H	-2.27775205912433	-0.24813710267959	3.62844823791463
C	-3.27277447443901	-2.14675846132033	3.81829563462694
C	-2.34250737798658	-2.61816564043679	4.75136820615021

C	-4.38518371556325	-2.94232407145500	3.51329570606948
C	-2.51708652611776	-3.85954621555689	5.36781566779669
H	-1.47642285820750	-2.00095418109103	5.00510191814641
C	-4.56239846418166	-4.17988469523814	4.12786085814022
H	-5.12109525950787	-2.58360705315033	2.78898808061886
C	-3.62686460401274	-4.64287575924319	5.05645224652233
H	-1.78440499598564	-4.21178430979730	6.09571901273842
H	-5.43593086067318	-4.78674089763980	3.88340018180226
H	-3.76651714081297	-5.61220011278717	5.53764032284856
H	-3.98331581550912	-0.23062935856354	3.12700987563895
C	-2.43390085349168	0.36149652859250	1.01269001963533
O	-3.27774077908914	0.94434662006943	0.39119311147659
O	-1.23655720326707	0.87783958026853	1.30052366660692
C	1.59517814223579	-0.84212166240713	1.82543932677351
H	2.43887147109678	-0.71481959218510	2.52439801154452
H	0.69377546388798	-0.49457604558202	2.35523419469341
C	1.80133475934315	0.07191776245211	0.62039653562478
H	2.68362143730787	-0.25746806787514	0.04241186760649
H	0.92477377940875	0.02513303127955	-0.04668476054158
C	1.98167551344000	1.53153494953469	1.00846903463762
H	1.17800683124289	1.81801198095384	1.70701245124968
H	2.93615336745900	1.64411992770016	1.56069672646090
N	1.88528979350766	2.36973498066508	-0.17988466915110
H	2.00990158488239	3.35335226006366	0.05344225316754
H	2.64416289672000	2.13651801181841	-0.82212461962448
H	-1.14812032374328	1.72707728251129	0.83458125612597

76

Reduced Intermedia O_s

C	-0.37083469060678	2.47083980952540	-2.32092387495124
C	-1.73083483877127	2.20686113108855	-2.30120474314614
C	-2.19294324105537	0.98059887864207	-2.77740378884961
C	-1.31777200510192	0.03119152998207	-3.29382160957718
C	0.06539037136750	0.28870328000379	-3.31491997959495
C	0.50567064155429	1.51253909753761	-2.82097821339445
F	0.09868809737170	3.62033612390827	-1.86078117615868
F	-2.57395539120980	3.10296410370548	-1.84125181257847
F	-3.49334776544260	0.75906013450173	-2.70634819677718
I	-2.17726711346362	-1.79194987255745	-3.86598348242050
F	1.80997830140285	1.80492132128268	-2.76819599808910
C	1.05823685071309	-0.77108640504523	-3.76107959740661
O	0.88534203778778	-1.44157083556858	-4.75309199500429
N	2.09070113847897	-0.95771136591193	-2.89136410147957
C	2.80772625213394	-2.21275849061518	-2.77321021788726
C	4.33229745427071	-2.03660691489668	-2.83371038631768
C	4.84493656938235	-1.08470536852215	-1.77962100120416
C	4.80319493182839	0.30082320734145	-1.99373829264885
C	5.22495102285654	1.18994733540639	-1.00436649637568
C	5.70265489512249	0.70473516747903	0.21447955834884
C	5.75277897287758	-0.67217287413828	0.43719197181964
C	5.32478429004601	-1.55965961277386	-0.55149816371836
C	2.40722822770845	-2.93215634663586	-1.45672701717857
O	3.12147243076795	-3.78527321622274	-0.95922585393837
N	1.23947618547382	-2.50188904225125	-0.94567799190881
H	2.16579927272578	-0.32775829870226	-2.09753815675934
H	2.48617575982973	-2.84422280116502	-3.61490666903338
H	4.78391668854341	-3.02795944801126	-2.70277907262921
H	4.58790013278299	-1.66602484337545	-3.83616474878030
H	4.43646656371502	0.68424903908498	-2.94903215802348
H	5.18792283907480	2.26522760349368	-1.18989725673495
H	6.03998126418707	1.39839509029974	0.98686353436318
H	6.12967159386772	-1.05927932552511	1.38568745261307
H	5.34950821001593	-2.63654953656153	-0.37506973173422
H	0.68320714742343	-1.84592825210287	-1.48535203157608
C	0.67726172053511	-2.73652833349496	0.36534079324982
C	1.67703157732041	-2.43360370277146	1.50151030244150
C	-0.57725198318421	-1.83927350404775	0.38920771229521
O	-0.91244570204658	-1.21436758510446	-0.61141226456524
C	-2.33176033337914	-0.72369711520382	1.58998454400466
H	1.35431992930902	-2.98131634152768	2.40120818557927
H	0.34420945064893	-3.78466564395814	0.46340253092727
H	-3.16052876999238	-1.02106386542087	0.92963718735914
H	2.62781207999581	-2.89383579569932	1.19607497063486
N	-1.27005735262552	-1.71592385735418	1.53302568293771
H	-0.98400184083114	-2.20483052884039	2.37505604244104
C	-2.83533607845820	-0.56136910211686	3.02843280646636

H	-2.01219545814655	-0.17866653569706	3.65091188477328
C	-3.36814380385590	-1.85387536117683	3.59853662729205
C	-2.67475267575304	-2.53995667991105	4.60111135583763
C	-4.55687686220074	-2.40632349561222	3.10286416833509
C	-3.15564666101468	-3.75342715000390	5.09937372192046
H	-1.75223598328114	-2.11324746798170	5.00401928721932
C	-5.03972278112144	-3.61511405124930	3.59856871060769
H	-5.11098427654448	-1.87814661065253	2.32253707378873
C	-4.33851770867087	-4.29366009007450	4.59863347428660
H	-2.60408224354531	-4.27443558819252	5.88375121964431
H	-5.96924949252715	-4.03012293034449	3.20527101201715
H	-4.71736280808675	-5.24014058103062	4.98740063013681
H	-3.62532025464807	0.20412889549412	3.01487979085410
C	-1.83704217202716	0.62737188461116	1.04790979957153
O	-2.49690052487689	1.31409704463940	0.30851754837714
O	-0.65369561091389	0.94324727539863	1.52642084533664
C	1.87400739810775	-0.96104825569749	1.87473320091505
H	2.73409253491467	-0.90035209589317	2.56240132452245
H	0.99722416693302	-0.60950588391012	2.43864659698230
C	2.09103975525069	0.03154277735120	0.72881242261686
H	2.99101878825928	-0.23747672295938	0.15080092671271
H	1.23252572075097	-0.00928054200882	0.03771243163007
C	2.20037851431282	1.47499407214902	1.24637883029991
H	1.89283936566510	1.51106207119091	2.30312924498846
H	3.24678366597780	1.81894473856694	1.20197924546775
N	1.29339039166973	2.36315111324802	0.50268448685925
H	1.38949716688818	3.32898168093004	0.81755507780287
H	1.55357011534193	2.37048087824668	-0.48412616406739
H	-0.14260953512088	1.66239355850539	1.00602594473614

Entire system

93

Entire reagents

C	-3.20204414927106	-1.12919421087232	-4.26435813098786
C	-3.52397378309683	0.17165352529928	-4.61985754128810
C	-2.77887266137668	1.22879550777681	-4.10516979177296
C	-1.70832340330571	1.01527274001514	-3.24483228748137
C	-1.37792022237623	-0.30309152143684	-2.88190721127352
C	-2.13220846524977	-1.35702733006151	-3.40380106288754
F	-3.91407601747042	-2.14253815920417	-4.72462390921478
F	-4.56958408800796	0.40725150378255	-5.39184703952479
F	-3.16524426693339	2.45553249046920	-4.43227814901061
I	-0.81536408322821	2.74806963473882	-2.40815596663551
F	-1.85512348462876	-2.61691402136891	-3.10796429403905
C	-0.28057452386543	-0.70327266920183	-1.92590459766189
O	-0.48901907491878	-1.52343624821247	-1.04499521016077
N	0.94350269911143	-0.16033864889637	-2.11591347919544
C	2.10479402061780	-0.71946477020771	-1.44715746086131
C	3.40157859095175	-0.08982575530304	-1.98269169627988
C	3.54776504340945	1.38110249458704	-1.66947471631944
C	3.13248564417093	2.36125657374048	-2.57775244181018
C	3.26831038268434	3.71878176797499	-2.27783484934642
C	3.83048552617662	4.11336538114616	-1.06384550765723
C	4.24683410745075	3.14384891726439	-0.14783858927618
C	4.10264331150762	1.79055339485516	-0.44794422622509
C	2.08253835338022	-0.62122395501701	0.08301637925735
O	2.78835946124173	-1.37905828097383	0.72567705378766
N	1.30935429568169	0.34478694567896	0.62430984017156
H	1.08337147345943	0.48669080100705	-2.88578955704156
H	2.13985333187414	-1.80348303590099	-1.64139488523724
H	4.22972520320733	-0.65329992085467	-1.53174183756188
H	3.44085075596408	-0.25586799463267	-3.06983253456243
H	2.71879093183398	2.06517703495294	-3.54628006809254
H	2.94636529943259	4.46707935714564	-3.00526194810142
H	3.95242080298919	5.17344185879093	-0.83278808693471
H	4.69068799993888	3.44391119033321	0.80317069264698
H	4.43137751072575	1.03266065734298	0.26871800545899
H	0.81856899853826	0.96535725417699	-0.01199102870355
C	1.16309975841837	0.50572266806430	2.05878569150388
C	1.65749077202820	1.84348577372810	2.62287254156249
C	-0.24196827532033	0.16552361617789	2.54647490792525
O	-0.58998173340086	0.46425191999596	3.68984357081466
C	-2.36697057920478	-0.95189125402586	2.10154261485659

H	1.74109664419164	1.71667410040744	3.71083125522349
H	1.78321080159055	-0.28995160565570	2.49981117183804
H	-2.83371856747075	-0.14095140074865	2.68572584054317
H	2.67570593119695	1.99688470187300	2.23154369016668
N	-1.04770366050906	-0.49080104425757	1.69812806861805
H	-0.67850115482365	-0.83664763715919	0.81222871291758
C	-3.21138419304655	-1.28359237984134	0.88314115296594
H	-2.65781150534031	-1.99043292908461	0.24302210244584
C	-3.65257287868067	-0.09267845503148	0.05821725526007
C	-4.46123985128956	-0.32278988964564	-1.06652322583701
C	-3.33974175432640	1.23120422317160	0.38661142450306
C	-4.95836333229568	0.73518615375283	-1.82323556223410
H	-4.71195757652567	-1.35192051046472	-1.33940330308252
C	-3.83099417692161	2.29444996463403	-0.37729798852923
H	-2.70802606376053	1.45088237751638	1.24948409359773
C	-4.64478621666872	2.05402489373046	-1.48114010946141
H	-5.59283637733440	0.53268989453553	-2.68962618855389
H	-3.56542447195250	3.31791668022166	-0.10295330341671
H	-5.02672484729014	2.88371344202218	-2.07841490470120
H	-4.10222255740884	-1.82795779914910	1.22832799096013
C	-2.25947959474289	-2.15584406564719	3.06111910414658
O	-2.60819359065326	-3.26697262884265	2.76031881760743
O	-1.79024905656847	-1.86833117728833	4.26357934663782
H	-1.42208819345461	-0.95277816151053	4.27964623502604
C	0.80083769609781	3.07609106792521	2.33239748474652
H	1.31158471874283	3.95072917573573	2.76779000500896
H	-0.16394912758844	2.98705715983461	2.85805024462608
C	0.56508794521101	3.31782372001844	0.84479596715429
H	1.51018667691443	3.16014543925797	0.29234834099247
H	-0.15695309472486	2.57781715072345	0.46400641141280
C	0.03524272647621	4.70541002238623	0.49771743343218
H	-1.03505053308089	4.77189151700030	0.75241473613282
H	0.55819700502158	5.46192500836539	1.11237860304987
N	0.18511850274498	4.95854506302987	-0.93772395738251
H	-0.24122482138803	5.84747873989412	-1.19654798271066
H	1.17706262970264	5.03333844020669	-1.16801007462035
C	1.44316939591596	-5.98809168409041	1.29958888605060
C	2.21865360766736	-5.56908055586603	2.54816484236286
C	2.49285209372373	-4.05850790811209	2.55398024032838
C	1.22199257336839	-3.26536283041524	2.34828075528962
C	0.38613880548350	-3.69537581812085	1.16467115625484
C	0.13380903510110	-5.20883213155150	1.18303184678231
H	2.96902891442901	-3.72207561551229	3.48431555508558
H	1.63052040674397	-5.83225551280248	3.44292981316013
H	3.16954186484517	-6.11696611814297	2.61978158673189
H	2.06180926590040	-5.79749028107481	0.40522978970925
H	1.24151950968727	-7.06941619969405	1.32425789045303
H	-0.55338271854796	-3.12986869927507	1.15528025648598
H	0.95559964680598	-3.42225034048895	0.25909294496309
H	-0.51762493255205	-5.44730413593052	2.03997239404566
H	-0.41551391966727	-5.49896730006432	0.27580413981365
H	3.15391236195826	-3.79519170926190	1.71077532766925
O	0.90138573907005	-2.35497238427588	3.08186036741210

93

Entire TS0

C	-3.30185554415351	-1.12170576489363	-4.25464564329671
C	-3.60846136277118	0.18452698492293	-4.60251373139045
C	-2.82600826674779	1.22671123803530	-4.11288484062718
C	-1.73441041014681	0.99313780275859	-3.28547879996776
C	-1.41529898676919	-0.33221110683162	-2.93211219699348
C	-2.20845826439041	-1.37061035903302	-3.42896211202824
F	-4.04360242479667	-2.12170444164658	-4.69577810462867
F	-4.66727772142696	0.44172342967497	-5.34821624928355
F	-3.20138908832798	2.45960441756485	-4.42844649032569
I	-0.81805514818319	2.71895542833927	-2.45932166152018
F	-1.94948097082665	-2.63700227091361	-3.14717385566537
C	-0.29732996899888	-0.75427029503228	-2.01007878884145
O	-0.48231976727334	-1.60855997218198	-1.15516394363357
N	0.91772507345359	-0.18935108190789	-2.18925416988925
C	2.07752531813220	-0.74043565090993	-1.51224778193629
C	3.37763269492481	-0.10918812939568	-2.03536505178192
C	3.52155025300658	1.36016878420099	-1.71513591661210
C	3.13457787048797	2.34374467009985	-2.63188568967242
C	3.26921157487571	3.69998140443195	-2.32553302304045
C	3.79938568207589	4.08928869017827	-1.09547615722129

C	4.18650767303383	3.11594181211358	-0.17062524871097
C	4.04570789838037	1.76408676053873	-0.47813572412547
C	2.03545819218222	-0.64274697938094	0.01797293176898
O	2.74710397793635	-1.39015258006455	0.66836965114420
N	1.23423247325608	0.30247964681534	0.55488965745344
H	1.05120352553263	0.48900965890219	-2.93289223799004
H	2.11621381360001	-1.82444858075158	-1.70440225836026
H	4.20172416031118	-0.67414542263983	-1.57876879380563
H	3.42618593843490	-0.27185400997920	-3.12251329905132
H	2.74404794209259	2.05043109783334	-3.61082820835839
H	2.97159453758454	4.45131509426552	-3.06017574827570
H	3.91982659370828	5.14832316691969	-0.85878329135269
H	4.60537307357222	3.41194036039996	0.79289084005473
H	4.35289027007097	1.00304649412627	0.24467258695458
H	0.73384681969224	0.91675206473108	-0.08034372510759
C	1.05670095800068	0.43175105120059	1.98940978405266
C	1.50612339875029	1.76496757001370	2.59901299558794
C	-0.34524595631393	0.03125998602404	2.45143927326189
O	-0.73101999337304	0.30083683538576	3.58164565859426
C	-2.44383405414542	-1.12145316052166	1.90073573785091
H	1.54313268493296	1.61220455688264	3.68624927631145
H	1.69954265269203	-0.34684782368782	2.42460216556112
H	-2.86860001919283	-0.38713690354509	2.60745599387929
H	2.53946493024710	1.93828905540035	2.25792936259657
N	-1.09802704218963	-0.67959562309421	1.58447765515803
H	-0.71696711016046	-0.95457690436740	0.68010158366563
C	-3.29518851601451	-1.21846859584414	0.64327442784145
H	-2.77108757033359	-1.85488257984169	-0.09053958716845
C	-3.66661290066645	0.09478831688100	-0.00836721571320
C	-4.47997120283607	0.05809737799658	-1.15194595985693
C	-3.28602571938638	1.34471690023893	0.49409442967276
C	-4.90762865643303	1.23107642202256	-1.76926983162483
H	-4.79268947972122	-0.91193667378028	-1.54958407944811
C	-3.71367260783148	2.52262820073499	-0.12550590748844
H	-2.65613952925709	1.41428864734626	1.38371415937686
C	-4.52340727418425	2.47319070576169	-1.25773860976215
H	-5.54208533472824	1.17882125577135	-2.65748804660684
H	-3.40555723225122	3.48684954415240	0.28392811152594
H	-4.85246791204497	3.39380053190581	-1.74248447536258
H	-4.21463815403167	-1.75846064757016	0.90732043097091
C	-2.46520240845352	-2.46076372132435	2.66586798634754
O	-3.23544582905324	-3.34534908284354	2.40741740750726
O	-1.61083261990872	-2.58004611930512	3.67294923577923
H	-0.99008580266534	-1.83257487444843	3.74553204946339
C	0.65356544683317	2.99710895356238	2.29943668846085
H	1.13176264873645	3.86241762441686	2.78742144567388
H	-0.33728089217578	2.88456352517403	2.76957098243516
C	0.49844449859033	3.27542079905797	0.80754022372215
H	1.45881380789485	3.08074906235030	0.29457554561830
H	-0.24051973973399	2.57687691845251	0.38314713207691
C	0.05852570231433	4.69239933079006	0.45988476592328
H	-0.99724765745567	4.83797578225472	0.73798091493231
H	0.64683998401341	5.41337243361143	1.05823026131445
N	0.19750227724842	4.92656601822390	-0.98027646798822
H	-0.20502353283232	5.82540753919022	-1.24259613397668
H	1.18831059369582	4.97092739189051	-1.22236364921642
C	1.65587482046399	-5.98437642624814	1.59259860023751
C	2.49366511179981	-5.49297611419939	2.77295341857902
C	2.74089422016048	-3.97840131285207	2.69525145905899
C	1.44108830128598	-3.22565609794365	2.53365432690282
C	0.57192229830126	-3.71335034671778	1.39756030247145
C	0.33203863684896	-5.22569761929749	1.50648422510958
H	3.26226131621898	-3.59489754372521	3.58192911435191
H	1.96306696026253	-5.72202608579329	3.71196217270620
H	3.45719081588241	-6.02124906632768	2.81479841687879
H	2.22139111690820	-5.83458130788673	0.65654299244693
H	1.47001908446858	-7.06475848470841	1.68481653367861
H	-0.36699078785100	-3.14954089308326	1.37479903192839
H	1.11758397072823	-3.49222784460735	0.46420798864564
H	-0.27156864351320	-5.42696313139948	2.40724278343072
H	-0.25990315424618	-5.56458511305695	0.64438987863546
H	3.34575595279776	-3.74246597464517	1.80339800457721
O	1.12390871137476	-2.31829062531793	3.27497610552944

93

Entire Intermedia A

C	-3.18592132246083	-1.09697876438117	-4.42272386357934
C	-3.48920457531527	0.22739162339203	-4.70838178535634
C	-2.74932755735050	1.24440949011047	-4.11045519205404
C	-1.71438666459769	0.97236943123384	-3.22253649511518
C	-1.41194259101927	-0.36857820774838	-2.92087296841675
C	-2.15493807972922	-1.38387309642970	-3.53193522128883
F	-3.86017125793008	-2.07409084262344	-4.99963493013503
F	-4.47560932450793	0.51814489042369	-5.53465845036693
F	-3.10028329614374	2.49164095772000	-4.40004111817593
I	-0.81037180202293	2.67083985813976	-2.32024658083483
F	-1.89032497501711	-2.66041394837419	-3.31034343020133
C	-0.35756401720266	-0.82925749511241	-1.94486583898125
O	-0.59273992035620	-1.72077949836504	-1.14195540679160
N	0.85585869207781	-0.23905258712067	-2.01354849167763
C	1.96958721701804	-0.76863481034660	-1.25258700968826
C	3.30204285050080	-0.17800470429206	-1.73563007915224
C	3.43664020444139	1.30491429297401	-1.48364526673429
C	3.10520126943975	2.24054225537903	-2.46978451388489
C	3.23280651507221	3.61031835194245	-2.22782986336377
C	3.70892300189435	4.05935170063678	-0.99676158998277
C	4.03886051418985	3.13450688491895	-0.00305301474794
C	3.89731532343081	1.76961741842897	-0.24229271702180
C	1.85254138589922	-0.61151386725108	0.26875630754433
O	2.62129299243230	-1.24644107542828	0.97422507642140
N	0.91149297217301	0.22817420063111	0.74657331428011
H	1.02848344650639	0.48143256788085	-2.70789319407436
H	1.99969838546773	-1.86030091412688	-1.40026962175928
H	4.09402512088454	-0.72217990221912	-1.20412674552551
H	3.40709785290835	-0.39731277890791	-2.80840445874158
H	2.76464169498194	1.89765137577456	-3.45145044692467
H	2.97348643821364	4.32423408129513	-3.01216893067184
H	3.82906107074080	5.12863595732069	-0.81053114414492
H	4.41197245743535	3.47920170573685	0.96316320305263
H	4.15895873153955	1.04596815447508	0.53428071756286
H	0.34944463117632	0.75512878918850	0.08285488052003
C	0.62080435176451	0.33741024779890	2.16510004533421
C	0.90400141349638	1.71049829317819	2.77781426812149
C	-0.81552608311848	-0.10871877848945	2.48700774035265
O	-1.36962735914737	0.26438398670962	3.50594073456018
C	-2.76946595239427	-1.37489229483588	1.69733355564033
H	0.70951701106481	1.60441239738293	3.85253952619393
H	1.26822917802714	-0.40414827274717	2.65690177838365
H	-3.20168160410640	-0.84661119473122	2.56274595251600
H	1.97737601353135	1.92791165567284	2.65686410349993
N	-1.39714532747011	-0.93925791991016	1.58654558546916
H	-0.89619956263558	-1.19776120268449	0.73803167647212
C	-3.56936538201799	-1.06806521603615	0.43336221770817
H	-3.07825622503569	-1.56539151073636	-0.42092787058988
C	-3.75884208427102	0.39801254749692	0.11590570881158
C	-4.28956781025348	0.74474410492520	-1.13536690567852
C	-3.48438787492878	1.42724509197209	1.02521003426407
C	-4.55087634888750	2.07131837632734	-1.46968802487336
H	-4.50596416360874	-0.05071935992246	-1.85550112085773
C	-3.74709116631607	2.76008330704949	0.69342161559260
H	-3.07565716749793	1.20224989614950	2.01309411160234
C	-4.27945739734254	3.08857280537894	-0.55183198786177
H	-4.96231036162299	2.31374496119070	-2.45212308144851
H	-3.53408678567903	3.54447144696731	1.42293462903578
H	-4.48235729339752	4.12973269052909	-0.80881467160842
H	-4.55325540747543	-1.54576370743751	0.54629289443777
C	-2.90812564108827	-2.86525767559842	2.04833643322609
O	-3.93806513946780	-3.45766773870858	1.86420773992764
O	-1.86316848410553	-3.45931622709246	2.59956326218301
H	-1.06113918602099	-2.89308625090034	2.69202830466811
C	0.05992156734296	2.85805360309836	2.21188141611012
H	-0.10342440812239	3.61161329372256	2.99864147675352
H	-0.94559788152150	2.48277687595532	1.95100322557497
C	0.67129553040329	3.54834402063642	0.99610834701431
H	1.63556501229359	4.00614594747182	1.27434969332638
H	0.91653816915864	2.81284883788102	0.21086387726752
C	-0.24768306691955	4.60878499031378	0.40414661795355
H	-1.27805192498302	4.21519235801278	0.36696151991345
H	-0.26777307329877	5.49203535315879	1.06849351818358
N	0.15847228450792	4.93260213807006	-0.96366741482418

H	-0.32531511235324	5.76222800580495	-1.30515127555366
H	1.15791795617314	5.13643753937826	-0.99861326305997
C	2.54935989738372	-5.91424959619104	1.46123310107900
C	2.93497727578389	-5.30635537946024	2.80982989133726
C	2.79075252455609	-3.77502950764768	2.79777134472007
C	1.40596963037390	-3.39153226393835	2.34680035949060
C	0.97701595016240	-3.98482150279568	1.02740455528871
C	1.12910698301998	-5.51389909895020	1.06263327648927
H	2.97973692031983	-3.33129384721378	3.78379429567219
H	2.28276299904581	-5.72321264906712	3.59482413448603
H	3.96721225825776	-5.57333446438318	3.07837738119866
H	3.25611585118924	-5.56541045754418	0.68839486922672
H	2.63648075598320	-7.00996634303633	1.50274666587216
H	-0.04726624022712	-3.67318254328287	0.78659100795717
H	1.65545854332536	-3.56950963378920	0.26368794010875
H	0.40917784401799	-5.92593809151790	1.78894180156053
H	0.86476095419478	-5.92959846190217	0.08004298700370
H	3.49184581807990	-3.33608793484037	2.07001560719991
O	0.67122843708673	-2.69946214171808	3.02999765757888

93

Entire TS1

C	-1.66323179150539	-1.02140874374808	-5.57749124783471
C	-2.47338698903588	0.10842220168702	-5.53492349965760
C	-2.55747082830855	0.86314237012166	-4.36539060958300
C	-1.83816759688212	0.49455678774839	-3.23393728422137
C	-1.02351629623172	-0.63956817833341	-3.27178822290974
C	-0.94174495696149	-1.38337210975539	-4.44279710456547
F	-1.57753501669511	-1.73469861291302	-6.68402756933075
F	-3.16247065255174	0.46435664577752	-6.60153884514995
F	-3.34105934735069	1.92870360299385	-4.36721996930446
I	-2.05078397967294	1.62515073779710	-1.49235544848937
F	-0.16204449764466	-2.45468691670292	-4.49609904744438
C	-0.22410838305682	-1.11815719651527	-2.07941987254988
O	-0.65829657995208	-1.94382530815907	-1.29810588164615
N	1.00362129330955	-0.56349626755367	-1.97937106082341
C	2.01559137339631	-1.11140736474986	-1.09909442458653
C	3.42164537509228	-0.70773999510431	-1.56641605635139
C	3.67145664729271	0.78008890779584	-1.48635521773732
C	3.45371396521921	1.61083522949414	-2.59244724489687
C	3.67102132025648	2.98765756150196	-2.50721948483449
C	4.12668340752871	3.54884265057648	-1.31495759973837
C	4.35087907691176	2.72980914848202	-0.20622200643512
C	4.11706786714120	1.35842770943643	-0.28804124350934
C	1.84097086661859	-0.78185986607973	0.38612081957129
O	2.65779209034045	-1.22067840359088	1.18106557652595
N	0.75976439380380	-0.06076752826593	0.74234771960340
H	1.27355251585767	0.15153131637334	-2.64912053926822
H	1.93619973354936	-2.21230839313275	-1.13849686460345
H	4.13453895822866	-1.24368563249294	-0.92682397487044
H	3.55530564864840	-1.06662129240716	-2.59713551217375
H	3.13683987272036	1.17460949027527	-3.54456343255866
H	3.49626150440061	3.61847223626178	-3.38052984927653
H	4.31299531843001	4.62254243847716	-1.24948416307638
H	4.71076492302013	3.16237583211233	0.72916439452283
H	4.29146618916281	0.71730742706360	0.57918131096437
H	0.20336199178276	0.37079220435778	0.00923606048297
C	0.49866195470284	0.28332488132205	2.12521287930200
C	0.86407902883010	1.72573428520731	2.48840004257217
C	-0.93423793813355	-0.03403873271698	2.56831653451338
O	-1.34167504231566	0.40668213765717	3.62854328685987
C	-3.06163856196896	-1.16833555289084	2.10758390857808
H	0.65638185968790	1.83015500012989	3.56076829688180
H	1.12496591007107	-0.39112057321938	2.72800625661680
H	-3.27423213028144	-0.64170085440578	3.05254957215396
H	1.95047805342644	1.84647433843718	2.34934207062128
N	-1.69448602766946	-0.82186360791989	1.76135815877853
H	-1.31789468032843	-1.14952991047666	0.87280279649142
C	-4.08499788613437	-0.76812034571479	1.04924597082228
H	-3.74326702481581	-1.15011097190554	0.06976368676099
C	-4.38920300493943	0.70530983563639	0.91322305097356
C	-5.35681122949939	1.09180000001942	-0.02630912392423
C	-3.72926924138435	1.70509224825906	1.63532508459962
C	-5.63570024270004	2.43455488118461	-0.26461922350807
H	-5.88498782409995	0.31957617392944	-0.59161834256878
C	-3.99614344240478	3.05692241936055	1.38644523180302

H	-2.99759256429843	1.44742476451705	2.40317644983013
C	-4.94247016080533	3.42763226386388	0.43272462400570
H	-6.38897023537313	2.70962589662351	-1.00497367024461
H	-3.46019154396745	3.82113244658983	1.95334509079107
H	-5.14801149703595	4.48181600561826	0.24037558675447
H	-5.01305011499286	-1.31043106282414	1.27397984607961
C	-3.22570738497138	-2.66057538717607	2.45309290113789
O	-4.27218341143537	-3.23360024097946	2.30438242870049
O	-2.18232110262924	-3.27733292820707	2.97863879507372
H	-1.35006403192426	-2.74973787397853	3.05135980385380
C	0.11014394843539	2.80447102060132	1.70124368053510
H	0.01173190438401	3.70147417877829	2.33364929002151
H	-0.92789723485228	2.46977696058965	1.50999745089997
C	0.76992308772971	3.21986925858185	0.38782882392414
H	1.81706787003823	3.50624031305519	0.58280051820103
H	0.82199980293952	2.38003135934550	-0.32587511840193
C	0.05453242521393	4.37817796400033	-0.29352790587030
H	-1.02984992361549	4.17055285844793	-0.32485935369976
H	0.16912413089903	5.28353907079951	0.33563458817410
N	0.53509454887465	4.54400057464180	-1.65843660568827
H	0.12928558735362	5.37820014998216	-2.07897377089186
H	1.54414733866867	4.69387737350942	-1.64508292783456
C	2.92753197696381	-4.67975620228134	0.87706183156253
C	3.19584192044495	-4.75965227377727	2.37857080013171
C	2.49560938591709	-3.61726846115835	3.11407876672004
C	1.08224642740145	-3.33405856106719	2.66675301024431
C	0.58553958147416	-3.81614168815828	1.32082144587621
C	1.43584100939528	-4.87568675155669	0.61535805865016
H	2.47749062543291	-3.74935158485143	4.20533731624927
H	2.83454771831993	-5.73099048801470	2.75525390793704
H	4.27347723191726	-4.71908253540435	2.59140333952651
H	3.24898715432185	-3.69147106642866	0.50415561864262
H	3.50923707201851	-5.44308341951224	0.34009531257673
H	-0.45430859013265	-4.14822510598545	1.45815662048103
H	0.50779417236057	-2.90756593536164	0.69876107561503
H	1.14682374684118	-5.87687556351023	0.97375764132689
H	1.21194668597050	-4.85268533817835	-0.46157259588196
H	3.02415809030123	-2.67098545756253	2.91349451123919
O	0.35479440551095	-2.64743287429411	3.36793007117998

93

Entire Intermedia B

C	-0.09754027971118	0.18395911510397	-0.05083288369144
C	-0.16722691328227	0.23900093753508	1.33067858275433
C	1.00626184778360	0.19228038366931	2.07841922283924
C	2.25044297355375	0.08513592895304	1.47085030942354
C	2.33738774122540	0.05736009563178	0.06363642332043
C	1.14735326009953	0.09328616469598	-0.66576357044591
F	-1.19727070591422	0.22297580450412	-0.78037967939643
F	-1.33438001807707	0.36394371777349	1.93212727230990
F	0.88190796908951	0.29259248444302	3.39348302086267
I	3.89344731632713	0.03933343115564	2.78309067555187
F	1.14295206876790	0.04896293262615	-1.99769076964738
C	3.67271036263697	0.02231960274281	-0.64735936537136
O	4.64633037195434	-0.53526611290648	-0.17364239764409
N	3.74261685106635	0.71116850404962	-1.81172459452244
C	4.97544896357278	0.73110284282093	-2.57302984886824
C	4.74503629145135	1.34453058085136	-3.96518949381366
C	4.23486148213803	2.76380881433878	-3.88877740539723
C	2.86411290841817	3.04261167047704	-3.95323107314871
C	2.39131470680782	4.34724456492752	-3.79428661764738
C	3.28758936918176	5.39233471485536	-3.57472386886690
C	4.65848868869319	5.12843915701910	-3.52109763036192
C	5.12673839659938	3.82511499936396	-3.67725219690347
C	6.08281558643214	1.48301370701462	-1.83849317531766
O	7.26210897840578	1.30441615247832	-2.12961812922174
N	5.70000852297719	2.33476394870761	-0.87255301515774
H	2.93026955346468	1.19748473122053	-2.17462320988285
H	5.36697024427811	-0.29471594578323	-2.67144550220414
H	5.70429023062620	1.30618354128722	-4.50033733585071
H	4.03317773409083	0.70477181188412	-4.50637382607536
H	2.15627093647520	2.22897422347444	-4.13890709987649
H	1.31974392807735	4.54608998367884	-3.84941413342324
H	2.92109677358498	6.41303265069859	-3.45461547339922
H	5.36655293263000	5.94364672457948	-3.36204472392473
H	6.20048999928206	3.62078753407806	-3.63633141894633

H	4.71081901876440	2.51630119402171	-0.75256600317249
C	6.66671405278331	3.10420358915752	-0.12534317711181
C	6.02425440251477	4.22675518854340	0.69177108008962
C	7.58256258295678	2.23384439554339	0.74416265226033
O	8.57446782757729	2.73937792511197	1.23918719780557
C	8.19107380363198	-0.00817744642301	1.49423219255520
H	6.85573451769346	4.72312635237194	1.21109011198486
H	7.37412201808807	3.56147318214425	-0.83723493179830
H	9.00933552617059	0.59925748392321	1.91360717290802
H	5.60246722327779	4.96343036767653	-0.01157789974013
N	7.26274824008370	0.92236698849107	0.89217593967287
H	6.37948246646391	0.55808855804368	0.53013618382016
C	7.57564514969240	-0.86576488281949	2.59431742248855
H	6.73553615077274	-1.44277100993561	2.16960534028323
C	7.09799031051068	-0.11629439113038	3.81762078257848
C	6.36942828881910	-0.82362849334553	4.78827570186474
C	7.34552139925755	1.24495895691691	4.03547920225241
C	5.89165860500928	-0.19120956471618	5.93515610573926
H	6.17182207303789	-1.88788242211406	4.63369845138721
C	6.86422635648301	1.88015272737868	5.18401802769029
H	7.91549237703436	1.83087339669035	3.31165618127017
C	6.13367958316659	1.16974941570745	6.13469873896237
H	5.32491470392966	-0.76259939991096	6.67234124808160
H	7.06511317452163	2.94289015911467	5.33098700776439
H	5.75669959826890	1.67032033404500	7.02782073721452
H	8.33373632154410	-1.60529038663172	2.88936901097359
C	8.88610257437857	-0.90400204250608	0.45346694260888
O	9.46368981596841	-1.90720474997651	0.77934287463574
O	8.89478891997210	-0.49758914230798	-0.80828621654296
C	4.95376066764928	3.81817166740433	1.71108137487176
H	4.83828362244632	4.65991503837491	2.41298220488740
H	5.30833158262835	2.96579934309140	2.31819792458518
C	3.57289617866583	3.50187814185401	1.12618081883796
H	3.35579598489734	4.19537414344680	0.29173964134366
H	3.53282857541138	2.48147102347877	0.71082492113068
C	2.44243307445887	3.59871912477949	2.14409353871693
H	2.63126115946687	2.87781901999814	2.95685704943528
H	2.46704372831777	4.60529865039343	2.60694436980282
N	1.16435854797796	3.26919028570382	1.52339972109792
H	0.43148225929846	3.23490243094873	2.23090748432419
H	0.89407960946190	4.01189610112305	0.87878021616411
H	8.30833477649042	0.26038588146142	-1.01439859727487
H	10.69904415245438	-4.06146693729077	-2.20717761469898
H	10.44102153220231	-2.44765863582528	-1.53212053427392
C	10.15604772616331	-3.11660510467598	-2.35702019590816
H	11.62491337762064	-2.29969705807084	-3.72489209326249
H	8.33584039101514	-3.67730150366731	-1.26068154591880
C	10.53992111612978	-2.47895386848126	-3.69056055331717
H	10.30507341314927	-3.17196377425854	-4.51839492016024
C	8.65076562325987	-3.38262743401566	-2.27104087887399
H	8.38646296330035	-4.21357438739190	-2.95211957584186
H	10.05280376035561	-0.47055138714040	-3.08401894141371
C	9.78356275043472	-1.16731736154883	-3.89266418529360
C	7.77322731781371	-2.22976523961365	-2.71577093573959
H	10.07166510406126	-0.69131922147514	-4.84161073957224
O	6.67363912312197	-2.05614793513581	-2.23559769350079
C	8.26892110898049	-1.39049164874563	-3.87649532335598
H	7.97498706573617	-1.95253614986222	-4.78281223390772
H	7.70869155194956	-0.44553688587369	-3.86851884946533

93

Entire TS2

C	0.03378232202694	0.05406865671831	0.03231504325804
C	-0.01685140691981	0.05878348823635	1.41542465404591
C	1.16948339174073	0.07607701682900	2.14412211392513
C	2.40752063185193	0.07846111622449	1.51454910994945
C	2.47347398766338	0.09267316779388	0.10548941055627
C	1.27118712034022	0.06920766534087	-0.60323567130356
F	-1.07772840353003	0.03814028278582	-0.68034902138410
F	-1.17946742343954	0.07651076504425	2.03869235502854
F	1.06090333960749	0.11763104119548	3.46409249287630
I	4.07105732234128	0.11705567305207	2.79801820083211
F	1.24573528648016	0.06520803885272	-1.93582005441334
C	3.79626016007566	0.14310260458656	-0.62814900755744
O	4.80704211748103	-0.36493080962106	-0.17554175977776
N	3.81458280062123	0.84156501617675	-1.78884642544738

C	5.03354854776916	0.91307058531859	-2.57036210888789
C	4.75272617242460	1.49429367233232	-3.96632705452493
C	4.15508028613864	2.87921541171926	-3.90087815100472
C	2.76939725490771	3.07006049907572	-3.96744632533742
C	2.21530242245367	4.34449504037140	-3.82791766281118
C	3.04417522082635	5.44705847414180	-3.62489725283905
C	4.42889494652218	5.27057293368965	-3.56717050176930
C	4.97841236617481	3.99709395740486	-3.70362496191482
C	6.12119378844507	1.72327324466516	-1.86542775223814
O	7.29829863354485	1.59484014968359	-2.19469698698662
N	5.70932606221498	2.56396629273618	-0.90450255898654
H	2.97367599661650	1.28624022288033	-2.14050931077641
H	5.46628801357234	-0.09583536081446	-2.66767005494872
H	5.70820173586339	1.50853993709537	-4.50908318604815
H	4.07788153474711	0.80499206535450	-4.49371464519653
H	2.11461685182415	2.21047387036637	-4.14037736850403
H	1.13331852761061	4.47463935293414	-3.88575255466699
H	2.61427348934863	6.44456124024510	-3.52087882792527
H	5.08409132707723	6.13099328456928	-3.42026761896431
H	6.06286849135067	3.86141126333922	-3.66070449854775
H	4.71041124097831	2.67791107782722	-0.78329572766774
C	6.61107303105852	3.37951549135354	-0.12129875783108
C	5.88020830604572	4.49182490519667	0.63412594862092
C	7.50327541720514	2.56167735491635	0.82404987462478
O	8.43614110653557	3.11469585049713	1.37736738213426
C	8.23397561276515	0.29828430954109	1.39425853762792
H	6.66488500275115	5.05613024115474	1.15672224585955
H	7.34048810525061	3.84588297672242	-0.80423132137933
H	9.05564647545292	0.88745145812003	1.82634322991966
H	5.43136595548660	5.17405706096723	-0.10662459972229
N	7.21175183629953	1.24676894542574	0.97804491665164
H	6.38678504077004	0.85290945282092	0.52481542867649
C	7.72407054677251	-0.74715268750635	2.36693904402008
H	6.88458476960864	-1.28822465125144	1.89780277128901
C	7.29080625802359	-0.20746865823264	3.71295823128314
C	6.69399812908366	-1.08892474710526	4.62852868063840
C	7.44461517775114	1.13466118548397	4.08594770424305
C	6.25654418977127	-0.64643557205498	5.87559271300101
H	6.56264852119789	-2.13724122827147	4.34763516768863
C	7.00474588215625	1.57924788761501	5.33620796801009
H	7.90386235119471	1.85269657515999	3.40299826377046
C	6.40785732625182	0.69530916233601	6.23338019444065
H	5.79131722070729	-1.35024229373667	6.56785030867690
H	7.13130095600776	2.62941633536599	5.60535714456225
H	6.06227227435206	1.04734615624653	7.20657075420149
H	8.51855384123985	-1.49627263080472	2.50961706014301
C	8.74713478763221	-0.30314029629540	0.08407198701977
O	8.62208228790778	-1.45516824558208	-0.22719668739034
O	9.32284111264336	0.62541906957883	-0.70234518698252
C	4.81231825506134	4.05461769743025	1.64465940212378
H	4.57757623916372	4.93384714983925	2.26605541936272
H	5.22703132552283	3.29792033719464	2.33479229227564
C	3.50435224197992	3.54065280253576	1.03465548274246
H	3.25362722475740	4.14158855129336	0.13895429285863
H	3.60463165511109	2.49508658863474	0.70212963415183
C	2.31372211580927	3.57953449476781	1.98446261634360
H	2.51122174560486	2.90597360107975	2.83561420636735
H	2.22613841808383	4.60074062628379	2.40561985347790
N	1.10883433990179	3.12176396591909	1.30078849978197
H	0.33239300680565	3.07028092889049	1.95914898835330
H	0.83246643353187	3.81043593881579	0.60082919312346
H	8.71714743896377	0.89881641973402	-1.42905089096874
H	9.95437783491524	-4.89395496524432	-2.11588652584646
H	9.85330693746177	-3.40126512671199	-1.16401668378723
C	9.66060182132871	-3.83471160356917	-2.15651076413658
H	11.55975724979507	-3.18223087771149	-2.97439689144975
H	7.53587175977553	-4.14084177377168	-1.65645388205674
C	10.48704875735797	-3.08159994101293	-3.19722151996735
H	10.32517979539203	-3.52886662459715	-4.19446042273053
C	8.16416618048585	-3.73414109577349	-2.46037483681161
H	7.94029494526791	-4.31675983487210	-3.37374058942767
H	10.30085616730533	-1.16046488040572	-2.23852098991347
C	10.09373734265635	-1.60606861670262	-3.22255656118193
C	7.67423232748825	-2.33311056209179	-2.76648445681537
H	10.69434844524099	-1.05429321691297	-3.96072723451839
O	6.53958876619853	-1.99737431236713	-2.50479297031771

C	8.60738681414038	-1.42651565663101	-3.54415286919046
H	8.43166511367402	-1.68766807174097	-4.60485820569724
H	8.26225303193334	-0.39145094055223	-3.40541285341376

93

Entire Intermedia C

C	0.03776461911748	-0.02158236544170	-0.01935709984084
C	-0.01641218915883	0.01572231824491	1.36358367815052
C	1.16754822798134	0.05320461520261	2.09497132645292
C	2.40692147140979	0.04317182896797	1.46876678030644
C	2.47655018697135	0.03293422654482	0.06074141450026
C	1.27726627836741	-0.01249723176675	-0.65207200689543
F	-1.07218724091175	-0.06151998759265	-0.73389192104603
F	-1.18095558982140	0.04541134084624	1.98331346594073
F	1.05559539039367	0.13136173913180	3.41285144820069
I	4.07193569233747	0.11175301452977	2.74765327229164
F	1.25846140452531	-0.04478866979928	-1.98402871871975
C	3.80060086475759	0.08603464552190	-0.67214006522135
O	4.80796505193016	-0.43802552888526	-0.23102711457757
N	3.81393680680826	0.80559029187598	-1.81839826849008
C	5.02885988481835	0.90132769551236	-2.60368930764417
C	4.73254104300936	1.49722305298708	-3.99035626978341
C	4.12310126065673	2.87602175434151	-3.90647452458980
C	2.73541734494626	3.05533331378008	-3.96421315399362
C	2.17019971255023	4.32275106487997	-3.80555317510639
C	2.98983076792314	5.43020535155794	-3.59229831450855
C	4.37641640863297	5.26538438432529	-3.54298428164132
C	4.93700105237425	3.99890900689307	-3.69807747323212
C	6.11552072948144	1.71669312115830	-1.89769566009847
O	7.28523237361847	1.62032872533721	-2.23437144787520
N	5.69101061484225	2.53517582569739	-0.90978215703976
H	2.97160987396818	1.26199591044061	-2.15144823423733
H	5.46815377358943	-0.10144719169143	-2.72085787570511
H	5.68528922050760	1.52710718928612	-4.53707986784335
H	4.06008350201951	0.80948187424207	-4.52304694157612
H	2.08776216043561	2.19174621586833	-4.14422613100292
H	1.08678150806420	4.44352519481199	-3.85618165232490
H	2.55138071112352	6.42227665059914	-3.47320736302559
H	5.02444117690059	6.12970425301003	-3.38712894145270
H	6.02259796339755	3.87173961493986	-3.65981757167664
H	4.69303960823354	2.64718759438772	-0.78592308838440
C	6.59516114508702	3.36340213100805	-0.14745666782348
C	5.87410440603761	4.48581173979866	0.60224478590427
C	7.49566852466389	2.56055443070274	0.80098308690578
O	8.42913165066389	3.11857172619454	1.34957224978300
C	8.23598461812725	0.31040422371927	1.39637068547202
H	6.66466836866577	5.06040993917361	1.10448589759128
H	7.31995721873281	3.81935331509348	-0.84259242494796
H	9.05092253539307	0.90680832878895	1.82884321460967
H	5.41169815293976	5.15587623032813	-0.14132241565401
N	7.20576566922779	1.24658550797849	0.97299591901130
H	6.40135366670234	0.84429383355652	0.49119365885633
C	7.73124923160616	-0.73748356171013	2.36955148551469
H	6.90326283761724	-1.29036268134697	1.89443136254367
C	7.28149923560077	-0.19378114458143	3.70845492203998
C	6.68947507960398	-1.07617598199925	4.62600403089131
C	7.41787388027631	1.15257389043079	4.07328124883595
C	6.24000210851068	-0.63068491636158	5.86766463066675
H	6.57134589495957	-2.12761873704738	4.35092340918161
C	6.96497451308024	1.60019320098635	5.31776607671018
H	7.87307485844577	1.87137660957454	3.38848925641803
C	6.37343065284672	0.71504922995777	6.21729851041570
H	5.77913621600450	-1.33533735524146	6.56200090699961
H	7.07680605310453	2.65365752868543	5.58029423062280
H	6.01808168800363	1.06942483562102	7.18610298165992
H	8.53346228161768	-1.47598733054679	2.52626689006594
C	8.74062892976443	-0.29969831976995	0.09121385878726
O	8.46653995855442	-1.40180495685624	-0.30816828054000
O	9.44479339169201	0.59427645432639	-0.59905492895478
C	4.82487424661851	4.05991855743726	1.63715181155894
H	4.62177955220135	4.93740084918354	2.27229480213361
H	5.24443483531426	3.29020269895886	2.31005750084696
C	3.49065775920235	3.57397515171282	1.06347415365860
H	3.20978068053409	4.20355635160255	0.19772807671574
H	3.56741065918428	2.53849455571904	0.69444276870727
C	2.34597290873948	3.59249364533301	2.06901082379175

H	2.59239419781110	2.91377802295718	2.90271386303175
H	2.26972125894476	4.60909951398099	2.50359278119497
N	1.11179396374225	3.12785930735913	1.44518123882291
H	0.37167521125007	3.06106536109460	2.14273776937776
H	0.79148600260598	3.81814536407280	0.76599976799003
H	9.44347921811521	0.35555463162707	-1.54027899606150
H	9.91841861759274	-4.94021693656104	-2.18481399422639
H	9.81418534769128	-3.47177948861440	-1.19839604868741
C	9.64878946497950	-3.87437081980438	-2.20978776872541
H	11.59291781430731	-3.26199960511879	-2.95640757431972
H	7.49970604566509	-4.13610521467327	-1.80116106771540
C	10.53021406985873	-3.11391162359128	-3.19828549862080
H	10.37538405279573	-3.50950509020517	-4.21776106558961
C	8.17032982856770	-3.73181396082174	-2.57142995485849
H	7.96738025267033	-4.29539443485975	-3.50115999617573
H	10.41212652236226	-1.25967044698650	-2.14808154422245
C	10.19053845782145	-1.62546246987478	-3.16337205146590
C	7.71922109264532	-2.31631469475632	-2.87188552053758
H	10.83260232059481	-1.05840549770445	-3.85319507114733
O	6.56331895570381	-1.99019429306687	-2.72007265527691
C	8.71974100726351	-1.37297974022566	-3.51521628352016
H	8.58656453037713	-1.52938571812469	-4.60183075934120
H	8.38019469150834	-0.34417598625839	-3.30923284721542

93

Entire Intermedia D

C	3.59352283859550	4.21110124571058	0.18759414788991
C	2.99923666782597	4.40588302513422	1.42763516938377
C	1.75784630395547	3.83127051723495	1.69736113629230
C	1.10555966408550	3.05845151091432	0.74340486231668
C	1.70182627822639	2.86105842003158	-0.51159459991399
C	2.94048877041429	3.43820331148444	-0.76821321370725
F	4.77687701492260	4.73956959079598	-0.07106223189868
F	3.61257512660006	5.12435750426936	2.34959995175627
F	1.23786901398476	4.02928978000436	2.89997305574111
I	-0.72474248115416	2.18319534123329	1.28920142496303
F	3.55113149795925	3.24675951954783	-1.93004521920619
C	1.05371444916917	1.97559081488181	-1.55506561660068
O	-0.09042418225583	2.16127385075398	-1.93388620956132
N	1.82990290596662	0.94709011137256	-1.94531557977553
C	1.33372056674582	-0.12724652576469	-2.78714858575106
C	2.43449857515627	-0.64199152814627	-3.72615625788909
C	3.78174499349090	-0.82683543393957	-3.06182811289718
C	4.74116142462244	0.19167709209363	-3.14043613327177
C	5.99051829535239	0.05151220972635	-2.53489348580799
C	6.30113305901954	-1.11658647579966	-1.83711433144948
C	5.35046206947930	-2.13579675998531	-1.74412405353864
C	4.10044608023929	-1.99185052369048	-2.34911715875036
C	0.71265946494138	-1.27636087796995	-1.95033229835215
O	1.03394069324910	-2.44081704568812	-2.11150674855145
N	-0.20868611147565	-0.90077930500274	-1.03260121655717
H	2.71841231687098	0.81949172304133	-1.44165087636945
H	0.51145723332009	0.29185338963695	-3.38776650514391
H	2.08806379802419	-1.58974997997452	-4.15682787313568
H	2.54149778578491	0.08616815018754	-4.54159878964656
H	4.50363874054807	1.10867058915219	-3.68542199431733
H	6.72371021720528	0.85596655232305	-2.61270920204278
H	7.27963559688786	-1.23346249489859	-1.36889494838669
H	5.58553261931035	-3.05385048286739	-1.20274722380667
H	3.35492554800213	-2.78528661252053	-2.27709312031508
H	-0.47322048710720	0.07918357330332	-0.96215342287067
C	-0.70868585029550	-1.84836287712316	-0.04503614230090
C	0.32948848593279	-2.36716366474754	0.97336464600832
C	-2.02338079413277	-1.29622491680826	0.52079715954581
O	-2.95641755104576	-1.03847743858040	-0.22321901728738
C	-3.36106072622028	-0.88808476292483	2.53384680320524
H	-0.21308255527747	-3.02205497660004	1.67505794118609
H	-1.03688173245819	-2.73804753371205	-0.60855628972023
H	-3.98106971578816	-1.79908805951007	2.53746719574307
H	0.99838249865839	-3.03276223136881	0.40808476504201
N	-2.10399935736019	-1.13769924484330	1.85903682738674
H	-1.31575928638325	-1.40222952769274	2.44004302554115
C	-3.09424033215539	-0.43065682994185	3.97978739966597
H	-2.64720809782630	0.57454354349608	3.95297821993969
C	-2.17791700737747	-1.37923583622246	4.71309730687661
C	-0.84484710618640	-1.02949385643102	4.95823346681250

C	-2.62615178540480	-2.64776472749161	5.10531914469692
C	0.02539223711788	-1.92791585308717	5.58094800454426
H	-0.48788078384998	-0.03848466526739	4.66211897959892
C	-1.76066715816553	-3.54467142221773	5.72825142827840
H	-3.66546515189314	-2.93122153689007	4.92216502738895
C	-0.43094768924786	-3.18740986529234	5.96597174286961
H	1.06103036047069	-1.63797209109282	5.76891161349605
H	-2.12467087671883	-4.52698218629433	6.03245648333650
H	0.24562381058835	-3.89008106841404	6.45474443304997
H	-4.06606963835894	-0.35441772788181	4.48871686871892
C	-4.20512376151288	0.16830286851474	1.83144481376224
O	-5.37901456090379	0.03616705740478	1.62721338942434
O	-3.51761496816711	1.28065151701461	1.56843292616679
C	1.21234936958448	-1.37411454210644	1.74677761003609
H	1.80596327202703	-1.95791982234004	2.46997695987404
H	0.61411375513617	-0.66289698653445	2.34314911308164
C	2.13342158291865	-0.60263384939573	0.81211561563079
H	2.53158563678982	-1.29836256313492	0.04872156647225
H	1.53238325638314	0.13053339747207	0.27218649340546
C	3.31171572570002	0.16150982086774	1.39163648409283
H	2.94790412485082	1.00933323196095	1.99659258451977
H	3.90842963248959	-0.48282004953609	2.06556684694354
N	4.08082822739465	0.68855513225116	0.25953300273295
H	4.78766525215381	1.35440295459346	0.56881252754858
H	4.59286657281354	-0.07344891799790	-0.18993088312380
C	-3.66873368559944	0.23807148894460	-3.77821089574707
C	-5.16053879776444	0.26879892601823	-3.44826887300796
C	-5.39053320514992	0.49516786623719	-1.95155392571352
C	-4.58011138243948	1.62052639582313	-1.36137513174535
C	-3.16572733743408	1.78598538653727	-1.85640818738953
C	-3.00705576728626	1.54966453182643	-3.36045303268907
H	-6.44506859857703	0.65173126431452	-1.68993419207693
H	-5.63874662681427	1.08045977596489	-4.02136634489053
H	-5.65008188489008	-0.66691998975139	-3.75461917040734
H	-3.19150055506276	-0.59915577292220	-3.23916083925859
H	-3.52114778080332	0.05978047520752	-4.85408472514013
H	-2.78146648500862	2.76224625761311	-1.53273869504907
H	-2.59481064177891	1.01031976191531	-1.31453171237189
H	-3.46992832809865	2.38189031987903	-3.91711949707232
H	-1.93518254893771	1.55829298432102	-3.60204449112144
H	-5.03653476672860	-0.38916044970885	-1.38900338967533
O	-5.01842401025235	2.29566087138564	-0.44518634733440
H	-4.08181729951377	1.89088990126468	1.03705184839000

93

Entire TS3

C	3.80848689871214	4.13431425770040	0.38771781800369
C	3.67843628380766	4.08993830880439	1.76971536521537
C	2.60852208172325	3.40326563834783	2.34287283870918
C	1.66767551410825	2.75716968102740	1.54701580033918
C	1.79835240058205	2.78961403795010	0.15432828603508
C	2.86965468784008	3.47990771975379	-0.40485549187524
F	4.81303860654680	4.78747467345221	-0.16499875685483
F	4.56284126249972	4.69814396925335	2.53686997030449
F	2.53155582040480	3.38201886650551	3.66418727884799
I	0.14667964313652	1.71749656591486	2.54697314593373
F	3.02778976321903	3.53578097856958	-1.72154410006635
C	0.86007586978081	2.06054505873788	-0.78375057663724
O	-0.32927835872912	2.31924013887354	-0.86321991475613
N	1.49066058128525	1.11427370913916	-1.50162435673728
C	0.85527148721470	0.35371652896604	-2.55959464126368
C	1.85875651951720	0.06591379266632	-3.68690682867774
C	3.21310215896748	-0.39752389949576	-3.19551639162709
C	4.25334396517614	0.52986247983444	-3.04356033207947
C	5.50739625214571	0.13279193816109	-2.57685005794756
C	5.73988207811458	-1.20421289085315	-2.25116020919670
C	4.70862888462090	-2.13611274274089	-2.38998201834144
C	3.45497428860130	-1.73668912823754	-2.85653117341036
C	0.18195155002689	-0.93869097809695	-2.04999531649305
O	0.35414282211937	-2.01610924431075	-2.59203467485802
N	-0.63699638846425	-0.78744807718803	-0.98210789545699
H	2.42287293843589	0.83428208766395	-1.17529569837080
H	0.03815350721943	0.97909591900253	-2.95304502957460
H	1.41050592274377	-0.68896241859693	-4.34503331322705
H	1.98775230958466	0.99254393177336	-4.26217374942262
H	4.07602654117806	1.57834311254246	-3.29499166866329

H	6.30529386627722	0.87002125771274	-2.47205026923961
H	6.72087182063067	-1.51991462067819	-1.89204196717522
H	4.88271436401392	-3.18423446490136	-2.13989815482319
H	2.64679262522052	-2.46185596707795	-2.96391292027177
H	-0.74194202184222	0.13259416792369	-0.55774689087070
C	-1.20506115033705	-1.96229286375665	-0.33476103826081
C	-0.21031526568760	-2.81200665916274	0.49618403963403
C	-2.49066386584657	-1.56770620395284	0.39364573365752
O	-3.58499571797734	-1.77381223611394	-0.10307849110651
C	-3.51672922217145	-0.78029096351582	2.45666701723688
H	-0.81304544094784	-3.53952383167097	1.06374908897132
H	-1.56074013784951	-2.61635943621256	-1.14171731006801
H	-3.92588092928491	-1.75395504817323	2.77198278144142
H	0.36066966482940	-3.39813897526393	-0.24018403621275
N	-2.35777654767737	-0.97899508143912	1.60606091044726
H	-1.43439704350137	-0.90443395053400	2.01807739570190
C	-3.16889639778128	0.06812412594183	3.68055013770039
H	-2.95944275636681	1.09839028526490	3.34852938487010
C	-2.01065924916241	-0.43475286331412	4.51691159722129
C	-1.36021187268232	0.45926179359039	5.38002726441662
C	-1.57214672446082	-1.76528150326020	4.48236454200628
C	-0.29162965663633	0.04344437114128	6.17244594017930
H	-1.69657199282497	1.49875574035162	5.42458595552076
C	-0.49807665120565	-2.18259522298378	5.27099831140385
H	-2.06355177914106	-2.48963317289159	3.82873419101100
C	0.14809441620598	-1.28005988834543	6.11477214532894
H	0.20331751314802	0.75809313853544	6.83212271843358
H	-0.16655193495728	-3.22128662017970	5.22309006900512
H	0.98923423053634	-1.60670727813579	6.72800237351056
H	-4.07567253345440	0.12359119241162	4.30423187552586
C	-4.66097714251952	-0.09709995783477	1.71240308805345
O	-5.80766826988866	-0.42709912194302	1.84282898578072
O	-4.24990217920526	0.93896168829247	0.99362535659106
C	0.81957494105100	-2.15047067790328	1.42812406869018
H	1.38549921198486	-2.96992130106864	1.90223825955457
H	0.35115310351726	-1.60870087302775	2.27035217174073
C	1.77735865943544	-1.22379424338307	0.68811656053866
H	2.06313710307834	-1.68908136199521	-0.27478777031038
H	1.25037320598398	-0.30140595145657	0.43706448397527
C	3.05080525041254	-0.78903382269673	1.39324954493371
H	2.79374552871556	-0.20897695664393	2.29577639367050
H	3.63207193694673	-1.66816511436075	1.73235211697790
N	3.79329561041071	0.07911469717321	0.47329802006126
H	4.55561299325907	0.55236710826457	0.95604175799873
H	4.23623067627168	-0.49184119921210	-0.24962744219065
C	-2.74869992202447	0.30822031759977	-4.11577819095706
C	-4.15256123676038	-0.26047009637262	-4.32266876761812
C	-4.84627808864817	-0.54711969268911	-2.98598898974183
C	-4.76834210496459	0.59458647968385	-2.00229370468136
C	-3.43769690443616	1.30336149574146	-1.90802353206807
C	-2.81453053385199	1.58335541822301	-3.27766986911244
H	-5.89919422788412	-0.83532709958940	-3.10319049080224
H	-4.75083130190068	0.46698222035350	-4.89591513306678
H	-4.11704327442460	-1.18268007085669	-4.91990747767332
H	-2.12292217374906	-0.44320396438345	-3.59975976986108
H	-2.27377828898627	0.50961513085966	-5.08773843144288
H	-3.54772367915226	2.21156583511505	-1.30138735712092
H	-2.77683010065065	0.61308499313163	-1.35702817933277
H	-3.41625494572888	2.33682986290096	-3.81266413858596
H	-1.81950910453253	2.02504864221647	-3.12218656305191
H	-4.32936210605706	-1.37514862174224	-2.46656916380931
O	-5.70318713528817	0.87987643974174	-1.27795824147712
H	-4.97785100159825	1.21814656143214	0.39242170129093

93

Entire Intermedia E

C	2.53562476831045	4.55790298811079	-1.10307664040664
C	2.37390491504191	4.87368767312799	0.23891539180995
C	1.37031137645506	4.24801870758658	0.97711535128237
C	0.52792126630317	3.31248555554442	0.38550370867806
C	0.69105490713730	2.97477725441580	-0.96394851502828
C	1.69474936342182	3.61456214033932	-1.68918402823530
F	3.47546155472168	5.15075630333356	-1.81437940972783
F	3.15992016730743	5.76573489715689	0.80907043855484
F	1.24947288341766	4.58049164434891	2.25276711579271
I	-0.93615467053039	2.47518675880855	1.62173660366920

F	1.87821849228806	3.35684955012023	-2.97818625513984
C	-0.15776444817100	1.94728524602483	-1.68727793202431
O	-1.37392965475391	2.03910238299433	-1.76276069836267
N	0.57567866116270	0.97392905726623	-2.24922955541060
C	0.05029144119983	-0.01600740999191	-3.17366296716387
C	1.12722878508431	-0.38055910688899	-4.21086933075093
C	2.50531512427393	-0.57990941888895	-3.61525244652150
C	3.40970044564713	0.49127058825531	-3.58898403697077
C	4.67324805114285	0.35242183749307	-3.01282490984644
C	5.05621209821308	-0.86870695873689	-2.45652735295897
C	4.16509480368103	-1.94394875700392	-2.47824375087171
C	2.89941165466188	-1.80127466819703	-3.05004584435626
C	-0.49968025019223	-1.27294631010966	-2.46132056238073
O	-0.10445208944714	-2.39427242554170	-2.72860720767334
N	-1.44222802675415	-1.02991370658486	-1.52516816511619
H	1.52482029407433	0.85249083598550	-1.88015526072415
H	-0.80727643442143	0.45237192378691	-3.68268629630650
H	0.79763153593757	-1.29031192003545	-4.72785049899986
H	1.17432689324091	0.43599322123287	-4.94405933433185
H	3.11466112770998	1.45011624728637	-4.02118520563277
H	5.36117218370092	1.19982626061588	-3.00469687024032
H	6.04573083683923	-0.98382508102092	-2.01078699100026
H	4.45824561086452	-2.90430436163305	-2.05021130801856
H	2.19883002098140	-2.63694170029004	-3.06030759927394
H	-1.82616641132955	-0.08762267135410	-1.46208231771294
C	-1.93318836068523	-2.09693338947780	-0.66057724681479
C	-0.86059242525976	-2.74996576597529	0.24893204004929
C	-3.11153580202014	-1.61929017093008	0.17916398892986
O	-4.08101412865183	-2.33141291682252	0.36015038241915
C	-4.00011981392968	0.19879782200888	1.56652381461394
H	-1.40671258282245	-3.46801801449361	0.88143274122396
H	-2.34215977625367	-2.90177859636901	-1.28821331785731
H	-4.84072970137947	-0.51037375195834	1.51306173263787
H	-0.20545740454381	-3.33913404094790	-0.40908204452812
N	-2.96931853322282	-0.39320850911439	0.74457220848796
H	-2.10535192380439	0.11963252404742	0.60144853919462
C	-3.60111233798978	0.42804597003421	3.03549611876163
H	-3.13600381903760	1.41965958184975	3.14528781502298
C	-2.69072967166909	-0.62977841718260	3.61008997350082
C	-1.55699309649645	-0.25892250349284	4.34194157039962
C	-2.94307335066299	-1.99474461246356	3.41304852450392
C	-0.69083911639571	-1.22240849308756	4.86340650416326
H	-1.34677957712997	0.80122443143439	4.50757029617821
C	-2.07860129411432	-2.95868494558500	3.92982852681622
H	-3.81063103355833	-2.30848536624702	2.82753305972237
C	-0.94777397321768	-2.57634018390172	4.65524893470370
H	0.18838193622774	-0.91070123868407	5.43001902700421
H	-2.28785505107239	-4.01667862790901	3.76068808936246
H	-0.27046503778399	-3.33255294721166	5.05562233884408
H	-4.53136777626251	0.49465449769251	3.62150779530414
C	-4.50394407502021	1.49826093198559	0.92934347469219
O	-4.66371039205487	2.52111781062470	1.55296464024007
O	-4.73815209498117	1.36840174453912	-0.35882959191676
C	0.01325733065927	-1.85067284309127	1.13571394003810
H	0.66357686446381	-2.51868665403998	1.72561856410881
H	-0.60098464006452	-1.30641160059121	1.87414836748833
C	0.87280841459412	-0.88039525769989	0.33044097608266
H	1.25847475986799	-1.40117324873430	-0.56625093365298
H	0.24342552651472	-0.06564739869777	-0.04179650232333
C	2.06072995556073	-0.22231315810143	1.01276462131762
H	1.70598686381692	0.47606860096466	1.78950565779468
H	2.68348727712538	-0.98069090073243	1.52521294489529
N	2.79666752389990	0.54140997094752	-0.00243642580655
H	3.47922975137879	1.15985672627506	0.43333839081921
H	3.33780504717518	-0.10129772826451	-0.58517201211382
H	-5.04201949800621	2.24941779197035	-0.73527874591690
H	-1.99879831642781	4.91399369691550	-2.22353146157211
H	-1.30677744118333	5.21492877786422	-0.62669540792111
H	-1.89751993765646	7.36263156042482	-1.79020368216738
C	-2.23239769217200	5.29842176660349	-1.21688189583052
C	-2.68294027211875	6.75412110595433	-1.31851249000760
H	-3.80573507986853	6.49153886493682	-3.14206806028183
H	-3.03717473374925	3.34753846293027	-0.66381870056581
C	-3.30756799574721	4.40840077308341	-0.58345636197797
H	-2.84176606084538	7.16331497006300	-0.30565845459970
C	-3.98061849794049	6.85839050541215	-2.11734031873003

H	-4.30643956205943	7.90448582766534	-2.20512161638615
H	-3.41289379591600	4.64490959060783	0.49121197522769
C	-4.67981712346926	4.61367458514308	-1.17197457896924
O	-5.45279125632370	3.68765210681941	-1.35363970191331
C	-5.09680223456493	6.03229647874772	-1.46831942238419
H	-6.01647620828403	6.00652756984366	-2.06709042648228
H	-5.34953603208722	6.47320665686068	-0.48675249242899

93

Entire TS4

C	2.98619137278893	4.26873428276219	-0.63026309444310
C	2.66431715257905	4.61088435237172	0.67596770445897
C	1.45648134314942	4.18066552554836	1.22103603079658
C	0.56858822647767	3.41329328822885	0.47459499566688
C	0.89349212634271	3.04052987936318	-0.83764878233036
C	2.10136002091029	3.48925636588253	-1.37033132190875
F	4.12112759296936	4.68290705653062	-1.16298461284750
F	3.49089858152315	5.34833168734022	1.39198757684907
F	1.18511648290322	4.53801951558743	2.46684340499521
I	-1.24327143381657	2.91982787926459	1.39863239555012
F	2.45535390985590	3.19322747925070	-2.61448563637910
C	0.00805764506542	2.14848098135635	-1.68840501759329
O	-1.17729082106940	2.37824026833637	-1.87074250830938
N	0.66936799570965	1.09864394500907	-2.20557908690273
C	0.09193208862005	0.18152043785213	-3.17097381820388
C	1.14549385501422	-0.22269048432557	-4.21670192068376
C	2.49765857308449	-0.56713135983187	-3.62674426662991
C	3.51477703758961	0.39699387468053	-3.61568455541537
C	4.76337708300109	0.12151303619328	-3.05580945273464
C	5.01729778399745	-1.13219623078013	-2.49809938206050
C	4.01263093816670	-2.10250742654737	-2.50350320003151
C	2.76351568506728	-1.82328639840021	-3.06083565197767
C	-0.54887846291865	-1.06177351689238	-2.51203949547419
O	-0.27620486871707	-2.19324437037940	-2.87428624198817
N	-1.42458475330756	-0.81164036341892	-1.51360128340141
H	1.58827460661550	0.88598596656229	-1.80083941476235
H	-0.72822064685346	0.72448328879912	-3.66703295418544
H	0.74486212710100	-1.07539858421715	-4.77900738150937
H	1.26791363697443	0.62050324932752	-4.90985904337121
H	3.32359694483571	1.37904093784753	-4.05274725236153
H	5.54042707164483	0.88793537449849	-3.06158745962777
H	5.99408919428601	-1.35438774818649	-2.06505675335564
H	4.20342520679182	-3.08843167671167	-2.07570563578761
H	1.97819498143174	-2.57977957652013	-3.06276180256995
H	-1.71789505816647	0.14911539597754	-1.34788794247035
C	-1.94551312310817	-1.91126808240453	-0.70813631013408
C	-0.87612115763577	-2.65863994849429	0.12763167276599
C	-3.09364066050513	-1.46960718163653	0.18959059752680
O	-4.06345333931037	-2.18456556995971	0.35147828684769
C	-3.92100651437484	0.22485524098259	1.75914504354136
H	-1.42572928468750	-3.40700144917513	0.72063772591911
H	-2.39170215646196	-2.65417235794846	-1.38404724601991
H	-4.73621589832666	-0.51173006165907	1.73166127626730
H	-0.25100660466657	-3.21424592964239	-0.58673101511288
N	-2.93073867724566	-0.27977764040581	0.82758989373595
H	-2.04016027374746	0.20402752559855	0.75981888601098
C	-3.40236993736360	0.40297272024715	3.19086110695342
H	-2.87446227339683	1.36260813415380	3.27906408777888
C	-2.49721168876605	-0.70763802914101	3.66501513449481
C	-1.31112060608139	-0.39908018936307	4.34101825014547
C	-2.80324690796660	-2.05474598137710	3.42703966828418
C	-0.45118027036106	-1.40850978209597	4.77803455734916
H	-1.05563386235978	0.64860545203334	4.52029354192224
C	-1.94308904487437	-3.06429370695901	3.85739987399998
H	-3.70985702481168	-2.31955321081709	2.87738065140154
C	-0.76450475283268	-2.74510579743213	4.53543348703086
H	0.46903369385793	-1.14716168803859	5.30366975112004
H	-2.19397382614861	-4.10792543292382	3.65848981870850
H	-0.09202697617140	-3.53692321787310	4.86970546607774
H	-4.28095821449638	0.49243648812103	3.85002773369333
C	-4.49555359847030	1.53150908000887	1.20351560321235
O	-4.34443160446097	2.60828455171733	1.74001899922686
O	-5.12672738871569	1.34802683719774	0.06910154257749
C	0.03876172105888	-1.84222622866812	1.05155276692609
H	0.69080321984223	-2.56330652771896	1.57297693362547
H	-0.54304826776248	-1.34342626146360	1.84457987462841

C	0.89874858854612	-0.83330749987929	0.29371907755291
H	1.21264114170309	-1.28728939265341	-0.66509800793681
H	0.29553867124107	0.04376782317979	0.02932663249382
C	2.15816641103195	-0.30412264265929	0.96165018234145
H	1.89001151264685	0.36182565703013	1.79849961511315
H	2.74631054543886	-1.13708199831568	1.39239603338871
N	2.90601968486418	0.47018465691514	-0.03720810265201
H	3.62834351104007	1.03537642960206	0.40657700151279
H	3.40181865405672	-0.17093529999760	-0.66072179936779
H	-5.46159440694497	2.22150147945934	-0.30460766636511
H	-2.87431645880366	4.66669279494905	-2.81510027047990
H	-1.80399505864061	5.09612029204560	-1.48413211714043
H	-2.68620111148137	7.14210922793017	-2.67007536293017
C	-2.84777320679115	5.15213078120510	-1.82623166324848
C	-3.32139189481883	6.59757235994934	-1.95622071308230
H	-4.86546530923834	6.16729493568858	-3.40022147218915
H	-3.46542258526112	3.28700240168776	-0.86591388550888
C	-3.72188709692992	4.35497170965760	-0.85400498188718
H	-3.22284017435145	7.10984108669623	-0.98330038163286
C	-4.77982450000353	6.64249532634148	-2.40916193382686
H	-5.12662305844963	7.67931185616188	-2.52232447661673
H	-3.55260351345113	4.71280544365890	0.17821965440304
C	-5.20061639497825	4.52476548639725	-1.07766056491831
O	-5.99021851396347	3.60725236827926	-0.92340640915534
C	-5.68984810873601	5.91040021826339	-1.41666401252910
H	-6.73093275748307	5.83777464127443	-1.75736965029322
H	-5.69237616771425	6.45872274888441	-0.45685536900344

93

Entire Intermedia F

C	3.49598766408288	3.97453758096414	0.58832590013353
C	3.04734788495052	4.21009446306858	1.88126101666241
C	1.69088783658715	4.07821686410850	2.17619094290504
C	0.77897281974169	3.71820612528979	1.19054017862457
C	1.22552452706066	3.48394786261285	-0.11683343555498
C	2.58163311803357	3.61024012702438	-0.39601644075242
F	4.78132870288697	4.08437588388195	0.30104781296304
F	3.90346730586855	4.54838431749966	2.82746321555242
F	1.31002853217749	4.29346927629274	3.42666579141398
I	-1.22644131422465	3.46870095614670	1.75676472124073
F	3.04988989976317	3.37577601108254	-1.61520719411380
C	0.27055222909852	3.08342079013524	-1.22359092290506
O	-0.67080374274039	3.78948249765040	-1.54038570477108
N	0.55917218576084	1.88698622129774	-1.77243328347829
C	-0.17116273691770	1.33242250973887	-2.89592301231408
C	0.75780947989567	1.04201313892768	-4.08703742157371
C	2.03398001702840	0.31857684762150	-3.71413076562826
C	3.23144092024859	1.03641981393462	-3.60161446705306
C	4.42197847845152	0.40113804356672	-3.24483820286376
C	4.43183964491045	-0.97104361936809	-2.98992669703270
C	3.24414364197457	-1.69864046419266	-3.09655546767246
C	2.05582793985101	-1.06094662687821	-3.45723574108246
C	-0.99751954833089	0.08097431660033	-2.51393113108210
O	-1.05286157440151	-0.89069708302371	-3.24641209121468
N	-1.67644459670166	0.14184500323001	-1.34317213670577
H	1.37520295646843	1.38631121719951	-1.39553342816856
H	-0.90359200748432	2.09983542763365	-3.19594285613539
H	0.18471052229457	0.45797097385056	-4.81822797163135
H	1.01846110903607	2.00553334207616	-4.54534906017085
H	3.23013543678182	2.11013081707121	-3.79956392509054
H	5.34330809712751	0.98112024661765	-3.16765598489046
H	5.36088838033618	-1.47262983470081	-2.71369060446388
H	3.24342940558798	-2.77348782999761	-2.90645375394639
H	1.13002534871319	-1.63126925301572	-3.54743951391891
H	-1.57538311369798	0.97641462584235	-0.77580130467697
C	-2.28893989682965	-1.06411003782350	-0.79409323388062
C	-1.28071456942285	-2.13593750760918	-0.31380204919889
C	-3.31250088271306	-0.78456573983570	0.30169662330651
O	-4.30316834449710	-1.48095820919140	0.39791772601043
C	-3.90579164322715	0.52763777432340	2.29685303290596
H	-1.88461994260478	-2.96047000630174	0.09613623807218
H	-2.87400484477616	-1.52421693514738	-1.60112149842093
H	-4.71241085359767	-0.21436258837272	2.26155100731128
H	-0.78104987971399	-2.52452742489875	-1.21480534666805
N	-3.03366444064851	0.22687901674325	1.17094004693103
H	-2.11819874336035	0.66739705982955	1.16937794030891

C	-3.16602210964434	0.50620225840841	3.63361499748162
H	-2.55485946220736	1.41651274440113	3.71898551347558
C	-2.28908342665650	-0.71040330836194	3.81444558826975
C	-0.99511681368295	-0.56969546267204	4.32855322268217
C	-2.73361501655619	-1.99143823503245	3.45760029177702
C	-0.16644866052186	-1.68091324514800	4.49863234790322
H	-0.63302176065256	0.42607092662261	4.59724585761214
C	-1.90699864186951	-3.10209440411002	3.62365493101483
H	-3.72887437825787	-2.12409762201164	3.02610873934952
C	-0.62118837686844	-2.95115896135493	4.14741374897941
H	0.83886727997816	-1.55060020462781	4.90339155397577
H	-2.26860162072699	-4.09158966055280	3.33816209663258
H	0.02497445884915	-3.82117387183149	4.27536499042606
H	-3.91945265325089	0.56392047265229	4.43594827654643
C	-4.50935190309385	1.89748061921576	1.99541966487206
O	-3.97878409750615	2.93892700930897	2.32140680305944
O	-5.58985498883518	1.82737350857327	1.25557678371910
C	-0.21181868081247	-1.71506297647018	0.70205267634868
H	0.37023516907086	-2.61753911830317	0.95351237417112
H	-0.67337094267857	-1.38447436432194	1.64734733312703
C	0.72302085738699	-0.64260598827466	0.14953374165073
H	0.87587522267194	-0.83669441429159	-0.92870137190692
H	0.24782958936016	0.34497478171005	0.21670438422358
C	2.10748470758602	-0.48493579114866	0.75716136034413
H	2.03122616521341	-0.06466306476654	1.77269347377935
H	2.60382720730428	-1.46927613665836	0.85282364698773
N	2.85115968757895	0.45305740960574	-0.09284921604528
H	3.67321312348534	0.81619384095231	0.38726943936143
H	3.20721812654059	-0.04103976459542	-0.91379979833051
H	-5.74498434317110	2.72281637145695	0.82515172297996
H	-4.94803675500802	2.35500965875972	-3.17605430664837
H	-3.29600184153444	1.73496088457321	-3.11016671082278
H	-3.48460780155237	3.69409632621391	-4.68408218120366
C	-3.92152565964059	2.60529325483204	-2.86104871219147
C	-3.44796044747310	3.85777454498866	-3.59684604505769
H	-5.34430825470986	4.89528022340639	-3.55533306720804
H	-4.27799635531806	1.93475516754653	-0.79951891774924
C	-3.89584399237562	2.81277657636189	-1.33649136382489
H	-2.39860608070197	4.05847430500767	-3.32436795231972
C	-4.30768824643339	5.06379857569565	-3.21986296639783
H	-3.95189530479889	5.97248960250664	-3.72550753266862
H	-2.85068990585401	3.00666378036435	-1.03249938095797
C	-4.68386036147218	4.04228970287804	-0.95854433194665
O	-5.57447489724246	4.04075041443508	-0.12445218878791
C	-4.30482695888581	5.29699428324278	-1.70019025527330
H	-4.98051436965639	6.10659855084623	-1.39656852546636
H	-3.27987089420333	5.55126080846200	-1.37699828322472

93

Entire TS5

C	2.24155729771899	4.14267463271105	-0.89240868201129
C	2.40423593158092	4.26566861156418	0.48033106145883
C	1.61038943257337	3.50697106868697	1.33878107861090
C	0.65298869453583	2.62938079932283	0.84049469417905
C	0.48828731766102	2.49164599726096	-0.54368072903753
C	1.28966108835621	3.25603853135648	-1.38968260830062
F	2.98103242404144	4.86310357254842	-1.71511480898574
F	3.30085117467398	5.10037458252507	0.96973745713878
F	1.80718721712762	3.65313887877734	2.64028558021439
I	-0.44776036864036	1.56388994963161	2.27081862745269
F	1.16434502895033	3.17605285735259	-2.70908896383410
C	-0.50848096287833	1.54055921301896	-1.17468718856040
O	-1.70437371131468	1.58968769672872	-0.93674663850480
N	0.07209537490586	0.67400360595347	-2.02333834585368
C	-0.65938086305841	-0.24512561504595	-2.87601561202584
C	0.12839812606454	-0.49384349967534	-4.17327830687738
C	1.60879159624563	-0.72217120158242	-3.94975316451227
C	2.49827829064473	0.35846730026658	-4.03424539970159
C	3.86275329069805	0.18728651518928	-3.79534437961700
C	4.36247650048762	-1.07543142618212	-3.47477937966698
C	3.48670910992204	-2.15989513581409	-3.38830877581450
C	2.12121534901620	-1.98528564108060	-3.61981606540916
C	-1.00675849279860	-1.56664875281717	-2.15889881004928
O	-0.69757275441664	-2.65802174717472	-2.60972078137173
N	-1.67310403889369	-1.42093314282833	-0.99491202373374
H	1.08353536752046	0.54883585969510	-1.92358751674099

H	-1.62240259224927	0.23698601862821	-3.11129097475627
H	-0.32113643263414	-1.35935796703159	-4.67578322368379
H	-0.00157588030230	0.38606530540250	-4.81771219861442
H	2.11286872690651	1.34974692267722	-4.28424753214180
H	4.53690299401382	1.04256066624795	-3.86755776403336
H	5.42978621228612	-1.21514376381938	-3.29501962255945
H	3.86903740658065	-3.15204751786118	-3.14147697198274
H	1.43391318638801	-2.82808623563915	-3.54049440133911
H	-2.10549879667559	-0.51754410005091	-0.80292641118982
C	-1.98497972507763	-2.58903966479491	-0.19047171800226
C	-0.76116999440210	-3.24283680592954	0.49998190656020
C	-3.09793677480070	-2.30510898536742	0.80799688725934
O	-3.99287614971051	-3.11933976691455	0.97763140160725
C	-4.10648767169152	-0.86096992220232	2.46569774368970
H	-1.16627061696324	-4.00075491947117	1.18909769124176
H	-2.40586285600140	-3.36123906939467	-0.85140279935590
H	-4.35784580467426	-1.78423684607877	3.00474406286305
H	-0.21722425721031	-3.79162907994450	-0.28342387113586
N	-3.03765353912943	-1.15872812728557	1.52641155623663
H	-2.32265547735364	-0.46056110137225	1.33914488681744
C	-3.73322351063911	0.25028745394316	3.43947495775493
H	-3.55676488621386	1.17825650631769	2.86940385525157
C	-2.54665412256224	-0.03171357071975	4.33761587416824
C	-2.07200050839270	1.00018384459144	5.16308753680155
C	-1.90450557551992	-1.27528139506112	4.39162035217180
C	-0.98005185988859	0.80218586967869	6.00568749104513
H	-2.56550342566611	1.97529945821021	5.13332126702893
C	-0.80539583903290	-1.47335724357403	5.23108536034270
H	-2.25342005906728	-2.10149525085561	3.77023652874093
C	-0.33692289225420	-0.43700311584977	6.03674627135762
H	-0.62544116875138	1.62078496615575	6.63409524727745
H	-0.31406748795442	-2.44766059203062	5.25137070959751
H	0.52379174848965	-0.59323148371465	6.68872602843223
H	-4.61705428538346	0.44555185908283	4.06765120174932
C	-5.35804017760940	-0.45804824280632	1.68354718929005
O	-6.43652950418557	-0.94503834807268	1.88226955694902
O	-5.12764570757011	0.51007552053348	0.78494059943248
C	0.23866798213942	-2.33734408570171	1.23312951483155
H	0.95721055009435	-2.99700164394017	1.74802914424692
H	-0.26384658270542	-1.75562649468681	2.02661843365914
C	0.98181609600717	-1.39899791104427	0.28316813841487
H	1.15437156843523	-1.92135614558126	-0.67702447293009
H	0.33401495774889	-0.55047380266491	0.05291242457291
C	2.31251824364038	-0.81046484188290	0.72072613758013
H	2.16695545881403	-0.16748008783027	1.60478050082673
H	3.01303434931703	-1.61385450814594	1.02169559897599
N	2.81432529360515	0.01622315944900	-0.38273038139914
H	3.64188250632270	0.53748510645870	-0.09694958674098
H	3.12145629622433	-0.59053417851670	-1.14660168072661
H	-5.03197638675923	0.11782863797111	-0.12859412586064
H	-6.37313586590555	-3.49292437387521	0.27957405633586
H	-6.66604135915896	-5.47691897709184	-1.21367971391858
H	-4.41531293279862	-4.46377396507208	-1.05714318264892
H	-8.00311444470346	-3.50267020246487	-0.42110682541652
C	-6.93587257086542	-3.39834651564129	-0.66188164846880
C	-6.48486883073139	-4.48029883997471	-1.64202118471823
C	-5.00202256742548	-4.32325162298611	-1.97778093619068
H	-4.67751625154742	-5.08500236414025	-2.70076305048222
H	-7.08511074281151	-4.41494016570876	-2.56654677491637
H	-6.95046914128304	-1.20425520325504	-0.52639637704970
C	-6.68662412315073	-1.99693473236329	-1.23859269157737
C	-5.25541203373158	-1.83427244929135	-1.67543428539471
O	-4.58146449453087	-0.86327288923898	-1.36346329387832
C	-4.69978465581414	-2.92773546529854	-2.54993741155371
H	-3.62490352742110	-2.75693604026818	-2.70712121991000
H	-7.29922847334422	-1.86514432608168	-2.14864355510159
H	-5.20536681311421	-2.82280750037664	-3.52677159213509

93

Entire Intermedia G

C	3.20340291671505	3.87889582622711	-0.17191430399209
C	3.21574896681643	3.93943901211027	1.21562660561761
C	2.16929867900117	3.37374443494859	1.94388482503229
C	1.09972162106572	2.76573445542682	1.29612145323795
C	1.07511761638775	2.71047210861417	-0.10322074501055
C	2.13440971420974	3.26189759095845	-0.81811111226794

F	4.19327318890267	4.40867398039552	-0.86690703847728
F	4.21839638442187	4.52543076522208	1.84192016965519
F	2.24010569717786	3.43180361680190	3.26440425864872
I	-0.36459400323850	1.86505950010248	2.49890153516933
F	2.15324038085905	3.22477403287123	-2.14464070214627
C	-0.09187765277987	2.12601675570763	-0.87274954775634
O	-1.21879075238182	2.56619368203220	-0.73701446185868
N	0.24996622713988	1.11513725312509	-1.69612685005560
C	-0.68515357436483	0.58295896588623	-2.66536204349163
C	-0.03132559828150	0.41525484429182	-4.04458919020323
C	1.29352739523666	-0.31108603739047	-4.00187612760486
C	2.49159362975176	0.40176556866391	-4.13263256543132
C	3.72483870793855	-0.24796433368540	-4.06637086538306
C	3.77788776547707	-1.62655647529048	-3.85577920766395
C	2.58961109993484	-2.34772493740101	-3.71773553755109
C	1.35765919309176	-1.69685822901595	-3.79524608916958
C	-1.38195076270431	-0.71033642059683	-2.20527349696084
O	-1.80374344856050	-1.52103593146504	-3.00895633828077
N	-1.53311956613517	-0.83739876553309	-0.86169168750841
H	1.22093838368423	0.74076762406113	-1.63247268618645
H	-1.50518092007995	1.31498439843595	-2.74812333469962
H	-0.74701796408955	-0.12323084598174	-4.67965715815345
H	0.11843512252009	1.41682697343161	-4.46951792945581
H	2.45545524339356	1.48244326726869	-4.28792305183451
H	4.64679290190186	0.32515038952463	-4.18025283582278
H	4.74027291953879	-2.13890210924119	-3.80628758837878
H	2.62149023535325	-3.42764515877886	-3.56129603688011
H	0.43015994588357	-2.26609961112014	-3.70357736474487
H	-0.98919638176434	-0.21179002794200	-0.27359729729973
C	-2.04380694995833	-2.05291761409995	-0.27272432511834
C	-0.94829612752246	-2.96840024696043	0.32152526031255
C	-3.16333325070578	-1.81323807162821	0.73954735540755
O	-3.72327859225286	-2.77744891411889	1.24185166411282
C	-4.49739968672910	-0.21558893305638	2.03403163021875
H	-1.44462312981461	-3.64073659554488	1.03444987215720
H	-2.53988618269468	-2.59445522517439	-1.08741070795579
H	-4.82827056866869	-1.18055986703077	2.45108410622511
H	-0.54975515697873	-3.60193270925523	-0.48633718222029
N	-3.48937083422774	-0.53473633833282	1.04382013748156
H	-3.01656178074208	0.22405128888535	0.55998624175512
C	-3.97873517306515	0.67937523944154	3.15826245622186
H	-3.64675378820870	1.63773944101174	2.72148853464580
C	-2.87263204160390	0.11904390058176	4.02366930942222
C	-2.30487060573126	0.96181452079150	4.99384876290982
C	-2.39122811713448	-1.19286463519133	3.92437711492019
C	-1.27517541527431	0.51905583670104	5.82253965775024
H	-2.67979205962246	1.98408374396045	5.09523395159229
C	-1.35167947303396	-1.63424844894823	4.74766037875717
H	-2.83030943631499	-1.89513905784451	3.21489211953550
C	-0.78500155672414	-0.78165273702460	5.69358741103478
H	-0.84896594751297	1.19581116649137	6.56510161988661
H	-0.98659132683988	-2.65798662946630	4.64495466802548
H	0.02816844122338	-1.12927858542691	6.33251198297279
H	-4.84220039615712	0.92995436398880	3.78966662931498
C	-5.76931054037976	0.41983428591711	1.43863554171931
O	-6.51684050201901	1.06061025085563	2.12954396607788
O	-6.03310773773780	0.21018694687937	0.16295016946781
C	0.21327708500241	-2.21061303490702	0.97735640701608
H	0.66143854513206	-2.83094513332617	1.76813685677052
H	-0.17027804432380	-1.31308931485402	1.49901282169942
C	1.28835613649607	-1.81514593477680	-0.04265646052682
H	1.90298424091257	-2.69786973498511	-0.28992303933331
H	0.81092497421583	-1.52415958020184	-0.98931928625636
C	2.18547607133433	-0.65916965994567	0.38201371475502
H	1.56841680362423	0.09593781016128	0.89808760518316
H	2.93417864623051	-1.00417984233140	1.11821557414007
N	2.77725539884739	-0.02871510353384	-0.79895090609720
H	3.52704919719108	0.61117560180499	-0.53874356125579
H	3.19623220594274	-0.73029868208820	-1.41287689972856
H	-5.39285195003293	-0.35161854171993	-0.34319665953468
H	-5.66845738310662	-4.12826764604211	0.67712975674156
H	-5.56413344432861	-6.30514929753179	-0.50872930710565
H	-3.68588191236361	-4.69931571147472	-0.82847733957840
H	-7.30424885362799	-4.64143981878433	0.21790989868314
C	-6.33505709547592	-4.30833827947251	-0.17947912091365
C	-5.71612905383350	-5.37722637640139	-1.07934951081203

C	-4.38719182876117	-4.89276847868053	-1.65808147249662
H	-3.93082381582946	-5.65919349091247	-2.30044642588794
H	-6.40879998222111	-5.61781414001530	-1.90465293312090
H	-6.87783950449216	-2.17333869830231	-0.27683804724869
C	-6.52837500554181	-2.98120865194532	-0.93275293241366
C	-5.25650021785826	-2.55325245883739	-1.61916995217854
O	-4.77986432482567	-1.43761431048956	-1.48966218605661
C	-4.58004048917055	-3.59864477007696	-2.46727096806315
H	-3.64032843263302	-3.18289524248517	-2.85609507855057
H	-7.27830238701768	-3.12395633112558	-1.73075379807949
H	-5.24897695507333	-3.80764368578373	-3.32021072947257

93

Entire TS6

C	0.27316256072154	4.95731468705894	-1.40684924923755
C	-0.67389904941013	5.04280286018887	-0.39526736334313
C	-1.36216943159534	3.89792547622621	0.00453753547059
C	-1.10863875887157	2.66619612603121	-0.59025367949244
C	-0.14462722286501	2.57001618122306	-1.60128599999206
C	0.52900291857669	3.72328574532191	-1.99824100049928
F	0.91977176805946	6.03732298024229	-1.80513412354836
F	-0.92684512724218	6.20435207193625	0.17722931246263
F	-2.25753897674226	4.02948587901467	0.97130943906938
I	-2.19976522458598	1.03020927241331	0.13640545198817
F	1.43683740899617	3.68724090190560	-2.96622942624984
C	0.23264797061556	1.26099208982952	-2.25843889118455
O	-0.58334620313135	0.54345682417354	-2.81773647228616
N	1.54434518020032	0.99115846802980	-2.15387285251041
C	2.19958337023236	-0.12700166916416	-2.80728119364025
C	3.65454171673590	0.24284027247031	-3.14213839723259
C	4.37131004365744	0.96727835322610	-2.02186070534131
C	4.40302249349277	2.36909372163709	-2.00987635316629
C	5.02243288624557	3.06555583963534	-0.97099543835558
C	5.62939128373746	2.36716118213802	0.07337141155249
C	5.60480596517816	0.97070044373325	0.07259409177430
C	4.97836508134155	0.27591677283263	-0.96344498626970
C	2.11907822710656	-1.42981193994134	-1.97985583562765
O	3.10890917253369	-2.07581392246233	-1.68394669349782
N	0.86975835747162	-1.79776274843050	-1.61614555939388
H	2.05945465650055	1.51287962228474	-1.43788741936152
H	1.64429700690445	-0.31833409415979	-3.73963570707017
H	4.18294370498018	-0.68345673565071	-3.39937745303948
H	3.63872366254371	0.88985008410932	-4.02960846710266
H	3.92979101183444	2.92173155367705	-2.82463805980289
H	5.03593724628805	4.15673234566103	-0.98274680706531
H	6.12121767101519	2.90798732622878	0.88381282583881
H	6.08017319735892	0.41586278131160	0.88379638773489
H	4.94803792488457	-0.81433277440790	-0.96050417931536
H	0.09379421851370	-1.25477206508777	-1.98972323061261
C	0.67858800386118	-2.92342108410632	-0.71612231658674
C	1.21455053410807	-2.71147425384664	0.72150114248445
C	-0.74965154656588	-3.44948258956808	-0.65983940732661
O	-0.93472841104327	-4.60123176773518	-0.29819070315960
C	-3.15143877197042	-3.14729915515567	-0.95321880277540
H	0.91006816644549	-3.60483063445166	1.28749306740433
H	1.24578631778832	-3.77300356666047	-1.12361028358871
H	-3.17281678928231	-3.97547463362895	-0.22945625334912
H	2.31233072953824	-2.73402941647616	0.65870816377337
N	-1.78548927171273	-2.62737958170193	-0.95703203425613
H	-1.64399304576182	-1.67374889013383	-1.27890364125738
C	-4.17210348513354	-2.08782535834906	-0.58732717293557
H	-4.09447796809949	-1.24997192056128	-1.30182404115721
C	-4.08922895755835	-1.57255241428712	0.83382657610898
C	-4.98190672907689	-0.56334633467866	1.23014733965457
C	-3.17185923389295	-2.05387552005014	1.77777518679217
C	-4.95385038767035	-0.04207830916976	2.52170037295743
H	-5.70436928622424	-0.17885850584147	0.50471595194869
C	-3.13735953981151	-1.52665644274318	3.07170972473620
H	-2.46957632203293	-2.84741192340884	1.51580403254499
C	-4.02319854764771	-0.51892602664000	3.44774328743812
H	-5.65559694673485	0.74450453985149	2.80403662420604
H	-2.40904454273253	-1.91118154085394	3.78721847324016
H	-3.99229384085232	-0.10803515572403	4.45754613059716
H	-5.17118484612022	-2.51814093515592	-0.75627473449823
C	-3.42194678886276	-3.73076528889646	-2.34699908891413
O	-4.01435166868686	-3.13988023670887	-3.20662752759381

O	-2.86983792786093	-4.92044741282323	-2.56439379833207
C	0.79227746861993	-1.43679483504750	1.46331392133308
H	1.13363221480775	-1.53436819651416	2.50743725332794
H	-0.30903855708386	-1.35268372494592	1.50514730698229
C	1.39003356929000	-0.17985890354812	0.83452995295961
H	2.41434457210785	-0.40636566223415	0.48161002901050
H	0.81078388156836	0.07099616036850	-0.05628640799711
C	1.46871173030257	1.09268812627439	1.66085828468736
H	0.45340729354008	1.43468144634249	1.92186814585353
H	1.99763380128619	0.90414433539920	2.61544429911123
N	2.11136007159445	2.12006802602266	0.83335387302725
H	2.05312075544286	3.03390227930560	1.28032731350298
H	3.10967909430150	1.91419495788119	0.75270307347959
H	-2.62363103944950	-5.35010716591744	-1.71651924797719
H	-4.70864543927414	-7.57594887159246	1.44666557014881
H	-4.00114789536066	-9.07628960256201	0.79292849962150
O	-3.40340788229580	-6.35746097015371	-0.30740029237985
C	-4.63998288060104	-8.20792820765466	0.55154044420247
C	-3.92031575182016	-7.43323350311484	-0.53012610046911
H	-3.18691787654990	-8.95430142704739	-1.79683900530827
H	-6.46019221461219	-9.34045284375934	0.85807926912580
C	-6.01036791271886	-8.71111664313278	0.07639281458192
C	-3.87394771218109	-8.09467267385004	-1.89000671547998
H	-3.44865147837493	-7.39166135636290	-2.61640170742097
H	-5.27736436992363	-10.37978701324950	-1.08726510051042
H	-6.68203383355330	-7.84878678554419	-0.06437857902717
C	-5.89395693385741	-9.47661494922852	-1.24058554494737
C	-5.25756153631920	-8.60335521537288	-2.32027739641519
H	-5.91089165667983	-7.73823685346848	-2.51978763518708
H	-6.88628042676142	-9.81946090226200	-1.56742228931934
H	-5.16419347081802	-9.15480683433273	-3.26624838863319

93

Entire Intermedia H

C	0.04193974611788	0.97766061108070	0.46840411339382
C	0.26219777398895	0.86362371521495	1.83383108673695
C	1.48402067561193	0.37526558605114	2.29689785367617
C	2.48553060681170	0.00044950333894	1.40826046504239
C	2.26761246497063	0.12022990424432	0.02828304129790
C	1.04364873265260	0.60029241058111	-0.42057693170960
F	-1.09681475576980	1.47306467217659	0.01568178155022
F	-0.67506808648283	1.22641396232886	2.68981475182636
F	1.65968689922678	0.30643001221730	3.60958221397710
I	4.31261139239648	-0.62130149296811	2.22857469122990
F	0.80555488358892	0.74388391308556	-1.71650467586910
C	3.37347576750764	-0.16012425732732	-0.96957160917948
O	3.93476656633665	-1.23276082833618	-1.03548251520917
N	3.69744026855209	0.93511340916923	-1.69738653598391
C	4.78941677359187	0.95690996713812	-2.64829367710541
C	4.31705300027802	1.26735327869786	-4.07693687418493
C	3.36939193371162	2.44253582902608	-4.17051689322810
C	1.99104869266995	2.22098646592023	-4.28505589348788
C	1.09569152550419	3.28824650393571	-4.37384455605985
C	1.56824240962645	4.60121649364941	-4.34052236816260
C	2.93970754041916	4.83607373449917	-4.21616646761735
C	3.83322931807427	3.76662076568556	-4.13394278895734
C	5.91362934050783	1.92070875993805	-2.20330706197475
O	6.53233050479843	2.59913856617082	-3.00328020807558
N	6.18204016013513	1.93853531231868	-0.87584725568128
H	3.10294376640174	1.76448416307396	-1.59370200309667
H	5.22950201400036	-0.05261052040304	-2.63210317259687
H	5.21113356202691	1.44663458668791	-4.68796031730255
H	3.81262969164431	0.37082134550829	-4.46164415062249
H	1.61539339432442	1.19561773846106	-4.30928062759659
H	0.02629291111030	3.09211246961713	-4.46908629571614
H	0.87174720572005	5.43822152694235	-4.41317648919021
H	3.31863481941893	5.85950950749012	-4.19474598293173
H	4.90600016458419	3.94887092891572	-4.04835225854931
H	5.60551610713097	1.37379803860782	-0.26179146204340
C	7.08218361935241	2.93776551600941	-0.31442101000440
C	6.51137542389963	4.37455044243741	-0.28464057220943
C	7.58975867631526	2.48811712588860	1.04792666308148
O	8.79401261927110	2.42326727817719	1.28209335873754
C	7.08136885656239	1.87995893245818	3.35788444855115
H	7.28589345180521	5.01233373739964	0.16956152472696
H	7.97637416987719	2.96237046906164	-0.95000963853766

H	7.92826008621735	2.54077546652516	3.59858582317151
H	6.42784001309469	4.68518274357041	-1.33794313520637
N	6.67210495626698	2.18252425037247	1.98770477791453
H	5.68005690355501	2.30439085843963	1.80380707407057
C	5.95598466791906	2.08222035105333	4.37175925320593
H	5.28607340547079	1.21416547074025	4.35174988687992
C	5.16641410855818	3.34567540108986	4.14016943709921
C	3.81132419024386	3.28028264800899	3.79426276663370
C	5.77472027156167	4.60413670872956	4.24947603178027
C	3.07504058904106	4.44844736613730	3.57822568393957
H	3.32256569866505	2.30453615088413	3.71591165284019
C	5.04457635931111	5.76917738568643	4.02271012779831
H	6.83124614280115	4.66736170112903	4.52303782457148
C	3.69004568446517	5.69458042287444	3.68721151516553
H	2.01310123232894	4.38067625963887	3.33364813003395
H	5.53154914100285	6.74154411117851	4.11490682586270
H	3.11590466058290	6.60713717214062	3.51858610977732
H	6.44028530744465	2.09277174170099	5.35879312055191
C	7.61632042557590	0.43926783290778	3.40882640899017
O	7.01610427468643	-0.47892422619940	3.90840333852758
O	8.79138541918523	0.25840377843024	2.82442559679715
C	5.15993627279172	4.62065926196930	0.40024303927922
H	4.93976116178274	5.69597849303901	0.29473490619915
H	5.21524497296711	4.43991806769899	1.48671704577453
C	4.02767017265197	3.81240084156894	-0.23063402527572
H	4.23621156702474	3.71410931790808	-1.31175419661812
H	4.00879917807285	2.78532760229923	0.16848574695081
C	2.60631005001141	4.34119015233852	-0.12066903083589
H	2.25356491108278	4.27330521718474	0.91912739084732
H	2.57622461839025	5.41165994228434	-0.40159469688401
N	1.76000090793664	3.49783257190715	-0.96715806157003
H	0.77254502181924	3.61510106476436	-0.74404736131197
H	1.85626347817305	3.78407632146390	-1.94309416411774
H	9.07261252487763	1.08612387620143	2.36189154454090
C	11.19399829375064	-1.83317388167268	5.28957076004621
C	9.68266798795396	-1.88342205039501	5.50523725827894
C	9.19365076426314	-0.66794389390485	6.30367596573868
C	9.67191397071459	0.63917970736326	5.71310394155026
C	11.13219379503666	0.69562076093085	5.32066843148489
C	11.58955294190752	-0.55358784183792	4.55488725851726
H	8.09945442445202	-0.63459831118210	6.38514350104476
H	9.17041950693122	-1.89330086657554	4.53047211083036
H	9.39182952390715	-2.80395379527452	6.03156914030331
H	11.70930956656303	-1.87466375448144	6.26547663966215
H	11.52397705869104	-2.71272643053677	4.71732717551904
H	11.30311889388567	1.62278237872270	4.75721083254612
H	11.69952093865267	0.76671576550355	6.26641774700688
H	11.11927917206321	-0.55416379008108	3.55955439305612
H	12.67756547339672	-0.50835170490219	4.40251236713844
H	9.61429596573726	-0.71310330118958	7.32497860926238
O	8.93760368629592	1.59810489792895	5.59022180804243

93

Entire TS7

C	-0.02506964589367	0.01242730151256	0.03210744699118
C	-0.00072819946748	-0.02079679279485	1.41844099946189
C	1.22356038841232	-0.05034211577056	2.08375250643975
C	2.42420772726083	-0.04978451114374	1.38099540219039
C	2.40795954084582	-0.01406347528172	-0.02467341822207
C	1.17447682112620	0.01226911298701	-0.67334777907030
F	-1.17762955623154	0.05638469818091	-0.61321226632098
F	-1.12776715212836	-0.01825590924157	2.10390777890325
F	1.19304549172159	-0.06909115270057	3.40797916403781
I	4.16976394093487	-0.06363512820516	2.55056289126191
F	1.08941907788530	0.05121494129770	-1.99602393747681
C	3.68760116314772	0.02680008176197	-0.83500919125886
O	4.64058137165773	-0.68545712083613	-0.56728136539865
N	3.68715307445282	0.94560802882688	-1.82021104841263
C	4.85150349099392	1.18750382235237	-2.65198275696342
C	4.44456216801071	1.48864396464256	-4.10194634422697
C	3.28056131045129	2.44699169891425	-4.23370877622035
C	1.98532008940043	1.94392801137169	-4.41273090263905
C	0.88986720687186	2.80070643043196	-4.52800093933476
C	1.07519603673421	4.18261851119108	-4.46679054362095
C	2.36091977101191	4.69700079716154	-4.28295690081464
C	3.45460585715112	3.83728904672355	-4.16454642091956

C	5.75675753045804	2.30201945480282	-2.07452998481713
O	6.14627506174602	3.23877087196129	-2.74913678852695
N	6.08774499811500	2.16607473492310	-0.76877578849312
H	2.89443639670903	1.59984959141266	-1.85604710257169
H	5.45206972001042	0.26507965358295	-2.62675544401077
H	5.32707194122394	1.88663917518822	-4.61854446647161
H	4.17156443741222	0.53557548711823	-4.57455648803831
H	1.83503753592454	0.86311154492103	-4.46208660282596
H	-0.10975139697571	2.38636090382054	-4.67027736317689
H	0.22243499782813	4.85668394348818	-4.56388146171512
H	2.51571288230140	5.77657308947058	-4.23817135617241
H	4.45958012504515	4.23614319433669	-4.01970808795993
H	5.76912563820506	1.34534719886297	-0.25817846831592
C	6.77591558942060	3.24472586069501	-0.06652229439870
C	5.95104732984016	4.53642385378452	0.15502990720020
C	7.41792540449590	2.65031665418442	1.18162508941098
O	8.57280739093188	2.22840878468251	1.16022566308125
C	7.21867886675506	2.14952000817747	3.56632510681130
H	6.56101143389509	5.19479617542809	0.79453230266666
H	7.63244495866933	3.53478583838238	-0.69137470386062
H	8.23310371245315	2.57021334750042	3.64418963498487
H	5.90857833620063	5.02028109772950	-0.83224931445731
N	6.66171615997292	2.58186703801089	2.28991609060952
H	5.70431004777615	2.91677929793943	2.26923864485874
C	6.36461112306868	2.62252352609391	4.73679770270264
H	5.40664425096873	2.08174057988064	4.73168561411206
C	6.13284001586752	4.11248888104929	4.69339528686990
C	4.85251220884928	4.63389539109891	4.48347002431266
C	7.20977265045702	5.00093028763780	4.82011999401448
C	4.64811270338922	6.01322071094789	4.39220992727432
H	4.00463568663467	3.94907483087493	4.39211317075057
C	7.00970945389801	6.37656888467303	4.72965531266236
H	8.21358997519415	4.60482672622264	4.99657812373710
C	5.72699880707134	6.88711398942766	4.51114423297239
H	3.64192061286958	6.40315245668297	4.22805890436987
H	7.85754115330040	7.05602790805619	4.83244374474179
H	5.57113568447057	7.96473871115497	4.43996113328387
H	6.90341398938633	2.33016031463401	5.64916250693447
C	7.37761696862088	0.62375244616730	3.57411118850793
O	6.68467805540877	-0.10908531320723	4.23740825378072
O	8.31769982458201	0.15085676980726	2.77403491691510
C	4.50745384586028	4.49269798197267	0.68793922470949
H	4.14021171202984	5.53234497460513	0.66477729296983
H	4.45079477849080	4.20719496219603	1.75204896709738
C	3.59432216465522	3.60785219517096	-0.15137930881132
H	3.85243084915513	3.75451428688077	-1.21670799623417
H	3.80488277650628	2.55673522119938	0.07537211904214
C	2.08861977369519	3.79484528476890	-0.03473584498132
H	1.72947887005076	3.39131805403954	0.92632700543292
H	1.82921197139266	4.87077446893140	-0.04910117924012
N	1.46414531476462	3.03782604895210	-1.12420097373663
H	0.45960198356433	2.94153392355264	-0.98176283032495
H	1.57180561576401	3.55051428350723	-2.00197787267509
H	8.68602515090011	0.88115111279961	2.21102264463974
C	10.57550405527417	-2.41810171425444	4.92919494836592
C	9.15369758363121	-2.08779810140616	5.38007415376620
C	9.11628037350894	-0.79789531231547	6.21194285605405
C	9.80810909464712	0.35025832880407	5.51292864791309
C	11.16759159311637	0.03975222004397	4.92697488055546
C	11.16416393441550	-1.26523489287600	4.11775336870102
H	8.09168414533394	-0.49258528074481	6.46045599868832
H	8.50734807702746	-1.95603846477972	4.49823592352704
H	8.72836400819035	-2.91194020695010	5.97070932334423
H	11.21008773747424	-2.61049066600938	5.81226709686629
H	10.57638303451051	-3.33981452285187	4.32886413420957
H	11.49707822729920	0.90378307740701	4.33476887906578
H	11.85730882049561	-0.06962004070907	5.78310217481825
H	10.55791983157888	-1.12141800461281	3.20985887114910
H	12.18916793686234	-1.49523885726502	3.79306692850848
H	9.66866808076564	-0.96024123978945	7.15545251660370
O	9.31559242223805	1.45803073674490	5.44391468983605

93

Entire Intermedia I

C	0.19418157565797	-0.39082969846715	-0.26895522292949
C	0.13751465379060	-0.46655423931816	1.11662217869735

C	1.31331739574697	-0.39358743203081	1.86249914119588
C	2.54785274373383	-0.24512874374862	1.23824655339761
C	2.60836203509240	-0.15477253025205	-0.15850330532466
C	1.42815654788470	-0.23062627029468	-0.89136690785844
F	-0.91434651541085	-0.46563954215937	-0.98376751102448
F	-1.02701116794502	-0.61117118818814	1.72203306953863
F	1.20743866149440	-0.46458881669872	3.18068891447380
I	4.24683036865605	-0.13218378351661	2.46325006218663
F	1.44228999802127	-0.14322717325139	-2.21652171957922
C	3.90283319504339	0.09812407636216	-0.90344702614809
O	4.85603727969291	-0.65881302583236	-0.84850778504704
N	3.87500142135855	1.24730464790263	-1.60395161128684
C	4.97801580831502	1.70441859568442	-2.42766545690824
C	4.44750674788589	2.43483695948769	-3.67213015092969
C	3.30942832650107	3.38933240590275	-3.37872843939990
C	1.98403470704799	2.96072069210190	-3.53663899539115
C	0.91428646481312	3.81022354690361	-3.25084899514087
C	1.15516206093521	5.10852792966778	-2.79876112213298
C	2.47112991575279	5.54509076625135	-2.63032953467906
C	3.53952694180187	4.69317660585307	-2.91648296184737
C	5.98008540503734	2.58016268474401	-1.63743787473430
O	6.34953714030215	3.66912043419224	-2.04563642199694
N	6.40307044893081	2.05042777920933	-0.47132609742673
H	3.10288066123937	1.89084426284759	-1.39824174919325
H	5.53312463945837	0.80479279908532	-2.73715846983523
H	5.28868446319074	2.97061484970564	-4.12920110062791
H	4.09653948927314	1.67361453510828	-4.38174208895280
H	1.78929231440086	1.94396344054691	-3.88557282609834
H	-0.10953086128628	3.45671348757421	-3.38542432140236
H	0.32161056380449	5.77813044828987	-2.57978079649114
H	2.66873253291063	6.55966977096505	-2.27944415267082
H	4.56850194245842	5.02963342134040	-2.78124856516430
H	6.17886982150214	1.08272185305402	-0.23756308316741
C	7.16542175784189	2.81844233731586	0.49420783161314
C	6.45708661980762	4.09604051268454	0.99172556245577
C	7.57534282779392	1.85720033187601	1.60745157369019
O	7.56303909007204	0.63823589011336	1.42823951661207
C	8.57187123434925	1.58777272144306	3.82882575116560
H	7.16512073210485	4.65119085616993	1.62755841513215
H	8.11296578438335	3.14334849971141	0.02469306815537
H	9.24053611294317	0.85140142621249	3.35613800013392
H	6.31329042968423	4.73107049404813	0.10649189314759
N	7.97435803948870	2.39519870890491	2.76999031214430
H	7.95176514913137	3.40121174845586	2.89422089864360
C	9.36643167233027	2.45350476935948	4.79779507883762
H	8.68668466873285	3.16324274269960	5.29270443580196
C	10.49351570261879	3.17259657614575	4.09977552090179
C	10.45363118907816	4.55342716328556	3.88298497074508
C	11.59082630534307	2.44455959931052	3.61952166995431
C	11.48627910733820	5.19795131903593	3.19736847271952
H	9.60869978658265	5.13310228787426	4.26465402472421
C	12.62243820631092	3.08471263623584	2.93639908101341
H	11.63059201225566	1.36528141408840	3.79221411866342
C	12.57192754547021	4.46457818712901	2.72192507843892
H	11.44217314731448	6.27678690532662	3.03843230165610
H	13.47270001661222	2.50610973089261	2.57120946883937
H	13.38045869639463	4.96577322986433	2.18740950492411
H	9.75646119935106	1.77461690973306	5.56832099809857
C	7.48807044157284	0.78684415943850	4.56072862709284
O	7.14791718478080	0.99026388195074	5.69253100477058
O	6.93660901265059	-0.18411364883269	3.82860878215626
C	5.11062327727985	3.92206779655158	1.70662448051527
H	4.81130242467744	4.90699540419185	2.10112215673956
H	5.21437977633594	3.25473759309176	2.58118876781670
C	4.03722271629594	3.39114994789577	0.76305138930844
H	4.12204410836599	3.92098703973832	-0.20511100691479
H	4.24493374888814	2.33919383407483	0.55261198893745
C	2.58190901411363	3.46211654932796	1.19390596517625
H	2.41011824408566	2.77967066508632	2.04348658216540
H	2.32566804515903	4.48045279006680	1.54529909213726
N	1.76378682128708	3.01649987351249	0.06105372757126
H	0.80505304823695	2.83250800165320	0.35289633443670
H	1.70609328622568	3.76342290869169	-0.63421041343829
H	7.28122296725609	-0.14220843112981	2.89633476709809
C	7.06110029491895	-3.61578932363284	5.86390751233349
C	7.10344927495158	-2.27955802892231	6.60327803361792

C	8.53413021925101	-1.72974970366312	6.68448772835044
C	9.21676769309629	-1.69611922603468	5.33570441476710
C	9.07783075038078	-2.94501543197189	4.49335966958454
C	7.63683082832279	-3.47359553254513	4.45595173211622
H	8.56932909951262	-0.72290161716086	7.12021469192171
H	6.47338561463436	-1.54566029239283	6.07695878342271
H	6.69714450841795	-2.38229290727267	7.61980279653424
H	7.64177410573940	-4.36865755228521	6.42570577886033
H	6.02640501058742	-3.98546003255655	5.81125889184317
H	9.47857698738801	-2.73671645953143	3.49230061959380
H	9.72621337786423	-3.70504462402947	4.96576879265202
H	7.01069571860852	-2.77320694094232	3.88157993242035
H	7.61691084130419	-4.43595382053174	3.92461861186508
H	9.14396355630463	-2.40133414853835	7.31625850659942
O	9.86508987582290	-0.73817631643554	4.96486760824856

93

Entire TS8

C	0.13780572762460	-0.30249964125099	-0.15419341740437
C	0.10719435540486	-0.35991137404165	1.23336757525990
C	1.29783114531962	-0.29351722525480	1.95612625829924
C	2.52098228350512	-0.17069913456781	1.30509249211287
C	2.55605684157042	-0.10181054223428	-0.09322393613986
C	1.36155261866559	-0.16712230630057	-0.80325605965699
F	-0.98542544689553	-0.37100946898022	-0.84617463222881
F	-1.04707443223245	-0.48137925284805	1.86267063169024
F	1.21705842309490	-0.34481148020624	3.27672652678843
I	4.25071146923230	-0.06373040905649	2.48485776824012
F	1.35155960091364	-0.09687740319388	-2.12919819264127
C	3.84778031775534	0.09913871538287	-0.85696671001185
O	4.77765917479940	-0.68581499542044	-0.79543520930305
N	3.84594424095116	1.23798762528148	-1.57354821563809
C	4.96469572737636	1.65187076987522	-2.39962066046066
C	4.46615120711236	2.36468639349563	-3.66671403745026
C	3.34457505994403	3.34863829154590	-3.41188452367277
C	2.01230052641526	2.94137691736128	-3.56813636904325
C	0.95784202036216	3.82047091076006	-3.31828710464459
C	1.22118519275517	5.12797791164579	-2.90695616697920
C	2.54413980393320	5.54350534865283	-2.74146071195000
C	3.59717428106709	4.66126592771756	-2.98967527612683
C	5.97833562230337	2.52019651258625	-1.61593340301215
O	6.36317622561137	3.60074740260123	-2.02957345311305
N	6.38685445779638	1.99737270592213	-0.43976355985611
H	3.08784600835720	1.90436203655798	-1.37705993046552
H	5.50244625431722	0.73317648268586	-2.68228097463716
H	5.32454605328748	2.87258975279906	-4.12376147481358
H	4.11000343406083	1.59472343219718	-4.36439259015922
H	1.80018715428143	1.91804957163989	-3.88663323168936
H	-0.07179230278677	3.48368050818028	-3.45067155072027
H	0.39961732001703	5.82099750466403	-2.71791406400173
H	2.75946176929842	6.56488299766422	-2.42193535234391
H	4.63158006357686	4.98093788847252	-2.85520037068481
H	6.16087773076433	1.03108979372022	-0.20131549900932
C	7.17179894535351	2.76513759040345	0.50770723822363
C	6.49870067466258	4.06567020902398	0.99648936254345
C	7.57312587526553	1.80875640712917	1.62921156271691
O	7.59066846099216	0.59046715792857	1.44411595367263
C	8.57148681003176	1.55194490473923	3.85124865085388
H	7.21650437133188	4.58973617484054	1.64684084716327
H	8.12304697910667	3.06203459459926	0.02653946455450
H	9.25638572199407	0.83476095745259	3.37232088956225
H	6.39358613482997	4.71364417153193	0.11541322778749
N	7.94936829646692	2.34857209299257	2.79823302822086
H	7.90108750188427	3.35253845740738	2.92950423527196
C	9.34564536389624	2.43525064584773	4.82126177823299
H	8.64544998328593	3.11128449043436	5.33447915066312
C	10.43217138686411	3.20837425131566	4.11663688006943
C	10.32753165588168	4.58759807966762	3.91110049858238
C	11.55457213185692	2.53422506072343	3.61631339334402
C	11.32113202552219	5.28303052789783	3.21756396323941
H	9.46231377394914	5.12607571110889	4.30808037201426
C	12.54753181018373	3.22519644100782	2.92522797316370
H	11.64533226728230	1.45662654444100	3.77885233567507
C	12.43246806822621	4.60294740151842	2.72263805754113
H	11.22646226103633	6.36008832101870	3.06778920569389
H	13.41771745198573	2.68818395158871	2.54459083456460

H	13.21066502246552	5.14394128687712	2.18210710813365
H	9.77238639213364	1.76371856260202	5.57853116449340
C	7.50823857662984	0.72147871710265	4.58067462374105
O	7.18638492658877	0.89440201004499	5.72304952342397
O	6.94971554008942	-0.23443899914555	3.83408907847616
C	5.13351856774845	3.92388886433936	1.68675296356366
H	4.92809721793626	4.84633379094856	2.25327260728279
H	5.15896809887406	3.10293908184693	2.42601868305516
C	4.03001666612554	3.67498006831384	0.66409084358637
H	4.04583382396186	4.48337386858039	-0.08962446429742
H	4.27255731812418	2.75523982893455	0.13261890242513
C	2.60307705000481	3.48415805381791	1.15006896111142
H	2.57223970140335	2.66956239270042	1.89361490513471
H	2.22745594527913	4.39403663010030	1.65630489267570
N	1.79719688048163	3.08443152725977	-0.00852349706062
H	0.84706529180649	2.84617536926784	0.27305362116067
H	1.71076624703797	3.87092004822078	-0.65600407123166
H	7.28420045996866	-0.17432962532856	2.89937369936503
C	7.12169905234132	-3.70896830146111	5.83759753086022
C	7.14238837148325	-2.37524871535267	6.58228134071214
C	8.56496620958052	-1.80512983660034	6.66835350229986
C	9.24805893431826	-1.75386848623573	5.32037147143520
C	9.12803694727968	-2.99984635881278	4.47085062973131
C	7.69594842827114	-3.55185432642345	4.43045223979818
H	8.58552584907969	-0.80020205704826	7.10987940177929
H	6.50285068783581	-1.64928141099316	6.05730413507658
H	6.73543522040048	-2.48764786068944	7.59744835653635
H	7.71411056147355	-4.45477847987049	6.39681033013120
H	6.09314141398900	-4.09485862777662	5.78286802542585
H	9.52520542029364	-2.77907130474232	3.47098370950897
H	9.78880900811859	-3.75225023373549	4.93861124463284
H	7.05878202067955	-2.86014866543161	3.85793671502455
H	7.69252471094606	-4.51263152193960	3.89559561172467
H	9.18356365526647	-2.47201943220259	7.29665301604318
O	9.88287656719617	-0.78479112642785	4.95503130648866

93

Entire Intermedia J

C	0.03902478649193	-0.11354678442090	0.11823450653153
C	0.07475557442313	-0.40241983725570	1.47494606521288
C	1.30254500355419	-0.49394844895460	2.13030944179950
C	2.49630657609188	-0.30698760986264	1.44223562350871
C	2.46803523216354	-0.01460186919985	0.06845312158523
C	1.23435691620476	0.07655078864872	-0.57181407533277
F	-1.12011228565347	-0.02229411516839	-0.50977646116117
F	-1.04971641668691	-0.57960098451757	2.14254956179096
F	1.28198073669361	-0.75360422313880	3.42837142320455
I	4.26598919684494	-0.36152987725166	2.57046160112612
F	1.14905375903980	0.35252212228569	-1.86672663655264
C	3.73750574040167	0.18468021674939	-0.73320818489958
O	4.65717073690021	-0.61686643239477	-0.67024372233892
N	3.75145926980158	1.29784108465264	-1.48453495261760
C	4.87572546002117	1.61031738171765	-2.34961327236641
C	4.41260319513241	2.27500249358776	-3.65365774740126
C	3.35411932840375	3.34238200291105	-3.47693468698507
C	2.01096774817530	3.03608061324553	-3.72944248076494
C	1.01148206173565	3.99623668297623	-3.56826054585414
C	1.34237925301636	5.28520704135137	-3.14821664870704
C	2.67723686165650	5.60063819115864	-2.88452262498438
C	3.67478298771062	4.63778809482582	-3.04599518752702
C	5.94217584737792	2.45229784005247	-1.61539960568538
O	6.40968172791419	3.47670795831829	-2.08398655416214
N	6.29243398889779	1.97510771621084	-0.40164579696026
H	2.99228390524490	1.99190561003066	-1.33869560498285
H	5.36410766146620	0.65207030610990	-2.58818257669944
H	5.30129813851162	2.69673069459056	-4.14117398878245
H	4.01004365719213	1.48659439731905	-4.30376575857974
H	1.74578557573371	2.02808276142509	-4.05690094510433
H	-0.02791443286826	3.73852532145722	-3.77908586315077
H	0.56458546826739	6.04158713226544	-3.02926415720889
H	2.94614257319224	6.60694648148987	-2.55752589544708
H	4.71721996057448	4.88143763402594	-2.83606138876157
H	6.03813360008096	1.02298524875046	-0.13712438338491
C	7.13722575470776	2.73766253976715	0.49307640001832
C	6.45498093889097	4.02985993502495	0.97777489518443
C	7.56546463057077	1.78635685315680	1.60948653313120

O	7.59343375788585	0.56935454535388	1.41588512508058
C	8.55792817152511	1.51563205831684	3.82961146664273
H	7.12455964805034	4.55169697913393	1.67797041338971
H	8.06897247236853	3.01730726327913	-0.03294248479144
H	9.26613552452286	0.82408806857968	3.34658738277542
H	6.38260995351312	4.69648594785289	0.10630480960261
N	7.93356119218395	2.31989234375187	2.78327145194640
H	7.88202791775280	3.32244225737515	2.92301134001279
C	9.29782249155856	2.39526965651167	4.82933974736947
H	8.57523059655700	3.04700883279034	5.34262975622624
C	10.38208536430163	3.20255446740849	4.16074795376297
C	10.25442254208863	4.58329567324227	3.97920440308156
C	11.52782026369098	2.56029738043585	3.67148936871359
C	11.24767822353219	5.31089771433149	3.31904351248311
H	9.37141392343129	5.09723936867265	4.36887801336895
C	12.52044189685825	3.28339517363292	3.01375540868935
H	11.63679216056784	1.48177197014328	3.81656187990953
C	12.38201202070071	4.66221636465620	2.83430108244642
H	11.13477130092748	6.38844682203130	3.18776475336915
H	13.40898152061316	2.77073273243985	2.64134037400594
H	13.15996895683588	5.22863963013149	2.31995055115801
H	9.72457899742879	1.71529586683972	5.57916515869556
C	7.50695915820965	0.64526350370764	4.53281312155206
O	7.13536063990109	0.82015192986081	5.65983886567514
O	7.02731701423885	-0.35050404954040	3.78486394991125
C	5.05798794641431	3.79154248686194	1.57402189157607
H	4.89629034152930	4.43691731542026	2.45078016612125
H	4.99159530576705	2.75270787487125	1.94452578005872
C	3.95043523141727	4.02265229707607	0.54389017241359
H	3.83702676381547	5.10345447643953	0.34939044488186
H	4.25951534009467	3.59214506249473	-0.41633853205058
C	2.60112406223515	3.40765935964495	0.89669984147374
H	2.76548404349975	2.42090514077013	1.36379586296883
H	2.08244324245399	4.02759710112671	1.65100147806151
N	1.82061636328292	3.20299980160055	-0.32520405535647
H	0.86605882881322	2.91914795663348	-0.10714060458941
H	1.74531278828293	4.07515978018814	-0.85316828459578
H	7.35963626401650	-0.25886708841871	2.85022645289056
C	7.28327690205177	-3.77411040909804	5.88717525380768
C	7.26641018868797	-2.43479742481431	6.62184210239532
C	8.66919714696987	-1.81583864725398	6.68939420899240
C	9.34095460870456	-1.75587287052889	5.33595765572927
C	9.25375322801502	-3.01254472102820	4.49818980298403
C	7.83857689972158	-3.60684919158309	4.47392281226567
H	8.65936906626507	-0.80655465750144	7.12101098046528
H	6.59669770720897	-1.73473691097716	6.09817203727012
H	6.87342486966742	-2.55301708114575	7.64192140833083
H	7.90526071720610	-4.49570072248795	6.44565833799220
H	6.26754384476952	-4.19433639882992	5.84483113538683
H	9.63632364931124	-2.78831129568939	3.49349744602246
H	9.94071720476446	-3.74022214135995	4.96700473838952
H	7.17626550027315	-2.93477470774824	3.90630437491139
H	7.85754814179616	-4.56910385459475	3.94238587083552
H	9.31549344533460	-2.45391819560858	7.31967868562165
O	9.94362489296489	-0.77156459721588	4.95788098531739

93

Entire TS9

C	0.12592336319243	-0.23817634788059	-0.38739474981522
C	0.10306343446019	-0.46770009681797	0.98110676994186
C	1.29983545627018	-0.49127749437096	1.69718783963814
C	2.52114760443875	-0.30059860591854	1.05996944347885
C	2.55039202852271	-0.06393027823196	-0.32433463267356
C	1.34748381432664	-0.03751031690465	-1.02558877388595
F	-1.00077758952686	-0.21460545221280	-1.07779213085774
F	-1.04776402141192	-0.65801665956480	1.59999071275422
F	1.22289156620668	-0.69743884411143	3.00381229333582
I	4.25074488197480	-0.29327530345553	2.25131210775844
F	1.31767388556876	0.17613938829313	-2.33537472627186
C	3.84748950046610	0.11612014732779	-1.08934706346989
O	4.75405645797765	-0.69451710900845	-0.99613412967785
N	3.88368331376521	1.21778975096115	-1.85985764888569
C	4.97832002260708	1.47128425316631	-2.77750539153186
C	4.46814611296518	1.98588745967191	-4.13222491689467
C	3.40301081935960	3.05584751659847	-4.02927385378063
C	2.05539951254738	2.71584098762679	-4.20211485421094

C	1.05004474853740	3.67548452638517	-4.07946363335102
C	1.37959537291262	4.99834476031535	-3.78127204793518
C	2.71996924385688	5.35011028307029	-3.60685652328902
C	3.72343256194771	4.38789178273331	-3.73014470745419
C	6.04730474843368	2.40713155675362	-2.17575921951899
O	6.52890366602194	3.33785774591963	-2.80175735399601
N	6.41805723414898	2.11209749063425	-0.91345346255098
H	3.11743427711624	1.91207964094141	-1.74894656132282
H	5.48787736870266	0.50615836164711	-2.92833928473919
H	5.33729862796331	2.36253011095474	-4.68703308905605
H	4.05536939316594	1.12886447717601	-4.68124764159538
H	1.79134002411345	1.68145709499621	-4.43164422794399
H	0.00640258581439	3.38866853578788	-4.22059872042120
H	0.59643176391625	5.75333389233127	-3.68977914274901
H	2.98813329781871	6.38393808766706	-3.37966825873507
H	4.77101934282603	4.65985795538513	-3.59185485550984
H	6.05511173267925	1.28892217407845	-0.43261204731870
C	7.28568890498176	2.99025462559232	-0.16251264323348
C	6.58646345546594	4.31256439141745	0.23709722277958
C	7.70391949148110	2.24227360311728	1.09701893151577
O	7.11519832798108	1.23398710619869	1.46616932967386
C	9.07008089370212	2.26128688095872	3.12533092006807
H	7.26610809486031	4.87812547716285	0.89115470164518
H	8.18034683881538	3.22673909969391	-0.76108153397164
H	8.93158304907692	1.16808809360086	3.07759811359379
H	6.46038223727623	4.91101751721880	-0.67766264509195
N	8.70016907537463	2.79141418453368	1.81945123253715
H	9.13459672138941	3.65352764582248	1.50631026225132
C	10.50989213972028	2.61887078772071	3.47761515632433
H	10.59807833082380	3.71432215117104	3.53284660912994
C	11.49403207666602	2.04738373524518	2.48713516606617
C	12.10889047472595	2.85804921225817	1.52764546165351
C	11.77957713608969	0.67621633249131	2.48979008682357
C	12.98694999264504	2.31302916607767	0.58869169306826
H	11.90377633383646	3.93146510507811	1.52081952466870
C	12.65506509652601	0.12756602811995	1.55478122725587
H	11.31123125751744	0.03425404909829	3.24038964141904
C	13.26091051585723	0.94609978606807	0.59916069698643
H	13.45969542026045	2.96023203409873	-0.15182118382124
H	12.86850554574713	-0.94280906189364	1.57257505861229
H	13.94689573728854	0.51820091987709	-0.13370495054164
H	10.70909338472599	2.23755640115808	4.48910544816284
C	8.08239268464001	2.80320330837715	4.17049808610551
O	8.38147636861265	3.61426052348169	5.00295228272846
O	6.84151649862736	2.34170378195013	4.05547480060912
C	5.22609119013276	4.07801891370783	0.91309456913589
H	5.07178208998245	4.80748518206577	1.72196763050163
H	5.22469656294704	3.08517306144898	1.39787798327245
C	4.06475778125085	4.14905565303016	-0.08203648378758
H	3.82539749082387	5.20298368613862	-0.30731488997136
H	4.37856943047717	3.71581683985569	-1.04156582737178
C	2.80733643882776	3.41284624250056	0.36062977941154
H	3.10233354268307	2.44849097856088	0.81134355995002
H	2.28981115914709	3.98337894577472	1.15366014170662
N	1.96130323058349	3.13280820680831	-0.80034841343330
H	1.03819672327054	2.80966473301910	-0.51314955574943
H	1.81273306459523	3.97985492501154	-1.35273445950607
H	6.79731066104624	1.60480837229601	3.41118262673856
C	7.83589624693529	-3.16046313125695	6.73651985101981
C	8.82744163784531	-2.14299403512076	6.17388844455622
C	8.51657752892458	-1.82341820012375	4.70555560611349
C	7.07088356349979	-1.42878186076246	4.50735132983817
C	6.04799346029035	-2.34180691564108	5.14182195867584
C	6.40138585627885	-2.65256015728556	6.60388403308617
H	9.16033964161148	-1.03455064890372	4.29341536845191
H	8.77301151402397	-1.21468948162990	6.76701826947274
H	9.85860381972967	-2.51408753588781	6.25667385612189
H	7.94023889764958	-4.11342087973043	6.18990766829330
H	8.06619217834890	-3.37001630087947	7.79142508008423
H	5.05277761091724	-1.88927795008067	5.04132059053903
H	6.05677126521252	-3.27994061010682	4.55813728022326
H	6.28506276942731	-1.73556991676809	7.20403987949940
H	5.68651667170523	-3.38636321634714	7.00269457542294
H	8.67208065795627	-2.73355933557201	4.09979714844146
O	6.75662807545924	-0.44815392176492	3.86324318477679

93

Entire Intermedia K

C	2.60880083094037	1.93598490371344	-5.52040085328857
C	1.41448142709627	2.61712898761705	-5.31608137952207
C	0.42243932752220	2.05345846402904	-4.51486080334388
C	0.61825193607355	0.81725884942981	-3.91106584625231
C	1.81875887555933	0.12733897153075	-4.11096934326472
C	2.79548680318683	0.69260718062883	-4.92094295889995
F	3.55477665141248	2.46866600562397	-6.27256822071875
F	1.22335320690261	3.79659580098572	-5.87909238225477
F	-0.69736672581461	2.74125164452083	-4.33780192519114
I	-0.87672231634023	0.06636193184563	-2.65843967706234
F	3.94999636053079	0.06855724989828	-5.12136775396120
C	2.11951804020514	-1.22055605820771	-3.48217531109175
O	2.14224419992337	-2.23597161178238	-4.14434923917534
N	2.34967447750191	-1.15081294844586	-2.14974291266298
C	2.85045338338675	-2.30504388206176	-1.42665083571987
C	4.38985421556881	-2.34766559243985	-1.41347064748480
C	5.01734442903823	-1.01671363571928	-1.07446692334660
C	5.43643299777512	-0.16066548786835	-2.10194504961098
C	5.99121280330939	1.08773515284385	-1.81582659408706
C	6.12579600122387	1.50470897574575	-0.48985864248431
C	5.70239865131965	0.66441823596338	0.54230885464524
C	5.15435566906362	-0.58683261767432	0.25290069229156
C	2.26053569931498	-2.41692956625608	-0.00732172173191
O	2.93711538370814	-2.80406965145188	0.93153616137398
N	0.95112163336692	-2.11247696823222	0.11010123325085
H	2.47294487221832	-0.20775207592692	-1.76192461709146
H	2.48962807458099	-3.18904797896175	-1.97590433106543
H	4.70068111329267	-3.11956021476626	-0.69802811704738
H	4.71437895308659	-2.65251078752554	-2.41824750807730
H	5.33112077657951	-0.48123775013332	-3.14121970814438
H	6.31734432236131	1.73517890798362	-2.63199818403868
H	6.56130730634157	2.47893026953525	-0.26120939716923
H	5.80673244391830	0.98152036616855	1.58160858474152
H	4.82212430427090	-1.24565380222472	1.05785580088925
H	0.45638597026668	-1.72854349235697	-0.69183073023632
C	0.30224502712345	-1.98363877846210	1.40118716459371
C	0.89972065830784	-0.89066049087623	2.32591925692097
C	-1.16676164963589	-1.66270318067629	1.16109566074969
O	-1.54589568713391	-1.06765894929810	0.15898363539579
C	-3.38321533830824	-1.55099400176613	2.13026272528803
H	0.36994593066710	-0.96229107625577	3.28831327307489
H	0.38293765582709	-2.94464363260764	1.93423452292682
H	-3.90619177778083	-1.97639101837420	1.25997677383770
H	1.94580546446392	-1.17428404728394	2.51304045019688
N	-2.00900596632318	-1.99832999189889	2.15531568637515
H	-1.66341162671052	-2.50284164216112	2.96502367229864
C	-4.10436013420962	-1.95723865487140	3.42411452460029
H	-3.63823665134115	-1.42419938199251	4.26685601661511
C	-4.06383940498959	-3.44706332105814	3.66414021637664
C	-3.26742818330888	-3.98866486038387	4.67877483493895
C	-4.80035556840814	-4.31683912789123	2.84881204943102
C	-3.20289056339509	-5.37022241679437	4.87513035932164
H	-2.69831721230953	-3.31946395356387	5.32961979428444
C	-4.73980297389447	-5.69482920686119	3.04361033360161
H	-5.43101015572178	-3.90643987223984	2.05582354294277
C	-3.93847671429354	-6.22587392597810	4.05745392764353
H	-2.57822409984012	-5.77634522204037	5.67247206270659
H	-5.32212931141182	-6.35936211823621	2.40316163208031
H	-3.89143284322457	-7.30527794022720	4.20967191128595
H	-5.14362390651646	-1.60724126685822	3.34187184211256
C	-3.47229734399971	-0.03410011017921	1.97079865760440
O	-4.36503427668272	0.49987094871324	1.35991769189907
O	-2.51565497162650	0.60487419813702	2.61467834494873
C	0.84162597070549	0.55438438283550	1.81494953251965
H	1.30475522000550	1.19612688432320	2.58287360091609
H	-0.20578405481219	0.88443051479385	1.72052266715281
C	1.57071544179903	0.71877215689727	0.48886128628150
H	2.53245712017688	0.17399549311762	0.54282787777746
H	0.97875887863731	0.24324274871210	-0.30007423988992
C	1.87478408171320	2.11607158409564	-0.01988444842416
H	0.93322711656426	2.64426960536432	-0.24113924503301
H	2.40797702529024	2.71111056461710	0.74618476724927
N	2.63202351044604	1.96304000994370	-1.26614341729589
H	2.63918572934529	2.82722047657485	-1.80522542736773

H	3.61006142076478	1.76110527529240	-1.04916691040537
C	-3.94422058729086	3.87725690810130	-1.71178624724441
C	-3.64210035852746	2.46818800423666	-1.20463511759325
C	-2.41673002671850	2.46965557829923	-0.27389081171940
C	-2.63725334450872	3.45010214793868	0.84662681119711
C	-2.96888794274402	4.85477338487487	0.41361481773697
C	-4.17082758551443	4.84149817231390	-0.54778784724375
H	-2.18829546442671	1.46795007780843	0.11749276567386
H	-4.50165636129513	2.06999012232437	-0.64356054423830
H	-3.45575129115187	1.78229739166116	-2.04341895116034
H	-3.09944970941639	4.23205236432372	-2.32760769768731
H	-4.82987234640071	3.86251545249211	-2.36366939398846
H	-3.15354878961321	5.46762087939281	1.30493727903567
H	-2.08793806831575	5.25474153357542	-0.11645301822382
H	-5.06874988777352	4.53087670509695	0.01071780064996
H	-4.35575657808076	5.86257070798021	-0.91034184000575
H	-1.53997791028346	2.82321327342430	-0.84551180624453
O	-2.59347032554259	3.14523881931545	2.02712359897484
H	-2.57644935704732	1.59083104623011	2.42998388138139

93

Entire TS10

C	2.54012611642318	2.09924593139638	-5.35865603709143
C	1.31103227531376	2.71720743179054	-5.15813631050329
C	0.31456206801471	2.06154828033393	-4.43673340401252
C	0.53964357737747	0.79429800050767	-3.91298521897123
C	1.77442553211904	0.16854125008367	-4.10800298395052
C	2.75602510291202	0.82535025299227	-4.83839699387521
F	3.49163914192703	2.72012687590728	-6.03232536577491
F	1.09284942206669	3.92414700887872	-5.64901093806579
F	-0.83987969662159	2.68922581511281	-4.25692333403263
I	-0.96453096149563	-0.09452175397390	-2.76990773992443
F	3.94270578630584	0.26151394521869	-5.03060100357160
C	2.10989961162646	-1.19421930205762	-3.53110525357817
O	2.16887798052112	-2.18673990915710	-4.22324287707359
N	2.32591600205679	-1.16137777451417	-2.19318072313119
C	2.85053369549907	-2.31808256368987	-1.49208742398654
C	4.39033349606484	-2.31150213940476	-1.44660188563139
C	4.95914551089058	-0.98530872537737	-1.00121205702151
C	5.34484924421641	-0.03356570781024	-1.95528753812462
C	5.83313629626492	1.21518517327798	-1.56667070081474
C	5.92976048505211	1.53701298241912	-0.21129215967057
C	5.54554109790721	0.59794796899251	0.74815688120700
C	5.06843481936624	-0.65428106293226	0.35694936247379
C	2.24471150948584	-2.48127346840839	-0.08480651906319
O	2.89999517406978	-2.95192628412792	0.83082563507968
N	0.95394585978676	-2.11464178035228	0.05267386192678
H	2.40987255662340	-0.23259193910962	-1.77213614308806
H	2.52903657112870	-3.19806695458641	-2.07130598924475
H	4.70965808953098	-3.11939317274818	-0.77625325572393
H	4.74742814383583	-2.53794638402598	-2.46111032942337
H	5.26609730084043	-0.27771676147788	-3.01745655922785
H	6.13606139288232	1.93861735301228	-2.32606739525754
H	6.30995891403382	2.51267451892071	0.09625870623121
H	5.62485596029960	0.83876943626860	1.80985602183400
H	4.76707873591211	-1.38980621315095	1.10558072857342
H	0.46715611333058	-1.68462084912571	-0.73010291913134
C	0.32427128066258	-2.01421550649262	1.35493599967454
C	0.95839817301876	-0.94614154790770	2.28171634063715
C	-1.14635534838078	-1.67758091068117	1.14729716826961
O	-1.54070601650663	-1.08715433423472	0.14884147966657
C	-3.34284703008992	-1.53269560792351	2.15358365700051
H	0.47892096080653	-1.04526632475609	3.26785571239601
H	0.40872557485296	-2.98728705860944	1.86461845640043
H	-3.88276830488608	-1.96405946505114	1.29662953830168
H	2.01363417669041	-1.23043015201964	2.41066529233105
N	-1.97177022563653	-1.99234991048045	2.16241388216774
H	-1.61830653445715	-2.49584939822496	2.96930051196434
C	-4.04721174416363	-1.91786968299047	3.46273496113533
H	-3.56235895221933	-1.38171768975382	4.29278875112364
C	-4.01749430578840	-3.40596191207490	3.71459021079845
C	-3.20332095848643	-3.94852222707707	4.71446974288572
C	-4.78027277250701	-4.27392356685350	2.92177349924094
C	-3.14657350701498	-5.32941244339537	4.91787958481333
H	-2.61345727265000	-3.28046589976379	5.34792664160964
C	-4.72762209871096	-5.65119518615526	3.12374715735962

H	-5.42467426171657	-3.86258261139914	2.14042868807850
C	-3.90803053669737	-6.18332038474305	4.12224638438481
H	-2.50740096156518	-5.73639160416347	5.70322056139252
H	-5.33010968513730	-6.31424234587537	2.50075153927320
H	-3.86685916537525	-7.26214474958545	4.27980536542741
H	-5.08417708799037	-1.55833191953853	3.39341121081358
C	-3.41987924200279	-0.01677803259690	1.97293865909970
O	-4.30892900894463	0.51731759843838	1.35753688285182
O	-2.45326189836922	0.62033107906158	2.60365786459771
C	0.86859460367387	0.51143509894554	1.81497693706311
H	1.37152231916028	1.13221368146321	2.57518085761967
H	-0.18257131976564	0.84078844463158	1.79085697628406
C	1.51678838396208	0.73683438561070	0.45530639708111
H	2.48020373514227	0.19304089807727	0.42355919184040
H	0.87569264494177	0.30511684024386	-0.32410334161948
C	1.79221665151250	2.16718009908024	0.02790315147914
H	0.84047569954574	2.71278428156474	-0.07696025972513
H	2.38176649343934	2.69628619783971	0.80179297684062
N	2.45641117675232	2.12014856390235	-1.27681764821033
H	2.45857159311617	3.03678208122529	-1.72108239927466
H	3.43930026223413	1.87361343889389	-1.14596929348868
C	-3.78981578963935	3.85653174882103	-1.79743297735389
C	-3.56394927378977	2.44658754701976	-1.25417825141019
C	-2.31056916999675	2.39579022300159	-0.36165804415057
C	-2.44263657939796	3.41894726067791	0.73421318288497
C	-2.68484126407349	4.83041797388462	0.26589809313889
C	-3.91395929213493	4.87009554716701	-0.66027485963153
H	-2.13064717453350	1.39322067118783	0.05183391846736
H	-4.42891431368480	2.12167308996791	-0.65518511053263
H	-3.44985594838468	1.72587954458277	-2.07629718469470
H	-2.94422663946601	4.13357752813013	-2.45078039082271
H	-4.69565666720731	3.88091783254232	-2.42079028627356
H	-2.80142735090484	5.48204836026717	1.14109606153051
H	-1.79500476753348	5.15130189032523	-0.30150430089478
H	-4.81287789612324	4.63942245086216	-0.06561351099010
H	-4.04056230214126	5.88971449789582	-1.05097163845550
H	-1.43420896492750	2.67733121538927	-0.97260588601890
O	-2.40918100303400	3.14349606728808	1.92255567398324
H	-2.48108701904526	1.60123285527568	2.39057908327893

93

Entire Intermedia L

C	2.35976396738077	2.51564153217355	-4.89781268311403
C	1.05302525890943	2.94836780940872	-4.70551572056491
C	0.09320220303161	2.06158014844504	-4.22599500314385
C	0.42589885815150	0.74638608833171	-3.92725346858331
C	1.74196776275746	0.30666204719670	-4.10497801736821
C	2.68877759940370	1.19458588311232	-4.60416457166615
F	3.27405218565614	3.35303260099203	-5.35181548474402
F	0.72706944150444	4.20006899118526	-4.97089403929160
F	-1.13803082782469	2.51877754235672	-4.04875588855158
I	-1.07547665109319	-0.48901719889853	-3.16414953214640
F	3.94805048911644	0.80817592329519	-4.77442885756849
C	2.21339089292353	-1.08742620584997	-3.73554604894369
O	2.54011357233691	-1.90822409463271	-4.56263038855988
N	2.23758778173410	-1.28356919416442	-2.39491316246573
C	2.84928490856227	-2.45009609091512	-1.79299021988557
C	4.37212490675989	-2.25977131272736	-1.61332536837211
C	4.67951276090047	-1.01348384218145	-0.81872004440128
C	4.79845549604369	0.22608288053188	-1.46461899997198
C	4.96854683577763	1.40257152330454	-0.73221999625996
C	5.02575105388783	1.35700691918943	0.66208637207170
C	4.93179472420496	0.12692375000928	1.31467924953268
C	4.76265158161978	-1.04821601054169	0.58027856691708
C	2.20665369043230	-2.77692601971061	-0.43565684514132
O	2.79769591417859	-3.46595274949962	0.37884957829467
N	0.99935781230626	-2.22693594296102	-0.19278857965525
H	2.10015003462251	-0.46509696086046	-1.80629430011662
H	2.67635490360594	-3.29856322772812	-2.47237977335263
H	4.76469851329612	-3.15265276362893	-1.11114897951972
H	4.81636834441528	-2.19372767564881	-2.61682801696940
H	4.76256416556461	0.26859609087392	-2.55652533526050
H	5.06289894981824	2.35764379634959	-1.25342078356307
H	5.15796454980599	2.27544254104426	1.23677188023801
H	4.99015894142204	0.08014969183718	2.40384888083540
H	4.67463090668450	-2.00904672655376	1.09080210665278

H	0.50243645974544	-1.70792426639806	-0.91197469210581
C	0.44823530252087	-2.18806594923383	1.14488446927118
C	1.23913344081356	-1.22956875171305	2.06482346293408
C	-1.00580792789730	-1.74080908243138	1.04489638737349
O	-1.43182011815498	-1.15718712039803	0.05666990720706
C	-3.13336690842739	-1.46091066140299	2.15983421537551
H	0.92541503299686	-1.41909875854975	3.10334779984886
H	0.49397656889586	-3.19942291665443	1.57826889069941
H	-3.72995440724792	-1.93128854806717	1.36275708308017
H	2.29479993300194	-1.53461792128716	1.99542607974863
N	-1.77498351785239	-1.95977690435076	2.12759676851272
H	-1.40853346595462	-2.47121977054465	2.92362201809654
C	-3.77362685068544	-1.73800228179343	3.52734416680684
H	-3.22604309076682	-1.16652213583590	4.29203061192480
C	-3.77692652441677	-3.20715332791403	3.87476089601893
C	-2.93572155098515	-3.71169299291428	4.87212190209953
C	-4.60199810784227	-4.09831488778590	3.17535816302428
C	-2.91446085755042	-5.07753424331796	5.16496411078090
H	-2.29625353038661	-3.02486181948865	5.43299945202504
C	-4.58465595838705	-5.46039362344893	3.46647682998775
H	-5.26809220750913	-3.71677033523217	2.39708454590132
C	-3.73828344261656	-5.95455510847869	4.46220268313343
H	-2.25382656825489	-5.45420735932738	5.94762284282814
H	-5.23609536808650	-6.14112518117125	2.91583279196480
H	-3.72505845514622	-7.02150287765000	4.69008865331303
H	-4.80181663263266	-1.34904952437296	3.49190783054502
C	-3.17947010133875	0.04158905077333	1.87842352946306
O	-4.05548938957834	0.55607171586810	1.22931393049449
O	-2.19930127095688	0.69244248086870	2.47216862884019
C	1.09188720151778	0.26761187504589	1.77286075383110
H	1.76060602488390	0.80277601069201	2.46853586304992
H	0.06745517189233	0.58769932414377	2.02087402459338
C	1.41475767179949	0.71460005190333	0.34784010723052
H	2.38479198992038	0.28327069383456	0.03836644866652
H	0.63679340478140	0.35181552074008	-0.34551340758473
C	1.49218778392868	2.22544027997589	0.19237828186639
H	0.56691938969443	2.67699658535915	0.58819324497127
H	2.32183372928416	2.60883782532679	0.81905760581937
N	1.61980503867651	2.57505429365413	-1.22007907436616
H	1.71701468762704	3.58368485581756	-1.33071916390359
H	2.48526380458667	2.17138051312720	-1.58477024024480
C	-3.41970764326448	3.80773867887992	-2.06784591092399
C	-3.22866008027963	2.40170830318835	-1.49929443204917
C	-1.96220653799134	2.31417164620685	-0.62502532197178
C	-2.03247408130618	3.38080601076922	0.43340703570474
C	-2.19977277899967	4.78588927046893	-0.08175458582681
C	-3.46459153029626	4.85525363688125	-0.95675541774828
H	-1.83788487205723	1.31871790918147	-0.17318439328445
H	-4.09404648814763	2.12021332285505	-0.87850793048627
H	-3.15475087123013	1.66254149464298	-2.30861770132530
H	-2.58821548346857	4.03901182238311	-2.75355528594428
H	-4.34517561412125	3.84996289306655	-2.66109076492297
H	-2.24238513349141	5.47700939791723	0.76939895525460
H	-1.31922567999058	5.02759179623443	-0.70057583154970
H	-4.34658060306084	4.67865598341711	-0.31957405610490
H	-3.56485016784102	5.86834104596549	-1.37152072073254
H	-1.07122547661758	2.52150491662610	-1.24790780373866
O	-2.02060539339673	3.15052998826468	1.63262901927607
H	-2.18319508928809	1.65824992901069	2.19564120678569

93

Entire TS11

C	0.01541411259176	0.01832532812826	0.10205917716252
C	0.05594841493279	0.07640180324907	1.48805859349805
C	1.28431698555372	0.12901040205698	2.14538706701737
C	2.47341193310638	0.12734022125472	1.42593446632252
C	2.43922331247450	0.07408268478483	0.02812597868078
C	1.20917825937675	0.01892851550793	-0.61179746346373
F	-1.14467380982614	-0.00726174933993	-0.52965731890088
F	-1.06599000266709	0.08947847627807	2.18381316617012
F	1.27364079435440	0.18501435421672	3.46822511927612
I	4.26931827628239	0.27044649315061	2.48421711717069
F	1.14244449898218	-0.00463304528495	-1.94034770409055
C	3.70017965592864	0.12575275802711	-0.81461100254810
O	4.47662502261004	-0.80002319518829	-0.88214390191368
N	3.84990731943278	1.31849663337929	-1.44145753091078

C	4.93741946392140	1.62267369749300	-2.34863196387714
C	4.43489047779774	1.84232406592827	-3.79051032900093
C	3.40050761019146	2.94159594509559	-3.86677637575044
C	2.04103740863297	2.64850810766096	-3.68955714447983
C	1.08617594855033	3.66752914434491	-3.66020184793944
C	1.47757346659594	4.99921781004464	-3.80576983119495
C	2.82548688303485	5.30077579973845	-4.00838297280675
C	3.77752675463892	4.28117443879538	-4.04284293114316
C	5.74021479493239	2.85901746213190	-1.89529532997578
O	6.42382171845126	3.48727198554829	-2.68587545703691
N	5.60376381065436	3.20673960360079	-0.60111261040324
H	3.10586552140407	2.00498582508188	-1.33105860135014
H	5.61435525447024	0.75525844027450	-2.32986672789366
H	5.30667369666675	2.08911423835628	-4.40917599837557
H	4.00842724814692	0.89398031379772	-4.14566882889794
H	1.72854955102232	1.60880971642103	-3.57057816060022
H	0.03082026247991	3.41840643459461	-3.52941513196621
H	0.73307089761860	5.79679062415193	-3.77969095999146
H	3.13915487772248	6.33771917428665	-4.14321633737957
H	4.83231165907441	4.51807739524088	-4.19128458652692
H	5.05781737117881	2.62575131746179	0.02820414506908
C	5.99172356051873	4.50919159997429	-0.11025270393120
C	5.09623379498884	5.64107070207007	-0.67329064554246
C	5.85736827650428	4.48339822451001	1.40360044823213
O	5.23744980171892	3.57644319409158	1.96448588427080
C	6.25711961999045	5.59034092147322	3.54074179719645
H	5.59412429410555	6.60324971087386	-0.47581488242851
H	7.03648776770538	4.71251318771570	-0.39270555752495
H	6.42145939351669	4.56951151260016	3.91887499309532
H	5.08467789642756	5.51443638686740	-1.76688705873052
N	6.34202909613939	5.53094983756135	2.08370624985137
H	6.85176151834032	6.25912802603861	1.59222972227890
C	7.30481863017020	6.52683482397073	4.12662982606259
H	7.10882145579459	7.55517949594216	3.78772633660979
C	8.70369426918291	6.09243069797734	3.76609659535834
C	9.48407518896252	6.82760784882091	2.86868469339037
C	9.22921528241339	4.91058945351111	4.30635118449411
C	10.76358953019842	6.39372738359602	2.51370672056779
H	9.08872374470210	7.75660178663357	2.44901824289563
C	10.50506917943620	4.47564787513526	3.95471711817213
H	8.62765345868441	4.33273794617824	5.01374751248209
C	11.27620494844028	5.21640300281585	3.05531509496531
H	11.36121496927188	6.98041026816880	1.81383076924395
H	10.90269549095426	3.55557387285505	4.38635125156773
H	12.27579196790938	4.87576120698828	2.78070142982180
H	7.16070242486296	6.51050498992992	5.21581487517701
C	4.84207650993094	5.98243137754710	3.99243120583916
O	4.59205351388757	6.97058975555227	4.62551441142744
O	3.88453072752875	5.12699649394725	3.63632314942467
C	3.66422314399078	5.69159159424565	-0.12794783071022
H	3.14907870239546	6.52978551257213	-0.62580522920010
H	3.67723843611161	5.94040199046102	0.94817327091555
C	2.85516185619221	4.41394383191502	-0.33611957814185
H	2.93775384622562	4.10798944640290	-1.39587097788836
H	3.26821071461512	3.60167133271269	0.28027686954330
C	1.38123958757741	4.51673493937509	0.02184698104154
H	1.28570883891498	4.79704514784545	1.08355189709539
H	0.91098604860477	5.32845888316418	-0.56851040833331
N	0.75255461865710	3.21126840729871	-0.17134073264658
H	-0.20627177734077	3.21900747633762	0.17371536501883
H	0.68476195018783	3.01623204391103	-1.17154467090959
H	4.26984795406491	4.36302104377964	3.14055967472662
C	1.54131115586741	3.97946982518648	6.55559584116975
C	2.32654978147168	5.28862551829156	6.62806845854890
C	3.75667173639899	5.05424901397625	7.13243727046661
C	4.46822683309633	3.96442734852249	6.36261710661778
C	3.67732342970110	2.69776934139555	6.12118068449308
C	2.24328249183705	2.97038031898664	5.64770152942809
H	4.36870980799844	5.96448386081960	7.09317494933668
H	2.37730167674413	5.74552220398741	5.62732560712182
H	1.82288885399280	6.00864925257762	7.28944120918493
H	1.44086841180094	3.55327402126083	7.56951585937232
H	0.52172634448280	4.16943007166571	6.18867028876401
H	4.23859126117308	2.06437654160175	5.42101953334364
H	3.63995993059437	2.17320342273248	7.09323895359276
H	2.27024260516803	3.36787859780002	4.62098759949278

H	1.68774522375741	2.02302584533756	5.60775345751401
H	3.71842494893765	4.70930434004518	8.18205941385701
O	5.61081782416865	4.08699702217032	5.97029716096620

93

Entire Intermedia M

C	0.54695889329761	0.60554711266138	1.09071875281210
C	0.87132664334150	0.37042669488201	2.41584887008562
C	2.19696023094663	0.11486223413795	2.77074627708509
C	3.20197051625478	0.08290275386797	1.81185414415507
C	2.87741556379621	0.29932862058717	0.46026839936777
C	1.55052877516642	0.54701694392717	0.12816419579868
F	-0.69493895023131	0.88143878999531	0.74302508578888
F	-0.06214855773150	0.42279874714091	3.34952368718072
F	2.45137848757193	-0.08696627946369	4.05508256746847
I	5.15904712147611	-0.21202067117129	2.49957822024584
F	1.19407426985313	0.76735179217028	-1.13414488747577
C	3.93844084193357	0.29388204685288	-0.62436129131621
O	4.75614858775770	-0.59377784288489	-0.72170226509837
N	3.90549820133681	1.39534945629173	-1.41638239517663
C	4.92826801977623	1.71328173400588	-2.38963453726953
C	4.35497419576763	1.89014513331687	-3.80741310068702
C	3.27407822430800	2.94450521316474	-3.85600845096554
C	1.93729007445475	2.59957971177062	-3.61460948288168
C	0.94083291312081	3.57757575843865	-3.57994751810010
C	1.26846653616162	4.91899096235390	-3.78374374275546
C	2.59427355853428	5.27191471531267	-4.04255670610122
C	3.58782845175024	4.29290966077041	-4.08087583507738
C	5.70144063499565	2.98516163715938	-1.98244319185643
O	6.35759322277416	3.61536992006641	-2.79397898587545
N	5.56716273419224	3.34795283381802	-0.69239750293585
H	3.15657083008742	2.07100425619499	-1.28059673913153
H	5.63954378708757	0.87410884956247	-2.38883458200704
H	5.18915955020293	2.16390960502662	-4.46554938777745
H	3.95468703260309	0.92050894753106	-4.13395282241846
H	1.67571050995900	1.55107941653104	-3.45461488452362
H	-0.09736351636181	3.28812128197116	-3.40338116537679
H	0.49088438543273	5.68432171447876	-3.75702173641615
H	2.85726230881656	6.31655139950829	-4.21936921806707
H	4.62580997769719	4.56839960676148	-4.27615171345449
H	5.02452991657827	2.77439395714357	-0.05520378920663
C	5.95226175235989	4.64577143863788	-0.19506161959516
C	5.06085928225489	5.77675828884266	-0.76461799863119
C	5.79481957850203	4.60133674804896	1.31794973233693
O	5.11338807622115	3.72255560991914	1.85430706935372
C	6.22580683115788	5.64457887375115	3.47977650974839
H	5.56569152023946	6.73798084252273	-0.58045365369208
H	7.00068124363823	4.85092870692428	-0.46219478044425
H	6.29825829377282	4.60471843092766	3.83583003148539
H	5.04398032174807	5.63618116938296	-1.85651316476681
N	6.32773379131960	5.60678924355157	2.02370857479683
H	6.88628597124870	6.31263603079961	1.55297170305344
C	7.34082644644823	6.47801511224641	4.09681857288284
H	7.25590957107992	7.52047962491948	3.75396957964902
C	8.70060784986438	5.90844580052407	3.77578110660454
C	9.58171286245684	6.56802256937503	2.91387106616230
C	9.08423890291243	4.67459914515275	4.31912341833273
C	10.82248817609767	6.00909615016047	2.59765777103086
H	9.29747405375925	7.53562220967812	2.49183109684869
C	10.32086331869245	4.11503052237280	4.00599634507390
H	8.40327055819788	4.15274910948213	4.99755399437710
C	11.19385810030638	4.78108143870362	3.14196483578402
H	11.50030133212441	6.53780450571203	1.92530569653859
H	10.60744977070867	3.15539218195168	4.43950751791980
H	12.16271363279311	4.34300777491052	2.89703176121202
H	7.16864732953809	6.48261553550860	5.18205795334509
C	4.84086787172170	6.13421442852423	3.92513338283827
O	4.66559937411119	7.06583767367455	4.65965244350881
O	3.81549952100165	5.42692272404444	3.44608252806267
C	3.63431189940771	5.84781401565739	-0.20946559611853
H	3.12536429301152	6.68363236602150	-0.71790245660621
H	3.66278643729514	6.12083138639794	0.86032788044002
C	2.78877334129073	4.58521188219951	-0.37298955805469
H	2.83624701652909	4.24461607863845	-1.42388954494851
H	3.18431659244013	3.77641630270854	0.26043490822515
C	1.32932815028228	4.77395698227943	0.01435408452254

H	1.27851411187321	5.14486542218509	1.05152569795988
H	0.88145477291119	5.55974211628441	-0.62704813465478
N	0.63296298771931	3.49394433919919	-0.05528446132846
H	-0.33293653358670	3.58883475015622	0.25390464560065
H	0.58790878121394	3.18409811004412	-1.02669104162731
H	4.13891694848239	4.64122942857482	2.94256913449528
C	1.11878093227115	3.92128961589103	5.73866155001026
C	1.94833519042391	5.13100333139798	6.16507951667728
C	3.31338018476261	4.69906277605979	6.71115809963475
C	4.05183968358322	3.74916362513998	5.79420954572859
C	3.23170823106654	2.65771532540890	5.13854946575283
C	1.84534980263724	3.11229842629823	4.66479536461807
H	3.97270575130145	5.55134800214331	6.92303555670944
H	2.10592224610656	5.79369596831576	5.30050502133251
H	1.41905489141371	5.71539113027975	6.93149040091997
H	0.92717483389732	3.27787699788414	6.61545806347364
H	0.13723491486150	4.24592094967673	5.36297763303242
H	3.83359602736132	2.21504533691109	4.33111840291024
H	3.10781276535598	1.87820609387676	5.91253760826766
H	1.95009364081688	3.72816031039252	3.75749454548858
H	1.25155546293182	2.22945407924615	4.38849072751323
H	3.16664028146305	4.14360553199818	7.65608053581854
O	5.25427110740949	3.82102716701304	5.64159500959227

93

Entire TS12

C	-0.23110777506821	-0.20642433969233	0.05740983394454
C	-0.28315882748377	-0.54502940121891	1.39991456532953
C	0.89873087008452	-0.63602258610709	2.13801945627656
C	2.13447086425764	-0.41103124915442	1.54181150272150
C	2.20015473961041	-0.10396324421558	0.17124383186434
C	1.01072673841769	-0.02288766171899	-0.54395739252229
F	-1.33916274807689	-0.07934187882730	-0.64693801424667
F	-1.44871624775313	-0.74444164431601	1.98586429815595
F	0.78681870028159	-0.90894250966578	3.42830473877498
I	3.81335946827225	-0.42456645669320	2.79245796874404
F	1.01689731112684	0.23336272991848	-1.84889304404042
C	3.52392401933208	0.09387253619468	-0.54553636885586
O	4.42983541583384	-0.70007870168960	-0.42090093875990
N	3.59570719613716	1.22594467722538	-1.29029711673334
C	4.77850553541611	1.58584867690745	-2.04752567795882
C	4.60796795920829	1.38135339881170	-3.56803332946332
C	3.47544484057307	2.20538985371516	-4.13386424736981
C	2.17967902600016	1.67678155722221	-4.19276469274325
C	1.10241138000149	2.45990377700758	-4.61223448317631
C	1.30950640403875	3.78692322194850	-4.99197128191466
C	2.60155609128785	4.31507257009404	-4.97001247065113
C	3.67541739612457	3.53105087335569	-4.54662679895748
C	5.20559117957455	3.03845236251635	-1.78208277593559
O	5.99878823271788	3.59612060718995	-2.52267976856753
N	4.62359036858399	3.65018820897713	-0.73363348837005
H	2.75617084899122	1.78842993109353	-1.41180102247660
H	5.58738697722270	0.92949626472009	-1.69448599620348
H	5.55959837657283	1.65910001703973	-4.03887464083974
H	4.42859626952988	0.31159761212939	-3.74511362612093
H	2.01723084918833	0.63526928599698	-3.91025524282514
H	0.09993984232005	2.02835895431902	-4.65100444090737
H	0.46993326018900	4.40156632740084	-5.32116082459470
H	2.77605905904751	5.34569278588830	-5.28494787041531
H	4.68328282982855	3.94842148905475	-4.51745537615752
H	4.03532325952859	3.13499318047033	-0.08706345334352
C	4.72787389357661	5.07659820539506	-0.54229823487294
C	3.83446175323489	5.86788109050491	-1.52087355269205
C	4.38853984857432	5.38441526705866	0.91128278043085
O	3.88528294963398	4.55190732051173	1.65303835877152
C	4.62036884376268	6.99705320282085	2.71908648623755
H	4.12193386795522	6.92998131599450	-1.46615299492533
H	5.77312279928265	5.37206414659014	-0.72569916588387
H	5.41638339039361	6.46768501212343	3.26828490445734
H	4.10575350858118	5.52315273058393	-2.53106814357310
N	4.65287546891749	6.64164552624600	1.31751143448312
H	5.16050123808629	7.25950423719669	0.69070126428578
C	4.81295564216887	8.51697115889770	2.87778062758537
H	3.93571027790079	9.02275383723063	2.44709052787999
C	6.07709105333856	9.01149472467918	2.21807635803363
C	6.02511675129596	9.74319155935357	1.02605758817913

C	7.32995661382069	8.70718597646190	2.76806252275091
C	7.19863447792538	10.16257641543023	0.39457255511987
H	5.05307663094348	9.99398843518681	0.59336374944010
C	8.50153106090979	9.12627384678411	2.14156233381630
H	7.38354137522014	8.14000617029795	3.70095861272387
C	8.43872373031049	9.85466905340066	0.95092840029366
H	7.14076013247108	10.73532428573257	-0.53253781602456
H	9.46956425580330	8.88628719888723	2.58454421600572
H	9.35657910457711	10.18314624519688	0.46084517479700
H	4.83221895991274	8.72947930106405	3.95609453237271
C	3.31423838876897	6.63702357271365	3.42530732816210
O	3.30883686825044	6.43818091628611	4.60699883247700
O	2.19061357473168	6.76248239794152	2.70901953448460
C	2.32353387944778	5.74530699949240	-1.30924968553148
H	1.83329072151615	6.34198331133921	-2.09713964541747
H	2.03757753677972	6.21246718904585	-0.35055990348199
C	1.76415560608755	4.32271253867089	-1.34753064684254
H	2.22756835420177	3.76432286512898	-2.18215700929418
H	2.01660578116248	3.79982165366916	-0.40943323709040
C	0.25233001209093	4.26038662907243	-1.51121642153182
H	-0.22237168060546	4.91765107987205	-0.76777344762589
H	-0.01431611306599	4.65430920199082	-2.51201885844562
N	-0.20814311173054	2.88975497222853	-1.28975011237087
H	-1.22404684955184	2.84009958352549	-1.35838264229493
H	0.14628480597826	2.29609208141576	-2.04121713533981
H	2.00031468115173	6.01360403898951	2.09929891251920
C	-0.68739096650060	2.05851859096087	3.81148516469138
C	0.79845348545946	2.38877237051319	3.67026941537206
C	1.15625079381652	2.84450849988976	2.24417307231261
C	0.23344209948263	3.94707586359064	1.80214127441505
C	-1.23503270085630	3.61752097533544	1.89609073939531
C	-1.56749223957166	3.21750463393366	3.34411023849941
H	2.19719526796484	3.18255257199885	2.16418400997504
H	1.06209752608633	3.19845727788992	4.37074699957038
H	1.41860690521665	1.52232153673242	3.94535245263456
H	-0.92832398273732	1.16376157199114	3.21594152322662
H	-0.91734648287480	1.80562252808277	4.85700207328797
H	-1.82266103080844	4.48149704292391	1.55903823871811
H	-1.42423053814047	2.76389391087463	1.22291512271970
H	-1.40823858664256	4.08844117579152	4.00090328801095
H	-2.63166507655249	2.95159897655079	3.41624292047936
H	0.99018952004612	2.01982951651350	1.52937213475698
O	0.62927676305874	5.04161513056378	1.44804468172651

93

Entire Intermedia N

C	0.96740838484977	2.95234504196135	-3.90699431720286
C	-0.36711302085030	3.17672982190166	-4.20256268042220
C	-1.22446850151205	2.09202091426967	-4.39764164191809
C	-0.75705156383523	0.78602955215427	-4.30903288359580
C	0.60069030076220	0.55051098216851	-4.03624833321010
C	1.43724993545029	1.64410483577331	-3.85023314688018
F	1.78566812370819	3.96381632998295	-3.68682927327745
F	-0.83028481869907	4.41236326788420	-4.27785130179947
F	-2.49554403319904	2.35575082074363	-4.65525597347198
I	-2.16864591728465	-0.74834153180189	-4.50637078365351
F	2.73106147644738	1.47548457897716	-3.59262513874245
C	1.16656121551862	-0.85480205288873	-3.94959040795752
O	0.98305106639679	-1.67273217193106	-4.82370576933497
N	1.84153519381616	-1.09753156723628	-2.80002250352473
C	2.39033957329288	-2.39296821530070	-2.45818777764751
C	3.93251428241415	-2.40544661165538	-2.46465732368914
C	4.51137936390129	-1.36868694998356	-1.53068173380767
C	4.77082574054107	-0.07102528088433	-1.99082779124278
C	5.20619762473150	0.92687090874547	-1.11647415353613
C	5.39630862086231	0.63782940940150	0.23593330451440
C	5.16957671758313	-0.65913276310274	0.70035835666453
C	4.73368997745407	-1.65420721804031	-0.17559565019174
C	1.88220041704366	-2.86714307734158	-1.08344231332296
O	2.46043942413830	-3.75358260569615	-0.47672736636645
N	0.80810583266700	-2.21692138666272	-0.59708264969378
H	2.00396901666346	-0.32470984012500	-2.15829992326773
H	2.02455568290325	-3.10014252858185	-3.21733858049419
H	4.25127355532177	-3.41307676567098	-2.16967422255565
H	4.26658125807189	-2.21891345758525	-3.49481847086650
H	4.62706667145829	0.15785004844258	-3.04844254141774

H	5.40444239604321	1.93166828070658	-1.49599567551529
H	5.73548594389211	1.41611687871613	0.92181324470823
H	5.33276238400462	-0.89911834656380	1.75280369384404
H	4.54521983709336	-2.66549895084350	0.18926548575546
H	0.32044357364506	-1.52660028700181	-1.15932802286249
C	0.41788291688193	-2.30304845351285	0.79085553458888
C	1.45372153195733	-1.62876710077494	1.71931947158815
C	-0.94046266324629	-1.62005960456921	0.92727091752110
O	-1.38747618227849	-0.89441401919764	0.04920072157353
C	-2.83855638811339	-1.14300353395337	2.36389565116613
H	1.25633801959540	-1.95678210766190	2.75201358255939
H	0.32695532419160	-3.36269765300813	1.07811188593177
H	-3.62026759512399	-1.50834161606427	1.67934004248682
H	2.43437864884626	-2.04480783049357	1.44273731653084
N	-1.58017374781014	-1.80623957201986	2.09751852972685
H	-1.20562914495532	-2.45254679034486	2.78447251888762
C	-3.26408147738196	-1.37746104606846	3.82161319717164
H	-2.52853693019129	-0.89180854168197	4.48022986156888
C	-3.38065634549768	-2.84335259308309	4.16197173324814
C	-2.43754865164109	-3.46394520330191	4.98840462258696
C	-4.41893460229982	-3.61659891126359	3.62529380868961
C	-2.52616206835415	-4.82870183807601	5.27335618117279
H	-1.62971827725401	-2.86841812167776	5.42200870082671
C	-4.51110962857596	-4.97716366956758	3.90949957962826
H	-5.16458837330664	-3.14270900339401	2.98156013578170
C	-3.56310225564762	-5.58776979198181	4.73451424410927
H	-1.78387451568567	-5.29677990007489	5.92201784225555
H	-5.32797040028864	-5.56522916256410	3.48777756527947
H	-3.63627620267576	-6.65330398554930	4.95757723644103
H	-4.22977913109158	-0.87134902802285	3.96654081472066
C	-2.74442447012412	0.36185286158705	2.11863106448015
O	-3.63600195664686	0.99381581327358	1.60906920936614
O	-1.62675466067940	0.88473316061523	2.58699253811926
C	1.48508928093497	-0.09744105180807	1.68610559788627
H	2.30126364623530	0.22924322968874	2.35274962925709
H	0.55208357646046	0.29141654063686	2.12447762112575
C	1.68936487771902	0.54726991949103	0.31547791366120
H	2.57910176141767	0.10777980771825	-0.17239777141921
H	0.81709602875752	0.35429487916076	-0.32977545897740
C	1.86846622332865	2.05648750164999	0.38325124489215
H	0.97391231950288	2.50330634309854	0.84966323163840
H	2.72424425871519	2.29127440366004	1.04746410166309
N	2.01283394470494	2.60108261964713	-0.96325698966193
H	2.10878739949060	3.61509226860313	-0.93522854451657
H	2.87478375127363	2.24750207127709	-1.38140406291726
C	-3.23905641207298	3.52021304955172	-1.93153469439048
C	-2.77117933417223	2.19599646724975	-1.32961031491788
C	-1.52602404405630	2.37377435909634	-0.44396703768099
C	-1.72384653799221	3.48831051990011	0.54944550803125
C	-2.20921961616810	4.79565260922793	-0.01793412807003
C	-3.48631514919090	4.55965325529486	-0.84089333315146
H	-1.26991712112303	1.44114909858261	0.07682160109174
H	-3.57316342634738	1.76821964970863	-0.70913715456477
H	-2.55521122390406	1.45763404910208	-2.11613197175105
H	-2.47733080132668	3.91101157132389	-2.62580011174022
H	-4.15191333033963	3.36084763487132	-2.52368498379457
H	-2.36070707204343	5.50539097767019	0.80508941561318
H	-1.42025456412350	5.18487308734223	-0.68451690294852
H	-4.28606088336155	4.20683612437018	-0.16914743420501
H	-3.82257132146607	5.51286966209354	-1.27262758356232
H	-0.65204855390573	2.65856462955963	-1.06345491293336
O	-1.52688919646315	3.35848477966100	1.74792865624991
H	-1.56828617332440	1.85489263968521	2.33509154643321

93

Entire TS13

C	0.86668727188206	3.05520318997741	-3.71137949001348
C	-0.44552303675941	3.26387446935150	-4.10486074667784
C	-1.26792825955706	2.16952614417118	-4.38127030499982
C	-0.78468562461907	0.86993693405618	-4.28262517405349
C	0.55890244048976	0.65054573691458	-3.93263766543010
C	1.35989042154880	1.75446708501643	-3.66103472559198
F	1.64318436801067	4.07720577334719	-3.40610294627282
F	-0.92092071979718	4.49428041884532	-4.18791526747424
F	-2.52124632195918	2.41614808283118	-4.72838124542472
I	-2.16180357767765	-0.67862695263411	-4.58624126718912

F	2.64168105729593	1.60410174978493	-3.34002036079767
C	1.15521473743247	-0.74547658442952	-3.90220859651517
O	0.98893730537936	-1.52244311635448	-4.81666296230585
N	1.84112454542915	-1.03188748019663	-2.77027151608952
C	2.41313657610342	-2.33492209934468	-2.49990612906247
C	3.95499684920123	-2.32029632187624	-2.50624462863644
C	4.51655712684583	-1.33072467646936	-1.51272980807964
C	4.74567069679119	-0.00199324289395	-1.89306271383496
C	5.16331710076225	0.94936104544121	-0.96030319037197
C	5.36743220382079	0.58106932609353	0.37057045869590
C	5.17081246878226	-0.74653566738185	0.75513810494308
C	4.75091308621035	-1.69457607789050	-0.17865377984669
C	1.91239283387082	-2.89234557403768	-1.15374941644908
O	2.50145298580894	-3.80402080318180	-0.59676300166177
N	0.83253012414759	-2.27879009983794	-0.63645306018208
H	1.98084077191506	-0.29591349868282	-2.08162324276463
H	2.06044292751911	-3.00549549444468	-3.29728034288366
H	4.29097710369316	-3.33817057958583	-2.27139254550365
H	4.28496650376101	-2.06761455993346	-3.52362296170025
H	4.59067829984706	0.28853869083840	-2.93395598953567
H	5.33795287630403	1.97978123835621	-1.27742373939370
H	5.69393751508473	1.32252582459163	1.10188692200392
H	5.34522423462175	-1.04745048333193	1.79000052625412
H	4.58519800455546	-2.73020786588303	0.12367998369926
H	0.33041678109145	-1.57745125739179	-1.17185139681870
C	0.42366467372823	-2.43629200239817	0.73921396991610
C	1.47178557404655	-1.86163823556194	1.71884452656517
C	-0.92244270844495	-1.72600172230505	0.88729431653839
O	-1.39568353362305	-1.04617502236287	-0.01354854037640
C	-2.77205155305953	-1.15305662179453	2.34364707698235
H	1.26456786419732	-2.26333109998215	2.72351308759773
H	0.28872818619296	-3.50756961293109	0.96193919178603
H	-3.57136875841212	-1.53861035577072	1.69119076175206
H	2.44092586257567	-2.28446644384705	1.41457148641392
N	-1.52954917822951	-1.84640713963733	2.08289919203228
H	-1.13378815613564	-2.44696603256016	2.79877666566149
C	-3.17622943785990	-1.31531500044041	3.81676549905475
H	-2.42542352795026	-0.81014066823217	4.44299647247194
C	-3.30421440631384	-2.76333623365658	4.22358339320867
C	-2.36357741149798	-3.35506007734901	5.07348459348095
C	-4.35089656546099	-3.55052821231185	3.72475461148723
C	-2.46266770512309	-4.70528719213340	5.41828933841831
H	-1.54879823408227	-2.74823558504155	5.47734326504068
C	-4.45331583132300	-4.89658560819535	4.06829648843993
H	-5.09488784607472	-3.09923364567129	3.06311447979224
C	-3.50754910186373	-5.47862016099369	4.91624504713173
H	-1.72196477441864	-5.15078150998619	6.08443450274245
H	-5.27646853834000	-5.49552027603188	3.67501752735386
H	-3.58864118655688	-6.53281990708355	5.18564245008022
H	-4.13324277414591	-0.79061860069415	3.95214598246930
C	-2.66932285969457	0.33800100566103	2.02520237434955
O	-3.59670040706229	0.96376963597798	1.57428132388658
O	-1.50542881246330	0.85916402058104	2.35898688813246
C	1.54344999217055	-0.33357489021807	1.80259280691421
H	2.40257243395810	-0.07544715675681	2.44493629100473
H	0.64391494095852	0.04011861804928	2.31517908567883
C	1.68355317647166	0.41074171933930	0.47432720412458
H	2.55325023931850	0.01502767752089	-0.08237173194636
H	0.78530103595774	0.25662235761006	-0.14523221936326
C	1.85327786868205	1.91262826165665	0.64739027928460
H	0.99477366767176	2.30499487005247	1.21589340137022
H	2.75770022302591	2.10578547447636	1.25871006024743
N	1.88631611785560	2.57236417513208	-0.65668645185696
H	1.95178534595926	3.58292748745008	-0.54153393164414
H	2.73470322532878	2.29494252078106	-1.15255169475793
C	-3.37697070922668	3.63584268492029	-1.99954541838497
C	-3.03288162953030	2.26230310647166	-1.42092949268110
C	-1.66123036146375	2.24517208014559	-0.71773890691456
C	-1.55197100661747	3.40441268017787	0.23499457384054
C	-1.81866492868176	4.75539149655959	-0.36938322652139
C	-3.24335616665974	4.73994570900846	-0.95370380845786
H	-1.48489908892440	1.29260012812528	-0.20046594377206
H	-3.79309359797166	1.96951114909223	-0.68100199740887
H	-3.04289688969733	1.49467835728978	-2.21051774335825
H	-2.70721906751679	3.87276972347135	-2.83915967948079
H	-4.39714647467367	3.61910061097519	-2.41049481951529

H	-1.68751162422033	5.52818394175529	0.39825037782992
H	-1.09586585193480	4.92112290383938	-1.18687920150629
H	-3.96299425461949	4.57232777814995	-0.13549942605132
H	-3.47077248514193	5.72321943922095	-1.38911948460683
H	-0.84926909020174	2.38379794805225	-1.45414230661923
O	-1.32841648112595	3.28361018497468	1.43023720587039
H	-1.45425808959129	1.81892199359426	2.06086444623696

93

Entire Intermedia O

C	0.78819567910998	3.13475469893010	-3.68471974645804
C	-0.48772061192776	3.33573286782284	-4.18655836864760
C	-1.27483738984699	2.23626662670171	-4.53501049657977
C	-0.79121316971072	0.93958064517025	-4.40217421795438
C	0.52113615849065	0.72736838144592	-3.94366011618866
C	1.28338213886260	1.83705178834350	-3.59259673952896
F	1.52270085808900	4.16169321026855	-3.30068977791902
F	-0.97019207278384	4.56060265575013	-4.28723341049411
F	-2.50217633353231	2.47829211082147	-4.96557527502987
I	-2.13258659340398	-0.61635512926616	-4.80870088451318
F	2.53360857682925	1.69865047650424	-3.15923269152140
C	1.13724035305192	-0.66142950472275	-3.90966235918455
O	1.02849007200388	-1.41366692777794	-4.85310300968186
N	1.77755255679776	-0.97049529781884	-2.75722029788501
C	2.38416409304815	-2.26524406208465	-2.51782922175481
C	3.92591114382545	-2.21121797469591	-2.51656098523683
C	4.45815141653119	-1.24684834828585	-1.48319834735219
C	4.65250899469218	0.10109650966236	-1.81122747869988
C	5.03452589300446	1.02864822977491	-0.84025709202381
C	5.24366871671872	0.61515774531958	0.47631521220202
C	5.08549082716661	-0.73154779113809	0.80859022696292
C	4.69677157231927	-1.65485597674569	-0.16282883413174
C	1.89715753843564	-2.87411590338932	-1.18987360848828
O	2.49898619437547	-3.80003477277448	-0.67109651353430
N	0.81936785186286	-2.28616619098579	-0.64169381096881
H	1.88218040494030	-0.25390739909965	-2.04263755152228
H	2.05383559613425	-2.92331920880042	-3.33471794600272
H	4.28538525382978	-3.22898694788292	-2.31880314979382
H	4.25320690792060	-1.91280201900557	-3.52248134909580
H	4.49920225889673	0.42520736932590	-2.84215571589575
H	5.17869411155033	2.07539721159291	-1.11599419370942
H	5.54376530368184	1.33721664415063	1.23780571452388
H	5.26475748057105	-1.06670943599123	1.83210015154002
H	4.56011255047350	-2.70565748305046	0.09904931746998
H	0.29220988251613	-1.58719588189688	-1.15578753250754
C	0.41017872103320	-2.50676329043158	0.72520444278724
C	1.46052370926272	-1.97904668114400	1.72750819311611
C	-0.94495340699901	-1.81699184750534	0.89332102104934
O	-1.45915492181950	-1.18062624770399	-0.01672068997509
C	-2.75475364493794	-1.20629641278365	2.37767233667677
H	1.26437405767692	-2.43409319026458	2.71166531324165
H	0.27711528230741	-3.58778135940259	0.89903806391751
H	-3.56960194059091	-1.61042325265288	1.75663793553644
H	2.42705486822722	-2.38574549533609	1.39508340532707
N	-1.51960530558350	-1.90944273680673	2.10712007910335
H	-1.08632433607721	-2.46094291947986	2.84030074810531
C	-3.12560048548578	-1.32466113670455	3.86292969200563
H	-2.35885289844518	-0.80756086290071	4.45957827078394
C	-3.25342123480255	-2.76078607185607	4.31047612909391
C	-2.30533522698243	-3.33398781868341	5.16459789663398
C	-4.30895484217189	-3.55651515673282	3.84480493456645
C	-2.40570415721371	-4.67434574824096	5.54585072337293
H	-1.48332012361966	-2.72036445908295	5.54270700993894
C	-4.41251110190756	-4.89272835963795	4.22436177391683
H	-5.05910054785819	-3.11980259668667	3.18036628432014
C	-3.45908428318061	-5.45642192929266	5.07608548823713
H	-1.65896209589314	-5.10532968017862	6.21483213791065
H	-5.24247015732705	-5.49829526654876	3.85636341755875
H	-3.54083420568750	-6.50297128223250	5.37367166542810
H	-4.07631962998793	-0.79029909161622	4.00494489853400
C	-2.65602661209610	0.27356230185594	2.00458881776767
O	-3.59803831622252	0.88749909070470	1.56795666023665
O	-1.47580499141702	0.79499123837682	2.26874075207845
C	1.53515475758498	-0.45725725754591	1.89106002068866
H	2.42489519353685	-0.23176952292647	2.50335351468521
H	0.66057033183166	-0.11246980096055	2.46290811029309

C	1.60545153265951	0.36413564188776	0.60260674945638
H	2.43722198084799	-0.00038105857384	-0.02837622988281
H	0.67013760222764	0.25262147619528	0.02860233334891
C	1.80119047828255	1.85031985094471	0.86343444149855
H	1.02740712100972	2.19799509893099	1.56476501250842
H	2.77899064489254	2.00104808271567	1.36250658341818
N	1.66681071441791	2.61080589046096	-0.37907544733515
H	1.76324517196780	3.60813001195582	-0.18932865722283
H	2.44199065123370	2.37333310142632	-1.00023098123778
C	-3.27670251483476	3.73747917461916	-2.01090951228435
C	-3.13830845967453	2.35065150579845	-1.37885917527201
C	-1.70626995603513	2.07368789325027	-0.87414895352985
C	-1.28505454510851	3.22104252408137	-0.00023337443000
C	-1.27865862907786	4.55970957991559	-0.68426564622252
C	-2.73365615143324	4.84649444844702	-1.10972196761835
H	-1.64768812979674	1.11790029931959	-0.33758309794834
H	-3.81789059970121	2.25746813187564	-0.51800976943734
H	-3.42213155605230	1.56888275299436	-2.10017318678133
H	-2.74121125648542	3.76747536737435	-2.97032982922147
H	-4.33341521002320	3.93176971094166	-2.24696858186117
H	-0.89542714851172	5.32354407836402	0.00422586823871
H	-0.63181741833306	4.51099989740899	-1.57522346493262
H	-3.35324548721081	4.92447513814991	-0.20122869021136
H	-2.78149404681340	5.82005196040620	-1.61815841950441
H	-0.99989816682839	2.03900809699462	-1.71951991327508
O	-1.11525426440360	3.13192966010381	1.20683011084459
H	-1.40725478274797	1.73477219093583	1.91020568989385

Pathway 1

93

TS14

C	0.59828498694013	3.00401348123535	-4.25593402835841
C	-0.75187367937742	3.14504554778370	-4.53747147093576
C	-1.57981107791294	2.02163485075825	-4.54190421352144
C	-1.06003297339233	0.75042771307878	-4.32128289560488
C	0.31655926931457	0.59177562372932	-4.08937422386907
C	1.11755380676460	1.72864167826329	-4.04703083498422
F	1.38078500458732	4.06547557696976	-4.19362189753533
F	-1.25581743094025	4.34479531312029	-4.75360133819761
F	-2.87109818162175	2.21962598793896	-4.74172703256790
I	-2.41552866482619	-0.84057802464492	-4.23751984381097
F	2.42243975422489	1.63750706263724	-3.79938971504242
C	0.95134274495024	-0.78206448664783	-3.96841695631822
O	0.75157824209174	-1.63356469685162	-4.80566770466237
N	1.72110078631052	-0.95604111264293	-2.86638969827229
C	2.40081185001454	-2.20049901292922	-2.56868670619172
C	3.93252715405004	-2.03138771863036	-2.52245033362925
C	4.36316148216669	-1.03782985618976	-1.46906113648245
C	4.45105867435911	0.32756947769940	-1.77378438496100
C	4.76865609208717	1.26379019095079	-0.78757029675460
C	5.01526268600506	0.84408835426330	0.52039336012187
C	4.94972308031496	-0.51527681736683	0.83188200376870
C	4.62559581228901	-1.44805298835987	-0.15404508359415
C	1.92638320906909	-2.81705711806112	-1.24053739024729
O	2.54614477805643	-3.73532305091974	-0.72819866999743
N	0.84607358080687	-2.24332534653740	-0.68567086742371
H	1.87489455918928	-0.16894605007165	-2.24123760440053
H	2.14127333485649	-2.90181505042139	-3.37454152846009
H	4.36084966200985	-3.01982918397624	-2.31402267427872
H	4.26894650557568	-1.70615473396011	-3.51684421831329
H	4.27955334509585	0.65680080734768	-2.80029057967413
H	4.83626377648535	2.32235541447900	-1.04677124125926
H	5.27212368518505	1.57213868052991	1.29210461387486
H	5.15531041296073	-0.85336902408145	1.84941180533173
H	4.56640233330267	-2.51059016262398	0.08978980997189
H	0.29607607708612	-1.56247798447087	-1.20114161306146
C	0.40113371968581	-2.52956692404425	0.65783942440260
C	1.41525087059404	-2.03688280134855	1.71313720810681
C	-0.97738235518413	-1.87789392534456	0.80565301443347
O	-1.50687835441102	-1.28495415912627	-0.12534401357737
C	-2.79330131704180	-1.25936881742963	2.27552607624788
H	1.22079108720321	-2.56342337845185	2.66131420822639
H	0.28343342404485	-3.62000001295993	0.77738282772299

H	-3.60185367531321	-1.68004049800439	1.65767143211992
H	2.39940242159725	-2.39001578064560	1.37189222340308
N	-1.55319246528366	-1.95943727396006	2.01711721465866
H	-1.09289142594162	-2.45932414933611	2.77041544934167
C	-3.16467956891011	-1.36503403008783	3.76104964372909
H	-2.41348142548064	-0.81825593924961	4.35144467061713
C	-3.25490278154929	-2.79599732694120	4.23346789148028
C	-2.29993553184433	-3.32545114735737	5.10808978391935
C	-4.27815093180447	-3.63331651860769	3.76848233536313
C	-2.36160311779694	-4.66210198261758	5.51006255581104
H	-1.50246203473323	-2.67969458636180	5.48539298591892
C	-4.34332443771003	-4.96600474544584	4.16842017590566
H	-5.03278903272877	-3.23204649596223	3.08706834144003
C	-3.38303819162618	-5.48545172237338	5.04039020687536
H	-1.60955578539503	-5.05794559247781	6.19473172973764
H	-5.14836350827709	-5.60409738367650	3.79992270364828
H	-3.43441482089012	-6.52945805231302	5.35340438357540
H	-4.12866321968202	-0.85217277238643	3.89163546342617
C	-2.70565146878587	0.22302065591387	1.88750894936784
O	-3.68266623529505	0.81164864562624	1.48302673569015
O	-1.53111934447491	0.75154044378551	2.10200421633933
C	1.43666449546098	-0.52825141750611	1.97948260629327
H	2.33288143670486	-0.30304669519668	2.58210290346388
H	0.56147156390481	-0.25287405087575	2.58657342440342
C	1.42860568074373	0.37083636682342	0.74333096857105
H	2.25529524790968	0.10072783686211	0.06199940289698
H	0.48402603033294	0.23060358708420	0.19587564786780
C	1.54398833257528	1.84151969449886	1.11238535731707
H	0.84448233381996	2.08525289613499	1.92914544043847
H	2.56389283725026	2.06235639060040	1.46668538290934
N	1.18382947610765	2.70083371316854	-0.01830426187061
H	1.59879221549850	3.62777968911420	0.07845240560190
H	1.51555639008385	2.31524949276965	-0.90568497875090
C	-2.88128118220283	3.89002189580400	-1.80772625197125
C	-2.77370365668703	2.44988029944399	-1.30011332485177
C	-1.32593454947167	2.05516987090812	-0.95347414007069
C	-0.79428476177200	3.09504744681805	0.01848965254644
C	-0.74967511497322	4.48407140153129	-0.59315027724217
C	-2.20623610050888	4.88873982752812	-0.86558116571673
H	-1.30740480632760	1.04770387443020	-0.51241219083919
H	-3.38163317500843	2.32361102587645	-0.39121836575655
H	-3.17406223563811	1.74815442639422	-2.04939652874436
H	-2.41340784497767	3.97287839334487	-2.80074029270271
H	-3.93969100960540	4.15640818690418	-1.94585940907448
H	-0.27105234641979	5.17874033874001	0.11244717279440
H	-0.17912674872373	4.47196396867802	-1.53765125723545
H	-2.74059463692293	4.91164894671314	0.09746205956862
H	-2.24351869928923	5.90430303754199	-1.28594394090403
H	-0.70188905156470	2.05738672752792	-1.86090216311993
O	-1.09768191357629	3.01837432191568	1.23866225296536
H	-1.43927937776768	1.74613143620804	1.73839962262284

93

Intermedia P

C	-0.25369889272097	2.41853419299110	-4.64506587196203
C	-1.63079555800306	2.25885672990473	-4.71084436288441
C	-2.19451124461010	0.99974731658427	-4.50736056395694
C	-1.39140229308273	-0.11237033088325	-4.28393269160111
C	0.00259974033787	0.03819209873547	-4.24448132708671
C	0.55001341879663	1.30383160634633	-4.41711394140363
F	0.28172415632497	3.61885422992998	-4.77682623541006
F	-2.40781087631852	3.30392299885484	-4.91898994202330
F	-3.51249163715453	0.91364710242963	-4.51464444159924
I	-2.34020762282951	-1.94482270171880	-3.94627523827802
F	1.86744527152571	1.48723769401027	-4.35769806076118
C	0.91844942948872	-1.15099689817486	-4.04172864603082
O	0.93763258125862	-2.07466660440014	-4.82343953713775
N	1.66526246232121	-1.08382664446400	-2.91140204479189
C	2.53656389677332	-2.15987280167110	-2.49197238131715
C	4.01350113716377	-1.73375193512299	-2.43393793740911
C	4.26593107780919	-0.62640503514041	-1.43932283921909
C	4.07632786450264	0.71665994567889	-1.79621972180593
C	4.29855823047346	1.73965560546831	-0.87060153254431
C	4.71337034664270	1.43342329695222	0.42727090115202
C	4.89861706793477	0.09918134350696	0.79482434960516
C	4.67443682483616	-0.92103588187996	-0.13059224033959

C	2.11938723977286	-2.76536276747760	-1.14006185715057
O	2.84768797275979	-3.57061121390927	-0.58159712810106
N	0.95439947659250	-2.31770770403066	-0.64478546350157
H	1.59701577047021	-0.25495063130971	-2.32674765415751
H	2.43080965735747	-2.95357716555366	-3.24543235787949
H	4.59688661270455	-2.62229296729801	-2.16178341848837
H	4.31317701319515	-1.41651674871208	-3.44270548184171
H	3.77458903248765	0.96347684606021	-2.81716539381845
H	4.17588756965111	2.78165801880042	-1.17595668520818
H	4.90774632861863	2.23239695378719	1.14564604825630
H	5.22846088181737	-0.14907855663083	1.80531880537540
H	4.81735879234991	-1.96558821945758	0.15475709390720
H	0.34516360272853	-1.72890641231006	-1.20831586642308
C	0.41747104686613	-2.65165935973111	0.65531095831233
C	1.35924215409345	-2.24398321277168	1.80707164426803
C	-0.95284518413521	-1.95184274393692	0.72165011879022
O	-1.38927249144822	-1.33635620804728	-0.24487226988796
C	-2.81916821937962	-1.24991583809843	2.10226830644106
H	1.09762233501912	-2.83702197190014	2.69763322672214
H	0.24903496761102	-3.74100779930501	0.71836297753475
H	-3.63444285760903	-1.64193494526415	1.47471783904410
H	2.36412666699716	-2.57689155246347	1.51153598846080
N	-1.60779641012115	-2.02103179840245	1.88724948185011
H	-1.22133621988971	-2.56729173940616	2.65021551405943
C	-3.21752049511209	-1.31742732015899	3.58209880633091
H	-2.45600438529585	-0.78172135398160	4.16945315899632
C	-3.36958735809601	-2.73038831726622	4.08994983560937
C	-2.43102429074806	-3.28510412391940	4.96752293452752
C	-4.43323532590942	-3.53207728149261	3.65264757810403
C	-2.54782707209272	-4.60907161151521	5.39875701135888
H	-1.60235822877524	-2.66714574891530	5.32423780882804
C	-4.55421939797889	-4.85169874846136	4.08200357187838
H	-5.17478546642124	-3.11112636223489	2.96880267727577
C	-3.60967196278602	-5.39569128165386	4.95642696450278
H	-1.80758801457420	-5.02355422853453	6.08543186894803
H	-5.39078844458638	-5.46059779231935	3.73473267146524
H	-3.70489755350034	-6.42939185467168	5.29267409667207
H	-4.16506289432990	-0.76830710441126	3.68394417428078
C	-2.63422098199675	0.23977136198578	1.71413653463725
O	-3.58132530113810	0.81073143852292	1.18602079831695
O	-1.51541839849215	0.75209535721621	2.04716547582800
C	1.35955430753675	-0.76122658884354	2.18696556820140
H	2.23519306561664	-0.57221878718827	2.83080586052719
H	0.46023421636187	-0.52907627244576	2.77713693979746
C	1.36859897767063	0.22300568531109	1.01813773059333
H	2.22696003175343	0.04622798195073	0.34844414957763
H	0.44943335237959	0.09347125046321	0.43527041572889
C	1.41251249219773	1.63744806801916	1.55731175655254
H	0.63805852378484	1.79386013428460	2.31752374993968
H	2.39355625788521	1.85218412457041	2.00305171480981
N	1.17829823906089	2.66485446594549	0.50205916638814
H	1.61747239169267	3.53973628871652	0.80668571678192
H	1.66087701960979	2.39582965447505	-0.36464141949597
C	-2.21246534119208	4.00198919581182	-1.85562314307511
C	-2.28214718366088	2.58007823184919	-1.30133881747336
C	-0.90902560577932	2.07454221174973	-0.84177293140869
C	-0.33509392001003	3.05436062726976	0.19174577877834
C	-0.23606744679561	4.46660247249415	-0.38924670332709
C	-1.61457382624753	4.95605985404058	-0.82502109305345
H	-1.02214044063020	1.07191787598002	-0.40607916880265
H	-2.96080563936061	2.53616887185156	-0.43583135677720
H	-2.69779079195833	1.88576330057173	-2.04726421291278
H	-1.58084225642619	4.02428538850328	-2.76081242707458
H	-3.21279872926118	4.34103487583964	-2.16209904765179
H	0.18428145404414	5.12854509110212	0.38555086069160
H	0.45126361480137	4.46086714228246	-1.25337375902311
H	-2.26388889784316	4.99878178702678	0.06257955472226
H	-1.53604731541035	5.97555553712213	-1.22909687731028
H	-0.21945167029504	2.00833639604855	-1.70491199802899
O	-0.99931256857390	3.07964726842369	1.36211993919717
H	-1.31191985909778	2.11118324801639	1.64441896580855

93

TS15

C	-0.97874412135417	2.24932082320493	-4.81990278510987
C	-2.31606194538232	1.87974301215672	-4.84428029610900

C	-2.67907605454341	0.56800297829119	-4.53273012597135
C	-1.71346224835685	-0.37478722524518	-4.19711556319865
C	-0.36109995583169	-0.00482834644951	-4.16364204973707
C	-0.01755201489271	1.30926392405216	-4.45503107770598
F	-0.62609407340053	3.48832429699963	-5.11037570259640
F	-3.23987414007626	2.77098111096337	-5.14661641280473
F	-3.96351921100099	0.26210034966564	-4.56026469403615
I	-2.35359680904050	-2.30936129578761	-3.72910882787300
F	1.25334166529956	1.70247524039555	-4.41283525651313
C	0.73126904625839	-1.03307308562977	-3.94375150742705
O	0.89262889468544	-1.93444198943293	-4.73581528617942
N	1.46698879435473	-0.86515889803188	-2.81618647972677
C	2.48630283020047	-1.81791471655276	-2.42284302960359
C	3.91438868384712	-1.26522756032669	-2.53313366291707
C	4.17584446312222	-0.06012173931671	-1.66406927193335
C	3.78716132544392	1.22144845649429	-2.08098759178859
C	4.05046405558911	2.34074731660424	-1.28766284748415
C	4.70117879050028	2.19217984722411	-0.05863061628298
C	5.07906079981665	0.91858806475381	0.37124764059721
C	4.81792969915186	-0.19696540452822	-0.42591427994789
C	2.23132296117679	-2.42103865801050	-1.02555589664628
O	3.13041673619473	-3.00961195352958	-0.44572474955563
N	0.97643968203736	-2.25889993073918	-0.57835259444694
H	1.27807229745922	-0.06291505376703	-2.22069213440764
H	2.39791642164235	-2.65541256397350	-3.13112725855733
H	4.59621363808337	-2.07850326190527	-2.25594434910276
H	4.09042926794112	-1.01116673787969	-3.58825258208675
H	3.29846233691518	1.34618953777840	-3.05022321813802
H	3.76673086056062	3.33461377091600	-1.64303210975944
H	4.92566976732627	3.06801261930523	0.55427948120945
H	5.59024067999625	0.79376850685081	1.32725256056554
H	5.10845649869956	-1.19434752565924	-0.09072046197249
H	0.31450574576036	-1.75637380215749	-1.16686906034745
C	0.38110653921364	-2.68247399048721	0.67385751235997
C	1.26453851040037	-2.47156853515724	1.92023042993431
C	-0.95937225431522	-1.91694986441609	0.72909377244393
O	-1.33276771394656	-1.25059517396956	-0.23085193872790
C	-2.85097132732212	-1.17297039787409	2.03298293169362
H	0.89172127470559	-3.14049879278172	2.71200359891045
H	0.13553974107833	-3.75834576882991	0.61835887958802
H	-3.63730370418235	-1.48772974391986	1.32988959327658
H	2.26539391565795	-2.83802401559532	1.65848842392972
N	-1.67329667868089	-2.00495080239357	1.85737155568834
H	-1.32709844170426	-2.55676984091135	2.63521272667422
C	-3.35749646847505	-1.28450725511227	3.47557414456804
H	-2.60544858572162	-0.83380606245089	4.14177632053272
C	-3.64044967516502	-2.70518538500914	3.89812410614664
C	-2.80295699203595	-3.36636159174985	4.80359918223089
C	-4.72838238844081	-3.40488043680815	3.35787938614167
C	-3.04256501224815	-4.69531421290631	5.16252343230698
H	-1.95801718440731	-2.82815802116511	5.24130200631238
C	-4.97220372047481	-4.72892109700040	3.71539646296168
H	-5.39212081882127	-2.89959626569087	2.65096655218771
C	-4.12817601824357	-5.37952009842563	4.61925532320139
H	-2.38087327442911	-5.19354062938278	5.87270106186293
H	-5.82711071148676	-5.25742660212180	3.28911408229240
H	-4.32026975297545	-6.41621330859942	4.90013420809574
H	-4.26905287786394	-0.67293541845314	3.54358378721042
C	-2.55393835472889	0.31824363295902	1.72698703627790
O	-3.44017559229794	0.97719052203831	1.19636921026989
O	-1.41374532421511	0.73350807473116	2.11876415757110
C	1.33230609772091	-1.04603725840647	2.47179588511381
H	2.11688209917635	-1.00847381245808	3.24525628165628
H	0.37922799810601	-0.78767345893646	2.95702207784590
C	1.58582801044715	0.02349944650060	1.41422918391198
H	2.54091655755490	-0.15080833449323	0.88933612347761
H	0.78636671696518	-0.03642164511979	0.67014847960572
C	1.57660153010193	1.40127253797048	2.04787825976847
H	0.68792202219582	1.53443997323432	2.67661153183642
H	2.47368150002395	1.55276805317974	2.66531528918695
N	1.55162559327578	2.50633837955656	1.04485808066813
H	1.91220066526997	3.35282379125985	1.49808836704286
H	2.20452117810784	2.29846339441065	0.27594831494954
C	-1.25561316446068	3.80282335833842	-1.97847566927951
C	-1.50171370365068	2.42958425334835	-1.36042266842753
C	-0.26533351471983	1.92213208476212	-0.61989309792348

C	0.14806656817376	2.93101435460615	0.45872534419789
C	0.40026373897251	4.30907749878314	-0.15949615644212
C	-0.84401226230965	4.80190467526331	-0.89803642722467
H	-0.50709659238320	0.95156500909824	-0.17201338768680
H	-2.33609994045183	2.47046037770396	-0.64197354364157
H	-1.78247772042250	1.68430721941287	-2.11945745339730
H	-0.44077513126020	3.73456470961949	-2.72084949500288
H	-2.14855676555124	4.15416164359241	-2.51853495342498
H	0.67107905779971	5.00498131476954	0.65111280150308
H	1.25132831840114	4.24392052351231	-0.86032466997253
H	-1.66004596106600	4.91349662733611	-0.16792451248008
H	-0.64765411132145	5.79328100241475	-1.33054450882778
H	0.57741664466889	1.80094414769289	-1.32969048673008
O	-0.71354042215026	3.03222919234511	1.49094021921425
H	-1.11069148427138	2.09019991545254	1.73840994213537

93

Intermedia R

C	-1.10212854573425	2.22290838285084	-4.74747170664065
C	-2.42896235406530	1.81603612209472	-4.72753792626863
C	-2.74556657965725	0.49728102184618	-4.39576052403573
C	-1.74410683366805	-0.41611989348038	-4.08456628823611
C	-0.40311395233902	-0.00739014063848	-4.09202975643158
C	-0.10468990610159	1.31293234594523	-4.40445436269951
F	-0.79379649219916	3.46770993526545	-5.06170380371030
F	-3.38614887983039	2.67636584873468	-5.01462179388338
F	-4.02076985983455	0.15387295268014	-4.38838113647968
I	-2.30946499417377	-2.37276485555711	-3.61197620144537
F	1.15581571576102	1.74016953315844	-4.40156359238525
C	0.72532325512269	-1.00020911415871	-3.89225926762457
O	0.93273169787144	-1.86903380108254	-4.70992502926765
N	1.43670047519107	-0.84173427568853	-2.74859906287142
C	2.49135961488841	-1.76199830833943	-2.37217571763936
C	3.89996665231532	-1.17907304150589	-2.54423593944432
C	4.15927849051020	0.09011956210388	-1.76989849022837
C	3.77475626379357	1.33449026115182	-2.29087220518506
C	4.06571421060201	2.51763251847254	-1.60707922550591
C	4.73906600704664	2.47191435187872	-0.38266667827528
C	5.10248870686077	1.23606874397391	0.15787815989877
C	4.81544884004744	0.05655062884127	-0.53179703913517
C	2.28202353847576	-2.34627778510979	-0.95767083382478
O	3.23345945772553	-2.79048679683124	-0.33493133370295
N	0.99853117555240	-2.34017493758008	-0.56202987322927
H	1.20217295146591	-0.07330626472160	-2.12559315429808
H	2.40167451794975	-2.61361926526011	-3.06423584213913
H	4.61150427839234	-1.95495356140908	-2.23579705169433
H	4.04410864883881	-0.98899324717319	-3.61756435501294
H	3.26905472529843	1.37741326072049	-3.25785288134519
H	3.78455232959771	3.47825313370282	-2.04370569528856
H	4.98565765844830	3.39651768293160	0.14338827307114
H	5.62856488119234	1.19047615669634	1.11326338380040
H	5.10107941202448	-0.91135976384947	-0.11450516558572
H	0.32333458429007	-1.89683253914362	-1.18234699578853
C	0.38693188644487	-2.72364541043301	0.69746912156283
C	1.26472711476662	-2.54063995434926	1.95021690577520
C	-0.92821744463488	-1.91196267707316	0.73983183923051
O	-1.28349000788194	-1.25463830711724	-0.23355500307834
C	-2.80354508825651	-1.09067384227113	2.00261147960298
H	0.83630507527302	-3.16908157754355	2.74680122417520
H	0.09683879246983	-3.78859422861194	0.64990403710654
H	-3.53621658915517	-1.33471272155639	1.21874113401852
H	2.24674783447365	-2.96910777008147	1.71702846595434
N	-1.65259682685067	-1.96440915485659	1.86446370476008
H	-1.29899308712219	-2.46467738285014	2.67262684953073
C	-3.43941761873645	-1.26364244850137	3.38431664408259
H	-2.72305272631463	-0.91877982123485	4.14692435909107
C	-3.85682048550866	-2.68551649931452	3.67283985037517
C	-3.21611202728885	-3.43817578321378	4.66269357302150
C	-4.88088061701068	-3.28949201242210	2.92987191246726
C	-3.58540556088576	-4.76325473747977	4.90743732834752
H	-2.42202410747717	-2.97629156422580	5.25538415397911
C	-5.25325681250355	-4.60978379594201	3.17189262142412
H	-5.39290540097722	-2.71288273467110	2.15518539971994
C	-4.60494490538943	-5.35233458070763	4.16238570817292
H	-3.07574046642170	-5.33399809618220	5.68572226521041
H	-6.05565055082341	-5.06306465685803	2.58709679599485

H	-4.89738696571338	-6.38630153285891	4.35239812530424
H	-4.30784945230885	-0.58979435015881	3.42132217077967
C	-2.43453158248692	0.40082528830766	1.79446825196341
O	-3.31482796878769	1.14062397789265	1.36874772227307
O	-1.25117220460082	0.73067199057376	2.13271620431217
C	1.40787951071647	-1.10910517531315	2.47444777277511
H	2.20014589264141	-1.09490274490587	3.24055177761191
H	0.47261006756772	-0.79489824138758	2.96087570440021
C	1.70514686681585	-0.07303285540810	1.39475394463724
H	2.64415299365938	-0.30893180897594	0.86695686656268
H	0.89495535107860	-0.10363419959230	0.65915234587738
C	1.77753554135935	1.31745403992801	1.99580471247400
H	0.92361766322876	1.50269864345620	2.65871517613477
H	2.70542391951366	1.44762121162162	2.57063650977889
N	1.75088148502771	2.39906091234584	0.96696846710686
H	2.18519159391930	3.23444390705015	1.37367494500920
H	2.34242886032543	2.14521901980521	0.16294925003713
C	-1.16882250684195	3.80797638587826	-1.89876629827343
C	-1.42729956658031	2.44197709873852	-1.26952010115749
C	-0.16458559089641	1.89476796459227	-0.60604262455549
C	0.33550722791577	2.88150668883356	0.45635319477700
C	0.60262055341305	4.25234325267167	-0.17076508195344
C	-0.66199714272888	4.78927145390859	-0.84204975219694
H	-0.39880701927204	0.92095533602525	-0.16091151082166
H	-2.21531970663599	2.50978362832137	-0.50228835258972
H	-1.77860686939975	1.70922479069045	-2.01160644826451
H	-0.40016033985343	3.71301623247841	-2.68621246657783
H	-2.07783465063587	4.19197332269975	-2.38722822142190
H	0.93787215196584	4.93543699586537	0.62656505584502
H	1.41385147744808	4.16078631398533	-0.91421310572180
H	-1.43270073883144	4.92886485715503	-0.06875516018583
H	-0.45441641404788	5.77387020003162	-1.28494366089155
H	0.63336058512542	1.77308621946440	-1.36583293271242
O	-0.46910833361160	3.00944346619378	1.53041849373558
H	-0.89217376033676	2.08282761606312	1.78949376793585

93

TS16

C	-1.21299968918975	1.96809249567424	-4.71836039854512
C	-2.54447356399843	1.67515383712414	-4.46256677272743
C	-2.90771371663304	0.39137207969284	-4.05492811342794
C	-1.94862683287786	-0.60292498312509	-3.88724533635359
C	-0.59564195633591	-0.30489315199590	-4.11773099184136
C	-0.25266634993332	0.97994502186698	-4.52839263428906
F	-0.85896273755570	3.18319783714411	-5.09232953200557
F	-3.46007486777221	2.61509103375319	-4.59239382480178
F	-4.18887151836361	0.16407837313134	-3.82624144183165
I	-2.62948206382226	-2.47557259364650	-3.24464607204641
F	1.01973888231340	1.31425884583504	-4.73150519890058
C	0.49005149506253	-1.33437994872980	-3.89002445632844
O	0.44396079800676	-2.43827413001431	-4.38464721371950
N	1.47074937060700	-0.91577474163378	-3.04748797938968
C	2.49386170831883	-1.80807747549589	-2.55408831088979
C	3.89969040809456	-1.19992718719203	-2.66020308796350
C	4.00803032218353	0.15097492657154	-1.99712782475059
C	3.73153863240244	1.32271210668021	-2.71849285479096
C	3.82051913660674	2.57403629557554	-2.10974138239655
C	4.19760153004388	2.67247142620630	-0.76836056521039
C	4.46543874531416	1.51390346609629	-0.03945575847258
C	4.36426288806438	0.26132001001525	-0.64817794465127
C	2.21396185447282	-2.32246548860574	-1.12588940980759
O	3.00626887824219	-3.08074657857804	-0.59168782333000
N	1.06236130986004	-1.89282771443759	-0.57709432179191
H	1.45001449853482	0.03969884913529	-2.69951903959856
H	2.45444993506748	-2.70878364403934	-3.18357784383470
H	4.59370176855880	-1.91339244605777	-2.19665961000522
H	4.15426876456005	-1.11379406872633	-3.72552596697334
H	3.45635958934106	1.24970416756851	-3.77344636321557
H	3.60876185387692	3.47578047400141	-2.68761177773302
H	4.29258959470652	3.65385229522705	-0.29808055016870
H	4.76752901353567	1.58329290270031	1.00813609924896
H	4.57465935811980	-0.64744488185458	-0.07781960425339
H	0.43816597627048	-1.31667751738855	-1.13767828072833
C	0.42365961668259	-2.43340175493474	0.60746016641891
C	1.26510892615682	-2.37749046548855	1.89962132042963
C	-0.93445844404112	-1.70472716936410	0.67882268160718

O	-1.32685941570622	-1.02639646627753	-0.26235049985347
C	-2.87994325614938	-1.04530261859948	1.94555854440519
H	0.84476088827806	-3.12887377983387	2.58796052410516
H	0.19225779469905	-3.49960815274429	0.42519370082338
H	-3.61789953141744	-1.35520247087864	1.19008344765393
H	2.26702789001367	-2.74275672523170	1.63509068599885
N	-1.66638117010227	-1.82820501155041	1.79931073189342
H	-1.32597045532805	-2.37837512862102	2.58054259220776
C	-3.46121403264462	-1.22847933732758	3.35308850943027
H	-2.74977019318072	-0.81481636323147	4.08439501818835
C	-3.75900030872699	-2.67314584091115	3.67348857633696
C	-2.98175146793056	-3.38051447236736	4.59675369583153
C	-4.80272133188062	-3.34244355670840	3.02007839086026
C	-3.23770535896551	-4.72784540832933	4.86300255351936
H	-2.17209550968871	-2.86605678757309	5.12151993822455
C	-5.06187503132789	-4.68524883167420	3.28471267237735
H	-5.42068732623189	-2.80028249290274	2.29958391803057
C	-4.27794841882838	-5.38311043557766	4.20697890251978
H	-2.62349568996329	-5.26386261056750	5.58853027112737
H	-5.88147115095475	-5.19086102690672	2.77121850573146
H	-4.48187515206984	-6.43468194783693	4.41464448740782
H	-4.37988687533720	-0.62595350810081	3.40657989058386
C	-2.63777114022817	0.45355406862801	1.69201535367342
O	-3.45701279171021	1.12645828327776	1.11300727373259
O	-1.52273505986300	0.89422444329052	2.21852647159327
C	1.33162260445408	-1.03990359705290	2.63437217574595
H	1.81683445041670	-1.20704109304828	3.60977449271097
H	0.30585915269188	-0.71095202072630	2.85802987692806
C	2.04469488278449	0.11088672711177	1.91158958216505
H	3.13501888214169	-0.02609359458351	1.96099592765011
H	1.78735435289200	0.10837001633795	0.84726660570696
C	1.62038644426914	1.42668373907531	2.57210722382479
H	0.60136256753754	1.30037710097315	2.95950896219100
H	2.25855750770258	1.64484127686619	3.44073556246323
N	1.56747210120123	2.67484563847585	1.78172761531547
H	0.61084514594144	3.25979318911171	2.30035312835861
H	2.48199541898057	3.06798603657284	1.55100648092962
C	-0.65095196078409	3.35131167864410	-1.94155108722626
C	-0.92561143156627	2.06067309587648	-1.17090993704931
C	0.23563110841209	1.70528447780842	-0.23869765164303
C	0.49358240421188	2.85857742944673	0.71838684475365
C	0.83422481131609	4.13014333608897	-0.06124851827133
C	-0.32950497931419	4.49643934757193	-0.98084059440684
H	-0.01646840414398	0.79949143638910	0.31795878066806
H	-1.83675565478400	2.17675103686717	-0.56166675656680
H	-1.11370741737278	1.21324995111482	-1.84820292758889
H	0.20292015619718	3.19812632341904	-2.62867902889149
H	-1.51618993840123	3.61898811445068	-2.56780221058086
H	1.03213451997534	4.94163870968534	0.65682185783342
H	1.74723375792249	3.95859233328780	-0.65487896489626
H	-1.21051630613842	4.70017392356527	-0.35267058568028
H	-0.09581798172518	5.41735437382417	-1.53440033965845
H	1.16123648255101	1.51787806333527	-0.81704677978830
O	-0.54200909859717	3.10381108461525	1.62970687161593
H	-1.24274259800816	1.89214754076026	1.95343725605375

93

Intermedia S

C	-0.84427778507187	2.21831620255700	-4.84532567517421
C	-2.21566156887080	2.05670332295423	-4.71931108777660
C	-2.73627814039211	0.81957812775701	-4.33887881933656
C	-1.89661016896314	-0.25721377929365	-4.06987275359816
C	-0.50516081885002	-0.09567439822085	-4.17431257606842
C	-0.00567669730936	1.14479514011390	-4.56160059281905
F	-0.34089944322342	3.38819670787554	-5.19076533119945
F	-3.02071788081330	3.07746937290759	-4.94232808023669
F	-4.04939066413627	0.71928386406418	-4.23160291975582
I	-2.80924575174930	-2.03937450257147	-3.45874896366730
F	1.30491045676791	1.35293511740294	-4.66009467161487
C	0.45497753771261	-1.23119229785044	-3.88254056773617
O	0.33091300043399	-2.31833534406596	-4.40093811525721
N	1.42170328264437	-0.92100762254549	-2.98035843826738
C	2.39634417488370	-1.88751667697984	-2.52387811228644
C	3.83626994921454	-1.36380445579089	-2.64280099979248
C	4.03305435312402	-0.02746063580669	-1.97028672656902
C	3.81725575534692	1.16239872023816	-2.68157166743196

C	3.96241460903225	2.40352878274265	-2.06183275788773
C	4.34527754728433	2.47250139741160	-0.72067674604730
C	4.56685145833713	1.29514814649025	-0.00484191277220
C	4.40203473161786	0.05405606596720	-0.62234510378703
C	2.11356788959381	-2.42173873709565	-1.10404968645751
O	2.88479436587330	-3.21392115652715	-0.58791353804609
N	0.97686553854089	-1.97555689158583	-0.54270420091344
H	1.47938564189099	0.03025260550500	-2.62515352553736
H	2.29076130479028	-2.76768819963772	-3.17486767753257
H	4.49020630903244	-2.12325523287665	-2.19431977504059
H	4.08245073426441	-1.28299619023263	-3.71057111664696
H	3.54167457929536	1.11099715499622	-3.73710710234107
H	3.79133020680003	3.31874524937522	-2.63192442582569
H	4.47874733590298	3.44303747936709	-0.23777253956008
H	4.87087302699115	1.34256381797281	1.04315234697718
H	4.56872305011798	-0.86823045666627	-0.05933215893920
H	0.37571062886162	-1.35504573656382	-1.07989157647740
C	0.34918979235054	-2.50285486593198	0.65139634230037
C	1.17738151237673	-2.39444713097216	1.95176212442224
C	-1.01338280419613	-1.79266977538017	0.71669702104025
O	-1.39026959673528	-1.06397616383039	-0.19222282814007
C	-3.01533164430831	-1.26533584008260	1.96767256182835
H	0.78839650122727	-3.15491034844529	2.64874675428952
H	0.14069784285776	-3.57704196936559	0.49343065575855
H	-3.77001170797424	-1.64183518294556	1.25945952965909
H	2.19645522118984	-2.71790707894599	1.69547318195351
N	-1.76820097607033	-1.98700615717127	1.81306796324060
H	-1.44781324740937	-2.59490049126297	2.55985431893707
C	-3.52891457269661	-1.39789602232384	3.41048262028465
H	-2.83277115637106	-0.86767902803940	4.07777553234712
C	-3.66714825324621	-2.83569178730392	3.84848335517319
C	-2.76227016930034	-3.39440690760713	4.75823534781440
C	-4.67990700379584	-3.64738857303769	3.32053479445565
C	-2.86440980195971	-4.73589713731780	5.13441868583116
H	-1.97383817296285	-2.76774078284926	5.18421723253237
C	-4.78518480937667	-4.98510594580390	3.69538321349479
H	-5.39510408859375	-3.22217825582392	2.61162701370693
C	-3.87617468715335	-5.53376652998812	4.60353523362247
H	-2.15298130070223	-5.15484811018767	5.84803071329992
H	-5.58212927698240	-5.60378802035061	3.27945077324026
H	-3.96016886920758	-6.58109232702303	4.89767602661210
H	-4.49952386514850	-0.88331588547881	3.46214629032218
C	-2.85038274350804	0.22245470695502	1.64139625808026
O	-3.67679689152103	0.85725211519007	1.04322431619833
O	-1.74353342709354	0.73042640996830	2.16612460088773
C	1.18905945423053	-1.03716094893040	2.65492241879478
H	1.74498910985725	-1.14229301338358	3.60110069953360
H	0.15923003047230	-0.76320666163078	2.93294380383627
C	1.78152604951636	0.10640469239489	1.83416371454919
H	2.87229357157999	-0.02059297790444	1.75015328771476
H	1.39840798475768	0.05922857698828	0.80861019805121
C	1.44545476922740	1.47211232374028	2.44641882722362
H	0.41205087662097	1.44391194051895	2.81906543205338
H	2.07726693399521	1.63065833362810	3.33680919073011
N	1.56066263406068	2.64708676263962	1.59359463698223
H	-0.49611920105281	3.77634875869432	2.17077954243882
H	2.44990158458910	2.67423784160363	1.09990126834382
C	-0.58917963352664	3.62484673873196	-2.02402021400242
C	-0.98838054615806	2.33581667438021	-1.30982679093505
C	0.10417571634650	1.87885910148141	-0.34454720722124
C	0.47001236366736	2.95856355067089	0.68523172166592
C	0.82596563691494	4.26361924252134	-0.03828581033302
C	-0.26596265404731	4.71715761488547	-1.00506198537597
H	-0.22483754188995	0.96932503055724	0.17201198783438
H	-1.92101104179016	2.49579363606107	-0.74452010033535
H	-1.19750210724006	1.52652403829621	-2.02606622985591
H	0.30461139006067	3.43580989463107	-2.64780895254680
H	-1.38626245414093	3.95925563512356	-2.70707893432146
H	1.03236764186943	5.03146526228046	0.72403662867016
H	1.76672873724267	4.09389923211655	-0.59038264769452
H	-1.17630872590970	4.95193294096096	-0.43050329354127
H	0.04564823128058	5.64265908216160	-1.51109814941943
H	1.03156615917562	1.63715247151663	-0.89858974766171
O	-0.71212005739205	3.14814796657885	1.46549377681433
H	-1.59379166931316	1.67037306844356	1.88931239342979

93

TS17

C	-1.43919882776416	1.80060244720103	-4.51307385163544
C	-2.74299215053159	1.53700035252295	-4.12028468017564
C	-3.07703802050685	0.27593697883448	-3.62596063674407
C	-2.11643536237924	-0.72505330967648	-3.50798039955862
C	-0.78736416076136	-0.45585683175489	-3.87828099864532
C	-0.47554196661449	0.80704356016377	-4.37670371137490
F	-1.10975493027782	2.99558341178588	-4.96801753768641
F	-3.65703449388040	2.48424702745236	-4.20241012883178
F	-4.33354605849304	0.07654377353131	-3.26955888841106
I	-2.76730762229462	-2.55894612641395	-2.73282399166791
F	0.77282104348603	1.11994341170965	-4.71793246961101
C	0.30617778289417	-1.49049885885521	-3.71110882692095
O	0.17585678391216	-2.63200906227789	-4.09237030153086
N	1.40273322352323	-1.03015095376050	-3.05429962387760
C	2.45545950881993	-1.91072680128767	-2.60302210369908
C	3.84637516610040	-1.29409592769042	-2.81308899090270
C	3.96079901847586	0.09047295613104	-2.22443400996074
C	3.66241185512288	1.22123951892232	-2.99859710973254
C	3.73795647213595	2.50284025189447	-2.45068931595471
C	4.12134804576489	2.67002360989108	-1.11892994258801
C	4.41035567279795	1.55125951624823	-0.33725456318873
C	4.32595535083246	0.27092886462185	-0.88445830223399
C	2.26405002024416	-2.38500167799765	-1.14485358304562
O	3.05497386104524	-3.17413882210579	-0.65597769135991
N	1.18435763599865	-1.88275325558361	-0.51921105856635
H	1.44570789055261	-0.05110384193558	-2.78395019168095
H	2.38291818989812	-2.83066548459036	-3.20055134670769
H	4.57309861470302	-1.97466022436612	-2.34943518621085
H	4.04754668581597	-1.25915543248163	-3.89258407984695
H	3.37707583477509	1.09349935030615	-4.04574821461947
H	3.51093018638505	3.37233275561620	-3.07077166699193
H	4.20230029518961	3.67147907109571	-0.69151104288891
H	4.70622221632890	1.67733299575930	0.70604264617117
H	4.55273406469000	-0.60639225004757	-0.27224366046860
H	0.55586219748929	-1.27375820795722	-1.03833812893353
C	0.58869700030102	-2.38704824154872	0.70242604515722
C	1.47592356188186	-2.29131180442468	1.96234471744741
C	-0.76174048169401	-1.64474867608312	0.80065239782827
O	-1.14228365889594	-0.93198523055455	-0.12259781345846
C	-2.66927895355707	-0.95607857762701	2.09777754194349
H	1.08905623573765	-3.02880667308944	2.68413378438168
H	0.35184611283032	-3.45740037184291	0.55871361508764
H	-3.38057549572251	-1.14152363790341	1.27955446717118
H	2.47149344283529	-2.65537928409690	1.66949736587790
N	-1.49728405620851	-1.79789338632478	1.91125768549802
H	-1.13897529685661	-2.34180739355380	2.68881167748389
C	-3.32460025519803	-1.25800647723015	3.44784697752387
H	-2.62385730492013	-0.97428819338389	4.24860760563152
C	-3.72964170568061	-2.70478637169060	3.59637372383341
C	-3.08352258753367	-3.54561381292349	4.50853145232181
C	-4.74674922037248	-3.24114757785645	2.79449958835155
C	-3.44109239383645	-4.89167591347532	4.61973870550448
H	-2.29488273027891	-3.13764763149686	5.14637556517081
C	-5.10700290423024	-4.58244148566882	2.90309819349500
H	-5.26312139384792	-2.59547991792255	2.07941859543014
C	-4.45336181253638	-5.41346117682223	3.81665727337212
H	-2.92721681198899	-5.53198123162401	5.33882041618362
H	-5.90411258901891	-4.98268055893912	2.27405456191714
H	-4.73592414200490	-6.46394050355083	3.90213999533205
H	-4.20421728788558	-0.60259540384993	3.53307683589167
C	-2.30445806154791	0.54864652773595	2.03301092513519
O	-3.09646912795261	1.32247756051656	1.49514256551088
O	-1.20756221181053	0.86734978298589	2.59384803997639
C	1.55253484640898	-0.93299101729981	2.65875452092666
H	2.06612647741274	-1.06848850827464	3.62462024552469
H	0.53221430483506	-0.59573333011914	2.89384561383593
C	2.24561761259686	0.18568606535790	1.87264096239431
H	3.33676626245621	0.04298789954608	1.88226244757823
H	1.95177239599181	0.13615746195132	0.81941532995138
C	1.86857904748481	1.54832160389020	2.47359085345490
H	0.89077398798701	1.45426177102084	2.96679162795397
H	2.59316982309079	1.83390879852525	3.24685545336610
N	1.78712218249321	2.69564607056875	1.55856334520437
H	-1.62561068603924	3.28496919231955	1.31038230746351

H	2.21710863175118	3.54533897203904	1.91244728205451
C	-0.66080924346546	3.34547035809464	-1.84119920113223
C	-0.87404111181950	2.06467603741159	-1.03967806877615
C	0.32891190037970	1.72949056420306	-0.15959507214569
C	0.86798931390572	2.89020389387465	0.62662021802575
C	0.90405940252939	4.22545938722813	-0.06912265698957
C	-0.33195669886363	4.51754995835886	-0.91725096579702
H	0.08045527782972	0.90324532120541	0.51168706115400
H	-1.77197294824720	2.14436191682625	-0.40787092483533
H	-1.05680889553309	1.20209899121479	-1.69635014087599
H	0.16815880422205	3.20145171247218	-2.55953965566866
H	-1.55625661957229	3.58025990327390	-2.43645436106606
H	1.08448575469395	5.01659357648536	0.67291213644007
H	1.78799859855340	4.17812978392149	-0.72939658352101
H	-1.18970734604325	4.72300318887155	-0.25895595724243
H	-0.15367954331884	5.43272424516953	-1.49908494801293
H	1.18287424261295	1.40794136546452	-0.79206034388368
O	-0.77244799101456	3.19012011947151	1.76399056544405
H	-0.92232767680363	2.25506757426719	2.20777999425608

93

Intermedia T

C	-1.47946241703268	1.79381852991480	-4.43288393632550
C	-2.78525084557966	1.51571474230540	-4.05883556601181
C	-3.10686014656247	0.25573323263673	-3.55274479692593
C	-2.13507945594892	-0.73129975706987	-3.41247718908668
C	-0.80375506463774	-0.44938505732114	-3.76897148713968
C	-0.50334640465502	0.81581179183034	-4.26955246936064
F	-1.16156070103540	2.98443171425484	-4.90760217834221
F	-3.71387203930348	2.44468760526832	-4.17401066786259
F	-4.36522991274957	0.04325496630664	-3.21141370189434
I	-2.77975158983645	-2.56547295619130	-2.63162805540996
F	0.74445335968783	1.14535765667599	-4.59984076986780
C	0.28989894548676	-1.48841630490316	-3.62515115515146
O	0.13347109473672	-2.63291720589213	-3.98757855519396
N	1.41931639335142	-1.03795130461514	-3.01798074245628
C	2.45840036220368	-1.93985151462952	-2.57495497601246
C	3.86084594240234	-1.35881058811374	-2.80426958524008
C	4.00941348821856	0.02982529591942	-2.23388886571760
C	3.75339804477617	1.15676271508043	-3.02839233906003
C	3.86052246944888	2.44351276903040	-2.49945671726072
C	4.23226632052146	2.62030797775260	-1.16512514227149
C	4.47917526668242	1.50557010445308	-0.36321559028939
C	4.36288059839276	0.21948324935021	-0.89199254501598
C	2.27472828653209	-2.40211910082709	-1.11153783839620
O	3.06157248805849	-3.19519854052071	-0.62224776746354
N	1.20558095777968	-1.88372487034115	-0.48262842799009
H	1.48986824756075	-0.05704858138936	-2.76261259655466
H	2.35676884303434	-2.86129289042420	-3.16584603764778
H	4.57482938381356	-2.04978198883362	-2.33634604339755
H	4.05638131489929	-1.34212002217954	-3.88511604670238
H	3.47657175578195	1.02113574231334	-4.07680558563377
H	3.66984335831297	3.30903223528412	-3.13721497272532
H	4.34002108366577	3.62633806042284	-0.75377836961720
H	4.77456182065320	1.63862632348952	0.67957718856483
H	4.55838811479285	-0.65465074930659	-0.26449054800639
H	0.57943071803144	-1.27079789920737	-0.99968151103521
C	0.61247539084582	-2.36434940649789	0.74897393020555
C	1.50508631917047	-2.24792680412701	2.00394574311617
C	-0.73441969654814	-1.61251852838140	0.83906940161714
O	-1.09049097730669	-0.87607246762545	-0.07632892887809
C	-2.67557194236509	-0.97265722517497	2.11947885249266
H	1.13002666637868	-2.98014883353089	2.73678967846266
H	0.37228533714474	-3.43614063182559	0.62551112235495
H	-3.37244483599718	-1.14361027420254	1.28578212806708
H	2.50312737455740	-2.60434749304696	1.70958539900436
N	-1.49024051957349	-1.79490346336926	1.92899869272621
H	-1.15315903658175	-2.37602489577455	2.68904152331515
C	-3.34938927546743	-1.32645498386344	3.44749426264663
H	-2.66819861087342	-1.05341871507638	4.26878194855030
C	-3.73145462405120	-2.78332613847264	3.54867626745691
C	-3.08356725136441	-3.63983912720741	4.44505653300119
C	-4.72657543344802	-3.31430705181044	2.71598245832380
C	-3.41760655095023	-4.99491873021860	4.51107728287961
H	-2.31236891530840	-3.23655628452073	5.10699346089676
C	-5.06351904346992	-4.66446833582820	2.77941663335496

H	-5.24388702418530	-2.65671690553857	2.01266410511687
C	-4.40809531724812	-5.51078702980765	3.67770020160034
H	-2.90253816966549	-5.64701581564053	5.21883151082755
H	-5.84389829617004	-5.05989060602808	2.12689109632613
H	-4.67254084321322	-6.56826558415446	3.72764549966933
H	-4.24107638723119	-0.68787979687479	3.53351018351956
C	-2.33506370651248	0.54387200535677	2.11546374605602
O	-3.16982145379725	1.31019789206581	1.62475105806882
O	-1.24335623826286	0.85891257675738	2.66908399772940
C	1.56922107015284	-0.88148168983213	2.68575176176023
H	2.12452900417730	-0.99035669238391	3.63114072409252
H	0.54766997277444	-0.57162835987927	2.95408424738496
C	2.19889334318892	0.24161665541613	1.85222930201420
H	3.29560965853537	0.15736708643810	1.84806568282421
H	1.88307912365118	0.14078593992906	0.80735890973550
C	1.74876557186845	1.59482216160188	2.41066267753025
H	0.70784734739244	1.50127038499817	2.74820275164147
H	2.36027793225315	1.88765738717776	3.27273708423187
N	1.77951678003429	2.73807451635950	1.48177293824117
H	-2.01836985388877	3.43915796588012	1.62698262928391
H	2.33137782516887	3.54123869814430	1.77844185418314
C	-0.71323454544362	3.42839136900096	-1.83120894805503
C	-0.98033271018507	2.20717565303169	-0.95906687681202
C	0.24727217855073	1.78935233294259	-0.14856401112155
C	1.03633494076184	2.91001694160042	0.43662529524897
C	1.11813443639931	4.20700069454071	-0.30355851893969
C	-0.20238794452596	4.58695416328020	-0.97763495105332
H	-0.04093006237198	1.07858033590368	0.63292531781600
H	-1.79653138166729	2.41070672675358	-0.25310540034787
H	-1.29468212056239	1.33834201956676	-1.55481563113880
H	0.04027268278988	3.18775447391661	-2.60459881263050
H	-1.62972401799339	3.72209852487372	-2.36519627108686
H	1.49091847795146	4.99768508028858	0.36334695833640
H	1.88033633540986	4.04547065605820	-1.08717961725349
H	-0.94053158906416	4.82864085359307	-0.20014107973300
H	-0.03986700026282	5.48690223068144	-1.58632063970510
H	0.97669453718378	1.27694103715674	-0.81207398022712
O	-1.09509698322225	3.42792238591671	1.91884808868366
H	-1.07589940601646	2.51434120390798	2.30668107374784

93

TS18

C	-1.56167148540839	1.67429726057709	-4.49014683835968
C	-2.84759226203259	1.41377669003928	-4.04143543520111
C	-3.14850471291926	0.16826954197774	-3.48926935643644
C	-2.17454963007047	-0.81951003556557	-3.37046406817465
C	-0.86056614622950	-0.55103927356976	-3.79526104701958
C	-0.58241773814887	0.69626898109826	-4.35188607398296
F	-1.26474971778106	2.85089382296137	-5.01228405059470
F	-3.77901981543149	2.34169584731517	-4.13889151028147
F	-4.39199381644591	-0.03118660026894	-3.08886079804295
I	-2.79751662089862	-2.63800064557511	-2.53510764486856
F	0.64731643484526	1.01093898878240	-4.75457475901101
C	0.25113158130665	-1.56650225188114	-3.62835312444505
O	0.10601407901959	-2.73510351679028	-3.91039335051080
N	1.38920244133648	-1.05777828863494	-3.08783997374070
C	2.46565616405773	-1.90911431112096	-2.63609669041785
C	3.83814985218411	-1.25574958417746	-2.86112814653338
C	3.90811171175273	0.14353841374432	-2.30099252065420
C	3.56760182705278	1.24768850979800	-3.09545906341475
C	3.57500705721453	2.53980056328840	-2.56561242258077
C	3.93164451195277	2.74497052388673	-1.23268261171980
C	4.27703243899576	1.65299549742440	-0.43466706916456
C	4.26000452306982	0.36248680364076	-0.96271983440209
C	2.28933427976132	-2.37818274518393	-1.17315781208190
O	3.07104604182228	-3.18182837347205	-0.69467534369436
N	1.22890063994914	-1.85116144764881	-0.53351632178157
H	1.44176203457442	-0.06417082556710	-2.87874777453045
H	2.41595722329044	-2.83805715831041	-3.22182344105609
H	4.58572249194865	-1.90497727640411	-2.38547698047438
H	4.03696240026874	-1.23698541754617	-3.94148590269164
H	3.29581055143913	1.09149716836999	-4.14251285435666
H	3.30861385466435	3.38755803093321	-3.19980560688971
H	3.94103676445954	3.75416112171522	-0.81610791213796
H	4.55306346490680	1.80746005369262	0.60989185891473
H	4.52476178861679	-0.49287155998630	-0.33497811025843

H	0.61322902822040	-1.22907106415166	-1.05159731193842
C	0.60243779905638	-2.36325784717599	0.66896448754015
C	1.47190079585902	-2.33396182683252	1.94341894277995
C	-0.72692113469523	-1.58228774454040	0.75992476026831
O	-1.09020349222485	-0.87160799289705	-0.17182802721470
C	-2.65467662581252	-0.88436275247096	2.02143573995117
H	1.04518727832345	-3.07320041422850	2.64058076245888
H	0.32863542566749	-3.42051665616768	0.49441512977801
H	-3.35051735856343	-1.10031899944559	1.19695221400245
H	2.45750794711962	-2.72835587206835	1.65630839201792
N	-1.46738950102310	-1.70715016520757	1.86999740124008
H	-1.13559066039960	-2.26776802143457	2.64738141814944
C	-3.33010122124165	-1.17284739500900	3.36543747211843
H	-2.65080343666630	-0.86314639425102	4.17508076360344
C	-3.71180517721354	-2.62421376179569	3.53017558443537
C	-3.06651177724502	-3.43907446089667	4.46604030772143
C	-4.70485354867608	-3.19082376639090	2.71892821662392
C	-3.40189884023169	-4.78964670294180	4.59164007616026
H	-2.29671642228715	-3.00730650002186	5.11120985561596
C	-5.04297501666460	-4.53661144415004	2.84206982349485
H	-5.22013766530625	-2.56548504902749	1.98517708131724
C	-4.39053183355802	-5.34160825635001	3.77951937977820
H	-2.88945930233034	-5.40967233196316	5.32923910832579
H	-5.82190649732801	-4.96063929121167	2.20587917855071
H	-4.65587542624143	-6.39556242117474	3.87648793428203
H	-4.22249820261194	-0.53201946239861	3.42010018943833
C	-2.34084931847809	0.62831453137195	1.93053685748549
O	-3.19461018548052	1.36203304135979	1.43138021967521
O	-1.23560688318040	1.00842368573015	2.43986127436261
C	1.59088623078341	-0.99603942427299	2.67196496220341
H	2.07231458245511	-1.17149545037882	3.64756337978560
H	0.57819296674565	-0.62326334790887	2.89046020186790
C	2.34251468606085	0.11002743191970	1.92236093942140
H	3.43092978108182	-0.04818721549248	1.97986929283203
H	2.09075238262910	0.06490424314696	0.85861681333114
C	1.94998661388129	1.47188567018021	2.50963456348009
H	0.93201359694620	1.39009620622484	2.91773070406386
H	2.61451291845889	1.72964500314568	3.34447812509707
N	1.96774118539539	2.63428612637773	1.60933399108241
H	-2.18629841653626	3.40421651319209	1.53483453498253
H	2.36933160820496	3.47556090445954	2.01291060344349
C	-0.21682624593561	3.25330558683758	-1.93711617729615
C	-0.62771378001810	2.08997241285402	-1.04185700630887
C	0.53290549243869	1.68327780789862	-0.13905307593988
C	1.14996890735802	2.84611515912415	0.57979098241128
C	0.69377513044307	4.14281428645161	0.27183581049285
C	0.12892559352642	4.49159127799546	-1.09828945264041
H	0.19189483374401	0.94887876588761	0.60066185609410
H	-1.49216503001760	2.36192587930874	-0.41497408727769
H	-0.93915679365495	1.20883916080191	-1.61999988693919
H	0.65958564280434	2.94205031419327	-2.53382785129950
H	-1.01101946754237	3.50741402966480	-2.65294861636730
H	-0.34883973114266	3.81742457922967	1.07793935528357
H	1.18334488489311	4.96144393573982	0.81448685460453
H	-0.77532124994749	5.10522834715759	-0.95779499783463
H	0.84468332151538	5.12834781150910	-1.64174029273327
H	1.34292891828351	1.21278010370913	-0.72983895530652
O	-1.29780963927666	3.44539762040548	1.92948989641770
H	-1.18975988348761	2.42722308883741	2.21528289162119

93

Intermedia U

C	-1.34897288916695	1.84803613312821	-4.53854300931069
C	-2.65470465176012	1.72038681470580	-4.09049327221372
C	-3.06823217196151	0.52996404802330	-3.49256895135644
C	-2.18599584108216	-0.53375056086710	-3.32537179096508
C	-0.85218606739033	-0.40165565250308	-3.75161329566275
C	-0.46170509728412	0.79208558221738	-4.35614826892669
F	-0.94198048082978	2.97660694077472	-5.08871864500036
F	-3.49656858653000	2.72729634771834	-4.21762495996150
F	-4.32470790337626	0.46282621821869	-3.08706067979732
I	-2.96571451724578	-2.25210919408797	-2.41292803252594
F	0.78968806610520	0.97690188394729	-4.76801223092347
C	0.16294823599111	-1.50858871868508	-3.54935925971542
O	-0.08663889247066	-2.66395237958279	-3.81443884713185
N	1.33715187606643	-1.09256726478845	-3.01112664489984

C	2.34573076920859	-2.02477756564264	-2.55972594090836
C	3.76432667990257	-1.49309836210405	-2.82151047584351
C	3.94539075139884	-0.08241997670910	-2.31927479127554
C	3.67980919515727	1.00540597531743	-3.16316984901879
C	3.76162467126888	2.31589538246793	-2.68838965097071
C	4.12174504708806	2.55442311818343	-1.36250307221705
C	4.39690554258006	1.47912278016750	-0.51690146250437
C	4.30280700513571	0.17030262017536	-0.98889414427404
C	2.15555171748540	-2.44419802029412	-1.08641593186171
O	2.86425860926864	-3.30890483036575	-0.60040052775788
N	1.15634726772343	-1.82218112260061	-0.43376305044923
H	1.47271983168824	-0.10501208406356	-2.80701893494807
H	2.20494602065821	-2.95894001903979	-3.12165455540972
H	4.46211323225297	-2.18262546543626	-2.32702262745107
H	3.94859009882890	-1.52860851236214	-3.90399369811472
H	3.40197471678719	0.82018287616203	-4.20378962548896
H	3.54605969864280	3.15040155316396	-3.35845361024386
H	4.17834536331131	3.57585981250754	-0.98284700620940
H	4.66761082663761	1.66738661254252	0.52201154510323
H	4.50595434688079	-0.67178575197331	-0.32101031165874
H	0.59787529236422	-1.12817178674531	-0.92321999057566
C	0.55577804896834	-2.29144776312404	0.79576630985843
C	1.42930901444061	-2.16818650194459	2.06457579004619
C	-0.77712903389940	-1.53261807667910	0.88765187660743
O	-1.09690108896491	-0.70890058703580	0.03920989693388
C	-2.82394341318343	-1.06393099549686	2.06646919183848
H	1.03649426802437	-2.89076918765906	2.79800640719966
H	0.31524254775362	-3.36323967874396	0.67509765555326
H	-3.49291887624880	-1.31121581901552	1.22704742235239
H	2.42853873453541	-2.53396529174546	1.78435291864114
N	-1.57727539560849	-1.79027919774331	1.93760429008421
H	-1.32164706011679	-2.48455560160085	2.63200087283582
C	-3.50493888368270	-1.39929251085128	3.40093119010135
H	-2.87548077049551	-1.02326364797280	4.22163701320995
C	-3.74226559183782	-2.88110749556588	3.56449089458621
C	-2.98828214652224	-3.63067525187997	4.47371035685358
C	-4.69544755878768	-3.53896749661359	2.77544323152991
C	-3.17828944702076	-5.00953217814737	4.59335259882927
H	-2.24926310681167	-3.12635942152481	5.10214483661070
C	-4.88854248599651	-4.91360856201891	2.89412165670723
H	-5.29419214704853	-2.96418024042725	2.06400486079776
C	-4.12823085884205	-5.65366127638615	3.80323861581410
H	-2.58325998951436	-5.57902117366617	5.30927327667474
H	-5.63777437412892	-5.41152874698068	2.27634758990111
H	-4.28002794760426	-6.73016901898832	3.89574860534002
H	-4.45695634239915	-0.84949449038204	3.43062686816431
C	-2.60722436061969	0.44722804354782	1.98123832212769
O	-3.39763995410970	1.18707330204451	1.44601261761177
O	-1.52382502524937	0.85675033999574	2.61288954820339
C	1.50119442628325	-0.79019223302218	2.72294002390319
H	1.98046743190843	-0.90295302870174	3.70897507617248
H	0.47647032861236	-0.43816702654676	2.92415529537576
C	2.23258554217035	0.28009657452559	1.91104146408837
H	3.32317697591124	0.13882194924303	1.98170355558143
H	1.98796541721361	0.16578342818612	0.84849324309213
C	1.85082864909405	1.69112061239749	2.37114606871832
H	0.77795293868963	1.69762227345983	2.63378825415929
H	2.38981793308199	1.92893104600039	3.30002279076376
N	2.14914861232649	2.74828281936076	1.41121764158020
H	-2.23282704632783	3.43763035404281	1.55634005206292
H	2.56708898280913	3.57237702585269	1.82893987069875
C	-0.22056847996179	3.48788334697944	-2.04640649460981
C	-0.63468960132842	2.34556308763736	-1.12639592826529
C	0.53688580421216	1.90401358580831	-0.25721046551618
C	1.25078979705228	3.06226857799672	0.39561501027872
C	1.04032834311899	4.33271145784336	-0.02178654220077
C	0.20284901562329	4.70490078787436	-1.22117913860807
H	0.17059927776274	1.20940844793577	0.51037880886069
H	-1.46135362624000	2.67345984816104	-0.47394733354815
H	-1.01316358539659	1.47530696559274	-1.68220121610788
H	0.62192171749402	3.14882688442097	-2.67695781506564
H	-1.03524247034344	3.76899784265609	-2.73040636266146
H	-0.70798264877220	3.72540557109978	1.46750059072241
H	1.54887668767117	5.14459941161673	0.50992133444963
H	-0.70071758895587	5.25115992400506	-0.89214238217517
H	0.75697154345817	5.41599739272029	-1.85525583027783

H	1.27659219361284	1.33803808794090	-0.85994137884684
O	-1.40653348757701	3.39229585144132	2.06060086765295
H	-1.44051993686310	1.85186209030505	2.48147124663979

93

TS18-1

C	-0.10569507367965	-0.19623996158764	0.03001476868105
C	-0.09206095485691	-0.06645056181798	1.41138548911717
C	1.12433926084349	-0.00617150822921	2.09001435386874
C	2.33094997725661	-0.11483059126580	1.40686060923742
C	2.32052404134437	-0.24485382935444	0.00917652519764
C	1.10147488175515	-0.28231468960921	-0.65345082656200
F	-1.25130340020955	-0.20820297660183	-0.63190811643268
F	-1.22647710273522	0.03645138554370	2.08032159005059
F	1.07806647953434	0.16674533437073	3.40113031067796
I	4.08272804821648	0.02148746756945	2.54487109522863
F	1.05373552419147	-0.35966429826318	-1.98406030991218
C	3.61303434839934	-0.24916650774512	-0.78204385412507
O	4.48604415294718	-1.06213716081433	-0.57615379550375
N	3.70845784259853	0.78497692734015	-1.66070349470103
C	5.00257809431485	1.23869102687228	-2.12376294029607
C	5.03888731241667	1.56075672138927	-3.62460669442099
C	4.15513721352053	2.71709552104997	-4.02745126482151
C	2.80289801801940	2.51767038195504	-4.33574868821004
C	1.99205928587233	3.58685703784228	-4.72589094246587
C	2.52629920537682	4.87356951755883	-4.81845471752655
C	3.87226009434905	5.08443773323803	-4.51222678576544
C	4.67633239634607	4.01667698911862	-4.11235872690147
C	5.51506500276168	2.44918864561571	-1.31620182030552
O	6.58813570335842	2.96145585078774	-1.59130589930140
N	4.68522107663308	2.87510385911933	-0.34696186093972
H	2.91034516389380	1.42344619762077	-1.72798310381435
H	5.71152984617171	0.42128749318648	-1.93100317686944
H	6.08216526309924	1.79166826546514	-3.87626423865865
H	4.74770149618271	0.65274371148857	-4.17113061705457
H	2.38985127836003	1.50573724556141	-4.28852599248703
H	0.94460493641323	3.40958592597226	-4.98017919561309
H	1.89933538435476	5.70696809683396	-5.14071915151373
H	4.29972039174095	6.08612153073946	-4.58598377879264
H	5.72906273124339	4.18023270377748	-3.87022698638040
H	3.85334654003487	2.33764702286001	-0.11978032084749
C	4.92833978442714	3.96170079602012	0.57470470689887
C	5.13154042232175	5.31354303022062	-0.14389323717368
C	3.72473395416284	3.93250316162290	1.54019792751134
O	2.90937710291731	3.01587723650059	1.48684437460187
C	2.36822765642958	5.22314577484093	3.11789012877967
H	5.75408908142798	5.96064169021184	0.49330033695831
H	5.83539501995936	3.74703279442727	1.16756535894677
H	1.68585033437411	4.39190514739779	2.90696635940726
H	5.74550007435668	5.08956057096054	-1.02668719710394
N	3.59841059053746	4.97043405874790	2.37976092815708
H	4.24259603438295	5.75486617057586	2.31983184783320
C	2.62543454600313	5.28841992034528	4.62908799368113
H	3.32383288482445	6.11623474910868	4.82309130929719
C	3.17164287567573	3.98604985497019	5.15738111850460
C	4.54774360867595	3.80150049839623	5.33067985149176
C	2.31181033260173	2.91545066357698	5.43802736212802
C	5.05358705534178	2.58332643350089	5.78932554504861
H	5.22870531977090	4.62765568384219	5.11193414454922
C	2.81165425586103	1.69898608296132	5.89921261362612
H	1.23490434225036	3.04208079077953	5.29612044967995
C	4.18598149667795	1.53061236919675	6.07965393922589
H	6.12955826665189	2.45958542865441	5.92562604068053
H	2.12683884798396	0.87643373642578	6.11240593928252
H	4.57860405533319	0.57862497186566	6.44178171374639
H	1.67314518902865	5.53420401608514	5.12308623394462
C	1.78969104871702	6.56168832555156	2.60373455919926
O	2.54834490459304	7.52417122323797	2.60716941183579
O	0.57514661094860	6.56508384071170	2.19584507115212
C	3.86668343293738	6.08964478432719	-0.52818374619938
H	4.14695698848237	6.86562592085230	-1.25885118886149
H	3.49853054587304	6.63823023155667	0.35379799780169
C	2.69718918873932	5.27396612372793	-1.08888376747078
H	2.90994788176627	4.93242441650191	-2.11469132214437
H	2.53023874758355	4.36736861540202	-0.49380135422329
C	1.43887500696037	6.14010293268638	-1.05523870557302

H	1.34185427306200	6.60799821159447	-0.06319359676732
H	1.52353855345862	6.95351933143727	-1.78876235922893
N	0.18055564782744	5.45185798950691	-1.32762817389567
H	-0.29016009453541	5.68057897785129	-2.19775197140952
C	-1.97813309921084	2.79452403638194	1.07586161243048
C	-0.77004884878189	3.44655520158105	1.74488328445674
C	0.01898830913633	4.38592640849572	0.82609179776261
C	-0.50252873760469	4.73202951403533	-0.44992430241514
C	-1.88146628240658	4.31668348091440	-0.88888543961634
C	-2.75187909914847	3.82543891739680	0.26184116933497
H	0.20998825376328	5.49284138190720	1.48795728917765
H	-1.11831545848324	4.01398400274729	2.62498086915494
H	-0.08782595051241	2.67550076417253	2.13778350219743
H	-1.64870062232789	1.99220885290487	0.39466192426486
H	-2.62167443443074	2.32452223250331	1.83208220848664
H	-2.35341051848626	5.14016524636602	-1.44629911610813
H	-1.72352525515679	3.49009796196170	-1.60508876236322
H	-3.03720833747529	4.67375839303998	0.90524415545009
H	-3.67931535309992	3.39896690289762	-0.14420938156439
H	1.08496437549255	4.13583884402895	0.77319139533563
O	1.16086389728293	2.40700997490729	-1.67171783249154
H	1.37450120462495	3.22988266327133	-2.13425856498862
H	0.70382789874321	1.87702352079916	-2.33960060891678

93

TS18-2

C	-1.58245324049071	0.99694489581490	-3.73962497043000
C	-2.78685163061492	0.58266803549196	-3.18889334509435
C	-2.89495691890928	-0.69235585864941	-2.63498924205340
C	-1.82881295239886	-1.58399331234106	-2.67308284677650
C	-0.60863242608424	-1.17127185047320	-3.23559349171104
C	-0.50310878017872	0.11764256046368	-3.74815907310025
F	-1.45933812479364	2.21453381931741	-4.24117191103199
F	-3.82185532206926	1.40139325648370	-3.15724284677405
F	-4.05116842503119	-1.00806168979900	-2.07479774067848
I	-2.13993246177409	-3.45776725508522	-1.79250510713805
F	0.64506874631432	0.55175875190383	-4.26078824748747
C	0.59436385731685	-2.09159296558982	-3.24744379780154
O	0.52700210646834	-3.22306412558432	-3.66966174072746
N	1.70821946026927	-1.55770514476644	-2.67347313553056
C	2.76735701755989	-2.40375943555583	-2.16473891278201
C	4.16736670913354	-1.83011488577782	-2.42526721128425
C	4.43304101665546	-0.51686787339801	-1.72645695343072
C	3.99723916835617	0.69700103990534	-2.27770652217202
C	4.25757591782111	1.90889869308281	-1.63432734076667
C	4.96767358087701	1.92574919548538	-0.43287015220540
C	5.40149545468280	0.72311521914840	0.12865553208822
C	5.12896186268119	-0.48757649765713	-0.50930694345297
C	2.59341295517270	-2.70422249211247	-0.65869898877950
O	3.45225830613766	-3.32546868389859	-0.05525549519865
N	1.46543295664528	-2.21179867149432	-0.11542389425403
H	1.66705907197019	-0.60110129899886	-2.33097473259258
H	2.68135494365194	-3.36631697256463	-2.68793061640116
H	4.88983793598309	-2.58143935403537	-2.08226729956554
H	4.28458317321333	-1.71226553863529	-3.51187919927111
H	3.46871645744515	0.69441339945600	-3.23523285335257
H	3.92069990942962	2.84402882344588	-2.08597071074302
H	5.18887357571577	2.87314144738790	0.06234637833728
H	5.95818998456886	0.72721369602382	1.06752752447058
H	5.46414261467925	-1.42959350324186	-0.06949848994947
H	0.77699002450325	-1.76182027779141	-0.70973277318246
C	1.06907095495654	-2.24322125546418	1.27448832936367
C	2.14576558844328	-1.64766776519864	2.20633179974614
C	-0.27281754553976	-1.48089674938841	1.31434720265699
O	-0.81784874785303	-1.12172974565552	0.27509669374951
C	-1.81179355098146	-0.19125623709930	2.71219961103579
H	2.01022959511577	-2.07045306682173	3.21418891755019
H	0.87293365774713	-3.28410584428757	1.58738213531615
H	-2.10603501923977	0.15701427077380	1.71583972415478
H	3.10320505835386	-2.04590019081746	1.84493971919688
N	-0.76226396273812	-1.18483626766700	2.52903395231724
H	-0.22842479353741	-1.42370231906559	3.36060884129812
C	-3.04162500358879	-0.78081071533799	3.41433336722329
H	-2.72816164187255	-1.17366680236618	4.39282655943264
C	-3.68786631143042	-1.85584526015620	2.57715888831778
C	-3.42551606636010	-3.20938294195487	2.81246078247546

C	-4.52821575495197	-1.51100902790860	1.50950288842067
C	-4.00113326774055	-4.19798855132898	2.01108147522845
H	-2.76935008034956	-3.49061334263704	3.63964711657955
C	-5.10651176229254	-2.49387587398815	0.70893877638825
H	-4.73468280538662	-0.45586522514695	1.30902883473647
C	-4.84625298599872	-3.84258306034602	0.96035927193935
H	-3.79155727298873	-5.25004676137426	2.21297379174798
H	-5.75750313190487	-2.20673042719786	-0.11837900685027
H	-5.29917751148079	-4.61362826095952	0.33438781644160
H	-3.75221463473376	0.03964595394856	3.59740293266679
C	-1.20480253942090	0.95976824390236	3.53510642481229
O	-0.69944605724731	0.65683736852110	4.61690727811948
O	-1.23540237361344	2.14039435094652	3.04962995286241
C	2.19000515915758	-0.11915948244880	2.31947191617520
H	3.16853439226123	0.16580896842873	2.74134688471056
H	1.45568396513568	0.22095044849707	3.06650954243169
C	1.95815191455618	0.68464130433368	1.03532111905344
H	2.79629652295949	0.56901182001296	0.33344131705553
H	1.06986531541539	0.32484238302065	0.50340171723564
C	1.76120777520522	2.15033202954106	1.41458642588732
H	1.08740798616170	2.21805926031951	2.28200136590550
H	2.71694489181684	2.58283821316502	1.74094067903866
N	1.23125307077369	3.03313720894624	0.37781876918460
H	1.84924319749008	3.76854938705002	0.04980588286570
C	-2.67919327310343	3.39578069884174	-1.12461669522105
C	-2.46888330066803	2.30683854777110	-0.07242245183581
C	-1.02596929836290	2.19034897625926	0.43307307773932
C	-0.04166387112335	3.13085393362212	0.02736681161204
C	-0.39950073712209	4.33440311801024	-0.80765826027681
C	-1.89094791481138	4.64997701829836	-0.76504797365991
H	-1.11771864054184	2.24698907577528	1.75937738139880
H	-3.12950782219378	2.51594032120051	0.78717787026484
H	-2.79684835054105	1.33086561983458	-0.46639452530024
H	-2.33369950967058	3.04788695929599	-2.11083648637101
H	-3.75081063849948	3.61647481558007	-1.22869471516046
H	0.21367040422775	5.19220353989324	-0.49316360409544
H	-0.10977728548705	4.09329085270733	-1.84649546246748
H	-2.17110145159170	4.99895315566612	0.24234476972370
H	-2.10738368566080	5.46814843941751	-1.46547219143892
H	-0.64587587368670	1.16286774895948	0.44677008915975
O	1.60266573246408	2.24095889254779	4.71015811831632
H	2.18055379246342	1.54928535908114	5.05189683173832
H	0.71742114305703	1.83693665240944	4.78609267830651

93

TS18-3

C	-1.32151373141366	1.16377737819612	-3.87451419266943
C	-2.40576014958353	1.14503255693196	-3.00788104176759
C	-2.67286312061277	0.00066840573912	-2.25764842972021
C	-1.87544016299024	-1.13716604704992	-2.36615164977776
C	-0.74941422929581	-1.10508667654558	-3.20751331078730
C	-0.50922313611292	0.04011800847212	-3.96427456380732
F	-1.04953263069205	2.24802973348219	-4.57743177145886
F	-3.16621182000248	2.21586311595689	-2.88102028798257
F	-3.70834278829746	0.04550676805046	-1.43591464127169
I	-2.43480990272579	-2.77866832462914	-1.18023420406357
F	0.56053137837463	0.11686343328066	-4.75377321324857
C	0.31531454575839	-2.18453184804950	-3.12094726635158
O	0.08325536088116	-3.36944153149734	-3.15507437056817
N	1.54387705378724	-1.65549012877483	-2.85215411830629
C	2.63393382626841	-2.40095113477937	-2.26805878566806
C	3.99026842329454	-1.79264218145427	-2.67638715160726
C	3.97155684135936	-0.28811823103615	-2.54788596173682
C	3.76070509272295	0.50864014258627	-3.68107843249390
C	3.63957397643493	1.89469646799115	-3.57351402219509
C	3.73003473316953	2.50576693090965	-2.32363303315699
C	3.93440498677311	1.72412829291502	-1.18281494897754
C	4.05297042463169	0.33576206269936	-1.29311610061713
C	2.54158715715749	-2.49023280378828	-0.72527263612035
O	3.34433385579398	-3.15574746770696	-0.10279746681556
N	1.54175903243409	-1.76695271903528	-0.16169768327954
H	1.65304466592170	-0.64736162487505	-2.90722251442707
H	2.57550856297266	-3.44152836988412	-2.61767603388318
H	4.75871194048129	-2.24312289955660	-2.03348133743429
H	4.20298691338334	-2.07333925494536	-3.71624379994425
H	3.68020380668660	0.03140505195226	-4.66069006607322

H	3.47659775444190	2.49668707624494	-4.46869241304487
H	3.65667555702912	3.59109034513838	-2.23885539233388
H	4.00406408559054	2.20067740465749	-0.20330784265412
H	4.22155804160894	-0.27185363041831	-0.39897822872966
H	0.85071367801897	-1.41652423366161	-0.81146816334299
C	1.05075776626644	-1.96846352206689	1.19141371642750
C	2.05287564805574	-1.59875242149468	2.31785333457221
C	-0.28078148961607	-1.21410971017798	1.27905546191123
O	-0.62070445381337	-0.38539993889912	0.40286070418426
C	-2.33298880780587	-0.83015309923839	2.54505041912337
H	1.89311839690874	-2.31712453631273	3.13608867001931
H	0.81004990471198	-3.03947122638713	1.31007589177037
H	-2.75580884590481	-0.55636232299700	1.56472066103708
H	3.05565931240978	-1.81632734541880	1.92287225567253
N	-1.08266413045572	-1.52491991781055	2.27415949048023
H	-0.73768630678518	-2.17133440481941	2.97810924088305
C	-3.30766655190327	-1.75425467746480	3.27477751642796
H	-2.81692885887512	-2.15758677993485	4.17675692756584
C	-3.82097644403257	-2.87089561624605	2.40139849207947
C	-3.08754501715547	-4.05022708271899	2.21688522150349
C	-5.02210579186063	-2.71906201013917	1.69610154374828
C	-3.52752345004224	-5.04034113481936	1.33554811202424
H	-2.15828909489460	-4.20091796519042	2.77253215793021
C	-5.47306219934823	-3.70848602533176	0.82318933028056
H	-5.60635547257151	-1.80587881667655	1.83313742977505
C	-4.72337469423809	-4.87106963395027	0.63344843287443
H	-2.94033185138360	-5.95131773297195	1.20417166590891
H	-6.41130955606480	-3.56955505296975	0.28331213083943
H	-5.07161727969238	-5.64497861119188	-0.05264986483689
H	-4.12851060397672	-1.11128463621177	3.61393020040557
C	-2.08985696121725	0.53021645633267	3.30469065228107
O	-3.07352885755461	0.98647470007315	3.89075379472715
O	-0.94094812499790	1.01092481639887	3.18245940214830
C	1.96530859313199	-0.18708107264543	2.89737184459521
H	2.69843057747753	-0.10966943126228	3.71668038567592
H	0.96903006058766	-0.02559942134663	3.33601034557186
C	2.20917201690191	0.92895050312892	1.88927126700647
H	3.24855824915487	0.89718248634151	1.52094534622373
H	1.55885137784472	0.76785625791938	1.01976404874998
C	1.88571951693955	2.31222999204246	2.46157184517377
H	0.88745896063271	2.27624421375473	2.92447354389840
H	2.60907378063623	2.57853082227265	3.24542420900256
N	1.90829953017723	3.36648135965853	1.45808390191690
H	2.76178668577014	3.89995972331117	1.34481370620107
C	-1.08861227665424	4.24863317597502	-1.41287723704548
C	-1.49162472281161	3.38840024655638	-0.21824733476325
C	-0.28971998639077	2.92454551479799	0.58395344786280
C	0.88912159936720	3.64563408091585	0.62105073807908
C	1.15829329968048	4.73816572136104	-0.38975196262880
C	-0.12127484753142	5.34235457382335	-0.96500691760628
H	-0.48597605796343	2.20026538590850	1.38592453088450
H	-2.17696547013032	3.95719922804060	0.43399262647777
H	-2.06868870937089	2.51086448102127	-0.55398386178587
H	-0.60081744764981	3.61964091153464	-2.18046728764648
H	-1.97898125121905	4.68720729344996	-1.88524530914861
H	1.78807824742668	5.51050420339291	0.07773164400897
H	1.75821614966131	4.29049947602224	-1.20149775156890
H	-0.61028423928382	5.95866389698525	-0.19289486484388
H	0.13376694928401	6.01215312557749	-1.79841946125777
H	0.31891654707608	1.98067665352595	-0.45709847924949
O	0.54603113358051	1.08816751807254	-1.12234584605040
H	1.51742459049555	0.99480278289039	-1.11363094371655
H	0.09963693576598	0.33708047809266	-0.47391408946445

93

TS18-4

C	-1.58370237996938	0.99540677710247	-3.74164037607360
C	-2.78762667190745	0.58156502183315	-3.18955220922053
C	-2.89534203460509	-0.69308272736158	-2.63474090975217
C	-1.82925727671690	-1.58478891382007	-2.67313956682516
C	-0.60951826654154	-1.17247970496559	-3.23688388320029
C	-0.50441853087819	0.11603523322227	-3.75057328990042
F	-1.46093179774789	2.21274739442618	-4.24385636823357
F	-3.82248832339976	1.40045673346951	-3.15735930921743
F	-4.05102743168165	-1.00828208702830	-2.07320832122244
I	-2.13976484860397	-3.45803436516447	-1.79122932375814

F	0.64333535589386	0.54971630563875	-4.26448885817963
C	0.59366419792010	-2.09259199779756	-3.24852190006926
O	0.52648278625762	-3.22431716856042	-3.67010984961312
N	1.70748998806042	-1.55812263479944	-2.67501180542599
C	2.76685868609210	-2.40360148737118	-2.16578992396986
C	4.16672472430566	-1.82947610865425	-2.42609883808738
C	4.43245733682734	-0.51671613849395	-1.72638382948535
C	3.99698097556636	0.69760646643293	-2.27686990063910
C	4.25792243677738	1.90906161976366	-1.63289507229874
C	4.96826651327905	1.92499193744586	-0.43157364349203
C	5.40161576086067	0.72187622395669	0.12929036011725
C	5.12846698165778	-0.48836679084521	-0.50925556799164
C	2.59290026817179	-2.70362683485685	-0.65969918009136
O	3.45124425972810	-3.32565108411595	-0.05634003039346
N	1.46571462362398	-2.20972898048672	-0.11612348788580
H	1.66621147834894	-0.60124072384190	-2.33332533911941
H	2.68142615729931	-3.36636961701618	-2.68866503954523
H	4.88934635916136	-2.58093883592052	-2.08368132821548
H	4.28386181648785	-1.71090491499004	-3.51263460866836
H	3.46835717460360	0.69575381432073	-3.23435572458994
H	3.92136475971320	2.84457725323107	-2.08399305200778
H	5.18998418656617	2.87203592893498	0.06407625125422
H	5.95843501279482	0.72525118969154	1.06808380551573
H	5.46328659229071	-1.43076626632233	-0.07000346720387
H	0.77725821093143	-1.75958840354297	-0.71028468964376
C	1.06933891827342	-2.24205121162921	1.27376908026415
C	2.14609678360211	-1.64714188678652	2.20595499476938
C	-0.27267358240274	-1.47998759918152	1.31410297016687
O	-0.81801233512060	-1.12074541523823	0.27504652458015
C	-1.81171217001372	-0.19101349601800	2.71251250678675
H	2.01081778287317	-2.07074165641981	3.21351286223883
H	0.87324672085317	-3.28318320256359	1.58590263414364
H	-2.10625820501421	0.15737193502344	1.71628037147677
H	3.10350740317043	-2.04502206328006	1.84407818560745
N	-0.76200024734145	-1.18434131673041	2.52895546021341
H	-0.22793712917093	-1.42325116757072	3.36037489815533
C	-3.04123300264066	-0.78101304329219	3.41481614740707
H	-2.72742230756227	-1.17421448331295	4.39305410412375
C	-3.68757741118854	-1.85577233084808	2.57737198186917
C	-3.42489534038909	-3.20937720151691	2.81193802171836
C	-4.52852989809320	-1.51059889705314	1.51030264031125
C	-4.00079889720979	-4.19771718576082	2.01043828764957
H	-2.76827110636939	-3.49086800273621	3.63866290824935
C	-5.10713528842932	-2.49320231141156	0.70963818573153
H	-4.73522969287107	-0.45538500239951	1.31037326697800
C	-4.84655965816835	-3.84197872915493	0.96034290156716
H	-3.79096741244870	-5.24982584241939	2.21176180139468
H	-5.75864614622330	-2.20578708551723	-0.11718543216651
H	-5.29972779337304	-4.61282012677729	0.33429850544657
H	-3.75188176009641	0.03925516155065	3.59850610770515
C	-1.20481867812067	0.96001180687176	3.53549587929141
O	-0.69938016319897	0.65701316318678	4.61724239639768
O	-1.23563129303865	2.14069844231536	3.05018162627511
C	2.19024841607887	-0.11872851483631	2.32020591194839
H	3.16873092991052	0.16599271215289	2.74234464974541
H	1.45590752193365	0.22097511454587	3.06739813810395
C	1.95846353360404	0.68565683557169	1.03644713573725
H	2.79669825427235	0.57041496848399	0.33461653967555
H	1.07032161279348	0.32586828710781	0.50425114091861
C	1.76122193769253	2.15118759879579	1.41618103422858
H	1.08728530312497	2.21856362951179	2.28350161610482
H	2.71684060006160	2.58374451607639	1.74278721967744
N	1.23129829437594	3.03416813876575	0.37954225165423
H	1.84917449556714	3.76983636464135	0.05189297384006
C	-2.67784321143486	3.39428451135831	-1.12638767087934
C	-2.46816362916033	2.30620136291005	-0.07319781654568
C	-1.02566363110015	2.19060801258299	0.43366068325531
C	-0.04146762699532	3.13141558719364	0.02843322554699
C	-0.39920077941483	4.33475962097322	-0.80695157940989
C	-1.89092701601598	4.64921462019437	-0.76644899853371
H	-1.11825688197071	2.24749508341266	1.75990124805985
H	-3.12971785561681	2.51570813549382	0.78559001065465
H	-2.79536244439585	1.32978621575309	-0.46671989220181
H	-2.33085910650336	3.04598240999910	-2.11194688284543
H	-3.74948445861686	3.61420476476214	-1.23195099641950
H	0.21286172737358	5.19301708194668	-0.49152943058734

H	-0.10776806421616	4.09397684203770	-1.84538939218074
H	-2.17267550025033	4.99844550745528	0.24043174455321
H	-2.10705209254736	5.46691902385473	-1.46750814384994
H	-0.64514173826879	1.16331481229404	0.44795008904277
O	1.60248830122830	2.24122190623910	4.71152018580118
H	2.18027992213772	1.55013096153067	5.05460487649012
H	0.71729600542917	1.83700796255967	4.78711613924064

93

TS18-5

C	-1.67150898433242	1.26452514176265	-3.55001912441382
C	-2.86769303109381	0.74938101316329	-3.06786248158653
C	-2.93738168712495	-0.59024621301520	-2.68316485531523
C	-1.83598128754698	-1.43346591908529	-2.80574119471040
C	-0.62556198798475	-0.91877996000114	-3.29658813344244
C	-0.57110635932590	0.42131998628471	-3.64681945172244
F	-1.57039610260515	2.54003872481517	-3.88802098389387
F	-3.92378783411395	1.52885228439028	-2.95593119102204
F	-4.08817251360503	-1.02494496751152	-2.20712777877204
I	-2.06915499604826	-3.40481743775191	-2.15425798280244
F	0.58449749889961	0.96059173467051	-4.09011984529783
C	0.60635567893235	-1.79649532188534	-3.41723032461863
O	0.59582749938969	-2.81363596376267	-4.06855240928591
N	1.66819291313116	-1.36613364922590	-2.67970760712656
C	2.70541149331352	-2.28213325848533	-2.24215543785049
C	4.12530313285547	-1.75818760902058	-2.50053365033763
C	4.45325578480211	-0.49375290310670	-1.74240221532290
C	4.14323614726012	0.76781252412567	-2.27201772308249
C	4.46317039350984	1.93505572271510	-1.57171040405968
C	5.08717647597073	1.85723127144789	-0.32505632447719
C	5.39318113908207	0.60712414430116	0.21388008484834
C	5.07874940065378	-0.55671246262840	-0.48915660847662
C	2.54367416205115	-2.65005551708778	-0.74796040682583
O	3.42024441832647	-3.28011292717094	-0.17939081092531
N	1.41410120075126	-2.19235716955270	-0.18478131845211
H	1.55593810108731	-0.51277790700807	-2.13885308053450
H	2.56647127467967	-3.20680535318505	-2.82043621084150
H	4.81887443709232	-2.55540047388767	-2.20464910697818
H	4.23224878589034	-1.59248695156434	-3.58169395182816
H	3.68088630546281	0.83152499331912	-3.26210174178472
H	4.25397218418136	2.91135344340534	-2.01580277768476
H	5.34881942874497	2.76846500108120	0.21596067465009
H	5.88633702155587	0.53576677715425	1.18495595895931
H	5.31692973396657	-1.53571520162294	-0.06833750480826
H	0.68790322599111	-1.78550446043484	-0.76716495606752
C	1.00077685094133	-2.25545882202105	1.20096564758066
C	2.07887783326783	-1.73219582533474	2.16697979519395
C	-0.33975983532230	-1.48127845975936	1.22982085585558
O	-0.89802804816793	-1.19145897915954	0.16995246880825
C	-1.98347799719852	-0.30205614361363	2.60099389827042
H	1.89230416283984	-2.16876625184873	3.16075295480797
H	0.76693224912281	-3.30035867697237	1.47381389997474
H	-2.27494161214712	0.02696657494904	1.59225090453230
H	3.02475359805738	-2.17281986922245	1.82440676393669
N	-0.82281304078412	-1.15757094652558	2.43085788085485
H	-0.21851961432604	-1.24913336660179	3.24176961879157
C	-3.17065691262782	-1.05095999772931	3.21757302915298
H	-2.88178599261876	-1.42513982557974	4.21269875965579
C	-3.64827474977827	-2.18823487857645	2.35169695836354
C	-3.17232556665390	-3.49121070282924	2.54015228932302
C	-4.55069115661268	-1.95514098874543	1.30413970723052
C	-3.59641953349810	-4.53681875540790	1.71704718905121
H	-2.46799027286912	-3.68844398917133	3.35220731336705
C	-4.98011365816596	-2.99534935778152	0.48192992016202
H	-4.92507259737952	-0.94106492405783	1.13958777938330
C	-4.50635548404332	-4.29273247543898	0.68850154522035
H	-3.22073912429000	-5.54776556769740	1.88694793480801
H	-5.68628227166424	-2.79321877486240	-0.32563381814154
H	-4.84527589523095	-5.10947280091699	0.04812630054429
H	-3.96301002542629	-0.30450660901243	3.35983176322056
C	-1.62405950963564	0.99289932472450	3.40130682546590
O	-2.58207008151822	1.74079068133468	3.63359668952068
O	-0.41254196415867	1.15775577713544	3.67333017987122
C	2.19391280759938	-0.20940426453619	2.31531535729530
H	3.18999922919505	0.02040262599655	2.72992080670579
H	1.45470606897747	0.14624449798352	3.04867498171076

C	1.97471859738753	0.63270608106759	1.05786793627583
H	2.73649114045892	0.43668596819305	0.28502102049690
H	0.99947936570987	0.39498259481308	0.60948520972248
C	1.98775109182689	2.10698185030130	1.43825139977767
H	1.33261375621853	2.26242776615444	2.31178386978415
H	3.00642143946300	2.39731436542511	1.73262630989046
N	1.60254502033263	3.00136411873325	0.33474395913248
H	2.18935095768475	3.83220993395826	0.28843353949265
C	-2.46672220762386	3.63179527099447	-0.79388224141539
C	-2.16940651201161	2.92547414838744	0.52355965481912
C	-0.77870344319857	2.29654600394064	0.48969558911445
C	0.27030050016253	3.28748145872309	0.05259453067824
C	-0.05197018155350	4.36850696764630	-0.72144764842345
C	-1.47347812880533	4.76461295882287	-1.04539837460373
H	-0.50659329934406	1.89567468531887	1.47663206982767
H	-2.22206235617934	3.63711720647021	1.36262532264561
H	-2.91365625133876	2.14657548434472	0.73913537050067
H	-2.39689153661502	2.90022425488239	-1.61327867177166
H	-3.49379901073378	4.02257216791493	-0.81173876719801
H	0.73103057299930	5.11544419555599	-0.90886812055484
H	0.73850443336910	3.38247361176616	-1.89194675658564
H	-1.74052563961248	5.63566525838721	-0.42234769685830
H	-1.54070081406443	5.10912526543206	-2.08921732779594
H	-0.76733409477965	1.43007433169840	-0.19998817749535
O	1.56984670886786	2.88149867646170	-2.35509448113531
H	1.28678655972207	2.19896943429634	-2.99866337455960
H	1.98826542197307	2.46666457594664	-1.54912846838856

93

TS18-6

C	-1.69748489786474	1.13915739883811	-3.17615278481233
C	-2.82189713979315	0.81276953590492	-2.43175498080993
C	-2.92148352278145	-0.45293927726452	-1.85702802838732
C	-1.92939038106612	-1.41118619548125	-2.04366970392967
C	-0.79365065480275	-1.09119239300853	-2.81071114986626
C	-0.70443106069600	0.18444356010659	-3.36154336941256
F	-1.55994244600424	2.35087270538929	-3.68941381627367
F	-3.77434915080571	1.70490609604752	-2.24675715580227
F	-3.99526921393753	-0.69956175046978	-1.12534128319484
I	-2.21159474187385	-3.21279427191074	-1.00953894083084
F	0.36959405387292	0.55794401101566	-4.05920938664683
C	0.35270944758290	-2.07178411786660	-2.94625613599618
O	0.17420617968243	-3.25499500095301	-3.11590380323856
N	1.59271524387218	-1.52753330378414	-2.75969352472905
C	2.66197745576737	-2.34625626529686	-2.23139722839564
C	4.04051242186146	-1.74081901317302	-2.53298094789830
C	4.13968009005232	-0.27340393095083	-2.19095660472291
C	3.96307737299201	0.69869606253227	-3.18779607589432
C	4.06500086534750	2.06207072800654	-2.89097182733026
C	4.34268524107434	2.47264330191383	-1.58262629819980
C	4.50063284283445	1.51299681972714	-0.57918199849903
C	4.39289865491118	0.15313331242953	-0.87941399826608
C	2.48515915363517	-2.64293801454465	-0.72179729903376
O	3.14450582737725	-3.51761609796228	-0.18982670400466
N	1.57334910468162	-1.87876725301400	-0.09196045654784
H	1.64765392939658	-0.52829833354794	-2.59515348562329
H	2.60245695229766	-3.33335233646861	-2.70833724286268
H	4.78086757934583	-2.31965313845686	-1.96324588669401
H	4.25211985039905	-1.87874603108805	-3.60181452182574
H	3.75665435674442	0.38143887918532	-4.21282795381763
H	3.94487942944120	2.80093742757400	-3.68520311931835
H	4.44331378114967	3.53464929136850	-1.35068568607971
H	4.72502441991700	1.82422392064597	0.44268138218070
H	4.52615380437282	-0.59061362349517	-0.08870534210461
H	1.07076741088566	-1.16303763019917	-0.60750721674869
C	0.98798535719881	-2.14211683904897	1.20806887446210
C	1.93845841557650	-1.95515902273051	2.41760813249251
C	-0.24866373883909	-1.22116656541972	1.26092350665466
O	-0.34894155133130	-0.27284183735626	0.47693417556114
C	-2.39370709396122	-0.74069820039481	2.28614033097575
H	1.62774451771323	-2.66758614819608	3.19696606642548
H	0.63364288542669	-3.18674267348592	1.21508097931558
H	-2.78322176746952	-0.55395371469736	1.27207983315626
H	2.93652742422120	-2.28173398528318	2.08693279160522
N	-1.18639917615983	-1.53447987182290	2.15198320194675
H	-1.00701227620885	-2.29044775146233	2.80317889341485

C	-3.45434289729571	-1.50175404620068	3.08953656483477
H	-3.03338870450738	-1.75977978447032	4.07558505765535
C	-3.96058825347407	-2.73588729129763	2.38540720070602
C	-3.33472036699757	-3.97826490314398	2.54453692886564
C	-5.04140000074650	-2.64474330695731	1.49830868365542
C	-3.75767070143841	-5.09407757381757	1.81853070647246
H	-2.50925820754635	-4.08089129652898	3.25443521119658
C	-5.47909308983097	-3.75831331309881	0.78354979471065
H	-5.53852798741498	-1.68067877455487	1.36469781521303
C	-4.83254200795194	-4.98669579911016	0.93417706352670
H	-3.25493870838290	-6.05353351103366	1.95489718728662
H	-6.32363673527980	-3.66619841731123	0.09822254159044
H	-5.16936190585879	-5.85856972054972	0.37068118838660
H	-4.27684223740194	-0.79310208836498	3.25403768412442
C	-2.14031845584292	0.66307688719966	2.92583206024408
O	-3.06138191836006	1.47559415463789	2.74890169459735
O	-1.06414989385567	0.82152327098430	3.53877899544001
C	1.96284297521201	-0.55069787650687	3.02195821189386
H	2.63047990058859	-0.55221581752092	3.89662848091395
H	0.95370396439480	-0.31218476685997	3.39693025483548
C	2.37920141291585	0.54134160490387	2.02345231325372
H	3.45690640855143	0.75945417515799	2.10465944695977
H	2.22241040747487	0.16335738047241	1.00241777314742
C	1.53320845720839	1.80340397893936	2.17469249173505
H	0.50517477111790	1.49287327314486	2.39389960492420
H	1.87115864102830	2.41674441921653	3.02671770938902
N	1.52808282248178	2.59423576533914	0.94036580576298
H	2.41053534986040	3.06261588248237	0.73743328231894
C	-1.97483875720621	4.17837685735894	-0.85753560084895
C	-2.02283219993305	3.92042528640461	0.64366329229368
C	-0.92089919619907	2.96171183615741	1.08109393107970
C	0.41500941413579	3.28187513691394	0.47168442253168
C	0.52598819059087	4.06202716257128	-0.65168390153573
C	-0.64220782934399	4.81185287760667	-1.25054449807851
H	-0.84756336703040	2.93658545685446	2.17735603681683
H	-1.90820380029155	4.87845799725802	1.17787932060589
H	-2.98175598689005	3.48705927856231	0.95689809325648
H	-2.09448457332723	3.22371915336844	-1.39312061634806
H	-2.80794293067720	4.82197756536198	-1.17452802579208
H	1.53555039950008	4.33892719964682	-0.98672028261309
H	0.74008053824898	2.51678312578374	-1.35887128676998
H	-0.61553740139149	5.86325407102468	-0.91414126789353
H	-0.53972634564774	4.84112396738111	-2.34637361993197
H	-1.16789667820074	1.91973291420201	0.80704052454721
O	1.23421378303513	1.55904719718484	-1.50232170455940
H	2.06171940663557	1.74155385699531	-2.00502713954498
H	1.49427303930805	1.46040709236173	-0.52723264124908

96

Intermedia R + H2O

C	-0.32460375852883	2.31038518900700	-4.68587485676028
C	-1.69109527918947	2.15126954445961	-4.86240454888958
C	-2.26031856091955	0.88288859912578	-4.75014816155687
C	-1.47231340282566	-0.23397235082044	-4.49553139400147
C	-0.08513660229512	-0.08347768992816	-4.33537188978569
C	0.46240438416132	1.19166213082151	-4.42614697139980
F	0.21700111237732	3.51499737853993	-4.73224268421588
F	-2.45504230411124	3.20235823806580	-5.08501831710637
F	-3.57259677313144	0.79332357307670	-4.86974474216817
I	-2.45851093519485	-2.06687098317284	-4.27534500321937
F	1.76746654238392	1.38785911893797	-4.24721115767797
C	0.82139820632701	-1.27644672542314	-4.11040834389634
O	0.79320770493341	-2.23427571705725	-4.85013923791493
N	1.62200218340771	-1.17730581657987	-3.01855288110868
C	2.49268564675551	-2.25460170779623	-2.59955520422374
C	3.97331171430693	-1.83846101146562	-2.56092506151673
C	4.25327640658114	-0.74023551016856	-1.56306397803578
C	4.06809249422873	0.60670413643000	-1.90648095616354
C	4.31237447416682	1.62138076169155	-0.97722777174301
C	4.74914447985356	1.30298429294179	0.31040969675497
C	4.93360660759332	-0.03526602725571	0.66378495157756
C	4.68383658424262	-1.04723812957330	-0.26410334704043
C	2.08944242569543	-2.84985265332433	-1.23869264505663
O	2.81266363960887	-3.66773963196257	-0.69183133708095
N	0.94163028357987	-2.38064894877377	-0.72461033704945
H	1.60331903482091	-0.32369793778623	-2.46668361203571

H	2.37370612692280	-3.05454792212985	-3.34403712641134
H	4.55325824641730	-2.73372400653910	-2.30376234867228
H	4.26035953523624	-1.51713519319662	-3.57208176451458
H	3.75362212247429	0.86381982711108	-2.92077463584676
H	4.18751367058106	2.66637668491403	-1.27129183061388
H	4.95966037276328	2.09561111725028	1.03115706675987
H	5.28069564541786	-0.29346042664757	1.66598451103087
H	4.82231071657834	-2.09488276762155	0.01192590259462
H	0.33836586858904	-1.77844592656290	-1.27957825604625
C	0.40833378663111	-2.72045017922902	0.57529786018497
C	1.36533748732385	-2.34318281072837	1.72477802883898
C	-0.95406125047145	-2.00695357723224	0.65506904518978
O	-1.40137362619028	-1.39847132880966	-0.31113197453596
C	-2.81346961287663	-1.29764495805719	2.03018997904665
H	1.10074689693526	-2.94274176931525	2.61023670395797
H	0.22485488503106	-3.80808608113609	0.62638011126742
H	-3.60180457011462	-1.66680606649908	1.35625674931562
H	2.36140037153328	-2.69089578141888	1.41669438671901
N	-1.59918270598247	-2.06484275832323	1.82722321558617
H	-1.19340191697576	-2.57677258197062	2.60353296582173
C	-3.27108516267843	-1.42017007786437	3.48869976444417
H	-2.53088074955549	-0.91491440917805	4.12879754717315
C	-3.44769178715548	-2.85088328063242	3.93557017827441
C	-2.55389994394255	-3.43667653241860	4.83905420173438
C	-4.48847586089591	-3.63503438460092	3.41908311478730
C	-2.69204987483252	-4.77406824023740	5.21924461022115
H	-1.74368291410280	-2.83305989563599	5.25718607004430
C	-4.63090783105163	-4.96815997127453	3.79751301931593
H	-5.19496130084285	-3.19021937146645	2.71351947396729
C	-3.73120458290061	-5.54306406855520	4.69908573408594
H	-1.98666381818099	-5.21290829212010	5.92710470571749
H	-5.44935059313700	-5.56337938287884	3.38877732561658
H	-3.84360063989468	-6.58721873566367	4.99532601769190
H	-4.21701989640920	-0.86613576284698	3.57488449715663
C	-2.62555257459254	0.20365401192921	1.69832686810282
O	-3.59699117091828	0.81285487355727	1.26882695160861
O	-1.47363140896972	0.68618368038040	1.96716807351177
C	1.39475314197514	-0.86501055502189	2.11993433847828
H	2.27390355212185	-0.69769189381603	2.76501001696920
H	0.50096495122442	-0.62319835414768	2.71489771715657
C	1.41693265376260	0.12844364894759	0.95925114617651
H	2.27608812382468	-0.04994657866456	0.29220797219760
H	0.50221975744180	0.00397222925845	0.37092731074680
C	1.46408423263029	1.53666825340638	1.51378408255419
H	0.69392003789574	1.68125835961517	2.28025434297160
H	2.44445349919521	1.74158082274282	1.96763106671528
N	1.24273029241703	2.59956142803134	0.49380197986083
H	1.60280368286457	3.46722840165894	0.92344224197297
H	1.78776257619046	2.40457165988846	-0.35439459144696
C	-2.11976903588246	3.84873505115891	-1.96186435826930
C	-2.16719164518001	2.43359514726050	-1.38881361532931
C	-0.79183855876977	1.96279877668401	-0.90316036221399
C	-0.24466364512300	2.95794672531140	0.12724113606540
C	-0.16170669722627	4.36367391311157	-0.47765636196860
C	-1.54201474098552	4.82500337962096	-0.94002640432406
H	-0.89539981270504	0.96120263902588	-0.46619483242987
H	-2.85838687010544	2.39284224892394	-0.53218279782595
H	-2.55502104196249	1.71648631607001	-2.12870515906501
H	-1.48458740644046	3.87083444280846	-2.86416612212151
H	-3.12438951341674	4.16707125044003	-2.27665931443321
H	0.24569605299944	5.04908415333908	0.28182284761651
H	0.53449724001395	4.34666850763941	-1.33438722934651
H	-2.20429359054678	4.87701386141661	-0.06235368484387
H	-1.47050190006106	5.83833680903202	-1.36053109019291
H	-0.08286127645431	1.90709644379327	-1.75051754722125
O	-0.95500327814031	2.98766532082069	1.28593445929466
H	-1.26932383498431	1.99968645910818	1.57535700242227
O	1.02800930831789	4.45905516220155	2.52111147920169
H	1.22346809216133	4.28001466343155	3.44834527989387
H	0.12757446855526	4.10540390916969	2.37855017702498

96

TS16 + H2O

C	-0.30757527747846	2.33246278373494	-4.76002362910256
C	-1.67909892681217	2.19947076504007	-4.92026906416065
C	-2.27415961981545	0.94557293045678	-4.78312042692175

C	-1.50728760811331	-0.18123279645636	-4.50771664313864
C	-0.11665700972192	-0.05641815203178	-4.35902810211538
C	0.45751682927138	1.20451399746102	-4.47850482851327
F	0.25832316740871	3.52316786827302	-4.84045565460475
F	-2.42147706120318	3.26358723046313	-5.15236572493602
F	-3.58887048304382	0.87932692458160	-4.89504836093937
I	-2.53022098060343	-1.98799374334934	-4.24059755751648
F	1.76839209427051	1.37243210585590	-4.31896048628600
C	0.77020922541229	-1.26137204265698	-4.12776313823907
O	0.70998213933558	-2.23252407741319	-4.84843173455653
N	1.59468426625391	-1.15519016185591	-3.05506181516922
C	2.45623333860115	-2.23890836299683	-2.63642031127873
C	3.93993619332528	-1.83532718566491	-2.58723912358141
C	4.23440357220312	-0.75283028998832	-1.57612610615298
C	4.06607470503938	0.60050726214551	-1.90188839436600
C	4.33255089602333	1.60026501943943	-0.96311626512632
C	4.77429109893413	1.26068136941715	0.31703961168277
C	4.94325575908321	-0.08425994984048	0.65295106041316
C	4.67146197301576	-1.08151070211350	-0.28444983409316
C	2.03955396917418	-2.84138256365831	-1.28528579159084
O	2.73574936019368	-3.69116298077285	-0.75280745990383
N	0.90818847059362	-2.34463433980608	-0.75762990482325
H	1.60580018829852	-0.28949662216637	-2.52147016403380
H	2.33776028758226	-3.03500695288438	-3.38495704661528
H	4.51047586220472	-2.73999251526206	-2.34123552892549
H	4.23224390388265	-1.50548040516513	-3.59414775823029
H	3.74597237359258	0.87507488217870	-2.91010348184489
H	4.21830044860572	2.64986899072235	-1.24355403690793
H	4.99856464683497	2.04164976681616	1.04604669444324
H	5.29467363062815	-0.35905427882616	1.64928070698454
H	4.80055836587981	-2.13419001561769	-0.02249574459518
H	0.32524406984468	-1.71089345419624	-1.29779933660478
C	0.38513315108325	-2.69842420954869	0.54130914206568
C	1.36623214230625	-2.34990887336917	1.67910418098054
C	-0.96643837815275	-1.97624618992111	0.65654488845714
O	-1.42913195318383	-1.34064017479218	-0.28278106634483
C	-2.82946505596459	-1.31046692167841	2.04743728081290
H	1.11455317487736	-2.96162022916763	2.56011465718383
H	0.18952005765987	-3.78471169513104	0.57706503815372
H	-3.62201380376097	-1.70806129389708	1.39474959386178
H	2.35199758753160	-2.70169699756189	1.34444851488829
N	-1.59746171984785	-2.04469632901058	1.84056812408466
H	-1.20487063772575	-2.58857310546458	2.60216105019886
C	-3.26195512113050	-1.40651760080391	3.51749270475124
H	-2.51853745218170	-0.88126675365088	4.13639722197404
C	-3.41096429915062	-2.83417024280287	3.98439095381299
C	-2.49610067411447	-3.39464260095266	4.88241944621712
C	-4.44903685257344	-3.63698611284562	3.49242596899362
C	-2.61197525213960	-4.72856957326173	5.28140627469967
H	-1.68802308409578	-2.77520952469333	5.28123326950810
C	-4.56828594609537	-4.96681152758214	3.88965502986183
H	-5.17276187561799	-3.21068039203437	2.79307983047096
C	-3.64811312004231	-5.51749978552826	4.78540679880038
H	-1.89130507444082	-5.14901071374393	5.98503622963985
H	-5.38455297719137	-5.57770357656207	3.50080989884721
H	-3.74237171531600	-6.55898303072676	5.09670281005602
H	-4.21558967078080	-0.86699986844726	3.61262737109972
C	-2.69084695423739	0.17037954690783	1.66983236177464
O	-3.60186243694973	0.77160830956540	1.15441020200773
O	-1.54301049011654	0.69508700582575	2.03126657825215
C	1.41569229371083	-0.87815946783569	2.09381706608956
H	2.30281108989324	-0.73026782697778	2.73255174923144
H	0.53599329315718	-0.64538840035393	2.71448422598020
C	1.43105052795314	0.14695485667726	0.95725769197795
H	2.30116299181385	0.00502390903892	0.29695568269744
H	0.53408463851451	0.01707066933684	0.34586200312602
C	1.43848856947230	1.53506283333960	1.57220695675694
H	0.66436697070274	1.59472305786178	2.34864026463792
H	2.40599350004857	1.70678087712401	2.07168007595637
N	1.21973897825468	2.70392399254972	0.69448087076211
H	1.24351007012615	3.58596012017021	1.51351820851349
H	1.91569795175287	2.75879528405417	-0.05238759688404
C	-1.98135582994169	3.82827195517682	-2.04091749318623
C	-2.04997193043696	2.43030728408199	-1.43006210724698
C	-0.70586599182319	1.97489339724857	-0.85133086694310
C	-0.19024969917195	2.99229996353359	0.17165461655606

C	-0.08891692562431	4.37903503397071	-0.49113448246085
C	-1.44777617315752	4.82941120398031	-1.01991654835098
H	-0.84872462463132	0.99114038749265	-0.39006477129029
H	-2.78803256827202	2.41938303700293	-0.61276911712202
H	-2.39621990464641	1.68762379328964	-2.16579257704554
H	-1.30486887876401	3.83095354617212	-2.91289736799776
H	-2.97185072839655	4.13330206121336	-2.40980270033310
H	0.29831585530536	5.09589824991845	0.24743509634250
H	0.63494083748068	4.32331659509980	-1.32356451457715
H	-2.14633220458228	4.90205626214241	-0.17196313557673
H	-1.36090078525971	5.83254358550397	-1.46216899093061
H	0.04886533690810	1.87117638506379	-1.65416444925078
O	-1.00284741136574	3.03921351700975	1.29215450513096
H	-1.43439116761176	1.69030698853249	1.72987907079892
O	0.64664905320328	4.30699097897560	2.45178901487126
H	0.86301972062710	4.01581338795217	3.34571744310913
H	-0.34989429258872	3.72888563867016	2.03629720289810

96

Intermedia S + H2O

C	-0.28488926552680	2.31040854027021	-4.73533117728812
C	-1.65765460548215	2.17458590700703	-4.88057683953658
C	-2.24913506188410	0.92035292601870	-4.73298577904871
C	-1.47705403377876	-0.20686568425982	-4.47740733266462
C	-0.08414719990586	-0.08100666670932	-4.35094179056017
C	0.48697283418654	1.18105362245508	-4.47428430095169
F	0.27639621285885	3.50319136221966	-4.81777458654361
F	-2.40641251907714	3.23582471587666	-5.10711180715767
F	-3.56577661456194	0.85454390611920	-4.82214894857807
I	-2.49325926714834	-2.01747571677089	-4.20910304832831
F	1.79948239593647	1.35014083311436	-4.33455469830482
C	0.80118322690240	-1.28945022737845	-4.12634063924868
O	0.73637707779629	-2.25704972857318	-4.85151198562261
N	1.62207261223740	-1.19466506588401	-3.04977360291565
C	2.46291382548330	-2.29238758063628	-2.62397139892325
C	3.95176543600758	-1.90896787990671	-2.56776990370688
C	4.24294484109663	-0.81455818169452	-1.56876519651982
C	4.10143262769178	0.53531383912263	-1.92073454759527
C	4.35311673021197	1.54655257750175	-0.99067604418782
C	4.75389788561289	1.22183870565421	0.30629974228900
C	4.89757779647791	-0.11913908622093	0.66792999220726
C	4.64033939763079	-1.12815261118732	-0.26091366245089
C	2.03174580143871	-2.87894688100931	-1.26923957087495
O	2.71185138262858	-3.73607185182865	-0.72779865996025
N	0.90774767903384	-2.36283165662410	-0.74403035131241
H	1.64398194822855	-0.33038819529276	-2.51410748335955
H	2.33370113926689	-3.08982844830964	-3.36924448953795
H	4.50852725419429	-2.81673926062978	-2.30261434396211
H	4.25782484291999	-1.59691893700632	-3.57627719407047
H	3.81044899316194	0.79690898449405	-2.94119677987057
H	4.25455426351853	2.59291382450275	-1.28765107224559
H	4.96300610839052	2.01205643469704	1.02967220514750
H	5.21681828749386	-0.38193858703829	1.67827024902161
H	4.74934951854206	-2.17801659950840	0.02053215538006
H	0.33554065591462	-1.71825893359785	-1.28215962473685
C	0.39654134012551	-2.69803605700646	0.56464827632281
C	1.37225211730099	-2.30709361655401	1.69523629111860
C	-0.96691719363616	-2.00292392773582	0.67287185985870
O	-1.44103607385485	-1.37189380883374	-0.26311606115652
C	-2.84861433548287	-1.37849997804759	2.05209373417885
H	1.13659312710277	-2.91189535521573	2.58571260762370
H	0.22480325787922	-3.78722294723592	0.62309912463736
H	-3.62894382941421	-1.78797770483180	1.39198026641366
H	2.36424270349053	-2.64271558577114	1.36167445160750
N	-1.60185734543116	-2.08728708337879	1.85564710498860
H	-1.20346389301226	-2.62427661159719	2.61905088739210
C	-3.29438317712752	-1.48128742481945	3.51838005280652
H	-2.56626251198043	-0.94470160610804	4.14550475433111
C	-3.42190244403502	-2.91270707179248	3.98058062729155
C	-2.50225813074075	-3.45970645818398	4.88196669601277
C	-4.44307887111909	-3.73146804357227	3.48002079431187
C	-2.59701099529586	-4.79681032804244	5.27569138983537
H	-1.70726170476705	-2.82787755356719	5.28718524467755
C	-4.54095409584088	-5.06462479964572	3.87191048923849
H	-5.17038277870444	-3.31571937808580	2.77786404743957
C	-3.61616420885537	-5.60205552782888	4.77085725243954

H	-1.87298345218885	-5.20697519575974	5.98171747669071
H	-5.34397521914525	-5.68864993557741	3.47609130633536
H	-3.69346354925842	-6.64625407383590	5.07772787721408
H	-4.25858979870412	-0.95940712257243	3.60480607925656
C	-2.72894686436397	0.09988721871827	1.67300472526785
O	-3.62846781118399	0.70615834048194	1.15164425709025
O	-1.57946431827064	0.63392231440921	2.04991439851143
C	1.39552636156014	-0.82722385103681	2.08557634937448
H	2.28837724706294	-0.65245298921032	2.71005775886910
H	0.52133123570139	-0.60267544296755	2.71715170756483
C	1.38030356184797	0.17705773377499	0.93344494914614
H	2.23155075436580	0.01354062255886	0.25206738569957
H	0.46650839672562	0.03943461336969	0.34573129924768
C	1.41733739097691	1.59847154893195	1.48478597362605
H	0.66543763734854	1.69429270695264	2.28251723863113
H	2.39618064921351	1.75355173058240	1.97190458236700
N	1.21585685538882	2.70885384507198	0.55827166221971
H	1.32538137904189	4.05293242114670	1.87628108013953
H	1.85641962213559	2.65777592242405	-0.23255918738802
C	-2.01640620618560	3.95426383910601	-2.03842562914075
C	-2.12714521448407	2.56087409991707	-1.42324377511756
C	-0.77808541715260	2.06022972599109	-0.89968640622041
C	-0.14468465076688	3.04410444043234	0.09752139270435
C	-0.06591211640465	4.44366601754342	-0.53293791066210
C	-1.42189923647973	4.93512636824333	-1.03185429414688
H	-0.91308523008501	1.07346992059353	-0.43613889850838
H	-2.83955439479375	2.58060950592909	-0.58343372521589
H	-2.53273804216537	1.83774275271148	-2.14845399677111
H	-1.35959568669549	3.92386950891349	-2.92505223677822
H	-3.00064017202717	4.30003115143027	-2.38774178126488
H	0.35119290200320	5.13424965771681	0.21576651729233
H	0.64959577155110	4.39812541038222	-1.37252306995663
H	-2.10318463427006	5.03529002878159	-0.17194673872834
H	-1.31295791833791	5.93502219061644	-1.47695863610644
H	-0.06461416503679	1.93926156326670	-1.73551434211929
O	-0.99684291996422	3.08019532685603	1.23016367881379
H	-1.50583722123428	1.58957534082785	1.76241717700340
O	0.85710035145094	4.56209119121440	2.57839471602063
H	1.19121165966195	4.20638405601989	3.41085320783081
H	-0.59881502476731	3.70533932592869	1.87663616186166

96

TS17 + H2O

C	0.49114975600691	2.59978321109850	-4.50855866937472
C	-0.88221875864132	2.69161190629680	-4.67995108357483
C	-1.67021134649619	1.54199514665797	-4.61133329462136
C	-1.09395888582714	0.29654032659171	-4.38682335964195
C	0.29787630462859	0.19318945886659	-4.24175449014963
C	1.06831976101950	1.34930433779343	-4.30382942895550
F	1.23651620188111	3.68964799611800	-4.53112811641150
F	-1.44205211560454	3.87316149571362	-4.85955937671486
F	-2.97753529108688	1.68969701605944	-4.73616654683925
I	-2.37703275245713	-1.34091179021245	-4.17559082040068
F	2.38969425680181	1.29415182605490	-4.17351568772037
C	0.98223653325964	-1.14828799806133	-4.06601311110293
O	0.83698530991607	-2.03787550322383	-4.87425454243422
N	1.72441084347812	-1.24295564117607	-2.93702216852054
C	2.43336422076528	-2.44740326231793	-2.55965653292812
C	3.96057335905551	-2.24106046294601	-2.53336204668495
C	4.38313842837634	-1.16512039939834	-1.55997763677188
C	4.44556086777303	0.17578775701495	-1.96359122277724
C	4.77885169679674	1.18330846112152	-1.05627030844017
C	5.06475346510932	0.86177615683953	0.27134243243044
C	5.02014945248743	-0.47208615097200	0.68208511706113
C	4.68096353026330	-1.47629296081444	-0.22528038992210
C	1.97354361809117	-2.98228758784274	-1.19242453791847
O	2.62243849073074	-3.84203477485666	-0.61794104495925
N	0.87409132003863	-2.40541595515667	-0.67816816339298
H	1.82448248150997	-0.42302967821996	-2.34334190474428
H	2.18930077296524	-3.20599021601461	-3.31724011640509
H	4.41201068813236	-3.20249721559734	-2.25803794358146
H	4.28361261111250	-1.98166625168736	-3.55136981630432
H	4.23862545127733	0.43020609803860	-3.00483893942458
H	4.82347844807201	2.22097191435095	-1.39241211120687
H	5.33399714662887	1.64625319271111	0.98106297760874
H	5.25398146173533	-0.73444359115354	1.71572707178996

H	4.64084638665620	-2.51926159655525	0.09510218791352
H	0.29083776105400	-1.79402711694799	-1.24316795967067
C	0.44987810310612	-2.59124363324919	0.69056297978823
C	1.49479498777139	-2.03503725606843	1.67978216810015
C	-0.91783032946552	-1.90220936613268	0.82621017465401
O	-1.45481352510949	-1.36755727639676	-0.13688388791818
C	-2.71645541677433	-1.22676173131260	2.30832262107166
H	1.33128238248169	-2.49988718874650	2.66534713044238
H	0.31544874048581	-3.66765233048313	0.89125704084929
H	-3.47174229657572	-1.59314842795474	1.59587006239790
H	2.47339509981947	-2.39899597262856	1.33683676484979
N	-1.46443443026873	-1.90914350965219	2.05006767429803
H	-0.97286421958208	-2.36006775338411	2.81399042652011
C	-3.18755725842884	-1.51144818489953	3.73909179097891
H	-2.47327482442027	-1.05581450751726	4.44378830379564
C	-3.33268190075885	-2.98468039397447	4.03190341974423
C	-2.43980265379050	-3.64403525238968	4.88364412772718
C	-4.34811273588266	-3.73189846813116	3.41895221149942
C	-2.55309389999341	-5.01656905493750	5.11865860031712
H	-1.64906597775185	-3.07160226572304	5.37620816927043
C	-4.46621379936807	-5.10019275764481	3.65218003677614
H	-5.05416188947596	-3.22941544205899	2.75270299397457
C	-3.56673721538553	-5.74812176512179	4.50289512030960
H	-1.84777913101011	-5.51320835293949	5.78735598438458
H	-5.26520248630653	-5.66591869504079	3.16961073196838
H	-3.65927748738040	-6.81998629788701	4.68530697227909
H	-4.14865840476527	-0.99251401202332	3.86053674233908
C	-2.65739662223533	0.31094214139275	2.09909832068914
O	-3.74402381272359	0.86544259037363	1.93874784090946
O	-1.51654445422386	0.86317645764028	2.15146436920807
C	1.48617871234318	-0.51606027292632	1.85185459742350
H	2.36818897114201	-0.22895439963120	2.44979730913684
H	0.59341072144620	-0.23414183671054	2.43114324359842
C	1.45119607484075	0.33606576434978	0.57681542974360
H	2.36472346812726	0.20471361247555	-0.02636580016313
H	0.59688811268980	0.03871413949779	-0.04111074325444
C	1.28355758850164	1.78650045142377	1.01296116200124
H	0.52786503491083	1.81246781622171	1.81167990961497
H	2.23197418444735	2.15392910099460	1.42963148537386
N	0.87566780358432	2.78409017601072	0.01488705668291
H	1.25440676393839	4.14695003950776	3.07973827746257
H	1.37444312032202	3.66313468119375	0.13141164003228
C	-2.90935601418290	3.39160131237998	-1.83086445541045
C	-2.67710819264717	2.10533980017448	-1.04141138502035
C	-1.19615706336687	1.76180676465359	-0.85740820074696
C	-0.33973855007654	2.93023947979266	-0.47591167000381
C	-0.62722852524110	4.23696286032283	-1.16568042519430
C	-2.11784699495809	4.56405939423946	-1.25711745954028
H	-1.09688344667197	0.94340490002030	-0.13723367263873
H	-3.13720910244247	2.18389022637245	-0.04621332203821
H	-3.16201054597911	1.25434889610913	-1.54399206961068
H	-2.62815097566871	3.24909657688759	-2.88508096036194
H	-3.98215706921061	3.63330491286432	-1.83510927030321
H	-0.06573884031697	5.04212897259190	-0.66790065220578
H	-0.20792390051973	4.13415288654972	-2.18433625948688
H	-2.48562378789333	4.79841442793065	-0.24994976553705
H	-2.24478782885594	5.45794956615995	-1.88477519127928
H	-0.75718098234262	1.41467158427150	-1.81522031468597
O	-1.44263165106732	3.30433227636307	1.30910856266185
H	-1.55905845194171	2.36992420200106	1.70681833344569
O	0.92313457435028	4.57161395216294	2.27692763060019
H	0.77896029138656	5.49086670580449	2.53780135845773
H	-0.74365951614473	3.73729158865858	1.82885831481242

96

Intermedia T + H2O

C	-1.80476584167247	0.93081811461829	-3.37875429001072
C	-2.90236735003409	0.61504618362109	-2.59181324861672
C	-3.01407107586495	-0.66251526169582	-2.05147998214967
C	-2.04150048510150	-1.63070183145409	-2.28090228865069
C	-0.90609304682783	-1.31078051743598	-3.04903668604240
C	-0.81813865394848	-0.02669190261040	-3.58743625139878
F	-1.69085597233212	2.13588411142720	-3.90446555688091
F	-3.82056975315400	1.52816756856105	-2.34578113812417
F	-4.07564624753263	-0.90759525238407	-1.29531856957091
I	-2.36575814432254	-3.46050934533642	-1.30446674047713

F	0.23585268177434	0.34488975965969	-4.31063514189557
C	0.24291169025982	-2.29204336854541	-3.17891296672876
O	0.05662767421016	-3.48643074686005	-3.25245256946811
N	1.48836043728577	-1.74867298114249	-3.08159129514045
C	2.56812626074103	-2.55608039205171	-2.55213398475801
C	3.94196177791584	-1.91395194425844	-2.82783048346339
C	3.91826690018973	-0.41817548570357	-2.62968137713967
C	3.81384960903552	0.43252179612792	-3.73730261253452
C	3.67783328074829	1.81178694038746	-3.57069036462173
C	3.63412765482239	2.35714996450249	-2.28823143549164
C	3.73703294653293	1.51885289880003	-1.17557759385365
C	3.88490966874726	0.14243976934625	-1.34551185865435
C	2.35815659437559	-2.86829151749282	-1.05207066666905
O	2.92708951218656	-3.81017284879636	-0.53183597457491
N	1.50553347430981	-2.05173562723799	-0.39649055823461
H	1.58910238406516	-0.73968134120127	-3.01657658842073
H	2.53543833343967	-3.54435692943495	-3.02851878455293
H	4.67024936001170	-2.39989424306340	-2.16250373822229
H	4.22986983302128	-2.14151490732306	-3.86298006456983
H	3.82809657713382	0.00411446185565	-4.74249394063527
H	3.59818558589521	2.45926300405085	-4.44561071370961
H	3.51231580616240	3.43217976655927	-2.14739888478556
H	3.68530048831774	1.94057537100375	-0.17029128088982
H	3.96217554887685	-0.50925771879412	-0.47082684871605
H	1.07285122040311	-1.27124304734788	-0.88087196577892
C	0.90003647628292	-2.37966146892462	0.87463233008137
C	1.80356555282253	-2.15758489343861	2.11164921755243
C	-0.38420165609536	-1.54651723589869	0.93472828972385
O	-0.57012581081462	-0.61001349261976	0.16491410202016
C	-2.52212537259308	-1.09869620501281	1.91345216242494
H	1.50033086867015	-2.88512122286891	2.88049566765388
H	0.62254716991267	-3.44731882780775	0.84976740135273
H	-3.01850741631556	-1.14810318587220	0.93077003553171
H	2.82228190328316	-2.44185835372121	1.80630060138288
N	-1.29323776864770	-1.85884711094004	1.87382539882120
H	-1.17650163736524	-2.67727979764135	2.46195659541493
C	-3.46205087469939	-1.63988759687351	2.99778442329071
H	-2.97290952665280	-1.52326101423245	3.97673386399117
C	-3.82117254935830	-3.08491339136559	2.74980741980027
C	-3.27899532241951	-4.10840286955393	3.53478168025362
C	-4.66659320547702	-3.42661917056237	1.68634068214839
C	-3.56723461248560	-5.44727216982341	3.26001437001369
H	-2.62789277580780	-3.85339818305697	4.37538356876930
C	-4.95870410917071	-4.76110144245554	1.41119573965492
H	-5.09099797819784	-2.63646662320078	1.06083652015079
C	-4.40612681001811	-5.77634535970363	2.19617675085423
H	-3.13636177774063	-6.23381077029992	3.88173671291126
H	-5.61911496077060	-5.01069777121313	0.57864879221138
H	-4.63396765778102	-6.82133896201458	1.98025888596476
H	-4.36552456941642	-1.01265926955831	2.99633171194479
C	-2.21672817551901	0.37114495888081	2.19381660947117
O	-2.96634307651063	1.19849503643303	1.50835837615359
O	-1.39558132361287	0.71298188409870	3.02074634792833
C	1.76378014084399	-0.75951857976451	2.72716571660644
H	2.38632964125182	-0.76154323047673	3.63687100655720
H	0.73480013957730	-0.55297708523546	3.06659831794544
C	2.20579201791373	0.38637846335954	1.81795960710217
H	3.29843034534764	0.36028334861508	1.66356487899412
H	1.74276831886416	0.28548317081367	0.82471232462813
C	1.78740553049720	1.73167359469715	2.42095751822901
H	0.71252581602852	1.67894537132801	2.64979965837516
H	2.29599872973243	1.86860527287461	3.38808284518201
N	2.06051073175037	2.90203784913779	1.61572758250470
H	-1.04882365908649	6.08305235284540	1.12552956026820
H	2.95189262907538	3.36002552707661	1.75889666448972
C	0.00719898861163	3.78903389349225	-1.97602541130493
C	-0.81621101334403	3.24497542376597	-0.81344405885881
C	0.08004013835891	2.53767340917050	0.19962854667796
C	1.33165523055983	3.32069107900889	0.52258377177696
C	1.69679746297632	4.40239438883775	-0.21211110047309
C	0.98525248952526	4.84614964990877	-1.46649342846668
H	-0.48268516125246	2.36093801935177	1.12770529896961
H	-1.33263489995020	4.08060986820927	-0.31337049826165
H	-1.59923848268444	2.55346160220701	-1.15767748672283
H	0.56683405304441	2.95734090219597	-2.44131689512953
H	-0.64289256016862	4.21230668162428	-2.75463725383622

H	0.16939871593967	5.15047085258265	1.20819858039402
H	2.60136302432985	4.94653310851527	0.08215380038629
H	0.43625395615001	5.79241197968253	-1.29285603731189
H	1.72529250630371	5.08313386147264	-2.24942865511020
H	0.36010322543738	1.53854556634200	-0.17201781999936
O	-0.62707038058500	5.43086107606461	1.69906803240881
H	-1.66559454763041	4.13381376483997	2.22881066513020
H	-1.61688593455869	2.88885267614058	3.16051345909035
O	-2.21185538039541	3.39681814534950	2.58731166192738
H	-2.74672539541643	2.14615568033267	1.80423401389850

96

TS18 + H2O

C	-1.92659190363716	0.83927645106100	-3.43931879741797
C	-3.02410418896875	0.48749169814216	-2.66794942393521
C	-3.09839642653728	-0.79261249110761	-2.12675732902602
C	-2.08808463109742	-1.72579278516811	-2.33827762333552
C	-0.95354500342659	-1.36757089992427	-3.09028334375791
C	-0.90570028788106	-0.08384102491191	-3.63389046318967
F	-1.83911843125266	2.05222102697263	-3.95559917005010
F	-3.97332521023515	1.37056782054348	-2.43381543446086
F	-4.15972347506561	-1.07345532432690	-1.38513808996816
I	-2.35417285864986	-3.55763895544161	-1.35060798797523
F	0.14434637728464	0.32516963053431	-4.34558870736146
C	0.23722329826954	-2.29954207652593	-3.19507793566542
O	0.10818593586754	-3.50142818257803	-3.26762721814152
N	1.45591661455523	-1.69877217333244	-3.09003053226512
C	2.58451389577963	-2.46209183296917	-2.60080754635879
C	3.91187782459612	-1.73973837905599	-2.89153317176789
C	3.87608592920059	-0.27790758578517	-2.51662949842505
C	3.50384706893570	0.68550006193435	-3.46567325107863
C	3.40372060407371	2.03330561245246	-3.11747699197697
C	3.68639618768272	2.43866733670389	-1.81250341171377
C	4.06228265805331	1.48859262777626	-0.86153737522320
C	4.14909876251508	0.14016849526808	-1.20841892339348
C	2.42397441898243	-2.82271595346604	-1.10321519515369
O	3.03406367070549	-3.75994849141499	-0.62315249102115
N	1.56641229377352	-2.03959382684444	-0.41641406352308
H	1.50379741713999	-0.68795154937783	-3.00224412102783
H	2.59464113981551	-3.43670437322173	-3.10581840228731
H	4.70000568596312	-2.26954292265742	-2.33816098447491
H	4.12700070023210	-1.83979188049892	-3.96444903906128
H	3.28732513904640	0.37135688590875	-4.49017698100991
H	3.11128396726575	2.76877785624195	-3.86940442295859
H	3.61896442917386	3.49289690676710	-1.53755792622259
H	4.28609830542934	1.80088719564738	0.15943576924407
H	4.43817200655273	-0.60146552805342	-0.45842495284041
H	1.08847591804156	-1.28623516290194	-0.90270715815561
C	0.91561832381072	-2.40513796013718	0.82353197423722
C	1.77237808252665	-2.21943951278170	2.09656979097517
C	-0.37473786305464	-1.56665445259696	0.84266345818136
O	-0.55009017536734	-0.67700806221335	0.01367027184063
C	-2.49053491788867	-1.01861819208118	1.83087939239872
H	1.41980591027331	-2.94365114006817	2.84745760300274
H	0.63328144432941	-3.47036780368116	0.76074560381251
H	-3.00044416418248	-1.08113463824254	0.85627125287566
H	2.79478360173470	-2.52925266796928	1.83027824843974
N	-1.28622483295724	-1.82718312576920	1.78666676343655
H	-1.14621827036927	-2.58346898932968	2.44799084568913
C	-3.42176672707057	-1.51388711706267	2.93967751791950
H	-2.91553090201664	-1.37715693152911	3.90765480569263
C	-3.82110117620901	-2.95692502578936	2.74794996711493
C	-3.32548912246630	-3.96244286934337	3.58533563861816
C	-4.65984798556461	-3.32102672285351	1.68594288614490
C	-3.65327156184024	-5.30264580545981	3.36647190306501
H	-2.67840609265463	-3.69041940635128	4.42365296168781
C	-4.99183044796542	-4.65633287483934	1.46588565637287
H	-5.04658370152625	-2.54690576692327	1.01756752709072
C	-4.48640178938701	-5.65288619478412	2.30496450962180
H	-3.25770802678907	-6.07368351619088	4.02991612760437
H	-5.64653504983016	-4.92185277489189	0.63359659855520
H	-4.74569235660994	-6.69869236050583	2.13237558845998
H	-4.30953826799837	-0.86430742285760	2.93296038841844
C	-2.14373818441307	0.46799632440809	2.06793500808730
O	-2.80936375063696	1.30681434855719	1.40492490787210
O	-1.26929248136595	0.72536873451793	2.91712852919944

C	1.74412170046482	-0.82483542299799	2.72111907493608
H	2.35417109786763	-0.83707943215522	3.63917901110530
H	0.71253457397018	-0.59559682879023	3.03448160555477
C	2.22002322656087	0.30487814888593	1.80891072900276
H	3.30922725558596	0.24756856015096	1.65182745342604
H	1.75398747379929	0.20480476011412	0.81865661559113
C	1.82647880825138	1.65682840546865	2.40174204608772
H	0.76225267750728	1.61662065143494	2.67705693854685
H	2.39341670381136	1.84658834864382	3.32390411981558
N	2.05126091308599	2.80468779634956	1.52489534645956
H	-1.18461705030922	5.86161572043237	1.05174143941891
H	2.81776278255254	3.42609278953244	1.76300532174583
C	-0.07839018203567	3.65706024046525	-1.98597926892040
C	-0.82540249371871	2.96454604330445	-0.85038530093201
C	0.17346958410540	2.26682962114471	0.06397635613591
C	1.27476549097936	3.18318571068236	0.51158084441636
C	1.36855348218013	4.46842656328997	-0.04671976276191
C	0.82480912464943	4.77047157177156	-1.43906510491375
H	-0.32433469676657	1.82295562786721	0.93765853703628
H	-1.40691912836488	3.69894932871802	-0.27119573284531
H	-1.54685374668387	2.22492762851578	-1.22083628107006
H	0.53106960138113	2.89947765004992	-2.50952224068345
H	-0.77514592243483	4.06772757667991	-2.72895352350821
H	0.34409845628745	4.90276690562775	0.81522029295713
H	2.22994611145990	5.06685236772603	0.27698159078781
H	0.26011224764473	5.71801768206139	-1.40592372663820
H	1.66091822575846	4.94816487113027	-2.13529804706397
H	0.63976504646845	1.42327024376525	-0.47347935540587
O	-0.57439059222573	5.29616521391521	1.54541625616115
H	-1.13912001771056	4.44811051240389	1.91975158544969
H	-1.33445477789629	2.80473301618658	3.02385760199926
O	-1.82788932589382	3.38850603150373	2.42100481485052
H	-2.32169399702645	2.65704581650080	1.88155723182416

96

Intermedia U + H2O

C	-1.65831525609706	0.84080224414463	-4.06830937473755
C	-2.75721755130732	0.74226462966869	-3.22773022114463
C	-2.92714761835533	-0.39651316700701	-2.44240944609075
C	-2.00960637260362	-1.44301677446536	-2.48352676764277
C	-0.86112758428911	-1.32090666097769	-3.28618953834029
C	-0.71784645849222	-0.18312564333100	-4.07855220666168
F	-1.48220059966262	1.92137712353724	-4.80864326041953
F	-3.61964896057758	1.73819795893768	-3.15458047301538
F	-3.99054123036426	-0.43509569509920	-1.65596499831095
I	-2.45571707464884	-3.09229763766576	-1.26648985914607
F	0.35460626017740	-0.01786393738697	-4.84998036543826
C	0.26564757784320	-2.33007970324423	-3.20010479120844
O	0.07568619848621	-3.52518136891066	-3.17900051941755
N	1.49642238587551	-1.76799260690986	-3.04633110746957
C	2.59077360386363	-2.53952246527641	-2.50166189601833
C	3.94889212541913	-1.89328291851068	-2.82385698523282
C	3.99848175539760	-0.42346073226625	-2.48205624338562
C	3.63795208499326	0.53693408932403	-3.43927602095614
C	3.63334982460774	1.89618026434208	-3.12495776733934
C	4.00553162519448	2.31772874564521	-1.84731466885794
C	4.37405347795243	1.37161596353510	-0.89012328517735
C	4.36060799120236	0.01090267416692	-1.20111011772465
C	2.42654849150239	-2.79947094919712	-0.98736246195938
O	3.06536795064475	-3.68222061706112	-0.44344126959865
N	1.54525471933665	-2.00056434379235	-0.35336056381250
H	1.57683659321052	-0.75532671390515	-3.03244292928681
H	2.56255712868618	-3.54268723634137	-2.94795083299092
H	4.71252482352212	-2.45088607145291	-2.26405056428072
H	4.14709001103409	-2.03010526944502	-3.89599603556563
H	3.36109755525208	0.21226731067803	-4.44591753603744
H	3.35424758839691	2.62776033038984	-3.88553227001883
H	4.02534857630293	3.38278073687153	-1.60653142569505
H	4.68599968525795	1.69472532096797	0.10528695466715
H	4.64702530297988	-0.72733115422689	-0.44660462744210
H	1.03060267954188	-1.30005940731469	-0.87933945231720
C	0.96213273941578	-2.29848334947919	0.93710130845462
C	1.90086792300252	-2.05882687586203	2.14365051159869
C	-0.32423332716776	-1.45769710552616	0.99574987699589
O	-0.53987108168623	-0.58799542087018	0.15173810502088
C	-2.38757913258118	-0.91882655645880	2.11836567449368

H	1.62531452743911	-2.77302507584418	2.93479336705065
H	0.67274839049213	-3.36356324930844	0.94358026207349
H	-2.86475397802433	-0.85236991992899	1.12824075154684
H	2.91001980514640	-2.34741417395598	1.81249657847504
N	-1.18461473721799	-1.71698285037167	1.98281131286838
H	-0.95895631041017	-2.42241124899040	2.67568407881606
C	-3.35782486309376	-1.57583215023515	3.10649704146214
H	-2.88070376337952	-1.61454207003764	4.09901095315229
C	-3.77100457250353	-2.95683630976546	2.66171509016486
C	-3.16722583988037	-4.10213491321790	3.19392615623690
C	-4.72366863942857	-3.11348527040774	1.64703336504817
C	-3.49133636104735	-5.37257416018299	2.71408003609069
H	-2.43576744872409	-3.99790512414544	4.00037227295804
C	-5.05603761637795	-4.38026931576662	1.16851965505686
H	-5.19997534401155	-2.22705735846674	1.21938137335480
C	-4.43538460496611	-5.51480356310499	1.69692216208463
H	-3.00782740813250	-6.25373423461933	3.13947958264324
H	-5.80010438136113	-4.48266768604077	0.37627489231004
H	-4.69277694364482	-6.50643393719863	1.32123424567647
H	-4.23140606568946	-0.91345103835391	3.18133563873536
C	-2.08632648332963	0.53408746334934	2.56597484412366
O	-2.95186895724921	1.37690688143225	2.23891929028705
O	-1.05097101281576	0.73533806848612	3.23536209510043
C	1.87257962915827	-0.65118443158268	2.73728250798157
H	2.56660808889216	-0.61344968638798	3.59245519272460
H	0.86604436497742	-0.44893261873017	3.13551861723692
C	2.21979891702877	0.46280951857459	1.75025605021878
H	3.29122181811345	0.44177872224828	1.49926142670048
H	1.67212159869335	0.31754631492768	0.80687030028276
C	1.82178423797139	1.82420987422001	2.32117858800643
H	0.78982349349347	1.76953475086187	2.69583220204484
H	2.47831379699927	2.12081260873586	3.14994027899792
N	1.88834227631344	2.88644830166763	1.31281032685874
H	-1.54875453730777	5.86969004253344	1.68400308791980
H	2.77554142255901	3.37607445445865	1.20152586564386
C	-0.57457104483058	2.97367022565111	-1.84178352289138
C	-1.33895218107701	2.98732735456010	-0.51935836302226
C	-0.40589287127443	2.55372627227680	0.60526733476891
C	0.91676009483380	3.22707271548709	0.52910406615350
C	1.07942000525216	4.34471137486243	-0.43542318072408
C	0.55957321746705	4.01344842809316	-1.85056774027480
H	-0.83858943284994	2.76542680199809	1.59621113705852
H	-1.72608549747354	3.99613616546508	-0.30484941503319
H	-2.19821013116990	2.30201305487406	-0.54043151069155
H	-0.14727381869903	1.96299695599662	-1.97262377485395
H	-1.24577329943332	3.15038679316467	-2.69335561772539
H	0.44165133926205	5.11900077404096	0.02687658851878
H	2.11191809593268	4.72023335036307	-0.44625463219553
H	0.20857570096533	4.95163666664173	-2.30137925853473
H	1.39216257616766	3.65358688601430	-2.47062690031904
H	-0.25878607241062	1.46183832481104	0.58134643374600
O	-0.78398555508185	5.32472766418838	1.90232770952447
H	-1.14794588449284	4.66708607482161	2.54040583425811
H	-1.24418335907875	2.73357327817635	3.84316670693821
O	-1.85314064805913	3.41858043311324	3.51947604300991
H	-2.45328615780386	2.81962806967017	3.00016274214355

Pathway 2

93

Intermedia O*

C	3.08802844882731	-3.73687754594245	2.29231687234947
C	2.03133814466144	-4.16994630956788	3.07865528471443
C	1.24903755695228	-3.24024764389735	3.76320335123979
C	1.51943612135597	-1.88050915290603	3.68327731638638
C	2.60372679377033	-1.43394148474959	2.91044996068973
C	3.36609434330663	-2.37253613113788	2.22262139199281
F	3.81148177138017	-4.60574156527409	1.61138962241694
F	1.72214642863017	-5.45138120172501	3.12594525947169
F	0.20852094723985	-3.70310627652969	4.43585608253334
I	0.18627226904563	-0.57932910130031	4.63769667131746
F	4.40012336419367	-1.99224372596837	1.47798889301325
C	2.98646791857489	0.03252407449477	2.86230085520190
O	3.16292973153832	0.66637212433425	3.87855814203714

N	3.07371101612961	0.54928365662715	1.61110673105521
C	3.40432195311174	1.93568983476222	1.35743269104673
C	4.65381937855797	2.07867458661518	0.46929449559065
C	4.50421044536326	1.40773102080670	-0.87733842853421
C	4.84659038552999	0.05865271967033	-1.04684291169735
C	4.69951276596307	-0.56825720669142	-2.28611203674114
C	4.21035324443899	0.14817499152380	-3.37903482615155
C	3.86525508623191	1.49222851999332	-3.22273235202567
C	4.01001003506301	2.11560332911725	-1.98292517335649
C	2.23817808539575	2.70843704570945	0.71383757042466
O	2.42968760461283	3.79554513915982	0.19499151268689
N	1.03895924655545	2.09568819716710	0.72850092415287
H	2.97974543110428	-0.07121092272961	0.81082134196694
H	3.60648193248815	2.39543446798069	2.33561185821973
H	4.83182147169723	3.15302303580192	0.33661741896855
H	5.50657800202273	1.64879865696778	1.01312779703399
H	5.25340267141031	-0.50127536702523	-0.20187214262234
H	4.98144718041353	-1.61664593917052	-2.39930363095361
H	4.10422939516841	-0.33682402722530	-4.35085522852200
H	3.48764655581764	2.06232246411059	-4.07369247573858
H	3.74594010848662	3.16797888651192	-1.86021605863195
H	0.92068059096794	1.20969927824562	1.20960473949237
C	-0.06634401254266	2.54052286896813	-0.09428481718475
C	0.24085224008771	2.39877644603519	-1.60915055392337
C	-1.27917815934765	1.68420746550586	0.26828128423861
O	-1.13133823838276	0.55106607754778	0.73058978996098
C	-3.68277083040947	1.38290336040780	0.11795319507194
H	-0.42823770116840	3.08393750197000	-2.15172903855367
H	-0.27380662196049	3.60011707855533	0.12067052671970
H	-3.83278148082258	1.11591684864318	1.17513449199824
H	1.26283817905758	2.77508698090614	-1.76120983226414
N	-2.48157010619677	2.19258996448658	-0.01319766975989
H	-2.55316540033162	3.14027275541362	-0.37040957922094
C	-4.89328922787837	2.15405734406183	-0.41663631058455
H	-4.74987389678960	2.30646854023123	-1.49727921154885
C	-5.10333632959330	3.47774115563300	0.27758257840790
C	-4.84652207004358	4.68449225103016	-0.38247637598237
C	-5.52380644773560	3.52030642470905	1.61415003973936
C	-5.00129957141004	5.90741800195510	0.27523474872465
H	-4.53066097178498	4.66542114091583	-1.42908033113165
C	-5.68118315491787	4.73775996069461	2.27248960537550
H	-5.73306899261030	2.58527831752076	2.14035434458894
C	-5.41811047879051	5.93655521694978	1.60454206583651
H	-4.79880874001566	6.83886659491415	-0.25624311168629
H	-6.01340649533932	4.75352124178643	3.31200263761895
H	-5.54193250088678	6.89002853897951	2.12041552737433
H	-5.77282733985473	1.50628254204041	-0.28608583857255
C	-3.53061617820493	0.06575942259765	-0.67546082287380
O	-3.94602571594615	-0.97733103250704	-0.12058032221558
O	-3.00964961806446	0.14719278113477	-1.80689812946285
C	0.05943713324967	0.99413489725094	-2.19464285181092
H	0.30903667396697	1.03293167634677	-3.26747218030094
H	-1.01402784328041	0.76128427348355	-2.14521052022175
C	0.88308898927985	-0.11867988888883	-1.53430758764365
H	1.92164169447967	-0.09465634711143	-1.90089820981737
H	0.93471952927856	0.03244345343579	-0.45055500934695
C	0.34903488422552	-1.53196273030773	-1.74398944382694
H	0.34646076553023	-1.83385187710177	-2.80003299379178
H	0.98415921814109	-2.22665219384451	-1.18613301339820
N	-1.04090642005092	-1.66749568686205	-1.22427457567356
H	-1.10250983897417	-1.16153240644482	-0.32236266071171
H	-1.71241459779910	-1.11353106935289	-1.81005219258172
C	0.13041288118972	-5.01859206358993	0.23190796249301
C	0.23466998027126	-4.76036966772126	-1.27342643676376
C	-1.04794257332444	-4.15935845429692	-1.86310045624420
C	-1.74059651748638	-3.05554886266672	-1.05787729215088
C	-1.76137643264144	-3.37936289138738	0.43657940940675
C	-0.40540424190365	-3.80277740870435	0.98968171487717
H	-0.90256544930784	-3.81967863260717	-2.89948008924126
H	1.10741238572319	-4.12433148895169	-1.47695396138311
H	0.43748460968983	-5.70673890924847	-1.79607040294992
H	-0.55381212988140	-5.86470911653232	0.41203881939160
H	1.11266641030801	-5.32100399576360	0.62613294355124
H	-2.18999284875221	-2.52047041747246	0.97768893411474
H	-2.47402942868672	-4.21145668566779	0.54653096911938
H	0.31571924699719	-2.96416679945413	0.93741162322723

H	-0.52141903809594	-4.04595625201059	2.05580296869907
H	-1.81735823758191	-4.94483859832135	-1.90803857490818
O	-3.00335670760779	-2.90235266993677	-1.56702631523194
H	-3.49797141790402	-2.21532570309540	-1.01460524799399

93

TS11-1*

C	3.06075002212155	-3.65991967195663	2.26535949911906
C	1.97874971837477	-4.07862327943484	3.02462659868579
C	1.20761188571819	-3.14132040268032	3.71150369837554
C	1.51018566860125	-1.78726953039441	3.65483421153677
C	2.61662744892808	-1.35556023054378	2.90615961187472
C	3.37197751125727	-2.30154371435563	2.22146059913006
F	3.77788289369519	-4.53631737395207	1.58759114019407
F	1.64168016108717	-5.35364648311424	3.05325070672747
F	0.15145984781196	-3.59067045662532	4.36916221361792
I	0.20262823500597	-0.46796019651940	4.61992600993209
F	4.42496066862161	-1.93239983416682	1.49856761649201
C	3.01886375198104	0.10606136510433	2.86738434582368
O	3.21743935205399	0.72928024359794	3.88621265304267
N	3.08639357584953	0.63271045459150	1.61947906087564
C	3.39641474411683	2.02470121892015	1.37071532988042
C	4.67042812744250	2.19250465379130	0.52306394913258
C	4.57501007890663	1.50554403568869	-0.82003547195019
C	4.95267709725478	0.16290120212149	-0.96239038736081
C	4.84353681491220	-0.48581860568489	-2.19412611783741
C	4.35864383497532	0.20265570467030	-3.30665885442542
C	3.98291584526458	1.54132072148993	-3.17847006233799
C	4.08811681917569	2.18620651751106	-1.94563624764542
C	2.23178703076594	2.76424739502597	0.68753625652645
O	2.41138330247370	3.85009464825286	0.16170324623218
N	1.04638788008678	2.12511923385189	0.68084004784675
H	2.98328472859579	0.01819861485814	0.81542099652845
H	3.55498125474258	2.49223060057709	2.35332501160527
H	4.82939934327943	3.26951902149124	0.38730242997162
H	5.51401874403557	1.78497653345746	1.09751931816490
H	5.35319406662508	-0.37486300424437	-0.10018853114722
H	5.14929802950216	-1.52964929704699	-2.28525627669075
H	4.27959676033813	-0.30003741312455	-4.27203729780868
H	3.60897536980066	2.08964460842548	-4.04522636950893
H	3.79494333446354	3.23300277774023	-1.84336673273164
H	0.93376139402496	1.24372235692696	1.17140968318127
C	-0.05165656203188	2.54665600552364	-0.16185429357491
C	0.27370971796103	2.38076823636056	-1.66941425189049
C	-1.26460616227142	1.69445695872317	0.20635977726239
O	-1.13008859334803	0.58326256569551	0.71365405674214
C	-3.65609242973127	1.36762021763877	0.04766623216586
H	-0.41257974192405	3.03094300749089	-2.23381533537750
H	-0.26668000209091	3.60899784976352	0.03231323981182
H	-3.75161400861303	1.06995105765784	1.10260037761656
H	1.28410796595394	2.78934380698601	-1.81857018939962
N	-2.46960881878283	2.18571742749223	-0.11894102671267
H	-2.54863014594162	3.11170853265000	-0.52659012121809
C	-4.90229022635889	2.13428683642284	-0.40834307138706
H	-4.81704915897159	2.31517986644811	-1.49077029524629
C	-5.08653894192019	3.43925199904078	0.32795291021076
C	-4.91693582204460	4.66307400978300	-0.32793382055024
C	-5.40025235854434	3.44580652218040	1.69390470634892
C	-5.05429800020116	5.86951947617993	0.36295337594051
H	-4.68287414745329	4.67088679104791	-1.39576109578307
C	-5.53920063145619	4.64701213226894	2.38551269141121
H	-5.54053017202209	2.49685841700928	2.21804677604252
C	-5.36507198647777	5.86388730215911	1.72134719581339
H	-4.92052113869937	6.81522610706962	-0.16494334580367
H	-5.78737285577404	4.63554920063513	3.44820795420956
H	-5.47455658558691	6.80449437843984	2.26336107537276
H	-5.76898657595756	1.47546735504854	-0.24949641799713
C	-3.51809254080418	0.08868777425027	-0.78900540477483
O	-3.94840880085255	-0.98339333133299	-0.20863994814876
O	-3.04690409431698	0.14458588281766	-1.91275466248475
C	0.15080429303388	0.95779579252482	-2.22478973033963
H	0.40904286870423	0.98538356422012	-3.29640859866506
H	-0.91316091574454	0.68109960902697	-2.18117119863802
C	0.99906589371201	-0.11947144565219	-1.53988267199432
H	2.05667604552210	-0.02747024920331	-1.83767212979770
H	0.96492066757457	0.01175489889592	-0.45127165677828

C	0.51965020140281	-1.55002964678074	-1.79765454781993
H	0.55797141279756	-1.81427892860015	-2.86550945802672
H	1.19062993671213	-2.23613512615639	-1.26350936755732
N	-0.86047670547875	-1.75453007683185	-1.31957288812171
H	-0.97902780810324	-1.23723317712063	-0.43908649846653
H	-1.52057533790790	-1.30273379636346	-1.96101463038792
C	-0.00346700726439	-5.09227325491519	0.19942234111369
C	0.00295781182635	-4.87973574664123	-1.31521912235462
C	-1.35289457928937	-4.40794347423955	-1.84111953212367
C	-2.02545813523152	-3.31192522351022	-1.03587967540574
C	-1.88557869642757	-3.43720541539284	0.47224492804036
C	-0.49691721937795	-3.85069191774246	0.94357846620977
H	-1.30742705447112	-4.08980615441148	-2.89280184141958
H	0.78123841753607	-4.14958302234943	-1.57602632313294
H	0.28101142837382	-5.81389141334208	-1.82514003965449
H	-0.66617737857317	-5.93896895167774	0.44843583113378
H	1.00433962364942	-5.36935777574893	0.54567628716908
H	-2.23331705782783	-2.50709435764489	0.94674829244596
H	-2.61355274203442	-4.22049461061262	0.74863479771062
H	0.21326547683140	-3.01595549390715	0.79621122627051
H	-0.53488048322842	-4.04829243926873	2.02452538804797
H	-2.07601909408645	-5.24179689827855	-1.79993714908056
O	-3.12854839547131	-2.87761191670950	-1.49359243018932
H	-3.66333963496057	-1.87817443707184	-0.80041360762649

93

Intermedia O1*

C	3.03065674653081	-3.63165622768746	2.14397605981864
C	1.95269686142243	-4.05066893538970	2.90899402043972
C	1.20295833875384	-3.11716782701573	3.62425140059926
C	1.52615769077402	-1.76707561428447	3.59389093828215
C	2.63127861998119	-1.33644725626612	2.84272748112374
C	3.36286319548169	-2.27767835080511	2.12662049382163
F	3.72853845333521	-4.50446372260097	1.44203639453520
F	1.60449277779640	-5.32339352666087	2.92527347224690
F	0.15240269581528	-3.56627163569322	4.29210977301758
I	0.25742770387132	-0.45038641907034	4.61287817005798
F	4.41403268992824	-1.90857463567672	1.40163450014945
C	3.06129409195586	0.11751162300953	2.84050443103681
O	3.28225035885403	0.70610253534482	3.87570135992456
N	3.12736599163419	0.67996649139393	1.60921374642174
C	3.43351893675444	2.08022635837878	1.40484887041666
C	4.71278412203036	2.28324645438961	0.57368000288947
C	4.63590417299221	1.63502262464513	-0.78936245398606
C	5.01110555144781	0.29541025305881	-0.96288125411526
C	4.91310059705479	-0.31991371884225	-2.21234109806945
C	4.44285445015673	0.40011773417670	-3.31121157309337
C	4.07178139634467	1.73670750338367	-3.15205164984554
C	4.16456660210751	2.34791474515304	-1.90127593403879
C	2.26479927303586	2.83071658597483	0.74061842854108
O	2.43901106135639	3.92724635586641	0.23530651817307
N	1.08096883832601	2.18930721146282	0.73314753333526
H	2.98952872425888	0.09796962970917	0.78582717975663
H	3.58314742550731	2.51731009057053	2.40278243336234
H	4.86397425779935	3.36512281092288	0.47137140956067
H	5.55426210986164	1.86588423570539	1.14428215312396
H	5.39638296804779	-0.26742060060416	-0.10966341806809
H	5.21333005949471	-1.36293755938737	-2.32764490164436
H	4.37080406221808	-0.07701000166801	-4.29000570117840
H	3.70887811049759	2.30882108334160	-4.00800470966531
H	3.87075858018822	3.39166362566188	-1.77310531798794
H	0.97794640967027	1.29338932048557	1.19881762210209
C	-0.02293280154532	2.61114808820663	-0.09944638174071
C	0.30202684610653	2.48292372214734	-1.60812801078150
C	-1.22057082019047	1.72815153281966	0.24667465988350
O	-1.08256116558225	0.63493379721215	0.78276711482567
C	-3.60346619653544	1.37027466408240	0.06493368449795
H	-0.41117469882437	3.11411004263925	-2.16134637881744
H	-0.26142556835364	3.66375951721855	0.12076629859581
H	-3.69982815616071	1.11229301812404	1.13075170503375
H	1.29608781095570	2.93095967318189	-1.75654497248343
N	-2.42722473711107	2.18654859162425	-0.13431259764670
H	-2.51744089444519	3.10945231634699	-0.54596367911890
C	-4.86105618913655	2.10758150963551	-0.41421284388449
H	-4.77882489882522	2.26300729545632	-1.50083663291817
C	-5.05839904951960	3.42772070739975	0.29110563678340

C	-4.88607720427585	4.63820158655009	-0.38828486229312
C	-5.38666619950399	3.46063104337363	1.65312911570758
C	-5.03461088081846	5.85779004049331	0.27670216343751
H	-4.64078677718822	4.62547668539998	-1.45355607762992
C	-5.53664774618510	4.67504796553427	2.31875263236424
H	-5.52954891456569	2.52208255671782	2.19505969629839
C	-5.35940578887956	5.87863845307785	1.63167264519582
H	-4.89830402987947	6.79305943059498	-0.26879887156633
H	-5.79585817663934	4.68430662371107	3.37880014828530
H	-5.47734287623602	6.82970954344712	2.15323303202302
H	-5.72002023352752	1.44367487340372	-0.23693253412633
C	-3.47130984503080	0.05987618383952	-0.71219427618994
O	-4.01963609980689	-0.95678585021631	-0.08715259290652
O	-2.95679004825256	0.00372899053083	-1.80419603545604
C	0.23642935229193	1.06170181833558	-2.17190849418497
H	0.48027863953882	1.10961017660621	-3.24692176196261
H	-0.80780120986013	0.71711922647439	-2.11311108979049
C	1.14094428239541	0.01583187715198	-1.51633257040333
H	2.20092303147549	0.23260158419700	-1.73087593253369
H	1.01815025791572	0.04541629534111	-0.42434122053108
C	0.78509519272673	-1.41095141971698	-1.93853792427466
H	0.86688393164670	-1.52393942289975	-3.03214304927607
H	1.52621631476429	-2.10448808303118	-1.50210445319483
N	-0.55503722965815	-1.82357448858282	-1.52655470184089
H	-0.76691504528809	-1.40074630567344	-0.61810773216587
H	-1.26673756807608	-1.43749214102453	-2.14711007414355
C	-0.14446670150464	-5.25165017782190	0.21253910723392
C	-0.28972193960591	-5.10943132198899	-1.30114057700292
C	-1.74183663295107	-4.83822217063441	-1.69850256662431
C	-2.40648335867446	-3.74133181826418	-0.90768086907802
C	-2.07275284439939	-3.63859563015040	0.56108244422005
C	-0.63225227979111	-3.99522930416577	0.93260353083467
H	-1.85835348272106	-4.60656669854075	-2.76544287948803
H	0.33015866346060	-4.26955432423431	-1.64661667858111
H	0.06278463466096	-6.01853728348683	-1.80931016008856
H	-0.72493806079099	-6.12371190669380	0.56194564122646
H	0.90589960446041	-5.44741311731720	0.47678756789759
H	-2.36579473976423	-2.64309285913675	0.92505604027060
H	-2.75736353399974	-4.36334158543752	1.04085634362872
H	0.02883564556328	-3.15071899201648	0.67744373370817
H	-0.58254405051655	-4.13653678970069	2.02133004069946
H	-2.35191610547126	-5.73830860736132	-1.49315074107698
O	-3.30527051848483	-3.07438065342194	-1.40091337950235
H	-3.84288661982719	-1.81160966969018	-0.60076092187581

93

TS11-2*

C	1.88035105252148	-2.55348328944615	2.96105083789208
C	0.54900333053655	-2.75622559975225	3.28727932924869
C	-0.31162008311923	-1.66377381380606	3.39010541356772
C	0.14292174874666	-0.35956834803847	3.20916104518414
C	1.49502847000186	-0.14462037681925	2.87034763432707
C	2.33696252946074	-1.25295817487056	2.76526371928150
F	2.70624319603643	-3.57759192672296	2.85500115733494
F	0.09797435276620	-3.98032237656834	3.48620139890360
F	-1.57708403076931	-1.92912421940303	3.67367623760135
I	-1.30984985857037	1.14438761293568	3.45383410872815
F	3.62704545898732	-1.11380556410956	2.46482564999935
C	2.03984054228419	1.24857536386780	2.61577261529418
O	1.68035297182570	2.20400473557261	3.26761073113519
N	2.90865190097502	1.34457813985071	1.57246931839260
C	3.28274498447569	2.62871101843934	1.01996164880903
C	4.67870862417792	2.58540836316952	0.37998726230706
C	4.75699138582482	1.59518801400144	-0.75713190923409
C	5.15404350176918	0.27064684522370	-0.53309528714632
C	5.18095991583539	-0.65378398358321	-1.58039398207952
C	4.81811947585249	-0.26110017467053	-2.86793550966081
C	4.42088989609958	1.05724861962970	-3.10238868867324
C	4.38433398553188	1.97547156555286	-2.05489480967322
C	2.25778538398887	3.14697576125886	-0.00973842994928
O	2.36215686566041	4.26182521758712	-0.48942628731890
N	1.29725821538042	2.26235616701652	-0.33191379573432
H	3.16271442224143	0.51184263030800	1.04868866792030
H	3.27942455646378	3.36194443786932	1.83802023344065
H	4.89386497518133	3.59891075178696	0.01592754017748
H	5.41110383475352	2.33616895955046	1.16051087949350

H	5.45805149317273	-0.03751732964544	0.47140020264888
H	5.49773544447231	-1.68056168479910	-1.38900942981382
H	4.84599913503775	-0.97993919723327	-3.68873191230790
H	4.13574659264390	1.37141824034130	-4.10825077687600
H	4.06861101388511	3.00603436480091	-2.23603846902579
H	1.25072208200398	1.38265604142471	0.16993520497786
C	0.10056205283365	2.56475190342332	-1.07388651345566
C	0.14903052186492	2.17811548074611	-2.57266866763509
C	-1.00003389411360	1.74952979131425	-0.39435012919522
O	-0.71236560019248	0.74752281449321	0.24527208121399
C	-3.37412831156797	1.26932016312829	-0.22690117825071
H	-0.63512161293593	2.75516886503510	-3.08725703429867
H	-0.09396497974655	3.64484951150359	-0.99017991030714
H	-3.06436638718198	0.73526924660972	0.68025542742077
H	1.11423113027197	2.53201149700144	-2.96966013103238
N	-2.27239095556908	2.13468875271226	-0.61028939861575
H	-2.45913583911878	2.90902358927676	-1.23981556155516
C	-4.63848148096422	2.08371036747450	0.05899803836457
H	-4.88274783109568	2.69358096613214	-0.82460832692047
C	-4.43127993794270	2.93820550270951	1.28548076995211
C	-3.97527837480638	4.25660078671949	1.18324140724421
C	-4.59776700025854	2.38185290185939	2.56010459090396
C	-3.67707950238704	4.99980813831603	2.32650084812931
H	-3.84865144232086	4.70639085254959	0.19533482138533
C	-4.30313503735635	3.12131334254871	3.70631514676860
H	-4.95248541135969	1.35181016462221	2.65442409014229
C	-3.83425156261273	4.43215827284821	3.59100151420669
H	-3.31834070330412	6.02564794119198	2.22856512600551
H	-4.44124132828975	2.67318878489364	4.69248506252084
H	-3.59933086439268	5.01096326702604	4.48553279123776
H	-5.47057062416865	1.38227658211128	0.21661479743277
C	-3.56987378345683	0.24073656101823	-1.34226043377627
O	-3.35245134898335	-0.99091450587500	-0.91641483464514
O	-3.83597951007286	0.54560732773099	-2.47655858960983
C	-0.05609336533785	0.69060538297233	-2.87935123591660
H	-0.24532079345869	0.58511494061164	-3.95880153871718
H	-0.99136072303605	0.35162032165781	-2.40055571137062
C	1.09574137710573	-0.24598472230924	-2.49208461612676
H	1.84314018051210	-0.24845608349262	-3.30106237236422
H	1.63180021393989	0.12484606357438	-1.60234183208104
C	0.63402192922037	-1.68030594742610	-2.21269589911356
H	-0.03234972700998	-2.01877061627557	-3.02516877916511
H	1.50867086270339	-2.35198763372203	-2.22234878029040
N	-0.07093373320535	-1.86985314831647	-0.95054859221918
H	0.49771987468026	-1.50477565908621	-0.18463146189521
H	-0.91899506203272	-1.30204480075587	-0.92484160582760
C	-0.49122701480914	-5.40863486633162	0.43960462890224
C	0.13433987700967	-5.13067952321931	-0.92739009466183
C	-0.92519492159764	-4.80541056166064	-1.98637018142502
C	-1.95610438689209	-3.79480514446934	-1.54484687464127
C	-2.45796624090689	-3.92851755490547	-0.12600165177002
C	-1.36007560275140	-4.23714913708433	0.89270597191205
H	-0.48574588465668	-4.46378542386242	-2.93334773275052
H	0.81546788005905	-4.27191072254661	-0.84002450741009
H	0.72715556576197	-5.99407263625977	-1.26309790253050
H	-1.10587457164962	-6.32449021620940	0.38881127519236
H	0.29855199817768	-5.59473197673161	1.18309880300852
H	-3.03523065846958	-3.03584507195610	0.14365084864844
H	-3.16549913297400	-4.77828113205359	-0.16284226978494
H	-0.72922365571070	-3.34185327052031	0.99975531507476
H	-1.81465188134998	-4.44154683011732	1.87220578627894
H	-1.50722036266285	-5.71919460636551	-2.21053183486115
O	-2.45624813391230	-3.01329252989268	-2.33989220652162
H	-3.26845165365403	-1.66897255308638	-1.64441230037603

93

Intermedia O2*

C	1.68116446908288	-2.73114963122006	3.20990140977454
C	0.36291361164147	-2.87168979374959	3.61030191994553
C	-0.46223125165446	-1.74998879712519	3.67998705988767
C	0.01293884535632	-0.47831224864445	3.37643357868443
C	1.35581130524922	-0.32413136774509	2.97524181470176
C	2.16215340910296	-1.46015204396834	2.90257867491065
F	2.46699971194903	-3.78832736973440	3.11727299079343
F	-0.12489806836604	-4.06816132413691	3.87843585787621
F	-1.72442482363096	-1.96274095153553	4.01865474736667

I	-1.40255449510232	1.07331110252958	3.50296797656500
F	3.43975969496482	-1.37667344239909	2.53675854486546
C	1.93769158945409	1.04278935720562	2.67109801864389
O	1.64189214428534	2.01370920727960	3.33258411395646
N	2.76930946515286	1.09839643297718	1.59653081611744
C	3.19994321902837	2.36763708227060	1.04876430291260
C	4.60978537238068	2.28875335128437	0.44712758658789
C	4.69009240936134	1.34563181412400	-0.72861648922812
C	4.91109351549558	-0.02585203661382	-0.54156107347242
C	4.94825127784731	-0.89826950395636	-1.63183256546722
C	4.78242074618604	-0.40596599601154	-2.92603115192253
C	4.56798824014194	0.95920561240293	-3.12357875401459
C	4.51138224001981	1.82584915933585	-2.03357730181520
C	2.20656409878681	2.92974807201015	0.01132988063923
O	2.37380437958140	4.03151895491049	-0.48192167062034
N	1.18341220538013	2.10877826137555	-0.28098887564231
H	2.95960095307687	0.25566549371130	1.06219432879013
H	3.20180293070313	3.09457504210826	1.87273429156836
H	4.87969717266301	3.30511070690433	0.13114450563021
H	5.30483339271689	1.97918823030167	1.24043674095913
H	5.07671666062022	-0.41326300124625	0.46801960931365
H	5.12356960853522	-1.96313008633669	-1.46835278040169
H	4.82147973024235	-1.08466167284638	-3.77983032908507
H	4.43466072736168	1.34991546637953	-4.13403610418490
H	4.33169168849401	2.89282815431739	-2.18598569623856
H	1.10230744385283	1.22268828601208	0.20614232745175
C	-0.00415991861208	2.47681033375770	-1.01022353857530
C	0.11081560877577	2.34547785701742	-2.54457250065026
C	-1.09761072665528	1.55575130492591	-0.44723285381970
O	-0.80084947949516	0.57184396924274	0.21887549522471
C	-3.45517500925696	1.04463431586008	-0.23060255346031
H	-0.70037509937627	2.94484068503134	-2.98813135145531
H	-0.24980653283098	3.52442035130813	-0.76968419014319
H	-3.09744088679055	0.61850819922640	0.72330832651753
H	1.05265889186139	2.83613501649291	-2.83860218933521
N	-2.37565670433084	1.86411234784076	-0.73281760156880
H	-2.58401893881322	2.65908146010561	-1.32698352414364
C	-4.70964788909316	1.87029731860765	0.06642018469527
H	-4.99017451858594	2.45285977002723	-0.82550992853776
C	-4.46940445416631	2.77343061401164	1.25174134156725
C	-3.96816786982169	4.06957859303605	1.08877114509391
C	-4.65307065964566	2.28422943699037	2.55125885302715
C	-3.64042509083369	4.85436432026313	2.19554062608290
H	-3.83128017598465	4.47136404747662	0.08150529754083
C	-4.33095496795610	3.06630308191082	3.66162958973167
H	-5.04400505852422	1.27299849100752	2.69324505255372
C	-3.81506070495509	4.35247811415295	3.48541997077094
H	-3.24667641805133	5.86153231145000	2.04946276685629
H	-4.48401313146242	2.67030496147450	4.66770969342952
H	-3.55826492474403	4.96387224607678	4.35183955992547
H	-5.52318604567210	1.16321411584662	0.27391916743564
C	-3.73623368164517	-0.20967403461597	-1.06240612032104
O	-2.76897546245204	-0.50135207636672	-1.91009027366527
O	-4.70430155370258	-0.89945449263838	-0.86608191971446
C	0.02867385903359	0.91757524601670	-3.09027787678102
H	-0.16925808856917	0.97450913363566	-4.17201373183156
H	-0.86533816393514	0.43544220374854	-2.66519843495797
C	1.25955283233017	0.02528939667824	-2.86452267691411
H	1.93849510420845	0.10628393886872	-3.72741320794816
H	1.84625034924054	0.36964153210255	-1.99595456332147
C	0.88375387673559	-1.44123086601563	-2.63385465025486
H	0.19220380401044	-1.78128046119205	-3.42521545608627
H	1.78496625536225	-2.07184815699912	-2.70953647026641
N	0.24928336382905	-1.69860238204188	-1.34699309512395
H	0.88495823689314	-1.43283709104011	-0.59291996683255
H	-0.56318827984252	-1.09113668132180	-1.22635074835050
C	-0.33838235539627	-4.85665221227592	0.67160374851629
C	0.32264265429316	-4.86078557614107	-0.70660653357628
C	-0.69113256512129	-4.64366101598608	-1.83354975907545
C	-1.68855391509691	-3.53822539310380	-1.59104852551135
C	-2.18016195240601	-3.34435718992038	-0.17686841329160
C	-1.12836904383717	-3.57035066692120	0.90657472340964
H	-0.20923080282790	-4.45181793482371	-2.80216108071884
H	1.07112089893475	-4.05685709656827	-0.74942219472795
H	0.84854917522313	-5.81227463353318	-0.87283200286130
H	-1.01707025368099	-5.72277615302674	0.76298723700957

H	0.42648636962645	-4.97341317960363	1.45506671012735
H	-2.66041525872897	-2.36108005424863	-0.08979092931968
H	-2.98831335191687	-4.09186731584108	-0.06737905566586
H	-0.43950207455471	-2.71145602791028	0.91110199340813
H	-1.62525292953870	-3.59980273712455	1.88765962498171
H	-1.30820662219351	-5.55385529630258	-1.95569585141866
O	-2.21285551949239	-2.95473311020258	-2.52994865990625
H	-2.81281981972272	-1.44948139859671	-2.24022798362235

93

TS14*

C	1.72610209995469	-2.74837867645734	3.30806964155604
C	0.40182325621591	-2.89626161771178	3.68620211817684
C	-0.43406614152004	-1.78125422660446	3.73138280727711
C	0.03342526664819	-0.51003634309953	3.41502680378515
C	1.38133471095153	-0.34902849621290	3.03537098778480
C	2.20066096269598	-1.47689632493526	2.99261929362561
F	2.52137261575261	-3.80014609375234	3.23788181519349
F	-0.08269058717526	-4.09553850631399	3.94671769186591
F	-1.70046410350753	-2.00092686293018	4.04793534591395
I	-1.39700820393198	1.03145788011397	3.47784341293278
F	3.48214209467864	-1.38305046015492	2.64345687426775
C	1.95230344846803	1.01764657668418	2.71234325064007
O	1.65423717285973	1.99451488166616	3.36373429315198
N	2.77398763424269	1.06479521774476	1.62998737131497
C	3.19520873301720	2.33031942851395	1.06749065070335
C	4.59645481913627	2.25092979308988	0.44616256397367
C	4.66509721645353	1.30552264698387	-0.72850788049702
C	4.89873546568204	-0.06401619811136	-0.54328517682880
C	4.93270249958817	-0.93640943465820	-1.63385976457815
C	4.74992261126660	-0.44608396737483	-2.92651698392533
C	4.52090301183127	0.91720841621861	-3.12231529425074
C	4.46812737546430	1.78360658563616	-2.03189167014621
C	2.18976045202922	2.88277396967741	0.03776111900067
O	2.35335533268862	3.97952888747258	-0.46791182694442
N	1.16213199568984	2.06186411159693	-0.24083032804092
H	2.97163010697547	0.21576519931862	1.10860574090611
H	3.20805870555643	3.06343267633049	1.88586824833755
H	4.85849509948554	3.26701876241237	0.12292277081631
H	5.30386061479401	1.94643754345250	1.23046164869939
H	5.07947271442722	-0.44959390972189	0.46453995605489
H	5.12188636656197	-1.99916291818189	-1.47227306577925
H	4.78933198699040	-1.12425698683494	-3.78074559396235
H	4.37809312597143	1.30762088763260	-4.13167736006602
H	4.27959325196729	2.84928153136733	-2.18271586167360
H	1.07571901596253	1.17905807179686	0.25139697358979
C	-0.01436101482846	2.43533056910994	-0.98656578627054
C	0.12799929160227	2.30579082549406	-2.51986758889592
C	-1.12173618464396	1.51732517631183	-0.44969781825839
O	-0.83146592718833	0.49654081856134	0.16816249071891
C	-3.48768373962448	1.03280276520386	-0.24025120677734
H	-0.65551741839398	2.93209336159297	-2.97505245038559
H	-0.25620811937588	3.48441102967424	-0.75019955405284
H	-3.14954155096490	0.58390997600367	0.71048544485490
H	1.09094586526770	2.76674794907282	-2.79251046705148
N	-2.39347920693999	1.85828931314239	-0.70668490005139
H	-2.59113889584002	2.68968527419394	-1.25318745678352
C	-4.73904422858419	1.86267901492147	0.05269618933042
H	-5.02181181064863	2.43871629811082	-0.84274455640125
C	-4.50128138066210	2.77584087091855	1.23054890131353
C	-4.07533144461870	4.09655183433143	1.05468795653294
C	-4.61590930055584	2.27693467883037	2.53483325236720
C	-3.75518072075086	4.89778208515772	2.15238008813661
H	-3.99364496883997	4.50564655373820	0.04419990707414
C	-4.29996183850410	3.07435906221428	3.63573658757226
H	-4.95065537653817	1.24703908501701	2.68715176483339
C	-3.86050561551829	4.38703594724546	3.44581155337634
H	-3.42171383394820	5.92492939318566	1.99558919587701
H	-4.40027701977279	2.67084102308295	4.64559261055036
H	-3.60985111422584	5.01114691620521	4.30489620723884
H	-5.55064199935004	1.15432342227215	0.26397120958532
C	-3.76388236821198	-0.20313818175859	-1.10905148254462
O	-2.80772808862466	-0.46523953624245	-1.96447773249307
O	-4.73479000589781	-0.89367682842635	-0.91151463401554
C	0.00034954990321	0.88680243371349	-3.07921346737737
H	-0.15722230537142	0.96050032754624	-4.16648787509709

H	-0.93197564692086	0.44806838547196	-2.69140457340150
C	1.17679466176810	-0.07070643646811	-2.82807379416557
H	1.89865508364607	0.00752140561151	-3.65422119853317
H	1.73635467974250	0.20787099801050	-1.91880865182383
C	0.71459860246868	-1.52436507798146	-2.69178373437298
H	-0.00937359213285	-1.76860281876371	-3.48922169231614
H	1.56625234123108	-2.21404502942143	-2.79777926694939
N	0.06400200678139	-1.79830820087539	-1.41352533037489
H	0.75668571795251	-1.83359097684038	-0.66509303968494
H	-0.56846224410666	-1.03339827542183	-1.16276151681380
C	-0.30689982747669	-4.78560764174213	0.62845636844361
C	0.37300169124607	-4.78347394750608	-0.74197708850593
C	-0.61463701827115	-4.46222390669311	-1.86361545526498
C	-1.48999438538096	-3.25333540294848	-1.60906227597934
C	-2.01555160464590	-3.10271459947405	-0.19276877194841
C	-1.02110192740705	-3.46335224278746	0.90635115099264
H	-0.12042297674353	-4.33215125836821	-2.83716504368826
H	1.18790489554074	-4.04180708206530	-0.74649817174430
H	0.84010199305264	-5.76048072285100	-0.93431501004704
H	-1.04384614742694	-5.60627867030386	0.66673774728342
H	0.43092409265555	-4.98645040999199	1.42111051012589
H	-2.40971531706392	-2.08772187347970	-0.05091090959634
H	-2.88504417094237	-3.78339322304437	-0.15126454860249
H	-0.27947283807447	-2.65295723176641	1.00108177536224
H	-1.55231140431054	-3.51243423596698	1.86886124793102
H	-1.32592060108463	-5.30010708806488	-1.98165395943858
O	-2.13313949341740	-2.76810771097469	-2.56253784187486
H	-2.73615804702601	-1.46809494056283	-2.26363886351571

93

Intermedia R*

C	1.66494560440375	-2.55757199128286	3.39065145123992
C	0.32053065621499	-2.61634112317191	3.72341972985917
C	-0.44686338839846	-1.45280961035119	3.71631931226572
C	0.11254849787351	-0.21920948462947	3.39844278764738
C	1.47955809515944	-0.15224176625378	3.06579277912263
C	2.22832590183200	-1.32708611946752	3.06353785396004
F	2.39274976217114	-3.65953988735777	3.36138706417093
F	-0.24347684343247	-3.77859844735882	3.99389911247334
F	-1.73512977953461	-1.58565275024294	3.99007908119316
I	-1.20218595864853	1.41873411062193	3.39145220951661
F	3.51942916553481	-1.31351709021583	2.74246496938326
C	2.13759019703297	1.16658443076061	2.71491610106372
O	1.92316798338087	2.17076604550984	3.35644832624900
N	2.92124072220182	1.14252916702977	1.60241366011525
C	3.31847463030839	2.37193966805992	0.95098966005256
C	4.68925879024195	2.25524930489153	0.27158685356678
C	4.72174696441519	1.21099223349117	-0.81771917026325
C	5.03344956554265	-0.12425836637387	-0.52689043364445
C	5.04917047520366	-1.08997342707707	-1.53574282048438
C	4.76128751064251	-0.72991550491408	-2.85238604169743
C	4.44977273008524	0.59782013333940	-3.15277657667134
C	4.42106369803623	1.55838459117819	-2.14251972042300
C	2.26314548129157	2.86665870541888	-0.05868570798293
O	2.41755820116459	3.91829658460539	-0.65515989883894
N	1.20070269030429	2.05596269207777	-0.21956330638351
H	3.05559733650559	0.26923911492379	1.10061704338703
H	3.37107121240946	3.14964891259809	1.72534779539962
H	4.92136055617413	3.24279362587789	-0.14853776495994
H	5.43626227155320	2.02640819165735	1.04455195459993
H	5.28745248688540	-0.40675136780609	0.49891454116383
H	5.30873031512151	-2.12218667574534	-1.29413914317989
H	4.78875081301543	-1.48004955738727	-3.64468733667681
H	4.22978669630358	0.88799905238106	-4.18201815281229
H	4.17704937064171	2.59777225864174	-2.37592843861557
H	1.11297800556097	1.21093268358165	0.33455289717402
C	0.01804937920419	2.40077960884709	-0.97078786130476
C	0.19061107320647	2.33030333119107	-2.50889334912621
C	-1.06978076766945	1.43127245524249	-0.48624564794472
O	-0.76347941153809	0.39735450344885	0.10821506124839
C	-3.48456461893280	0.96230123222238	-0.43406465649204
H	-0.51756221107404	3.04240952335920	-2.95949800887896
H	-0.27224241936892	3.43125179454993	-0.70719256292504
H	-3.16960428863918	0.33012912465050	0.41357202360034
H	1.19803835432950	2.71340774584617	-2.73393179913954
N	-2.32781504599215	1.77042796866540	-0.77958267055206

H	-2.48525727303229	2.61389262669128	-1.32201709316836
C	-4.64972634407512	1.85259622960853	0.00396416221044
H	-4.84183010186860	2.60016656402566	-0.78368101156674
C	-4.40021846859701	2.52222178123741	1.33186342165124
C	-3.59995416012343	3.66804209579203	1.43480214948402
C	-4.92735317766132	1.97173550849588	2.50744799696971
C	-3.31841757576883	4.23753266261990	2.67787816280893
H	-3.19199606537078	4.12546753518384	0.53050176103500
C	-4.65729904683427	2.54114632466204	3.75172494656035
H	-5.55653283170763	1.08075782834365	2.44250654191564
C	-3.84557308162156	3.67395172327141	3.84252305711017
H	-2.68985033934434	5.12795103758396	2.73764558274347
H	-5.07674256824887	2.09585111498931	4.65553036626091
H	-3.62931313816092	4.11980533859338	4.81491585358235
H	-5.53799918895370	1.21234752630707	0.05464784282844
C	-3.87467093076587	-0.02754752596301	-1.57071423489779
O	-2.88706478922220	-0.58421253768538	-2.16316825313342
O	-5.05793819855795	-0.24362747821702	-1.77861845672127
C	-0.04474734081732	0.95442946585634	-3.13325003829733
H	-0.01349134505020	1.05566795198592	-4.22900663717767
H	-1.07463332067276	0.63670509665503	-2.90211706425625
C	0.95416853035385	-0.13114738001857	-2.70996322004756
H	1.84462606685886	-0.09587006927504	-3.35252546864859
H	1.32467772391226	0.04932934323701	-1.68790357887772
C	0.38188883135462	-1.54090990116952	-2.76667306387054
H	-0.14966428903603	-1.73940058771248	-3.70800524971070
H	1.18141780023100	-2.28442148986477	-2.65099469256171
N	-0.59626398366680	-1.73915466919716	-1.67024653905022
H	-0.15746008849276	-1.52067409206893	-0.76949594954454
H	-1.40128358184298	-1.03725337274024	-1.75345147472619
C	-0.19162084977793	-4.34094580789486	0.81551351707866
C	0.58413718768426	-4.29541354960822	-0.50156172541448
C	-0.36758993464087	-4.24191071310392	-1.69604650572754
C	-1.33771322486902	-3.07045130287373	-1.61587559426781
C	-2.09175851842035	-3.08227330582760	-0.27975508441900
C	-1.15961866707518	-3.16226432557465	0.93191527085533
H	0.16592405939129	-4.21397557086245	-2.65684502573572
H	1.25065777939793	-3.41297010435531	-0.50478574508308
H	1.24055718627467	-5.17230698580032	-0.59268859690856
H	-0.76777565654076	-5.28037837277109	0.85938363680446
H	0.50460541915382	-4.36117021963965	1.66653966363790
H	-2.74111562099768	-2.19594678931217	-0.22664616504392
H	-2.74625156245653	-3.96654722868669	-0.31226072932017
H	-0.59299773117149	-2.21740721927107	1.03270609120298
H	-1.75980340803179	-3.24318730512366	1.85072205539971
H	-0.99634980659301	-5.14528584674742	-1.71410800040506
O	-2.17405988716971	-3.10307110152276	-2.71160676742990
H	-2.75506794809871	-2.30716352728523	-2.66429631358345

93

TS16*

C	1.96795678571076	-2.91507957452377	2.48905910833483
C	0.63803572043173	-3.06758775195460	2.85003518188993
C	-0.19315272026190	-1.95095012772912	2.93407581702922
C	0.28839522397670	-0.67266856815291	2.67572323872929
C	1.64190321961983	-0.50863846674227	2.32939968765063
C	2.45601638293579	-1.63428284467897	2.23685973336910
F	2.75318816037866	-3.97035031500448	2.37918648330061
F	0.14438379398881	-4.27273989451575	3.06258432503298
F	-1.46466333482315	-2.17085898669834	3.22834917165428
I	-1.11275561506683	0.89346650473681	2.74987612794341
F	3.73703110234920	-1.51875502431922	1.89660775846882
C	2.22248763989325	0.86531096198352	2.06422332120088
O	2.04754475530719	1.78496161838563	2.83196089062019
N	2.88770388823245	0.97640989933129	0.88414797315598
C	3.24868280074405	2.26339601125225	0.32889960162239
C	4.64106432260593	2.25320725686505	-0.31612195428561
C	4.75709118504656	1.29403597391605	-1.47774790212734
C	5.06753595808480	-0.05718614400335	-1.26847987720178
C	5.18405589060028	-0.93907243965290	-2.34506455080067
C	5.00264763278824	-0.47816065102035	-3.64937398208265
C	4.68618210844610	0.86385129957776	-3.86940835639760
C	4.55556987521259	1.74004108920415	-2.79199910370512
C	2.20535815594914	2.76933180829455	-0.68848553536025
O	2.41168756464965	3.77038624584084	-1.35353382364697
N	1.09112279793820	2.02197509639309	-0.77304367214268

H	2.98609889739236	0.15609748642385	0.29119658841727
H	3.24541696273186	2.98140945104021	1.16136958354174
H	4.84216053709975	3.27651604328200	-0.65790705407834
H	5.37425966920766	1.99921184321908	0.46218777483244
H	5.24408709988913	-0.41812735637205	-0.25117300586235
H	5.43615549847551	-1.98536492310030	-2.16359054596049
H	5.10893239227557	-1.16270099364668	-4.49256485372130
H	4.54171946618268	1.23184036554802	-4.88683533835508
H	4.30611579317221	2.79001085824347	-2.96097688656456
H	0.99001114940103	1.19243999237783	-0.19961262133355
C	0.00673721874911	2.25380090780431	-1.70016388753372
C	0.40249324661232	1.98745785126710	-3.17390446748568
C	-1.11319488604615	1.31972960464548	-1.22069297253557
O	-0.83187012972440	0.26154864356289	-0.65875777093343
C	-3.48075486297229	0.93269281079481	-0.87738472127127
H	-0.28428587015803	2.56301966189015	-3.81308870382430
H	-0.31990187281942	3.30194688607107	-1.61174213605164
H	-3.09835876290109	0.46774409081388	0.04881087285136
H	1.40234367831667	2.42714554433826	-3.31274495486716
N	-2.38034036306355	1.70234405771620	-1.42267470396279
H	-2.57020945155655	2.57737864229169	-1.89932425429064
C	-4.65971520721502	1.83124381745377	-0.49566291687518
H	-4.98046349943202	2.41696436093081	-1.37183653692035
C	-4.28382625827948	2.73614472224002	0.65241342801329
C	-3.79960753092539	4.03007199280878	0.43226020567260
C	-4.32737541560249	2.25202768553642	1.96665312939617
C	-3.35131818313011	4.81786659016516	1.49438022051655
H	-3.77448983134506	4.43010682443998	-0.58497789727619
C	-3.88563722093515	3.03734546066625	3.03267753963669
H	-4.70726315819205	1.24376647397741	2.15398792975954
C	-3.38727049468508	4.32141851103345	2.79715422175387
H	-2.97377311519329	5.82358649955211	1.30256538415255
H	-3.93363411618049	2.64691661447493	4.05152934882821
H	-3.03757894278548	4.93490543035502	3.62886616262859
H	-5.48774265908793	1.16722658803179	-0.21564290533123
C	-3.88978450285490	-0.29027673400558	-1.71001569131837
O	-3.04601077122283	-0.58760121045379	-2.66299999036489
O	-4.85903684244966	-0.94169041037679	-1.40139884796660
C	0.35694342677210	0.52381172361313	-3.61792619294386
H	0.56487664440541	0.48085335280217	-4.69918686519452
H	-0.68354186665335	0.18164541586019	-3.50305324867731
C	1.31452939695686	-0.42932049011368	-2.88621034038719
H	2.33851822087082	-0.31301000823569	-3.26954946458660
H	1.35564870884970	-0.17450579957266	-1.81861820077243
C	0.90437332004092	-1.90240549296125	-2.97611771539434
H	0.99336061403920	-2.28782156408982	-4.00305789214847
H	1.56200828884708	-2.50061539209305	-2.33038184285787
N	-0.49997862663895	-1.99170086157134	-2.55894632936278
H	-0.70411075473870	-1.29565340805601	-1.83053030679411
C	-0.32462340382757	-4.96812182435138	-0.19535753273433
C	0.29867337798499	-4.99227796140305	-1.59176533135570
C	-0.68212019307349	-4.52880146931967	-2.67574592104568
C	-1.38441884449667	-3.21447417269933	-2.35634188920295
C	-1.96962844253065	-3.21911314671810	-0.94756859526388
C	-0.93652606229690	-3.60125967488326	0.10598158443396
H	-0.19180449104520	-4.44491425197605	-3.65773873441083
H	1.19101118306523	-4.34718217193670	-1.58827950726801
H	0.65525787718985	-6.00410089141500	-1.83474264564836
H	-1.11590868147633	-5.73410167409457	-0.12935669426610
H	0.43212224491433	-5.22270105093556	0.56291262157433
H	-2.39846206714346	-2.22845095832498	-0.73285073234917
H	-2.80370097503639	-3.93883796907701	-0.95117515194383
H	-0.14072035856088	-2.83172357317101	0.11814887570623
H	-1.40357051771704	-3.59500572316912	1.10175005733212
H	-1.49054036309940	-5.26836398262976	-2.79095510262420
O	-2.32455772427899	-2.85458608674605	-3.34000057778066
H	-3.01689360728577	-1.60627403386655	-2.94082662636932
H	-1.38110727952311	-1.97913452069066	-3.43686808523097

93

Intermedia S*

C	1.86510952427318	-2.88375741324844	2.57587918767006
C	0.54188821393880	-3.01494899447884	2.96475274016449
C	-0.27608770989722	-1.88787829771446	3.03235619738229
C	0.21073998229163	-0.62115072415188	2.72898738766770
C	1.55716411026271	-0.47792538852854	2.34133485993407

C	2.35787076157526	-1.61645662042445	2.27181004401292
F	2.64139365516012	-3.94800803556018	2.48350785633475
F	0.04075758977805	-4.20914629003925	3.21791950178536
F	-1.54343547010756	-2.08974687780275	3.35777473356966
I	-1.18668925977933	0.94836581553607	2.81956773011720
F	3.63520539008461	-1.53344960118188	1.90796227768150
C	2.14691103615269	0.88189374267352	2.02592338260200
O	1.92112872342749	1.84517516193138	2.72504136340841
N	2.89405518527066	0.93466278544396	0.89094657478311
C	3.29970802707489	2.19952302983620	0.31624800391677
C	4.69916693561437	2.13676756854874	-0.31021246624080
C	4.79015476268880	1.15307712302238	-1.45160819227097
C	5.07154750678248	-0.19962208985331	-1.21594170123469
C	5.13481013637823	-1.11044715409173	-2.27258576590055
C	4.93169905365808	-0.67626241043460	-3.58242941362306
C	4.65277191203023	0.66949042490015	-3.82855588004959
C	4.57189705315103	1.57447599897388	-2.77103662825006
C	2.28187387244430	2.73094164197648	-0.71248332097345
O	2.47420616400355	3.78408542220601	-1.29686083808605
N	1.20382994917020	1.95004322303353	-0.89384956887776
H	3.01820700221092	0.09310902020818	0.33398946620754
H	3.30695528330292	2.93535226934328	1.13256069976374
H	4.93265685475822	3.14817074576089	-0.66779941143114
H	5.41731398768634	1.87590484446691	0.47979006222970
H	5.25984616187272	-0.54062798811342	-0.19393034852500
H	5.35870001609818	-2.15947814711328	-2.07126905416276
H	4.99350598501301	-1.38449216648118	-4.41047120502676
H	4.49367424477061	1.01638732978824	-4.85133522104130
H	4.34586176905631	2.62647558173400	-2.96067800936664
H	1.09624940985209	1.09398879890409	-0.36009921499182
C	0.05602334853754	2.30237727904051	-1.69251693452037
C	0.25788911165725	2.10218930898705	-3.21278213238328
C	-1.07434312578225	1.41285154272722	-1.16213575213904
O	-0.81429435534975	0.38153756239642	-0.55395154008051
C	-3.45747940570355	1.02087045281260	-0.92931624592139
H	-0.47285648717421	2.74326989361541	-3.73057335314722
H	-0.18534136820072	3.36225911538882	-1.51105082992022
H	-3.09892561524775	0.50801264294262	-0.01874810445111
H	1.25369332910468	2.50578802074070	-3.45617399935280
N	-2.33813893284470	1.79767689514263	-1.41473702942789
H	-2.51053236958186	2.64194794422799	-1.94960778905077
C	-4.64239426634738	1.90574783070768	-0.53592757766980
H	-4.91886643376251	2.55360042783218	-1.38308953676192
C	-4.30018843763166	2.72391640220425	0.68552471888581
C	-3.69186704531159	3.97959696837690	0.57201691015854
C	-4.49732083590965	2.18845602482333	1.96427342619772
C	-3.27266604509794	4.67711773115195	1.70560088463541
H	-3.54308311999533	4.41908234157153	-0.41766393786726
C	-4.08621604539805	2.88505050460212	3.10217578721445
H	-4.96973186116673	1.20805167310388	2.06849742355748
C	-3.46343572715212	4.12880147005029	2.97462475554631
H	-2.79558308376041	5.65252874020796	1.59753394238805
H	-4.25077175247023	2.45368693362451	4.09164234542728
H	-3.13556426309313	4.67176682531519	3.86243077464356
H	-5.49167710018871	1.24027404207706	-0.33422861277537
C	-3.84316064057889	-0.15180673275436	-1.83553568666776
O	-2.86968268119345	-0.48453146629128	-2.66531053662343
O	-4.88055691705398	-0.74875439301677	-1.71630997903584
C	0.09057763559584	0.66842707752138	-3.71958256473092
H	0.05901760619870	0.69642634133917	-4.82079149359208
H	-0.90120552375130	0.30441897922364	-3.40971280178512
C	1.16383452417281	-0.34309799135027	-3.29732575808881
H	2.05770568674563	-0.21887713807084	-3.92447615908540
H	1.48997927332979	-0.16957022288119	-2.25937991281665
C	0.65553511655680	-1.78147173385682	-3.38568777071493
H	0.13041219269506	-1.91068595206519	-4.35704970308401
H	1.50997024823986	-2.48002448009026	-3.39459237571580
N	-0.19775978605849	-2.05911066266127	-2.24428883673661
H	-0.63815762782514	-1.21649657535491	-1.87911679825040
C	-0.12908518076038	-4.81665702955571	-0.06529870417860
C	0.57080396491901	-4.79525674219163	-1.42674702855239
C	-0.41473391620939	-4.46932256139836	-2.55012625056748
C	-1.13060482688075	-3.14184529683782	-2.28595581477342
C	-1.86438306467421	-3.20345011836777	-0.94526906720004
C	-0.90355625717522	-3.52361164474720	0.19606784310542
H	0.09998353264891	-4.42741213302091	-3.52483121583337

H	1.36576380926863	-4.03235028219814	-1.41735621499293
H	1.05223600811141	-5.76520353458985	-1.62137993067696
H	-0.83164924495550	-5.66740514244467	-0.03393661435810
H	0.60638061058016	-4.98574187085724	0.73712894573508
H	-2.36032792363079	-2.23594582805949	-0.76540384003591
H	-2.65096921871450	-3.96971298864119	-1.02389764721056
H	-0.19642966481016	-2.68421656241313	0.29211646607893
H	-1.46152208339200	-3.59663381047797	1.14226591531791
H	-1.18391906267171	-5.25427563925773	-2.62421768103539
O	-2.16977102604870	-2.96123445914513	-3.28091784618585
H	-2.92531475789490	-1.41836988068520	-3.00319866467836
H	-1.78730973696101	-3.08933942754233	-4.15981069539441

93

TS17*

C	1.92294649503887	-2.60209185838664	2.71699506840539
C	0.57173844611287	-2.70394783496685	3.00818598936532
C	-0.23670413729552	-1.56863735809547	2.96422547963744
C	0.28748039539768	-0.31861539045585	2.65009022163405
C	1.66031738764164	-0.20791288409394	2.35681600796708
C	2.45143050940204	-1.35390554493446	2.39674848074954
F	2.69271488801675	-3.67481716771798	2.73526583227059
F	0.04145007663319	-3.88305816868141	3.27733787823140
F	-1.52687948915212	-1.74453914785373	3.20490284811123
I	-1.07328473872255	1.28272969884261	2.61511861253777
F	3.75144425775264	-1.29434900636070	2.12004648337857
C	2.28575775054436	1.12565142202069	2.00103353934673
O	2.04956599678198	2.12754816865024	2.63861090880596
N	3.07138467295109	1.10854006851683	0.89060219368542
C	3.46735990408241	2.33772392813521	0.23779272934596
C	4.85114728814766	2.22875512536147	-0.41652667481824
C	4.90790421662700	1.18152025378654	-1.50233677407626
C	5.20860495267735	-0.15379803488434	-1.20113622623740
C	5.24425422332012	-1.12218699272311	-2.20695810762829
C	4.99055754771194	-0.76423707454147	-3.53089451918645
C	4.68850508318405	0.56328000491814	-3.84184757934253
C	4.63820549894412	1.52599016408944	-2.83469026122936
C	2.42950811716088	2.81154925252614	-0.79932762275284
O	2.59401329394744	3.85109924171747	-1.41487929769572
N	1.37187626673216	1.99695588978677	-0.95905597213716
H	3.21043604164109	0.23537863607213	0.38915283499363
H	3.49677673228063	3.12092339896907	1.00795004509862
H	5.08323675841227	3.21643164934912	-0.83616231576384
H	5.58631812783108	2.00706752657610	0.36977567510449
H	5.43587043807573	-0.43511446321200	-0.16871888369282
H	5.48784529364707	-2.15596879684782	-1.95562672492912
H	5.03177589761812	-1.51694581264146	-4.32012959982408
H	4.48986477453767	0.85099506985039	-4.87604501098678
H	4.39836436607674	2.56453483943526	-3.07558285430060
H	1.28703189223312	1.14963185305569	-0.40851681937305
C	0.21822733321294	2.30333984051113	-1.77221182960967
C	0.43944948875270	2.06840206104449	-3.28720265055899
C	-0.89569893897371	1.40396385991677	-1.22584719936846
O	-0.59776176240710	0.33951260291796	-0.67479087065217
C	-3.25810603014390	0.93976628644448	-0.98138673532150
H	-0.25922099341956	2.72518496564316	-3.82795295321661
H	-0.04182425635004	3.36273274029836	-1.61995629679880
H	-2.92420952905849	0.41160839093216	-0.07392694647468
H	1.45275796374744	2.43361743776098	-3.51845144029528
N	-2.15969831201823	1.80109394165844	-1.37701803234275
H	-2.35305397497151	2.63240118894003	-1.92592758167045
C	-4.50391496446580	1.75115528243991	-0.61307576733857
H	-4.72648754025520	2.47369956987659	-1.41536244084034
C	-4.32529034768579	2.44567754133101	0.71478044019189
C	-3.57490640247222	3.62359280923465	0.82612397559626
C	-4.82685760527647	1.86110865341674	1.88456557391636
C	-3.31034574517167	4.18854719388073	2.07489780591194
H	-3.18485982578386	4.10323974223205	-0.07442790344058
C	-4.57386678764076	2.42669087748776	3.13443102072557
H	-5.41250309635204	0.94143340047525	1.81202065422171
C	-3.80594463371552	3.58848392314138	3.23494427660733
H	-2.71672394616792	5.10196533740689	2.14277567521716
H	-4.96940252150628	1.95334923204894	4.03474114529205
H	-3.59881202601009	4.02848533932470	4.21184354169313
H	-5.33810954197842	1.03985511520229	-0.56034344176135
C	-3.53551123292074	-0.19979734040400	-1.99631898181787

O	-2.79177543194838	-0.24587172785218	-3.01745496715627
O	-4.40251171416103	-1.02014766944758	-1.67895676224724
C	0.21781293985677	0.63694261664460	-3.78067611165825
H	0.24483456871822	0.64664037685461	-4.88214867988905
H	-0.81071783263485	0.34310610166053	-3.51830085210688
C	1.20683254778409	-0.42823642182521	-3.28761221772691
H	2.12633286976012	-0.40342878583860	-3.88828306421130
H	1.51892225298094	-0.23550332799750	-2.24839616554183
C	0.60915079376811	-1.83239192279602	-3.35227491772256
H	0.14222580319474	-2.00695602389774	-4.33675246624692
H	1.39244242618018	-2.59363793536096	-3.21049329412451
N	-0.39603749898038	-1.96856759338739	-2.30268565068966
H	-0.59805308577685	-1.11941544139922	-1.76298456185402
C	-0.41573932736732	-4.93284871511032	-0.11762043641038
C	0.20383105973289	-4.92514019495662	-1.51865514469067
C	-0.77881997982391	-4.35761644101785	-2.56120027384043
C	-1.18693283179515	-2.99569756881392	-2.08139881860475
C	-1.90767307163251	-2.98657369667506	-0.75999743531924
C	-0.93056292043371	-3.54948399436384	0.28318392155106
H	-0.33388434706612	-4.31219091014730	-3.56364784014809
H	1.11848143542769	-4.30868304638785	-1.51193962397380
H	0.50269772763207	-5.94011069484200	-1.81633093910132
H	-1.25460399259535	-5.64877752665794	-0.09727796573004
H	0.32121969640834	-5.28213257876522	0.62119341959046
H	-2.22409048568246	-1.96726932654058	-0.49846534869266
H	-2.80255640907745	-3.62130704501497	-0.82171456499075
H	-0.08134763048467	-2.84983731716677	0.36583101355353
H	-1.42485251517323	-3.58561037293363	1.26431693586434
H	-1.67620470390505	-4.98764541918778	-2.61746097810253
O	-2.73586436543243	-2.73917680857728	-3.31513418448963
H	-2.73763099077579	-1.71024634157926	-3.37108254858757
H	-3.55874581835742	-2.85998483754328	-2.81090231025940

93

Intermedia T*

C	1.83021153297110	-2.84399479783049	2.76701203318940
C	0.47764329417242	-2.92354095302085	3.05816590599729
C	-0.30667316869564	-1.77276512723838	3.02409872433582
C	0.24244355936413	-0.53358770930349	2.70689657478858
C	1.61403584545038	-0.44734396624974	2.40182414282144
C	2.38285270104822	-1.60963822128752	2.44113060230605
F	2.57295371563658	-3.93627532775473	2.75819033313877
F	-0.07593083240173	-4.09685109348465	3.29897357997950
F	-1.59954727252330	-1.92379435917789	3.26202231170164
I	-1.11397373893754	1.06972119849455	2.64180821939678
F	3.68146791061663	-1.58446379753929	2.15316794791752
C	2.25183620380586	0.87036824650314	2.01417237027529
O	1.98538200185122	1.90149557092421	2.59107819969176
N	3.08269362799899	0.80917187592546	0.94016990988196
C	3.49979652376640	2.01539201850232	0.26293746543160
C	4.84788210809440	1.83756253994819	-0.44926905152647
C	4.86352500440487	0.65707521619644	-1.38912349489014
C	5.29849321477099	-0.59822321809949	-0.94093374533473
C	5.28629719653786	-1.70564225770739	-1.79016616245609
C	4.84643430032375	-1.56875424666696	-3.10736106900262
C	4.41589460543203	-0.32228172979775	-3.56548178501433
C	4.41468515884925	0.78071676852146	-2.71067284681660
C	2.42314409655180	2.55394547418895	-0.69952128564640
O	2.55844678852205	3.65000047409074	-1.21531926138916
N	1.35307710322277	1.75889508963208	-0.89351178485537
H	3.25296132872380	-0.08093634409835	0.48150948555213
H	3.60211410654408	2.80601771117669	1.01919863077919
H	5.04712259810067	2.77103539686866	-0.99304553886001
H	5.62676098211174	1.71495995319320	0.31605631816117
H	5.66114459879679	-0.70432299115799	0.08515641636799
H	5.63842283881108	-2.67204824500897	-1.42481583999581
H	4.85259382613292	-2.42802635018729	-3.78019863371427
H	4.09059971506045	-0.20616210104726	-4.60102284863717
H	4.07787702767053	1.75647157066650	-3.07192947734342
H	1.29399802061839	0.85170790222981	-0.44182227450031
C	0.11305545511960	2.23053486801634	-1.46823323302862
C	0.13760859126099	2.42909085611178	-3.00157122668121
C	-0.95352040049237	1.20977994279962	-1.04860908946764
O	-0.64020911340761	0.06827110601528	-0.72157185519746
C	-3.32220911144410	0.77356186123428	-0.72069624788996
H	-0.59700437681447	3.21166674291789	-3.24574786557902

H	-0.12343582291515	3.20389323181551	-1.00719056839420
H	-3.03332127832681	0.22913291224585	0.19429009870704
H	1.12526372567674	2.84317407265497	-3.25611585810019
N	-2.21974933838943	1.64267757287579	-1.07186454208440
H	-2.41019238681822	2.59023020684341	-1.37919772156380
C	-4.58180460225765	1.59423400011353	-0.42211799342775
H	-4.87447988964284	2.15164788955969	-1.32645581556322
C	-4.36930008579893	2.53976968796500	0.73412449249351
C	-4.04747765454132	3.88580003191930	0.52464870728439
C	-4.41292177499673	2.06180083792799	2.05053538859957
C	-3.75444243398717	4.73004724069419	1.59748128241597
H	-4.02950414342476	4.28148575920882	-0.49483118049367
C	-4.12475468702605	2.90184259942719	3.12707419592466
H	-4.66725010076741	1.01374437889926	2.23075166133998
C	-3.78629555676546	4.23872236995899	2.90234439048969
H	-3.50179821259590	5.77548376974012	1.41252740269305
H	-4.16427885324250	2.51118099769363	4.14593740909834
H	-3.55716299123935	4.89543772619571	3.74297037295156
H	-5.37965101845975	0.87348992865415	-0.20085977648425
C	-3.60040770554296	-0.33672436837816	-1.76334441241122
O	-2.90228110247573	-0.33983478414218	-2.82056181805279
O	-4.46340686996693	-1.16101091302775	-1.44571958386098
C	-0.19585306997758	1.18586067646699	-3.82188089819193
H	-0.27704128137567	1.47117652628457	-4.88291604605009
H	-1.19803272332887	0.83772690037974	-3.52713782587957
C	0.81081607231938	0.03884321650287	-3.70291591077948
H	1.68435815482048	0.24031572522151	-4.34001343190571
H	1.19007181496703	-0.05696124687650	-2.67430480834272
C	0.21349204570531	-1.30654086234235	-4.11677511877411
H	-0.38706876571252	-1.19713761390523	-5.03274004045193
H	0.99762760703453	-2.04381116815782	-4.31166889886723
N	-0.68190941657341	-1.80172928214435	-3.07235950087471
H	-1.52732157802813	-1.18627036983701	-2.87692159960458
C	0.29424493854308	-4.34808735090441	-0.01532911430596
C	1.27193777484874	-4.03661254602873	-1.14467519288515
C	0.53505454139661	-3.86412163610304	-2.48562461588484
C	-0.56370549230289	-2.85548266820201	-2.34944881891434
C	-1.53894723147783	-3.11785110994604	-1.24412386673041
C	-0.77210612436764	-3.25921649569808	0.08026818636526
H	1.23388289419547	-3.63074407420880	-3.29413252088342
H	1.81882085002312	-3.10681502973286	-0.91374973592737
H	2.02114957218555	-4.83283722710136	-1.25474216887678
H	-0.19126807970724	-5.31994442403073	-0.20629702280273
H	0.83769333465614	-4.45282953117759	0.93479254714024
H	-2.30638496776982	-2.33919167228024	-1.19710271195064
H	-2.05068179249522	-4.06221529602387	-1.49029300064797
H	-0.30069441689606	-2.28793068488883	0.30627013181303
H	-1.48739001761696	-3.47147373272003	0.88864897883208
H	0.03698415828989	-4.81477140091901	-2.74070567901285
O	-3.62016190588326	-2.98111373334101	-3.47320496643232
H	-3.34875535626199	-2.07434088537576	-3.68901012981066
H	-4.16055332333203	-2.77089369925380	-2.69331890481021

93

TS18*

C	2.43445855505019	-2.43986302028260	2.36311105453976
C	1.10629027632437	-2.72342140372015	2.64386378129074
C	0.17964433218455	-1.68418883778026	2.71878849902150
C	0.55767174445892	-0.35585453703888	2.53949717480091
C	1.90189935379438	-0.06377755804790	2.23628932166784
C	2.81449413626595	-1.11573177812792	2.16568256321919
F	3.32618658484804	-3.41039602190655	2.29285584180009
F	0.72131028293920	-3.97264809649963	2.82865380107977
F	-1.07780449840084	-2.02156598948418	2.96617596014100
I	-0.97766051885141	1.06824549847729	2.72431042953577
F	4.09714884695457	-0.89190453987389	1.88967734120803
C	2.36228973405804	1.35285537965906	1.94821625749557
O	1.99011845691927	2.29630911985072	2.60918971914981
N	3.15971562049493	1.47159344614722	0.85135876743333
C	3.39754185225418	2.75089209924232	0.21784553841991
C	4.79416630776221	2.82100007447024	-0.41795662988463
C	4.98981705306974	1.77512264152314	-1.49010390421426
C	5.50898547300253	0.51166388290575	-1.17753139194435
C	5.65431706619972	-0.46554032953137	-2.16507089312917
C	5.28901433876455	-0.18771865773547	-3.48183375683850
C	4.76869849919697	1.06765255453290	-3.80438706862823

C	4.61496183162065	2.03857592127825	-2.81598647026035
C	2.33235860308463	3.07795034986885	-0.84909786989287
O	2.38191932515299	4.11288268910968	-1.49086335833541
N	1.39645234948184	2.12782592582089	-1.01102448949891
H	3.42564002585682	0.63655762411149	0.33568503843750
H	3.30801078555860	3.52597130142562	0.99150219208167
H	4.90146332657903	3.82592446146719	-0.84690825365439
H	5.54253074018071	2.70164720759994	0.37768191681540
H	5.81491908775033	0.29516219974011	-0.15018656576150
H	6.06635137340518	-1.44201123119580	-1.90454866230127
H	5.41161907658611	-0.94653242775675	-4.25679925968112
H	4.48112376898758	1.29251265078828	-4.83340720148231
H	4.20619381979889	3.02047781261835	-3.06647751836454
H	1.38548673770712	1.31807933398131	-0.40152535195907
C	0.24539372899779	2.23193784419623	-1.87226019106222
C	0.52474230751175	1.82753279707444	-3.34084795870616
C	-0.79836659387366	1.30317720362040	-1.24111042492414
O	-0.42459957319771	0.38827092822158	-0.50277235823431
C	-3.12569448988662	0.62889840047134	-1.05429175343034
H	-0.22022146047608	2.33763217340459	-3.97090385276386
H	-0.11082060630268	3.27389695104204	-1.85507617178261
H	-2.72652677668174	0.16019688350575	-0.13997950037263
H	1.50600313881245	2.25128237657773	-3.60897867015063
N	-2.08216398066473	1.51097588780139	-1.53674320810300
H	-2.32180268297259	2.23335914821784	-2.20813568998278
C	-4.38820440785359	1.40703211650647	-0.66961978258299
H	-4.69216244603883	2.05490741379489	-1.50821968461277
C	-4.16407430328677	2.21208215830721	0.58654897965153
C	-3.48517458204979	3.43777714772883	0.55408720896744
C	-4.54889701535613	1.69547297668416	1.82943982489032
C	-3.17640863220495	4.11693182330203	1.73250344979756
H	-3.18768102115840	3.86337100200587	-0.40679914148813
C	-4.24938272918284	2.37428909825407	3.01192275229899
H	-5.07871521470594	0.74035501004235	1.87021339953053
C	-3.55207753048335	3.58356389859950	2.96772889318212
H	-2.63958108592858	5.06578934902653	1.68815540614691
H	-4.55548628202461	1.95374870509810	3.97175058400708
H	-3.31033859489226	4.11294708753243	3.89083759383993
H	-5.18135648283397	0.66499928013425	-0.51434311210694
C	-3.39775559730438	-0.56923927051731	-1.99138348947795
O	-2.57009349746848	-0.74989772733897	-2.93606483623155
O	-4.33660990038862	-1.31480553264007	-1.69816023420366
C	0.44831638238446	0.32864530869762	-3.63999776685747
H	0.48446036270800	0.19552012792961	-4.73291738329290
H	-0.54909511433048	-0.02939355273672	-3.33817218900054
C	1.52543488271614	-0.57365565591046	-3.01769008529874
H	2.43201807716555	-0.57318653157391	-3.63847796569984
H	1.84413826864816	-0.20606106501503	-2.02777307748129
C	1.02014218581600	-2.00660137616113	-2.85803510722531
H	0.48212881203140	-2.31949719713368	-3.76881985435764
H	1.85266301680539	-2.71336370659043	-2.70712694257802
N	0.12027485695192	-2.06822500883317	-1.71601548363038
H	0.02947226699351	-1.20994763951102	-1.16363634719448
C	-2.44031223559116	-5.16243592188666	-0.42580664967912
C	-1.46080587320675	-5.46729049775637	-1.55895669768484
C	-0.89134703168554	-4.20385423199071	-2.19839389326599
C	-0.69334481857198	-3.05730638615626	-1.40178919271996
C	-1.56981947121241	-2.83658977592072	-0.19466381506897
C	-1.85127663713875	-4.14249959948203	0.54616152709451
H	-0.14604271252952	-4.37573662430457	-2.98230819474618
H	-0.63608685978979	-6.08499381878171	-1.16210980286981
H	-1.95616708917699	-6.07690804326564	-2.32986231640892
H	-3.37670164056366	-4.75222800390956	-0.84275273819551
H	-2.70592604822646	-6.09180791678152	0.09860454262889
H	-1.12803612187549	-2.06442634030848	0.45172532248045
H	-2.52860324718507	-2.43127946457079	-0.56502199110331
H	-0.91862316069156	-4.53411303099609	0.98352533910313
H	-2.53984891506276	-3.94707195409119	1.38005707207856
H	-1.97991193040227	-3.63828859226046	-2.86604337841961
O	-2.95276389439940	-3.11748787556321	-3.46603416032753
H	-2.76339821029696	-2.06772317307472	-3.36925079122596
H	-3.74789297212895	-3.18893527085128	-2.90877677445435

93

Intermedia U*

C	2.13973115036994	-2.75618483524959	1.24670487084613

C	0.91809488283248	-3.00746457408117	1.84749192986747
C	0.16538778035173	-1.94109216627692	2.33711566355596
C	0.62723316063581	-0.62864642657374	2.27097544801710
C	1.89103812131697	-0.36701755158912	1.70071502061013
C	2.61443925807380	-1.44941953270574	1.19574710766239
F	2.84609650681202	-3.74296979399708	0.72703922689693
F	0.46514425687932	-4.24308014742736	1.93407372362873
F	-1.01633168604111	-2.23459551972381	2.86348026559508
I	-0.72357985904041	0.83268168535852	2.95475212984832
F	3.80187099889536	-1.27844951382766	0.61525538157889
C	2.45776863400237	1.04414633921396	1.63642895994441
O	2.24840314840812	1.85210420770290	2.51517968719551
N	3.13897388510023	1.33902440068596	0.49768803056994
C	3.41633841360736	2.69054948269341	0.05325771528182
C	4.80348960863769	2.78909023162647	-0.60199089099627
C	4.98981689857506	1.77619386965767	-1.71026781284699
C	5.48605714373890	0.49726233649906	-1.42143971362628
C	5.60885336343443	-0.46828757387009	-2.42212498045809
C	5.24632092466129	-0.16358084031397	-3.73449678146137
C	4.76071671165356	1.11005890741347	-4.03713271227280
C	4.63043668331484	2.07088048663872	-3.03381689851741
C	2.33376354928650	3.17166708187146	-0.95055586763055
O	2.56376574176962	4.03827469230770	-1.77556430185427
N	1.15426364497159	2.53462114006587	-0.83488820669862
H	3.27855209133223	0.60979128278738	-0.19481504254637
H	3.37426836986114	3.34562140426108	0.93565035147017
H	4.90838933353916	3.80850114949410	-0.99340514379264
H	5.56081853132979	2.63418833637189	0.17860956260537
H	5.77926248328882	0.25576774985924	-0.39659990487379
H	5.99476314599444	-1.45868913720179	-2.17438429800095
H	5.34589313241820	-0.91456456843538	-4.51997063185236
H	4.48176051435229	1.35893798508021	-5.06275155553116
H	4.24348646944805	3.06416362845431	-3.26768452062909
H	1.03974149205380	1.83317130073168	-0.11267802893728
C	0.10779514997148	2.49384023226991	-1.83037650634771
C	0.58340792515304	1.86962307935995	-3.16901271679846
C	-0.97594259565735	1.60771158904023	-1.21124347616374
O	-0.66856530153806	0.79988903013107	-0.33960076461383
C	-3.26439124499043	0.81747052669550	-1.20420457691009
H	-0.11775135846224	2.19226689670455	-3.95440551317140
H	-0.28313046080964	3.50574617808567	-2.02055454934716
H	-2.82236988774783	0.30220788240027	-0.33425327879835
H	1.55314077155275	2.33336133002567	-3.40773609422953
N	-2.22790454067508	1.70465396251310	-1.68503502461801
H	-2.43522607707387	2.36275803224610	-2.42903571507275
C	-4.50337766298773	1.56624304847248	-0.70663163980300
H	-4.81685269844693	2.30846388717764	-1.45758752589104
C	-4.19962558116870	2.21931096072425	0.62136624072589
C	-3.49604162567820	3.42907950405744	0.69034619618129
C	-4.52722244430832	1.56607641702020	1.81556755718248
C	-3.11703104823582	3.96491713914796	1.92134469931025
H	-3.23857798036483	3.95478423031736	-0.23215280714000
C	-4.15817802477685	2.10296089814135	3.04963209005866
H	-5.07363765253026	0.62045622609455	1.77645417285895
C	-3.44518307870170	3.30205508136976	3.10609671535758
H	-2.56543431248664	4.90589776877334	1.95697772188231
H	-4.42484296011081	1.58028272447719	3.97015138678886
H	-3.15334203336874	3.72373462195114	4.06969018203274
H	-5.31259609339042	0.83253782729796	-0.59871775948639
C	-3.54791332107116	-0.35312634404154	-2.14390481802175
O	-2.53221719839862	-0.62188035338345	-2.93560585231537
O	-4.54832670986383	-1.02395861472787	-2.06130184645187
C	0.66462903377135	0.33833825716048	-3.20326396024088
H	0.92476532406209	0.03896471975947	-4.23145861391155
H	-0.34226050556476	-0.06620801285144	-3.02204517057233
C	1.64517104935882	-0.34411257933453	-2.23944181535663
H	2.67982676031327	-0.26186093180022	-2.60901049661893
H	1.61117069703930	0.14989802235924	-1.25953644369247
C	1.28429229863454	-1.80893502983419	-1.99464284984112
H	1.14221301020808	-2.33976856193214	-2.95701747523734
H	2.11922888186677	-2.32570492113565	-1.47951666915294
N	0.07601325081106	-1.87235113989288	-1.21191841417074
H	-0.29799926056799	-0.99877713716801	-0.85305312989241
C	-2.36524540028888	-5.24245419121156	-0.43168280731891
C	-0.92019941537144	-5.52133783138285	-0.84540490319362
C	-0.18287263233145	-4.26813759899285	-1.25164648292249

C	-0.59987825419584	-3.02139193053220	-0.91156383030760
C	-1.91429503563721	-2.78896013190976	-0.19344238754315
C	-2.43995590938582	-4.03949857743845	0.50571114126125
H	0.77999686376304	-4.38773264809890	-1.75505444592555
H	-0.39157925039277	-6.00091820178704	-0.00073581017538
H	-0.90038572248825	-6.25828078376730	-1.66446960895633
H	-2.97342467273863	-5.03651753848877	-1.33048331897073
H	-2.80298693625234	-6.13280342476068	0.04310331709539
H	-1.79948638366507	-1.95263495900282	0.51753297791582
H	-2.65088052870625	-2.45194253609056	-0.94286862309733
H	-1.83748011629185	-4.24202030256547	1.40380061456032
H	-3.47274690315156	-3.86726150257579	0.84177668598825
H	-1.80321565607223	-3.54917290265690	-2.95107823354406
O	-2.53205431989360	-3.15658665380580	-3.46829042234587
H	-2.58568544503778	-1.57192280907054	-3.27022735186941
H	-3.34304226149320	-3.52671544103513	-3.09237175180917

96

Intermedia R* + H2O

C	1.33479825887128	-2.39341553119457	2.76668670310449
C	-0.04236360079175	-2.24046178481814	2.85620505867271
C	-0.60535724647486	-0.96953334304562	2.75985155210073
C	0.19307137341704	0.15657360041850	2.57935188451124
C	1.58182620164558	0.00039786719989	2.43266406781697
C	2.12922208138371	-1.27575231766705	2.53588806462532
F	1.87605321616754	-3.59493719177103	2.85568747635134
F	-0.81132368200660	-3.30084407875676	3.01322472727404
F	-1.92358809654074	-0.87913404532370	2.84583576468122
I	-0.80878980487316	1.99730308598617	2.50295432479317
F	3.43837511060222	-1.46465105020706	2.39845944377437
C	2.45787098045887	1.16965345007805	2.03364315959466
O	2.40383892997430	2.24413013618394	2.58688346661484
N	3.22557868510366	0.92474611703250	0.93484682758302
C	3.69914223768046	2.01692574330451	0.11372641117376
C	4.97115340204726	1.64449262797963	-0.66329724866211
C	4.83201570710684	0.37167564263704	-1.46407979341170
C	5.03438944114160	-0.87701831784343	-0.85490692981685
C	4.86327128726040	-2.06066091577998	-1.57276796554854
C	4.50773233086756	-2.01306199426622	-2.92205004805750
C	4.32259661312060	-0.77762518514673	-3.54236671109090
C	4.47286249494865	0.40526702149114	-2.81625927236194
C	2.59499173898108	2.56716038163622	-0.81854388721312
O	2.76513138007670	3.61133151928336	-1.42215086981772
N	1.46257111453923	1.83742103217226	-0.87361952973344
H	3.19174152314834	0.00078696729015	0.51211999169540
H	3.93050422412781	2.85828006942686	0.78110692304415
H	5.20444359609057	2.49183842771173	-1.32229130236561
H	5.79083967616808	1.54096819631342	0.06163123003306
H	5.33423353711618	-0.91929278994561	0.19583162935881
H	5.01850309988681	-3.02204852532855	-1.07985652126515
H	4.38551047203248	-2.93690957445953	-3.49076497594477
H	4.05396388983927	-0.73400365232402	-4.59974115476545
H	4.31493887648916	1.37143024584746	-3.30333431897941
H	1.40611481213009	0.94584438392007	-0.39345271517212
C	0.21091501586975	2.29023016539859	-1.44044391202953
C	0.15125407964742	2.35928341150321	-2.98521268666495
C	-0.84429827996469	1.30948875569990	-0.92347831970224
O	-0.53641430076157	0.16367075789862	-0.60317237170000
C	-3.17938753517032	0.85889743108917	-0.48640959943465
H	-0.66018009942517	3.05625701154828	-3.24598061713272
H	0.00028300898082	3.29833134417757	-1.04616613667621
H	-2.86070171267871	0.30560192060345	0.41016319598294
H	1.08891626666801	2.82827703518257	-3.32117774326187
N	-2.10848622144890	1.75210087164964	-0.87624317970488
H	-2.32613694750451	2.68033223466215	-1.22111967597632
C	-4.46154496872322	1.64436612021208	-0.17433424106632
H	-4.70711246948614	2.28251724308475	-1.03802830437763
C	-4.31720885467117	2.45904038411448	1.08759934232556
C	-3.78790332446080	3.75453374919808	1.06043946095544
C	-4.64028143776654	1.89215007341409	2.32662156603038
C	-3.56323330154945	4.45906247871619	2.24524666321172
H	-3.55314607073540	4.22582709577106	0.10241322674995
C	-4.43197800805475	2.59698949878562	3.51103700398990
H	-5.05243924406207	0.88044123500871	2.36062832540217
C	-3.88402863011925	3.88043702796659	3.47465615362927
H	-3.14630177780527	5.46701054179262	2.20657061477577

H	-4.68951453129738	2.13934526526635	4.46759644375929
H	-3.71201845101683	4.43093276078472	4.40094382088838
H	-5.27611320150583	0.91563550454616	-0.05844073816019
C	-3.43462927048204	-0.19615173106886	-1.56369969210179
O	-3.39143009619735	0.29674149339466	-2.77907125348145
O	-3.67245117867395	-1.34771791543028	-1.28490914289696
C	-0.09349577699903	1.02524834508708	-3.69079492877290
H	-0.28837900128507	1.21671928654898	-4.75828919160702
H	-1.02114851298964	0.57778335710139	-3.30138813484599
C	1.06759273968298	0.04199888487168	-3.56593105568422
H	1.90873750546707	0.39901517855035	-4.17855354155325
H	1.44028058228057	0.02010075787490	-2.52951748511920
C	0.73344895577533	-1.38882210573121	-3.96206806573782
H	0.38833674556254	-1.43995071667747	-5.00791530768573
H	1.65961075566860	-1.98385541740785	-3.89458123365285
N	-0.32320010225050	-1.91898434818136	-3.09629587230307
H	-0.28472231411087	-1.48848233185710	-2.16987544949349
C	0.21661428156534	-4.09827012725333	-0.19639942785146
C	1.30261219703885	-3.75295464414880	-1.21681495956417
C	0.83282651507528	-4.10769930132203	-2.62716667552644
C	-0.46409060285294	-3.36804571724380	-2.99377634043933
C	-1.54317167244972	-3.67131055221792	-1.94659874310589
C	-1.09098927546918	-3.37201879194706	-0.51455001651281
H	1.60818444609605	-3.91180396723874	-3.38115532145024
H	1.53191123279402	-2.67181436453423	-1.16591916112498
H	2.23957584333712	-4.28181717250876	-0.98551323031157
H	0.04050065165636	-5.18785063540129	-0.22343932728075
H	0.55576681585969	-3.86984310543095	0.82257515829146
H	-2.44443148483892	-3.09542610521542	-2.19956017699580
H	-1.78500998277584	-4.74429202237340	-2.03572265245005
H	-0.96130747714205	-2.28319562420955	-0.38011736547162
H	-1.88263281652090	-3.66707751400026	0.19029366019096
H	0.61794481721677	-5.18980831808277	-2.67096311207871
O	-0.90234450356069	-3.76768039693088	-4.28195596592069
H	-2.94548079382378	-2.31613216757790	-4.58804619670045
H	-1.76222899461591	-1.56937928267838	-3.93074493289767
O	-2.62892255653564	-1.41868124828489	-4.42068591582601
H	-0.90952662207714	-4.73238583722299	-4.31131213283548
H	-3.28702191411973	-0.45558697736114	-3.47298256361251

96

TS16* + H2O

C	1.30466968996791	-2.43141749854989	2.88207619652215
C	-0.03444583308629	-2.20556382020460	3.16542037486748
C	-0.56636432575191	-0.92494950986133	3.03455849375406
C	0.23932606272689	0.15054341746180	2.67157867022178
C	1.60033358993016	-0.06873194817239	2.39544381564144
C	2.10598293786278	-1.36291213261350	2.49672259871214
F	1.79475407815218	-3.65626421298728	2.93457240709478
F	-0.81303938252396	-3.21979853377720	3.49084707466399
F	-1.86163028976696	-0.77754083276957	3.26186596822259
I	-0.73313390801485	2.00072408997607	2.49446943210775
F	3.37762417314148	-1.62493628749360	2.20654051366537
C	2.49525225217572	1.06559922567741	1.94503716315823
O	2.48376676436217	2.14501789610129	2.49253665685984
N	3.23936140082538	0.79413777883515	0.83797148758309
C	3.74642289221449	1.88100970073712	0.02985429870141
C	4.99340702473770	1.48209473259616	-0.77086205226377
C	4.79386118281877	0.27109455715262	-1.65067835766970
C	4.85964370123593	-1.02469093671151	-1.11334521771838
C	4.64962271319360	-2.14494755343324	-1.91752742640287
C	4.39635145520273	-1.98768976319171	-3.28190229962962
C	4.34764798918785	-0.70617669814273	-3.82996989331467
C	4.53317199988201	0.41405215410342	-3.01817611628743
C	2.64888105678970	2.49442412780314	-0.86975083709180
O	2.85652816283875	3.53141353346624	-1.47414912307197
N	1.48330903329880	1.81847465340654	-0.88870770823259
H	3.15893218431047	-0.12261783828824	0.40647617470667
H	4.02125782054808	2.69721214873335	0.71184985704537
H	5.27319529524609	2.35237585594342	-1.37965660654761
H	5.80598723998723	1.29391115715217	-0.05455504128576
H	5.09408712343899	-1.15743529033213	-0.05340650445959
H	4.70273105795390	-3.14406771539373	-1.48070524248077
H	4.24961834887460	-2.86304340669916	-3.91773011006280
H	4.15952368412654	-0.57647954840144	-4.89761126770663
H	4.48238434728692	1.41736764991877	-3.44919766933173

H	1.41368827744137	0.92088170836590	-0.42236970431667
C	0.23298756158259	2.29573916656978	-1.43687615899773
C	0.18888795329229	2.42910710361640	-2.97694836640523
C	-0.81985896196785	1.28441310427092	-0.97096670511426
O	-0.49692722895643	0.12056408922514	-0.71830465507347
C	-3.15397078983155	0.78167989296569	-0.58821688867513
H	-0.62420812054074	3.13047087800523	-3.21909482486987
H	0.01307826660287	3.28336886054181	-0.99814760454999
H	-2.84036301578449	0.16791811540290	0.27096813983130
H	1.12790674619028	2.91372078681851	-3.28443831268049
N	-2.08163409878747	1.71046570019406	-0.89230132853325
H	-2.30479890133052	2.65266701219419	-1.19379535366672
C	-4.43784701028381	1.53958271293503	-0.22867235393671
H	-4.69589035877177	2.21175705588645	-1.06276846470474
C	-4.30265622350049	2.30524904566760	1.06475801092839
C	-3.82875630446748	3.62250384622321	1.08504674678969
C	-4.58703096205673	1.67824089081890	2.28448559411305
C	-3.62651244309965	4.29183186245146	2.29431481545765
H	-3.62475131967941	4.13987213014547	0.14369869816650
C	-4.40077793268612	2.34660567543351	3.49358808917848
H	-4.95501634186300	0.64904203463491	2.28247131957176
C	-3.91340529044851	3.65466832971005	3.50284590676710
H	-3.25599198482247	5.31860196166586	2.29100484690695
H	-4.62962440535766	1.84193377472961	4.43372586710158
H	-3.76065122036778	4.17778855972291	4.44830905937291
H	-5.24092604488711	0.79419979067414	-0.14298747542176
C	-3.40255404644890	-0.18581366085601	-1.76358690018654
O	-3.23927928860646	0.33359547609281	-2.92284691954429
O	-3.73731922203178	-1.33869739666787	-1.52436946178405
C	-0.04835321137447	1.12287912159582	-3.73341004214079
H	-0.21804337105856	1.35396981020968	-4.79710487214105
H	-0.99520802569978	0.68394161666598	-3.38177693310471
C	1.09576345380979	0.11404912964165	-3.62921654909030
H	1.92986243072857	0.44307572485638	-4.26532265938011
H	1.49701374488385	0.07284225137157	-2.60442572987173
C	0.69885001713242	-1.29529320225756	-4.03291621628941
H	0.27305281673115	-1.31452946197774	-5.04735382312307
H	1.58175732976344	-1.94815699936893	-4.03608291463456
N	-0.33463033511771	-1.83477929861915	-3.11237561942995
H	-0.33983468604688	-1.31381964757634	-2.22388210038670
C	-0.20937382165737	-3.83710300529326	0.08337986552888
C	1.02121218518868	-3.27191450853408	-0.62456310722464
C	1.01185352887675	-3.69643147941893	-2.09562133882896
C	-0.26817684353553	-3.28861514764209	-2.84308248044999
C	-1.51928224586710	-3.69459919246451	-2.05302444841378
C	-1.47299461964414	-3.29801826250758	-0.57815165437225
H	1.89481436075573	-3.31704283366093	-2.63014347338646
H	1.01115747290911	-2.16769263644118	-0.54742046041657
H	1.95224216484903	-3.61280554650633	-0.14451918474196
H	-0.19099742588234	-4.93887129037986	0.01258624858512
H	-0.20337379722749	-3.60059939528086	1.15373362966149
H	-2.40499943182328	-3.25386898636995	-2.53243497527125
H	-1.59812057994013	-4.79250936597130	-2.13250425365267
H	-1.50053546572521	-2.19917759598864	-0.48756155830651
H	-2.38025289110127	-3.66649066038399	-0.07892448938997
H	1.06332277283165	-4.79781353964689	-2.14427226315419
O	-0.29123877181853	-3.84592157148430	-4.13541801111215
H	-2.99164751156574	-2.13480392130065	-4.45968083018741
H	-1.43444127442436	-1.63038790736143	-3.72406575928852
O	-2.41453130300715	-1.36237427336188	-4.40043084314601
H	-0.28240864531856	-4.80721424139307	-4.05121097332194
H	-2.92519883232965	-0.57762734262958	-3.76901950521243

96

Intermedia S* + H2O

C	1.35603046045447	-2.44618260767964	2.92233625747058
C	0.02841181119543	-2.21255770884514	3.24928959589608
C	-0.50683112639055	-0.93402283282642	3.11237736532846
C	0.28707371715451	0.13348569186922	2.70324015808620
C	1.64122299442290	-0.09009206648204	2.39761634066681
C	2.14790107995169	-1.38334083964884	2.50169240968260
F	1.84470895179935	-3.67188965283487	2.97172556684159
F	-0.74192407935085	-3.21847784464030	3.61827082300320
F	-1.79409186934740	-0.78168411433477	3.37733310513683
I	-0.69673164904476	1.97395792023953	2.49710640742929
F	3.40964078832770	-1.65139685426008	2.17548909983841

C	2.52857772337551	1.04318554396927	1.92932360445180
O	2.52789621127619	2.12031038115958	2.48131526317009
N	3.25583740557850	0.77460133694483	0.81007518851599
C	3.76418614600455	1.86399425665672	0.00446625999422
C	5.01198684150763	1.47104118147442	-0.79780535265346
C	4.81011357395833	0.27481371155812	-1.69677689338312
C	4.86844942329430	-1.02923270882571	-1.17917478740655
C	4.66007325541932	-2.13597735946364	-2.00186355927165
C	4.41398323182901	-1.95711756162174	-3.36508669058472
C	4.37319945174910	-0.66695480529572	-3.89358940088360
C	4.55808228154107	0.44001693068152	-3.06341756738379
C	2.67017697134127	2.48268806925285	-0.89664272494835
O	2.88978539942310	3.51082243292068	-1.51169077727783
N	1.49653276480670	1.82144900250145	-0.90495445070716
H	3.17097197000364	-0.14282498212090	0.37973758876051
H	4.03860570416913	2.67644660839516	0.69109670724141
H	5.29687920277498	2.34933727637139	-1.39237553866397
H	5.82217279420891	1.26846184287844	-0.08276926666666
H	5.09796016913384	-1.17932168486916	-0.12046686338137
H	4.70970701338244	-3.14187356910083	-1.58010799507618
H	4.26798787452896	-2.82224406634059	-4.01495771658178
H	4.19270712712701	-0.51954133832849	-4.96024760820358
H	4.51389736164774	1.45003745097691	-3.47899505083467
H	1.42096436602081	0.92926860798075	-0.42973235181598
C	0.24853453531892	2.30019909730762	-1.45882498350589
C	0.21186044893141	2.43750505291086	-2.99944642223429
C	-0.80471212489073	1.28387551293805	-1.00833997273964
O	-0.47847833043430	0.11217544407083	-0.78205532215401
C	-3.14169020910313	0.76805991255466	-0.66845966067855
H	-0.59800102956343	3.14163486058057	-3.24352259073953
H	0.02301222110251	3.28552481170253	-1.01804339085884
H	-2.83343843896341	0.11225533476384	0.16009341316456
H	1.15393841802066	2.91857656220007	-3.30265311225036
N	-2.06224765837206	1.70739800844999	-0.91848325972899
H	-2.28325039193440	2.65315421416081	-1.21152902674625
C	-4.42053323679621	1.51658401738999	-0.27804789041877
H	-4.67745141445783	2.21766966631978	-1.08850368170387
C	-4.28526502658289	2.23830026417941	1.04045655588544
C	-3.82623998383614	3.55956526154875	1.10365134602526
C	-4.55862512738253	1.56890199184223	2.24021607396164
C	-3.63230937215730	4.19274378661337	2.33370555824818
H	-3.62851145229213	4.10833046882401	0.17891028542532
C	-4.37928143863787	2.20011148407063	3.47014009886112
H	-4.91416824143861	0.53587083147440	2.20541599462297
C	-3.91058517281552	3.51393439008213	3.52130689642242
H	-3.27504670833278	5.22382843244389	2.36309738611696
H	-4.59970314929324	1.66204826942268	4.39375246035811
H	-3.76380335286316	4.00832911107002	4.48305578289902
H	-5.22525331909692	0.77032180245994	-0.21889050905313
C	-3.38767868848652	-0.12721944804570	-1.90903359555381
O	-3.23303123842272	0.45442826825103	-3.02267491849158
O	-3.71297820538493	-1.30055891105341	-1.71769072262640
C	-0.02849471454282	1.13369342563464	-3.75910979196464
H	-0.17654955527941	1.36527977040255	-4.82573524782421
H	-0.99005776839707	0.70867222773531	-3.42782991375743
C	1.10476741641368	0.11470168138378	-3.63296490666905
H	1.94068329502807	0.41253747730714	-4.28119246918883
H	1.51215640969324	0.09252873278274	-2.61043051062850
C	0.69473096254696	-1.29728450397698	-4.00470565295993
H	0.24792242752547	-1.34588409693241	-5.00682173659396
H	1.56012707522509	-1.97008086683000	-3.98685339721835
N	-0.34538800597773	-1.81467798240614	-3.06024853901153
H	-0.36326901742314	-1.25923107350739	-2.18587079530025
C	-0.36781362959594	-3.75478564042390	0.18737880877120
C	0.88746152829409	-3.15703192978424	-0.44756446286895
C	0.98534235059963	-3.59599757320494	-1.91167012985997
C	-0.26237582222997	-3.27387122489107	-2.74482215270890
C	-1.55055702680538	-3.68589361494470	-2.02590464272270
C	-1.60107610368856	-3.25960073451986	-0.56014003011811
H	1.87806806673764	-3.17971308624882	-2.40115010016134
H	0.83764084508945	-2.05322476547344	-0.38029417651538
H	1.79789171768507	-3.46729656094146	0.08981536979670
H	-0.31156399555369	-4.85602579133287	0.13187949420242
H	-0.43924548265004	-3.50532615526924	1.25240129423535
H	-2.41231263267358	-3.27289384771538	-2.56994295255163
H	-1.59902563943329	-4.78686211224805	-2.08652564497374

H	-1.66144052325491	-2.16100385169633	-0.49838739980679
H	-2.52971922526660	-3.63871315328049	-0.11140430573816
H	1.09104048445271	-4.69310619408631	-1.94775821802368
O	-0.18253792879964	-3.85530109659483	-4.01852171174630
H	-3.09597525548838	-2.06997879945876	-4.55214375333198
H	-1.29255079994764	-1.65989321980390	-3.57571346091556
O	-2.44305375198223	-1.35873982063341	-4.55759718556725
H	-0.32408968264268	-4.80584186293624	-3.92981378214218
H	-2.88427200291303	-0.59888584663741	-4.02156667757002

93

Product V

C	-2.97256138271607	-3.18014597242181	-1.70758087177308
C	-1.82483791991240	-3.94066068950889	-1.52862257334597
C	-0.57171998984546	-3.35714870541250	-1.70050655042144
C	-0.45780087883304	-2.02283629954466	-2.07313499752700
C	-1.61117003859460	-1.25521774766679	-2.26680666805243
C	-2.85350396569437	-1.84039974492877	-2.06506527267063
F	-4.16310510790152	-3.71812685312212	-1.50739978484613
F	-1.92621936166181	-5.20297271232547	-1.14609726912555
F	0.49435654493229	-4.10509544431005	-1.45949678292776
I	1.45395554640038	-1.17890953841035	-2.21643046194892
F	-3.96869781597213	-1.12956115321100	-2.20249452094102
C	-1.53653104348196	0.21223657293727	-2.65174278936503
O	-1.25707349242476	0.57016675259899	-3.77698058686291
N	-1.78299228913637	1.02624085182771	-1.60499936582196
C	-1.67172383179563	2.46608436021582	-1.69203653124546
C	-3.03065662808072	3.17750950249267	-1.81367194848884
C	-3.97401524622065	2.86429652452695	-0.67819536976757
C	-4.76626749741841	1.70901231778327	-0.71693454789299
C	-5.61494262057664	1.38819468918736	0.34337510643782
C	-5.68204947081677	2.22034356722598	1.46187599529586
C	-4.89954250815513	3.37535366542233	1.50975887258475
C	-4.05308440521412	3.69539021022828	0.44754009651564
C	-0.86158933026038	3.02549471795602	-0.51061548761278
O	-1.00832611649790	4.17097773017990	-0.12029931669611
N	0.01257037482768	2.16327501327808	0.05302761557200
H	-1.97043156866001	0.57154243539063	-0.69816568951095
H	-1.09341271709842	2.68227685033488	-2.60539007107445
H	-2.83397765925852	4.25630152712155	-1.85976450506433
H	-3.47571542043205	2.86598325952774	-2.76949204528314
H	-4.71846737197595	1.05775528189215	-1.59161080001938
H	-6.22790314368907	0.48609909118909	0.29263977887731
H	-6.34711745098073	1.97320730357198	2.29124537401209
H	-4.95266241589687	4.03534937431224	2.37769232625828
H	-3.43155402571337	4.59164773528152	0.48696346318188
H	0.20729443213887	1.27062023720882	-0.39455123597351
C	0.85007057753723	2.53433907466049	1.16901424626916
C	0.11513528384527	2.67808940574888	2.52716402587778
C	1.92098549568054	1.45740718016390	1.30126449988005
O	1.73962006213017	0.32769528165910	0.86552832712314
C	4.08392508113093	0.92974289013159	2.28452291387657
H	0.80835991242422	3.24780102999704	3.16254642396912
H	1.32496974084063	3.50546348783979	0.95207456436998
H	3.71835613496474	-0.09493708256511	2.11611581418688
H	-0.76125681268638	3.32408884498426	2.35849576292141
N	3.00769491649492	1.83617314651295	1.99475306683774
H	3.07103014047769	2.80379157965829	2.32885615474624
C	5.31582143557144	1.16488357435462	1.37896177258973
H	5.68730247928267	2.18525086184917	1.56277494688128
C	4.97659688970215	0.98789886702339	-0.07951427513662
C	5.18101987873387	-0.24310212693040	-0.71187681718096
C	4.41677893581493	2.03969822086635	-0.81744793458682
C	4.84977680944156	-0.41821544635242	-2.05661567583369
H	5.61544166627479	-1.07189132771130	-0.14752271657549
C	4.06971880507415	1.86523089990558	-2.15655571716263
H	4.25652950069713	3.00683437959960	-0.33486636435184
C	4.29300810556649	0.63687785493177	-2.78342496029892
H	5.03048636446104	-1.38072687545566	-2.53948403520226
H	3.63508759109075	2.69371811813177	-2.71837705784330
H	4.03806679231641	0.50463638878381	-3.83688659968696
H	6.10242647173865	0.45854673025379	1.67626057007519
C	4.46744399112913	1.04713107371133	3.74980123244544
O	4.02009438893621	1.85822508880504	4.52156179211565
O	5.38504300198926	0.15090203900332	4.09104932692358
C	-0.28045632538761	1.37878469836843	3.26230326973693

H	0.00390475962890	1.48480633966433	4.31942203485389
H	0.31169917055199	0.52506700246173	2.89301193476041
C	-1.76517392101323	1.00655969666036	3.21787062179944
H	-1.91771030322304	0.09809559984754	3.82490394172746
H	-2.36219066394742	1.80602848733432	3.68803652002955
C	-2.31244818681281	0.77358470098332	1.81389309268440
H	-3.37944088474629	0.51526714618600	1.85790061177284
H	-2.26557860488421	1.71699008240468	1.24772953551511
N	-1.61216210707622	-0.25267601034847	1.03946266416387
H	3.26736539759909	3.70820297076188	4.15497315393240
H	-0.61491952083701	-0.03981631311300	0.97073008291967
C	-2.12792231653324	-4.21043116997564	2.52365103113618
C	-3.29162048132863	-3.59433163527406	1.75190282096584
C	-3.17416566506566	-2.06976741803118	1.68985514382323
C	-1.76203599105457	-1.60480388959867	1.42292853525202
C	-0.70159830805876	-2.43049030576012	1.48430318112987
C	-0.80407770957751	-3.89179292856138	1.83152429171355
H	-3.83338403665153	-1.65834042205907	0.90427258910547
H	-3.29715180534404	-4.01117208053925	0.73283843393465
H	-4.25464343603403	-3.87224838039224	2.20395478380077
H	-2.11109984230611	-3.79710897492515	3.54655996423187
H	-2.26056372488843	-5.29839129182596	2.61549556894798
H	2.45068350557709	4.93018845444315	3.68745434767702
H	0.28938773617916	-2.01572384450611	1.26241936352626
H	-0.69943009766252	-4.51128281523082	0.92127388854844
H	0.04213837330002	-4.17609299974010	2.47631430567246
H	-3.52737193495345	-1.62995923649864	2.63936432800461
O	3.14295035910713	4.31833343686416	3.41058621224570
H	5.61127709139901	0.27796168240999	5.02805716648206
