# Supplementary material for: Challenges in Mechanistic Investigation of a Flexible Aminocatalyst as Demonstrated through Enamine Formation
Source: ChemistryOpen. 2025 Jun 25;14(9):e202500116. doi: 10.1002/open.202500116 (PMC12409831; doi:10.1002/open.202500116)
Supplement: Supplementary file 1 — Supplementary Material [file OPEN-14-e202500116-s001.zip › SI-10.pdf]

Supporting Information

Problems in Reaction Mechanism Search for Flexible Aminocatalyst on Example of Enamine Formation

Irina Osadchuk,* Tõnis Kanger*

Table of Contents

Scheme S1. Formation of enamine 2

Figure S1. Conformers of the catalyst 2

Table S1. Energies and Boltzmann distribution of the catalyst's conformers 2

Table S2. Non-covalent interactions in the main conformer of the catalyst 3

Figure S2. Catalyst reorganization 4

Table S3. Catalyst reorganization energies 5

Table S4. Non-covalent interactions in intermediate O (reacting system) 6

Table S5. Reorganization energies of the catalyst in the entire model 7

Table S6. Enamine formation without presence of water 8

Table S7. Enamine formation with presence of water 8

Table S8. Non-covalent interactions in intermediate T. 9

Table S9. Non-covalent interactions in intermediate T*. 10

Table S10. An alternative pathway for enamine formation without presence of water 11

Table S11. An alternative pathway for enamine formation with presence of water 11

Table S12. Non-covalent interactions in intermediate U*. 12

Table S13. Non-covalent interactions in product V. 14

Table S14. Recalculated TSs using RI and SMD-X/def2-TZVPP//CPCM-X/def2-SVP level of theory where X is M06-2X-D3, B3LYP-D4 or PBE0-D4. 15

Table S15. Recalculated TSs using RI and SMD-X//CPCM-M06-2X/def2-SVP level of theory where X is B2PLYP-D3BJ/def2-TZVPP, B2PLYP-D3BJ/def2-QZVPP, DSD-PBEP86-D3BJ/def2-TZVPP and DSD-PBEP86-D3BJ/def2-QZVPP. 15

Table S16. Recalculated TSs using RI and X-M06-2X/def2-TZVPP//CPCM-M06-2X/def2-SVP level of theory where X is continuum solvation model. 16

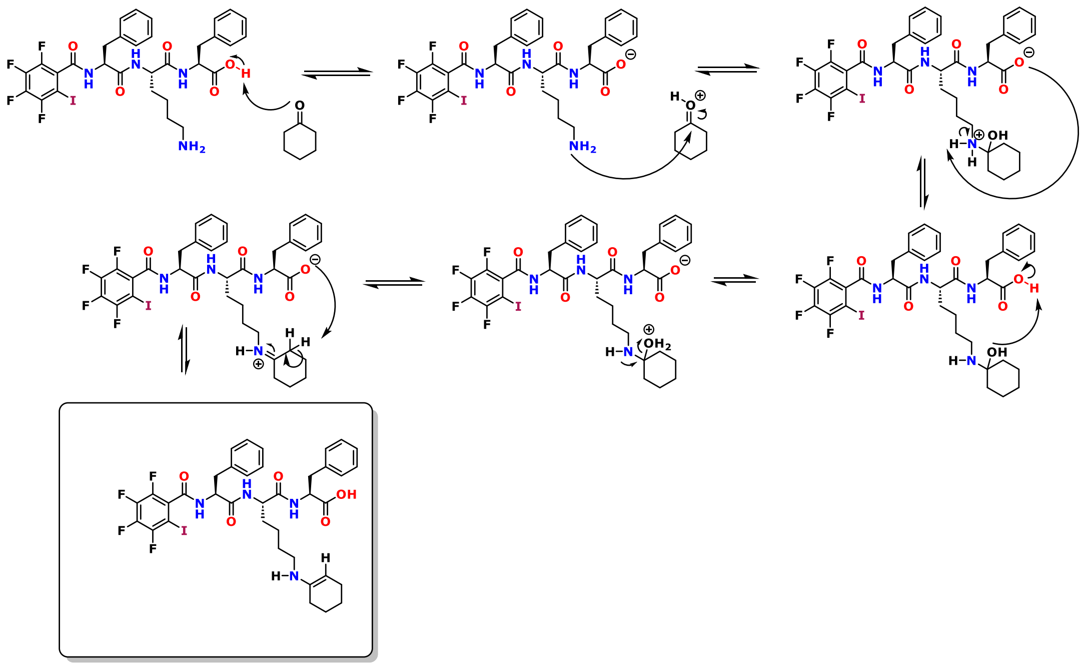

Scheme S1. Formation of enamine

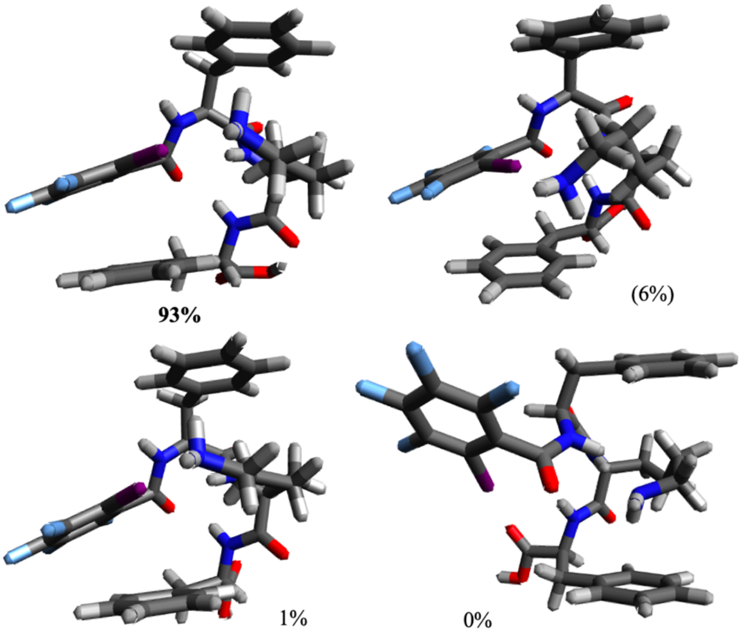

Figure S1. Conformers of the catalyst

Table S1. Energies and Boltzmann distribution of the catalyst's conformers

Conformer	Gibbs free energy, Hartree	Electronic energy, Hartree	Relative energy, kcal mol^{-1}	Distribution, %
1	0,53082204	-2493,47424041	0	92,9
2	0,53091348	-2493,47196662	1,5	6,3
3	0,53052702	-2493,46970126	2,7	0,7
4	0,52966901	-2493,46606378	4,4	0

Table S2. Non-covalent interactions in the main conformer of the catalyst

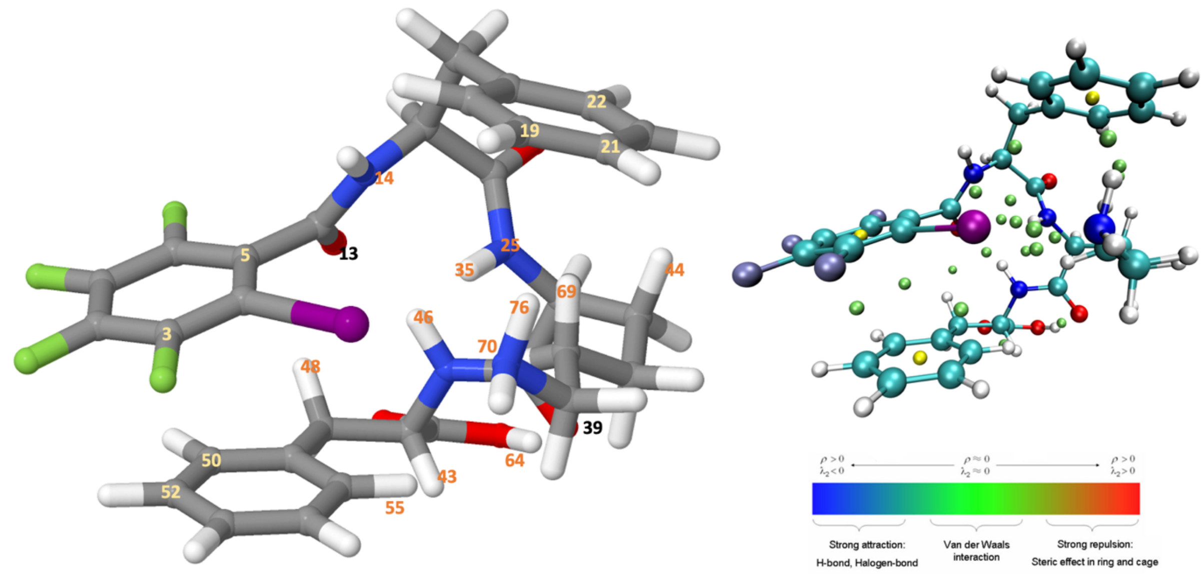

Atoms	Type of critical point	Density of all electrons (ρ(r))	Sign(λ_2(r))*ρ(r)
64(H)--39(O)	(3,-1)	0,042556	-0,042556
43(H)--55(H)	(3,-1)	0,012096	-0,012096
48(H)--13(O)	(3,-1)	0,008717	-0,008717
	(3,-1)	0,016784	-0,016784
46(H)--13(O)	(3,-1)	0,020943	-0,020943
13(O)--25(N)	(3,-1)	0,010119	-0,010119
50(C)--5(C)	(3,-1)	0,004455	-0,004455
70(H)--35(H)	(3,-1)	0,012297	-0,012297
	(3,-1)	0,006750	-0,006750
25(N)--14(N)	(3,-1)	0,015481	-0,015481
44(H)--22(C)	(3,-1)	0,003228	-0,003228
52(C)--3(C)	(3,-1)	0,008290	-0,008290
	(3,-1)	0,010785	-0,010785
	(3,-1)	0,005119	-0,005119
69(H)--21(C)	(3,-1)	0,317929	-0,317929
	(3,-1)	0,007595	-0,007595
	(3,-1)	0,010164	-0,010164
	(3,-1)	0,117041	-0,117041
	(3,-1)	0,025196	-0,025196
	(3,-1)	0,004280	-0,004280
76(H)--19(C)	(3,-1)	0,007219	-0,007219

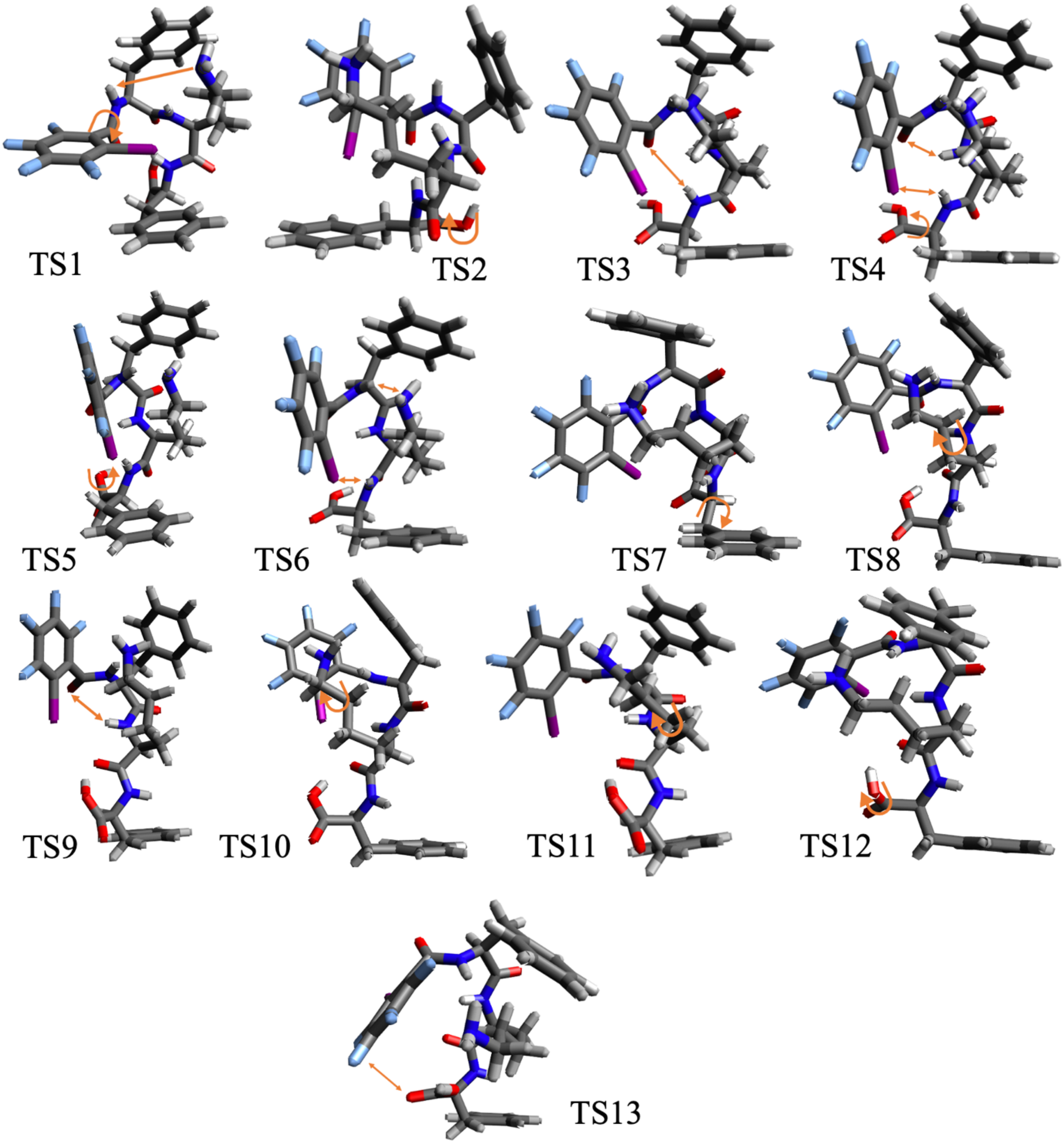

Figure S2. Catalyst reorganization

Table S3. Catalyst reorganization energies

Conformer	Electronic energy, Hartree	Gibbs free energy, Hartree	Relative energy, kcal mol^{-1}	Imaginary mode(s)
A_s	-2492,22643717	0,52901929	0	
$TS1_s$	-2492,22062262	0,52892372	3,6	-14.61
B_s	-2492,22604929	0,52899665	0,2	
$TS2_s$	-2492,21310778	0,52710922	7,2	-335.48
C_s	-2492,22135430	0,52670172	1,7	
D_s	-2492,21396841	0,52886278	7,7	
$TS3_s$	-2492,21394882	0,53045175	8,7	-11.92
E_s	-2492,21288108	0,52947898	8,8	
$TS4_s$	-2492,21273175	0,53051847	9,5	-14.57
F_s	-2492,21280959	0,52962922	8,9	
$TS5_s$	-2492,19855492	0,52724271	16,4	-489.94
G_s	-2492,21584936	0,52921958	6,8	
$TS6_s$	-2492,21513000	0,53060994	8,1	-18.85
H_s	-2492,21825023	0,53092005	6,3	
$TS7_s$	-2492,21446587	0,53144486	9	-20.97
I_s	-2492,22111923	0,52917316	3,4	
$TS8_s$	-2492,22016482	0,52973135	4,4	-99.22
J_s	-2492,22375197	0,52928120	1,8	
$TS9_s$	-2492,22408843	0,52871347	1,3	-31.51
K_s	-2492,22496123	0,52707660	-0,3	
$TS10_s$	-2492,22056867	0,52858070	3,4	-53.99
L_s	-2492,22093825	0,52756428	2,5	
$TS11_s$	-2492,22129951	0,52868478	3	-18.16
M_s	-2492,22130142	0,52835399	2,8	
$TS12_s$	-2492,20603343	0,52708764	11,6	-349.09
N_s	-2492,21874266	0,52798087	4,2	
$TS13_s$	-2492,21330430	0,53055867	9,2	-20.98
O_s	-2492,21438174	0,53110987	8,9	

Table S4. Non-covalent interactions in intermediate O (reacting system)

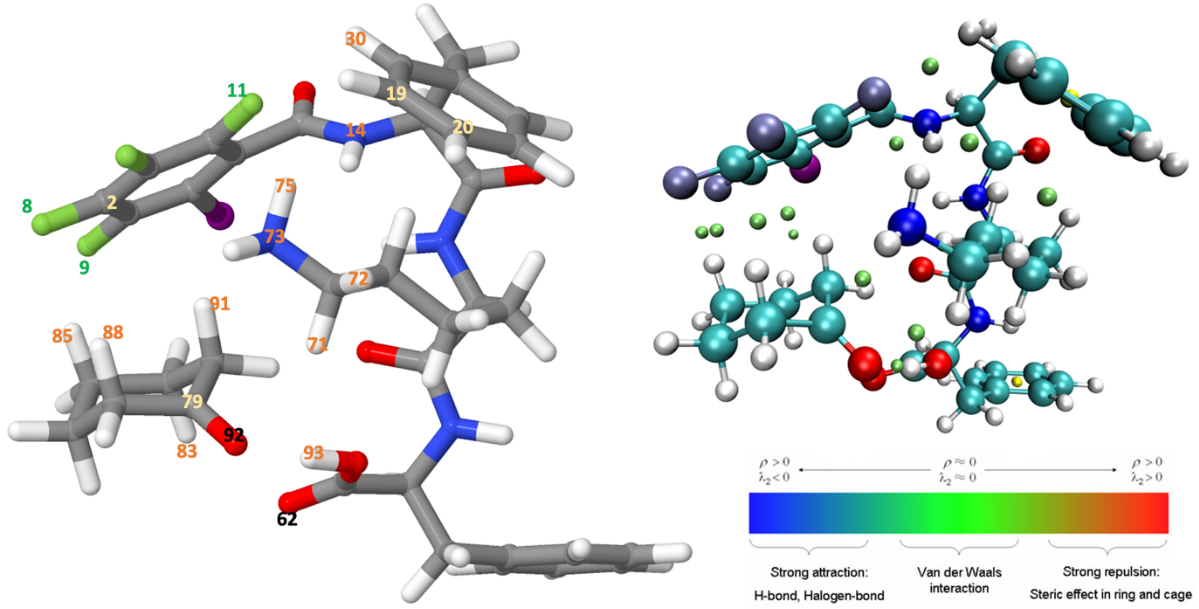

Atoms	Type of critical point	Density of all electrons (ρ(r))	Sign(λ_2(r))*ρ(r)
62(O)--83(H)	(3,-1)	0,009117	-0,009117
	(3,-1)	0,121932	-0,121932
	(3,-1)	0,002412	-0,002412
14(N)--11(F)	(3,-1)	0,013394	-0,013394
93(H)--92(O)	(3,-1)	0,058619	-0,058619
20(C)--72(H)	(3,-1)	0,005281	-0,005281
30(H)--11(F)	(3,-1)	0,010403	-0,010403
71(H)--92(O)	(3,-1)	0,012383	-0,012383
19(C)--75(H)	(3,-1)	0,004757	-0,004757
11(F)--75(H)	(3,-1)	0,012317	-0,012317
73(N)--79(C)	(3,-1)	0,009019	-0,009019
91(H)--2(C)	(3,-1)	0,007172	-0,007172
9(F)--85(H)	(3,-1)	0,010512	-0,010512
2(C)--88(H)	(3,-1)	0,005378	-0,005378
85(H)--8(F)	(3,-1)	0,011383	-0,011383

Table S5. Reorganization energies of the catalyst in the entire model

Conformer	Gibbs free energy, Hartree	gCP correction, Hartree	Electronic energy, Hartree	Relative energy, kcal mol^{-1}	Imaginary mode(s)
Reagent	-2802,116520346	0,076909073	0,67593111	0,0	
TS0	-2802,114875403	0,076767052	0,67586437	1,1	-101.70
A	-2802,121784945	0,076660909	0,67701446	-2,5	
TS1	-2802,108763025	0,075950207	0,67463466	4,7	-15.67
B	-2802,110257328	0,076733545	0,67276145	2,1	
TS2	-2802,096017266	0,076729697	0,67193961	10,5	-239.78
C	-2802,099474899	0,076521356	0,67146274	8,1	
D	-2802,106145743	0,078669942	0,67363025	4,0	
TS3	-2802,101115634	0,076469055	0,67555980	9,7	-10.47
E	-2802,108927414	0,077386714	0,67386150	3,2	
TS4	-2802,108195207	0,077479121	0,67541588	4,5	-8.99
F	-2802,111619744	0,077947045	0,67488050	1,8	
TS5	-2802,096489806	0,076892095	0,67342990	11,0	-119.18
G	-2802,115678227	0,076139702	0,67579693	0,9	
TS6	-2802,097954947	0,075581387	0,67451268	11.6	-13.00; -3.04
H	-2802,105882953	0,077522335	0,67436048	5,3	
TS7	-2802,103001253	0,077588572	0,67581538	8,0	-13.81
I	-2802,109489729	0,07790862	0,67325597	2,1	
TS8	-2802,108735829	0,07793316	0,67309314	2,5	-104.32
J	-2802,112073403	0,07799539	0,67300800	0,3	
TS9	-2802,108760207	0,07742187	0,67258757	2,4	-11.68
K	-2802,117657557	0,07628424	0,67244525	-2,5	
TS10	-2802,117702602	0,07623788	0,67325739	-2,0	-16.18
L	-2802,119424354	0,07669326	0,67429341	-2,7	
TS11	-2802,109582605	0,07574177	0,67272670	3,1	-18.19
M	-2802,109542949	0,07647168	0,67433988	3,7	
TS12	-2802,102437689	0,07602297	0,67360575	7,9	-172.35
N	-2802,119658197	0,07648787	0,67617729	-1,6	
TS13	-2802,119091387	0,07653651	0,67754986	-0,4	-24.06
O	-2802,119035081	0,07662740	0,67722161	-0,6	

Table S6. Enamine formation without presence of water

Conformer	Electronic energy, Hartree	gCP correction, Hartree	Gibbs free energy, Hartree	Relative energy, kcal mol^{-1}	Imaginary mode(s)
TS14	-2802,108545065	0,07719541	0,68095426	8,3	-135.38
P	-2802,113502816	0,07776534	0,68151207	5,9	
TS15	-2802,111593950	0,07799696	0,68252938	7,9	-14.16
R	-2802,110977212	0,07823098	0,68186675	8,0	
TS16	-2802,060040742	0,07874383	0,67718705	37,4	-1520.80
S	-2802,114141177	0,07727269	0,68155354	5,2	
TS17	-2802,088718456	0,07861396	0,67861276	20,2	-248.28
T	-2802,097418795	0,07791149	0,67733476	13,5	
TS18	-2802,066386297	0,07871356	0,67183999	30,0	-1210.73
U	-2802,102489102	0,07734340	0,67419441	8,0	
TS18-1	-2802,069077757	0,07903471	0,67201791	27,3	-1189.85
TS18-2	-2802,068183236	0,07754200	0,67074226	27,1	-1238.66
TS18-3	-2802,050084796	0,08082726	0,67162912	39,0	-649.87
TS18-4	-2802,068195989	0,07753994	0,67074063	27,1	-1238.68
TS18-5	-2802,017001425	0,07904225	0,67556719	62,2	-318.74
TS18-6	-2802,023144099	0,08086327	0,67482315	57,9	-509.83

Table S7. Enamine formation with presence of water

Conformer	Electronic energy, Hartree	gCP correction, Hartree	Gibbs free energy, Hartree	Relative energy, kcal mol^{-1}	Imaginary mode(s)
R	-2878,563035133	0,08116907	0,70376240	0,0	
TS16	-2878,541763433	0,08202369	0,69948323	11,2	-1375.79
S	-2878,567004385	0,08059600	0,70392763	-2,7	
TS17	-2878,533513254	0,08023281	0,69974899	15,4	-216.74
T	-2878,548899040	0,08222299	0,69798605	5,9	
TS18	-2878,516038668	0,08349998	0,69318708	24,3	-1174.45
U	-2878,547970567	0,08144709	0,69914008	6,7	

Table S8. Non-covalent interactions in intermediate T.

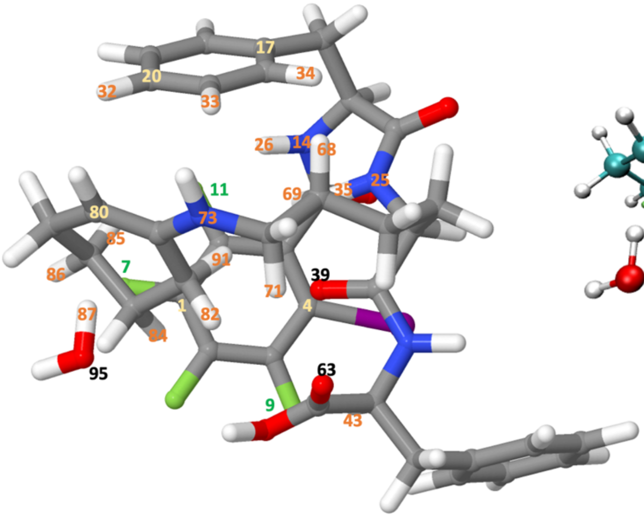

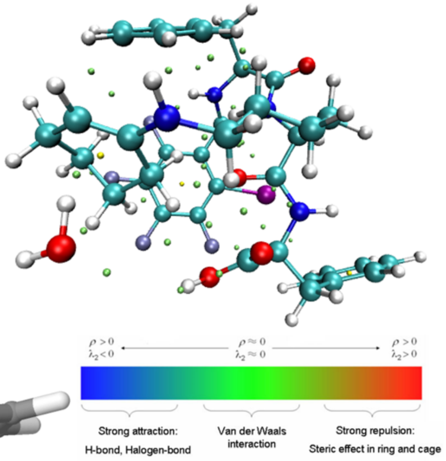

Atoms	Type of critical point	Density of all electrons (ρ(r))	Sign(λ_2(r))*ρ(r)	Atoms
108		(3,-1)	0,005930	-0,005930
115		(3,-1)	0,015679	-0,015679
118		(3,-1)	0,007594	-0,007594
123		(3,-1)	0,005920	-0,005920
142	43(H)--9(F)	(3,-1)	0,007893	-0,007893
154	25(N)--14(N)	(3,-1)	0,018940	-0,018940
155	4(C)--39(O)	(3,-1)	0,009313	-0,009313
158	35(H)--39(O)	(3,-1)	0,025245	-0,025245
160	66(H)--63(O)	(3,-1)	0,010069	-0,010069
164	25(N)--69(H)	(3,-1)	0,008654	-0,008654
168	25(N)--34(H)	(3,-1)	0,006637	-0,006637
171	39(O)--69(H)	(3,-1)	0,009413	-0,009413
183	34(H)--68(H)	(3,-1)	0,005714	-0,005714
184	14(N)--17(C)	(3,-1)	0,014998	-0,014998
187	63(O)--71(H)	(3,-1)	0,012114	-0,012114
189	39(O)--91(H)	(3,-1)	0,011947	-0,011947
190	26(H)--11(F)	(3,-1)	0,016817	-0,016817
196	69(H)--91(H)	(3,-1)	0,009778	-0,009778
200	63(O)--82(H)	(3,-1)	0,007396	-0,007396
211	68(H)--33(H)	(3,-1)	0,005632	-0,005632
214	71(H)--82(H)	(3,-1)	0,013055	-0,013055
223	1(C)--84(H)	(3,-1)	0,007034	-0,007034
227	82(H)--95(O)	(3,-1)	0,010583	-0,010583
229	33(H)--73(N)	(3,-1)	0,009364	-0,009364
242	20(C)--85(H)	(3,-1)	0,003614	-0,003614
248	7(F)--86(H)	(3,-1)	0,007449	-0,007449
255	32(H)--80(C)	(3,-1)	0,006752	-0,006752
256	80(C)--87(H)	(3,-1)	0,020825	-0,020825

Table S9. Non-covalent interactions in intermediate T*.

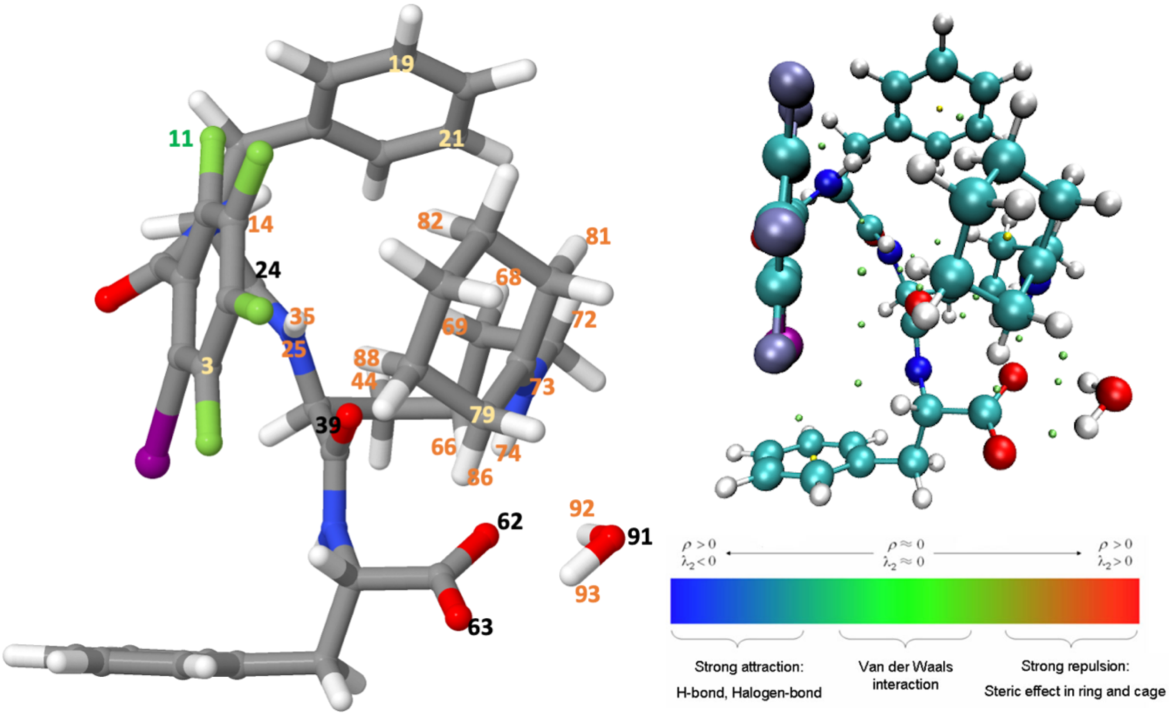

Atoms	Type of critical point	Density of all electrons (ρ(r))	Sign(λ2(r))*ρ(r)	Atoms
116	3(C)--88(H)	(3,-1)	0,008040	-0,008035
118	79(C)--91(O)	(3,-1)	0,009040	-0,009037
124	93(H)--63(O)	(3,-1)	0,022300	-0,022251
129	82(H)--19(C)	(3,-1)	0,000930	-0,000930
130	86(H)--63(O)	(3,-1)	0,011100	-0,011087
131	81(H)--72(H)	(3,-1)	0,015800	-0,015820
139	88(H)--39(O)	(3,-1)	0,007180	-0,007179
141	79(C)--39(O)	(3,-1)	0,004650	-0,004652
145	92(H)--62(O)	(3,-1)	0,025800	-0,025758
150	11(F)--14(N)	(3,-1)	0,014200	-0,014225
155	73(N)--39(O)	(3,-1)	0,008740	-0,008741
156	74(H)--62(O)	(3,-1)	0,055100	-0,055112
161		(3,-1)	0,006400	-0,006402
169		(3,-1)	0,007680	-0,007677
178	39(O)--69(H)	(3,-1)	0,007910	-0,007906
179		(3,-1)	0,008290	-0,008289
182		(3,-1)	0,013200	-0,013232
184	39(O)--35(H)	(3,-1)	0,023100	-0,023132
191	62(O)--66(H)	(3,-1)	0,018000	-0,017951
195	14(N)--25(N)	(3,-1)	0,019700	-0,019661
201	21(C)--68(H)	(3,-1)	0,005570	-0,005573
203	69(H)--25(N)	(3,-1)	0,010300	-0,010252
241	44(H)--24(O)	(3,-1)	0,009000	-0,008998

Table S10. An alternative pathway for enamine formation without presence of water

Conformer	Electronic energy, Hartree	gCP correction, Hartree	Gibbs free energy, Hartree	Relative energy, kcal mol^{-1}	Imaginary mode(s)
O*	-2802,114529	0,0757052	0,6752066	0,8	
TS11-2*	-2802,103785	0,0766048	0,6744582	7,1	-32.52
O2*	-2802,106877	0,0771538	0,6744612	5,1	
TS14*	-2802,102410	0,0778863	0,6766741	9,3	-175.11
R*	-2802,125835	0,0790926	0,6833552	-1,2	
TS16*	-2802,064509	0,0788043	0,6762333	34,0	-1506.98
S*	-2802,118152	0,0776311	0,678661	1,1	
TS17*	-2802,102661	0,0780934	0,6785076	11,1	-191.73
T*	-2802,116944	0,0776123	0,675161	-0,3	
TS18*	-2802,086047	0,0781593	0,67254763	17,8	-1213.05
U*	-2802,106052	0,0787805	0,6758001	7,7	
V	-2802,112598	0,0764057	0,6750191	1.6	

Table S11. An alternative pathway for enamine formation with presence of water

Conformer	Electronic energy, Hartree	gCP correction, Hartree	Gibbs free energy, Hartree	Relative energy, kcal mol^{-1}	Imaginary mode(s)
R*	-2878,56770837	0,08247285	0,70339688	-2,3	
TS16*	-2878,55940262	0,08302558	0,70088406	1,6	-883.23
S*	-2878,56437078	0,08276718	0,70405624	0,3	

Table S12. Non-covalent interactions in intermediate U*.

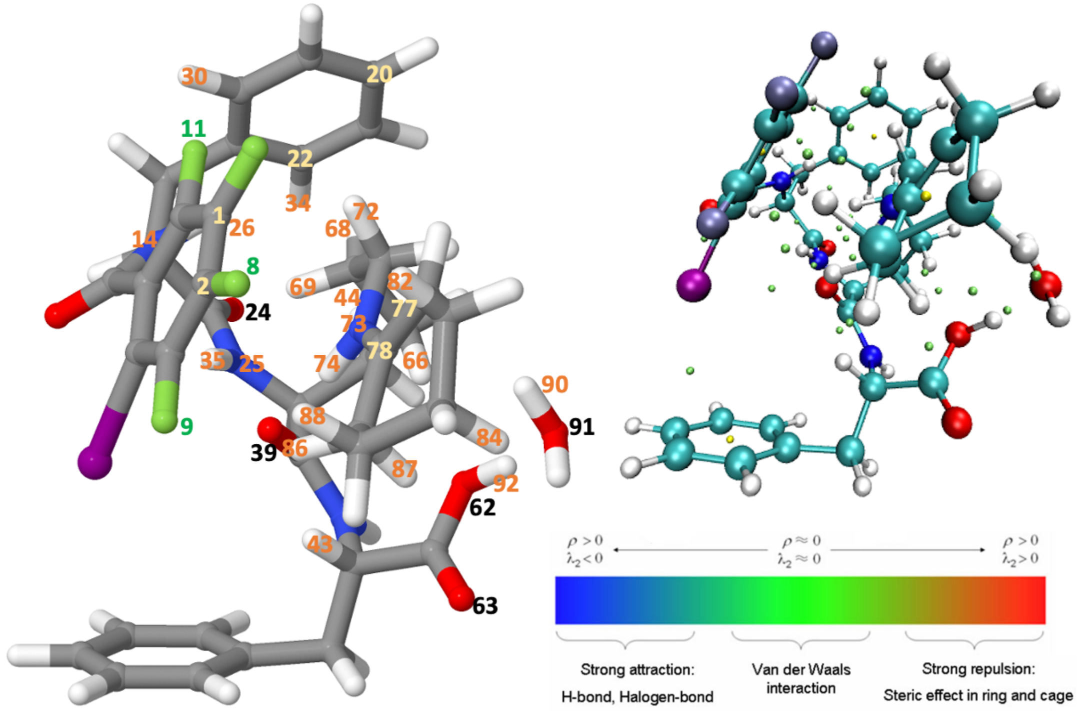

Atoms	Type of critical point	Density of all electrons (ρ(r))	Sign(λ2(r))*ρ(r)	Atoms
98	82(H)--8(F)	(3,-1)	0,004608	-0,004608
102	88(H)--8(F)	(3,-1)	0,010758	-0,010758
109	88(H)--9(F)	(3,-1)	0,006485	-0,006485
110	84(H)--91(O)	(3,-1)	0,005222	-0,005222
119	77(C)--90(H)	(3,-1)	0,014211	-0,014211
121	2(C)--78(C)	(3,-1)	0,008049	-0,008049
129	9(F)--86(H)	(3,-1)	0,008718	-0,008718
132	87(H)--91(O)	(3,-1)	0,008183	-0,008183
136	1(C)--72(H)	(3,-1)	0,007004	-0,007004
140	87(H)--63(O)	(3,-1)	0,008873	-0,008873
141	91(O)--92(H)	(3,-1)	0,057726	-0,057726
148	86(H)--43(H)	(3,-1)	0,003349	-0,003349
157	73(N)--62(O)	(3,-1)	0,004884	-0,004884
163	74(H)--39(O)	(3,-1)	0,025895	-0,025895
170	11(F)--26(H)	(3,-1)	0,018480	-0,018480
171	11(F)--30(H)	(3,-1)	0,005563	-0,005563
177	62(O)--66(H)	(3,-1)	0,014817	-0,014817
180	43(H)--39(O)	(3,-1)	0,020629	-0,020629
181		(3,-1)	0,010453	-0,010453
182		(3,-1)	0,006381	-0,006381
189	69(H)--39(O)	(3,-1)	0,010583	-0,010583
190		(3,-1)	0,014098	-0,014098
193	69(H)--26(H)	(3,-1)	0,012063	-0,012063

200	68(H)--20(C)	(3,-1)	0,006974	-0,006974
214	69(H)--25(N)	(3,-1)	0,012910	-0,012910
215	39(O)--35(H)	(3,-1)	0,027434	-0,027434
221	14(N)--25(N)	(3,-1)	0,020735	-0,020735
249	44(H)--22(C)	(3,-1)	0,003476	-0,003476
256	44(H)--24(O)	(3,-1)	0,009790	-0,009790
260	34(H)--24(O)	(3,-1)	0,011141	-0,011141

Table S13. Non-covalent interactions in product V.

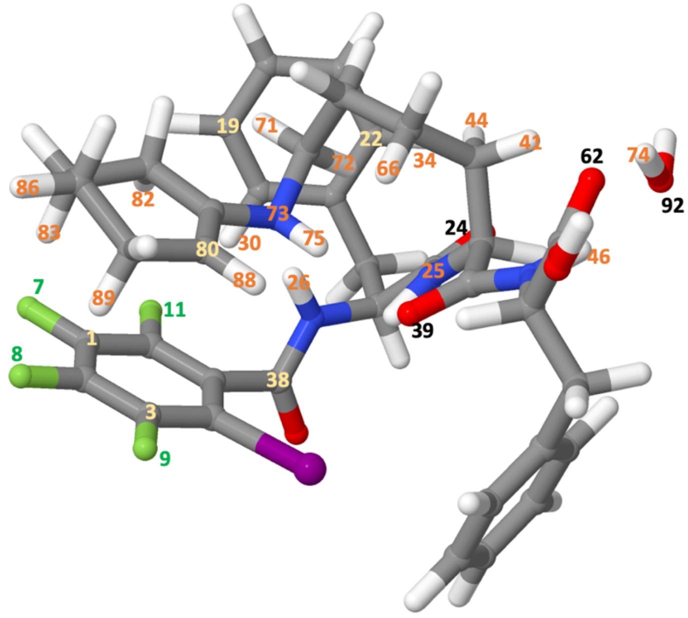

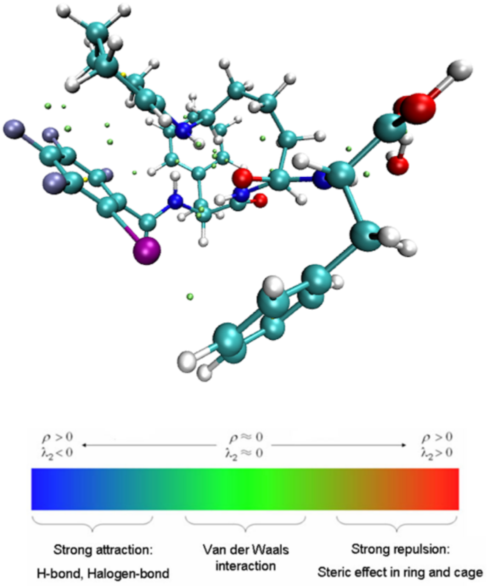

Atoms	Type of critical point	Density of all electrons (ρ(r))	Sign(λ_2(r))*ρ(r)	Atoms
96	8(F)--89(H)	(3,-1)	0,008064	-0,008064
103	83(H)--7(F)	(3,-1)	0,009720	-0,009720
104	89(H)--9(F)	(3,-1)	0,005398	-0,005398
118	3(C)--80(C)	(3,-1)	0,006859	-0,006859
121	1(C)--82(H)	(3,-1)	0,004216	-0,004216
136	88(H)--39(O)	(3,-1)	0,004858	-0,004858
148	75(H)--39(O)	(3,-1)	0,011427	-0,011427
153		(3,-1)	0,008297	-0,008297
155	73(N)--26(H)	(3,-1)	0,030805	-0,030805
156	11(F)--30(H)	(3,-1)	0,008929	-0,008929
158	71(H)--19(C)	(3,-1)	0,006891	-0,006891
166	66(H)--38(C)	(3,-1)	0,012918	-0,012918
170	75(H)--25(N)	(3,-1)	0,012149	-0,012149
176		(3,-1)	0,008531	-0,008531
180		(3,-1)	0,004648	-0,004648
203	72(H)--25(N)	(3,-1)	0,011146	-0,011146
208	72(H)--22(C)	(3,-1)	0,007969	-0,007969
220	62(O)--74(H)	(3,-1)	0,020597	-0,020597
233	46(H)--92(O)	(3,-1)	0,030061	-0,030061
234	41(H)--92(O)	(3,-1)	0,008798	-0,008798
240	44(H)--24(O)	(3,-1)	0,009004	-0,009004
247	34(H)--24(O)	(3,-1)	0,009718	-0,009718

Table S14. Recalculated TSs using RI and SMD-X/def2-TZVPP//CPCM-X/def2-SVP level of theory where X is M06-2X-D3, B3LYP-D4 or PBE0-D4.

Method	Structure	Electronic energy, Hartree	gCP correction, Hartree	Gibbs free energy, Hartree	Relative energy, kcal mol^{-1}
PBE0-D4	A	-2800,49851119	0,07640928	0,67392975	0,0
	TS2	-2800,47548697	0,07625172	0,66936259	11,7
	TS5	-2800,48509895	0,07504186	0,66660316	4,7
	TS6				
	TS17	-2800,48623294	0,07785296	0,67647459	8,4
	TS18	-2800,47542366	0,07777287	0,66948824	10,8
B3LYP-D4	A	-2801,99219877	0,07587182	0,66796483	0,0
	TS2	-2801,96935382	0,07575886	0,66388110	11,8
	TS5	-2801,97266647	0,07577090	0,66693485	11,7
	TS6				
	TS17	-2801,97647757	0,07699691	0,66903419	9,8
	TS18	-2801,96486582	0,07729706	0,66383011	13,7
M06-2X-D3	A	-2802,13167563	0,07685415	0,67591405	0,0
	TS2	-2802,10922511	0,07683106	0,67194613	11,6
	TS5	-2802,10997338	0,07698027	0,67361346	12,1
	TS6	-2802,11006851	0,07563745	0,67453257	13.4
	TS17	-2802,11620992	0,07816351	0,67879999	10,7
	TS18	-2802,10011674	0,07826299	0,67278318	16,9

Table S15. Recalculated TSs using RI and SMD-X//CPCM-M06-2X/def2-SVP level of theory where X is B2PLYP-D3BJ/def2-TZVPP, B2PLYP-D3BJ/def2-QZVPP, DSD-PBEP86-D3BJ/def2-TZVPP and DSD-PBEP86-D3BJ/def2-QZVPP.

	B2PLYP-D3BJ		DSD-PBEP86-D3BJ	
	def2-TZVPP	def2-QZVPP	def2-TZVPP	def2-QZVPP
A	-2801,12405287533	-2801,53462672725	-2798,51396376595	-2799,09460389197
TS2	-2801,10053928265	-2801,51180248731	-2798,48979073495	-2799,07130497064
TS5	-2801,10069377416	-2801,51153983381	-2798,49033339291	-2799,07127735829
TS6	-2801,10238371231	-2801,51389772686	-2798,49152757144	-2799,07335923614
TS17	-2801,11038831511	-2801,52179005358	-2798,49969810495	-2799,08148297232
TS18	-2801,09513974555	-2801,50675748330	-2798,48280054218	-2799,06492458382

Table S16. Recalculated TSs using RI and X-M06-2X/def2-TZVPP//CPCM-M06-2X/def2-SVP level of theory where X is continuum solvation model.

	SMD18+gCP	SMD18	CPCM
A	-2802,11556731907	-2802,09605844934	-2802,03865824632
TS2	-2802,09545700145	-2802,07445980262	-2802,01872729610
TS5	-2802,09484731237	-2802,07395140191	-2802,01795540902
TS6	-2802,09681552892	-2802,07507379560	-2802,02123411638
TS17	-2802,10210545295	-2802,08158031496	-2802,02401234447
TS18	-2802,08555479550	-2802,06438518730	-2802,00739550089
